# Asymmetric Synthesis of Chiral 2-Cyclohexenones with Quaternary Stereocenters via Ene-Reductase Catalyzed Desymmetrization of 2,5-Cyclohexadienones

**DOI:** 10.1021/acscatal.4c00276

**Published:** 2024-04-24

**Authors:** Michael Friess, Amit Singh Sahrawat, Bianca Kerschbaumer, Silvia Wallner, Ana Torvisco, Roland Fischer, Karl Gruber, Peter Macheroux, Rolf Breinbauer

**Affiliations:** † Institute of Organic Chemistry, Graz University of Technology, Stremayrgasse 9, 8010 Graz, Austria; ‡ Institute of Molecular Biosciences, University of Graz, Humboldtstraße 50, 8010 Graz, Austria; § Institute of Biochemistry, Graz University of Technology, Petersgasse 10-12, 8010 Graz, Austria; ∥ Institute of Inorganic Chemistry, Graz University of Technology, Stremayrgasse 9, 8010 Graz, Austria; ⊥ BIOTECHMED Graz, 8010 Graz, Austria

**Keywords:** biocatalysis, desymmetrization, dienone, enantioselective synthesis, ene-reductase, flavinmononucleotide (FMN), hydrogenation, quaternary stereogenic center

## Abstract

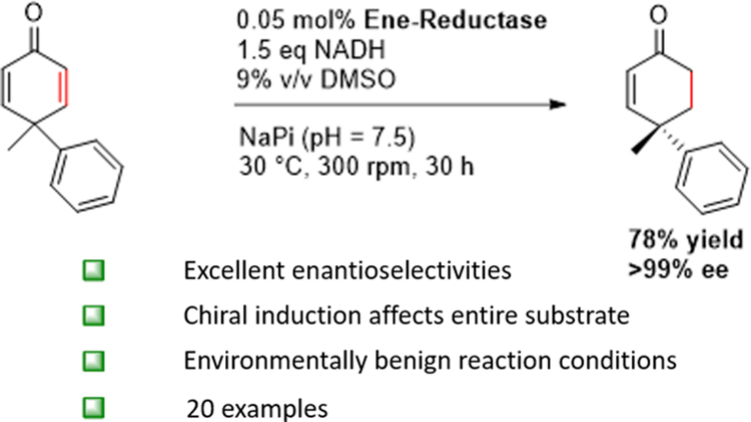

Stereoselective synthesis of quaternary stereocenters represents a significant challenge in organic chemistry. Herein, we describe the use of ene-reductases OPR3 and YqjM for the efficient asymmetric synthesis of chiral 4,4-disubstituted 2-cyclohexenones via desymmetrizing hydrogenation of prochiral 4,4-disubstituted 2,5-cyclohexadienones. This transformation breaks the symmetry of the cyclohexadienone substrates, generating valuable quaternary stereocenters with high enantioselectivities (ee, up to >99%). The mechanistic causes for the observed high enantioselectivities were investigated both experimentally (stopped-flow kinetics) as well as theoretically (quantum mechanics/molecular mechanics calculations). The synthetic potential of the resulting chiral enones was demonstrated in several diversification reactions in which the stereochemical integrity of the quaternary stereocenter could be preserved.

## Introduction

**Scheme 1:**
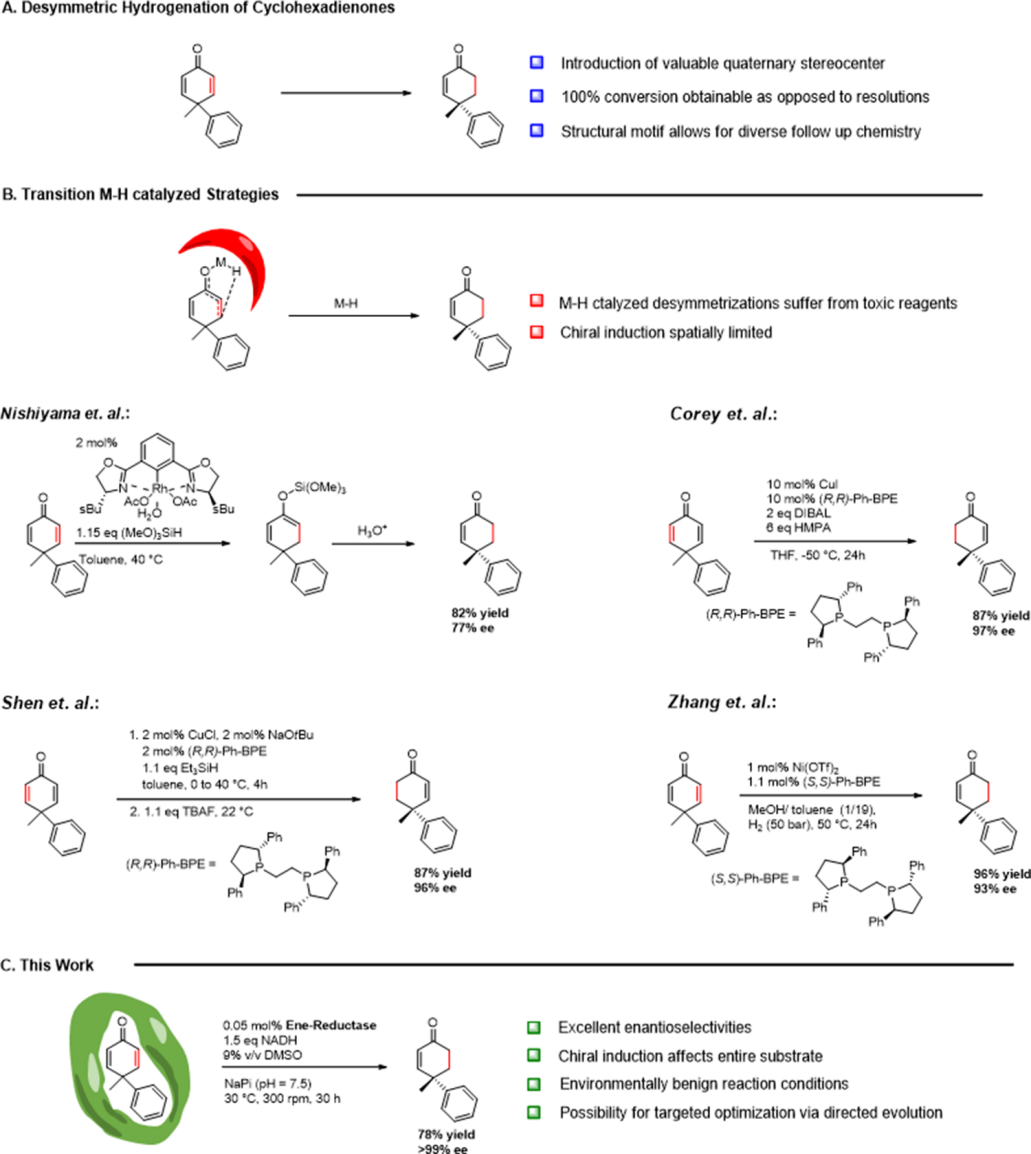
Strategies for the Desymmetrizing Hydrogenation of Cyclohexadienones; (A) Principle of Desymmetrizing Hydrogenation; (B) Previous Work with Transition Metal-Catalyzed Reactions; (C) This Work: Employing Ene-Reductases for the Desymmetrizing Hydrogenation of 2,5-Cyclohexadienones under Environmentally Benign Conditions

Quaternary all-carbon stereocenters are structural motifs frequently occurring in natural products and pharmaceutically active compounds. Despite their importance, the generation of quaternary stereogenic centers still represents a formidable synthetic challenge.^1−21^ While the stereoselective synthesis of such stereocenters at positions in close vicinity to carbonyl functionalities (α,α-difunctionalized^22−28^ and β,β-difunctionalized carbonyl compounds^29,30^) has been relatively well investigated, the formation of quaternary stereocenters in remote positions to an activating functional group is considerably less explored. For synthesizing carbonyl compounds carrying a quaternary stereocenter in γ-position, desymmetrizing 4,4-disubstituted 2,5-cyclohexadienones has been established as an efficient strategy.^21,31−40^ Such desymmetrization strategies most often rely on intramolecular functionalization of one double bond via the Stetter reaction,^41,42^ Michael addition,^43−45^ Diels–Alder,^46^ or [2 + 3]-cycloaddition^47^ reaction motifs. Furthermore, it was shown that intermolecular Michael addition reactions^48,49^ and Diels–Alder reactions^50^ could be used for cyclohexadienone desymmetrization. These desymmetrization reactions rapidly add complexity to the core cyclohexadienone structure by selectively attaching a substituent to one of the two double bonds. However, if, for certain synthetic applications, no additional functionalization at the α- or β-position is desired, a desymmetrizing hydrogenation of a cyclohexadienone would be ideal for establishing the desired quaternary stereocenter within a 2-cyclohexenone structure, allowing synthetic flexibility to access various synthetic destinations (Scheme 1A). In 2016, Nishiyama reported the first example of such a desymmetrizing hydrogenation using a chiral Rh catalyst with a pincer ligand producing 2-cyclohexenones in up to 77% ee.^51^

In an attempt to overcome the dependence on precious Rh metal as part of the catalytic system, Corey reported a Cu-catalyzed desymmetrizing hydrogenation with Ph-BPE as a chiral ligand and DIBAL as a stoichiometric reducing agent (up to 97% ee).^52^ However, superstoichiometric amounts of carcinogenic HMPA were required. These disadvantages could be overcome by Shen and co-workers using the same chiral catalyst but with Et_3_SiH as the stoichiometric reducing agent (up to 96% ee) (Scheme 1B).^53^ Another earth-abundant metal-catalyzed cyclohexadienone desymmetrization was reported by Zhang and co-workers,^54^ in which a Ni/Ph-BPE complex was used for double bond reduction (up to 93% ee) requiring an elevated H_2_ pressure of 50 bar (Scheme 1B).

In contrast to transition-metal catalysis, biocatalysis relies on enzymes as catalysts. Enzymes typically operate under environmentally benign conditions^55^ and can be engineered via well-established methods.^56,57^ Building on our previous work with ene-reductases,^58^ we reasoned that a biocatalytic desymmetrizing hydrogenation of cyclohexadienones could be catalyzed by ene-reductases (Scheme 1C). Ene-reductases from the old yellow enzyme (OYE) family are known to reduce activated double bonds by employing their noncovalently bound flavin cofactor.^59−65^ In contrast to the transition metal-catalyzed hydrogenation reactions described above, in which the stereochemical induction resulted from the coordination of a chiral metal complex to the C=O atom, we were expecting that a biocatalytic desymmetrizing hydrogenation of cyclohexadienones would lead to higher enantioselectivities since this approach would source its chiral induction from the all enclosing nature of an ene-reductase-active site (Scheme 1C). As the stereochemistry-inducing residues are engulfing the substrate in its entirety, we assumed that a potential ene-reductase catalyzed desymmetrizing hydrogenation would be even better suited to generate a quaternary stereocenter in the γ-position—which is spatially quite distant from the cyclohexadienone carbonyl moiety—than it is the case for the aforementioned metal hydride catalyzed approaches.

## Results and Discussion

4-Methyl-4-phenyl-cyclohexa-2,5-dienone (**1a**) was used as a model substrate for the envisioned biocatalytic 2,5-cyclohexadienone desymmetrization process to benchmark its performance in comparison with previously described metal-catalyzed processes. Initial experiments were performed at 25 °C with an enzyme concentration of 5 μM. The employed amount of NADH was held at an equimolar level to avoid overreduction as a potential side reaction. Furthermore, DMSO was used as cosolvent in a concentration of 9% v/v to ensure sufficient solvation of the rather hydrophobic substrate. YqjM wt^66^ and OPR3 wt^67^ were chosen as enzymes as they can be expressed in sufficient quantities and both ene-reductases have been shown to be very stable under various conditions (pH, temperature, solvents), which makes them suitable for preparative biocatalytic processes. We were pleased to see that both tested enzymes delivered the desired enone in high conversion with only minor amounts of fully reduced cyclohexanone being formed. As judged from these initial GC-MS measurements, YqjM appeared to be the more active enzyme in this desymmetrization process. Chiral HPLC showed that cyclohexenone **2a** was formed in >99% ee (Table 1, Entry 1). We were delighted that this very first experiment for a biocatalytic hydrogenative desymmetrization had already shown a higher asymmetric induction than all previously reported transitional-metal-catalyzed processes.

**Table 1:**
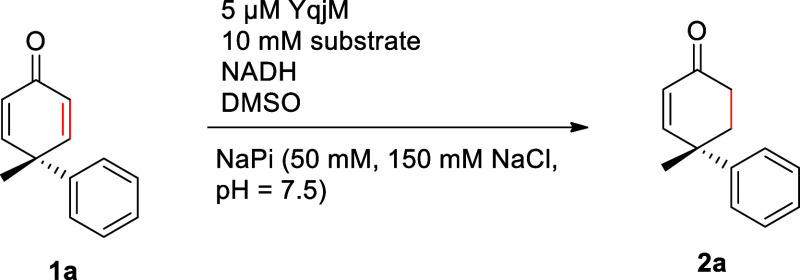
Optimization of Biocatalytic 2,5-Cyclohexadienone Desymmetrization^a^

entry	shaking velocity [rpm]	temperature [°C]	time [h]	c-NADH [mM]	DMSO [% v/v]	Yield—**2a** [%]
1^b^	300	25	1	15	9	78
2	400	25	1	15	9	73
3	500	25	1	15	9	75
4	300	23	1	15	9	72
5	300	27	1	15	9	78
6	300	30	1	15	9	81
7	300	30	18	15	6	76
**8**	**300**	**30**	**18**	**15**	**9**	**80**
9	300	30	18	20	6	73
10	300	30	18	20	9	79

a Reactions were performed in duplicates. Cyclohexenone yields were determined by HPLC at a wavelength of 254 nm. For quantification, external calibrations aided by 1,3,5-tribromobenzene (TBB) as external standard were used.

b **2a** was generated with >99% ee as judged by chiral HPLC (CHIRACEL OJ-H column).

In order to optimize the conditions of the observed biocatalytic desymmetrization process, parameters like shaking velocity, NADH concentration, and temperature were screened (Table 1). First, it was found that the variation of the shaking velocity had almost no influence on the yield of **2a**. Therefore, 300 rpm were used in the following experiments. When varying the temperature, the best results after 1 h were obtained at 30 °C (Entry 6). Increasing the reaction time did not significantly alter the observed 2-cyclohexenone (**2a**) formation (Entries 8–11), indicating a high kinetic preference for the hydrogenation of the first of the two double bonds of the substrate. When the DMSO concentration was reduced from 9 to 6% v/v, a slightly decreased formation of **2a** was observed (Entries 7–10). As an attempt to improve the yield of **2a** by promoting the biotransformation with an increased NADH loading of 2 equiv (Entries 9 and 10) did not lead to distinctive improvements, we continued to use 1.5 equiv of NADH and decided to carry out our biotransformations in 18 h reaction time.

**Scheme 2:**
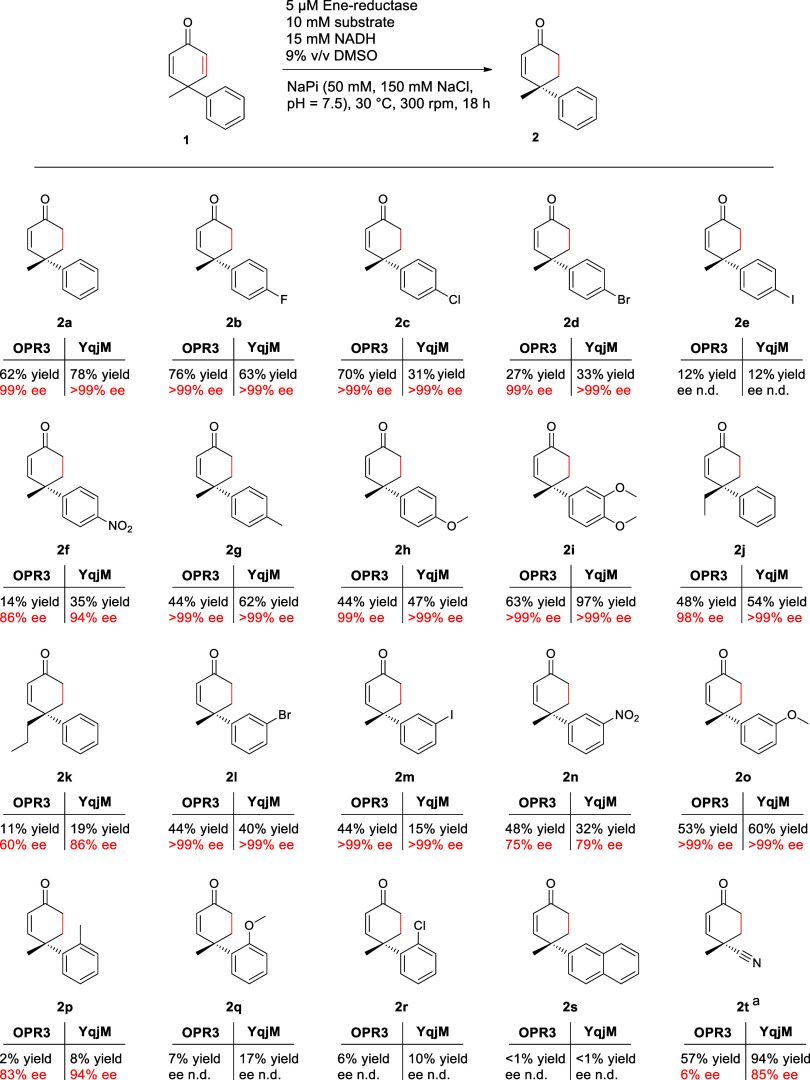
Substrate Scope of the Ene-Reductase Catalyzed Cyclohexadienone Desymmetrization Reactions were performed in triplicates. Cyclohexenone yields were determined by HPLC at a wavelength of 254 nm. For quantification, external calibrations aided by 1,3,5-tribromobenzene (TBB) as an external standard were used. Substrate-recoveries as determined by HPLC-MS are summarized in the Supporting Information (Table S3). Enantioselectivities were determined by chiral HPLC (CHIRACEL OJ-H): (a) For the biotransformation of **1t**, only 1.1 eq NADH were used, and the reaction time was reduced to 3 h. n.d. = not determined.

We investigated the versatility of our desymmetrization process by testing various 4,4-disubstituted 2,5-cyclohexadienones with OPR3 as well as YqjM as the biocatalyst (Scheme 2).

We observed that the obtained 2-cyclohexenone yields were affected by the size of the substituent in the *para*-position of the 4-phenyl substituent (Scheme 2). Nonsubstituted substrate **1a** was desymmetrized by the better-performing YqjM with an >99% ee in 78% yield, translating to a turnover number (TON) of 1560. 4′-Fluoro-4-phenyl-substituted cyclohexenone (**2b**) was obtained in yields of 76 and 63% for OPR3 and YqjM, respectively. These yields are similar to those obtained in the desymmetrization of **1a** and correspond to TONs of 1520 for the OPR3 and 1260 for the YqjM. The productivity began to drop when substrates with larger halogen atoms at this position were transformed. Notably, the 4′-chloro-substituted substrate **1c** showed a remarkable difference depending on the employed enzyme. In this case, OPR3 delivered **2c** in a good yield of 70%, while only a 31% yield was observed with YqjM as the biocatalyst. 4′-Bromo- and 4′-iodo-substituted substrates **1d** and **1e** delivered with the better-performing YqjM enzyme the corresponding 2-cyclohexenones **2d** and **2e** in yields of 33 and 12%, respectively. In these biotransformations, the mass balance was mostly complemented by the respective unreacted dienone substrates (Supporting Information). Interestingly and much to our delight, the enantioselectivity of the produced products was not affected by increasing the size of the *para*-halogenide substituent, as **2b**–**2d** were generated in >99% ee for both tested enzymes. 4′-Nitro-4-phenyl-substituted substrate **1f** was converted by YqjM to **2f** in 35% HPLC-yield but very good enantioselectivity (94% ee). A methyl group in this *para*-aryl position (**1g**) was better accepted, producing the corresponding 2-cyclohexenone **2g** in 62% yield (>99% ee). 4′-Methoxy-substituted substrate **1h** was converted in 44% yield with OPR3 and 47% yield with YqjM with high enantioselectivities for both enzymes (99% for OPR3, >99% ee for YqjM). Interestingly, the sterically more demanding 3′,4′-dimethoxy-4-phenyl substituted substrate **1i** was converted to the corresponding 2-cyclohexenone **2i** in higher yields than **1h** by both investigated ene-reductases (63% for OPR3 and 97% for YqjM) in enantiopure form (>99% ee). This corresponds to a TON of 1940 for the better-performing YqjM and suggests that, in addition to sterics, electronic factors might also influence the substrate-enzyme interactions. The steric influence was also investigated in a series in which we replaced the methyl substituent in standard substrate **1a** with ethyl (**1j**) and propyl (**1k**) groups. While the transformation of ethyl-substituted substrate **1j** with YqjM gave the corresponding enone **2j** in 54% yield (>99% ee), the propyl-substituted substrate **1k** was converted to enone **2k** in only 19% yield (86% ee), exhibiting a strong steric influence. *Meta*-substituted substrates were quite well tolerated. 3′-Bromo-substituted 2,5-cyclohexadienone **1l** delivered the respective enone **2l** in 44% yield (>99% ee) with OPR3 and 40% yield (>99% ee) with YqjM. As already observed with *para*-substituted substrates, an iodo-substituent (**1m**) also led to the lowest yields within the group of *meta*-substituted substrates. In contrast, 3′-nitro- (**1n**) and 3′-methoxy- (**1o**) substituted substrates were converted with good conversions. Limitations in the reaction were found for *ortho-*substituted substrates **1p**-**1r**, which were converted only in low yields of 2–17%, and naphthyl-substituted substrate **1s** was sterically too demanding for the investigated enzymes. In this case, the desired enones could only be found in trace amounts.

In order to test the limits of our reaction, we aimed for the synthesis of 4-cyano-4-methyl-2-cyclohexenone (**2t**), which has been recently used as an intermediate in the divergent total syntheses of several napelline-type C-20 diterpenoid alkaloid natural products and could be accessed via an asymmetric Diels–Alder reaction with 88% ee.^68^ For the envisioned alternative access using our biocatalytic desymmetrization reaction, the required cyclohexadienone **1t** was regarded as a particularly challenging substrate as the methyl and cyano groups are similar in size (Scheme 2). In fact, we found that **1t** is much more susceptible to overreduction than the previously described aryl substituted substrates. Fortunately, we could overcome this problem by reducing the amount of utilized cofactor to 1.1 eq NADH. Furthermore, the reaction time was reduced to 3 h for this substrate. Under these conditions, **1t** was converted to enone **2t** in 57 and 94% yields with OPR3 and YqjM, respectively. With OPR3, almost no stereoinduction was observed (6% ee). However, YqjM delivered nitrile carrying enone **2t** in 85% ee. This highly enantioselective conversion of **1t** highlights the ability of enzymes to induce chiral induction into substrates with only marginally discriminated substituents, allowing control even of stereogenic centers formed in distant positions to C=O groups required for activation of substrates.

**Scheme 3:**
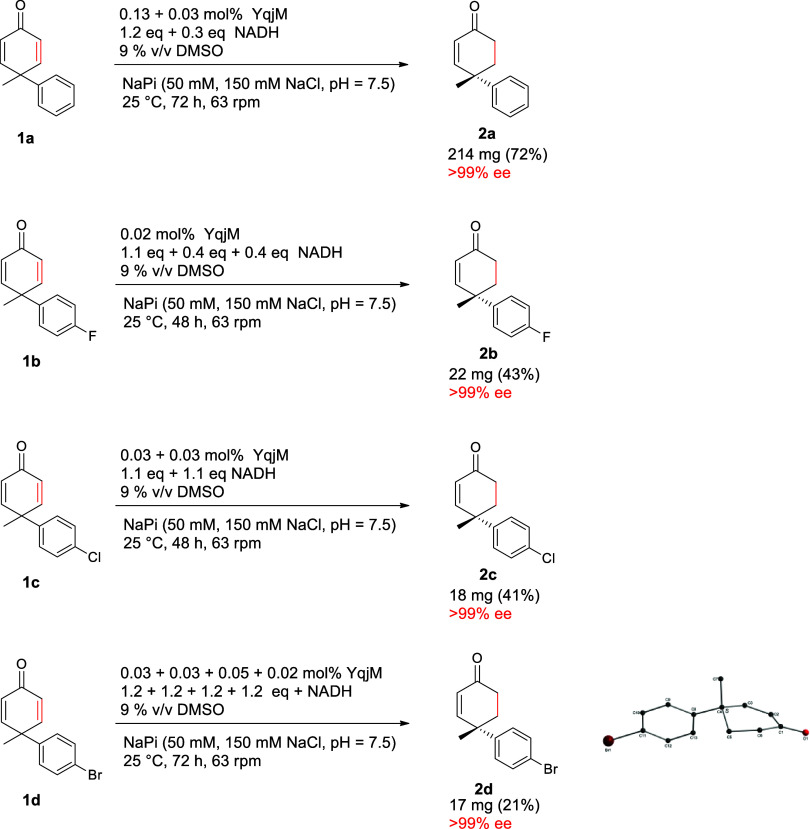
Preparative Scale Ene-Reductase Catalyzed Desymmetrizations

After having established the scope and limitations for our reaction, we wanted to perform our biocatalytic desymmetrization on a preparative scale for several substrates (Scheme 3). For this purpose, the biotransformations were carried out with YqjM as the biocatalyst in Falcon tubes and incubated in an incubation shaker (27 °C, 64 rpm). In the case of substrate **1a**, the biotransformation delivered enone **2a** in 72% yield (>99% ee). The reaction required the addition of additional enzymes as well as cofactors during the transformation to achieve this yield. Thus, at a preparative scale, YqjM desymmetrization of **1a** was achieved with a TON of 450. *Para*-fluorinated and -chlorinated compounds **2b** and **2c** could also be isolated in decent yields of 43% (>99% ee) and 41% (>99% ee), respectively. Brominated substrate **1d**, which is less soluble in the reaction mixture than **1a**–**c**, turned out to be more challenging to scale up. This substrate required additional enzymes and cofactors throughout the biotransformation. **1d** could only be driven to 35% conversion as judged by GC-MS, which resulted in the isolation of 17 mg (21% yield, > 99% ee) of **2d**. The absolute configuration of **2d** could be assigned via X-ray crystallography. The obtained (*S*)-configuration of **2d** is in accordance with the measured optical rotations of the isolated enone, which are in line with the literature data of this product.^53^

**Scheme 4:**
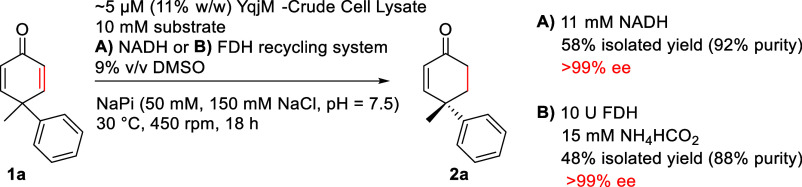
Crude Cell Lysate-Based Biocatalytic Desymmetrization of **1a** with and without a FDH-Cofactor Recycling System

While the use of purified enzymes served us quite well for establishing the scope and limitations of this biocatalytic desymmetrization, larger scale applications would profit from the use of better available enzyme preparations in the form of crude cell lysates. Therefore, we carried out reactions with crude YqjM cell lysate (Scheme 4) (∼5 μM/11% w/w). When this system was used with 11 mM NADH (1.1 equiv), **2a** could be isolated in 58% yield [92% purity with the respective cyclohexanone (from overreduction) making up for the remaining 8%].

Finally, we showed that our YqjM-crude cell lysate-based desymmetrization of **1a** could also be combined with a formate dehydrogenase (FDH)-based cofactor recycling system.^69,70^ For this purpose, we did several screening reactions, which are summarized in the SI (Table S2). Based on this screening, we used 10 U FDH for our 600 μL biotransformations. As terminal reductant 15 mM NH_4_CO_2_H were used. Under these conditions, **2a** could be isolated in 48% yield (88% purity; Scheme 4). Due to NAD(P)H already contained in the used crude cell lysate the desymmetrization using FDH-based cofactor recycling did not require any addition of NADH. Furthermore, we were delighted to see that both crude cell lysate-based systems shown in Scheme 4 produced **2a** in >99% ee. However, close reaction monitoring should be performed to minimize the overreduction product.

**Scheme 5:**
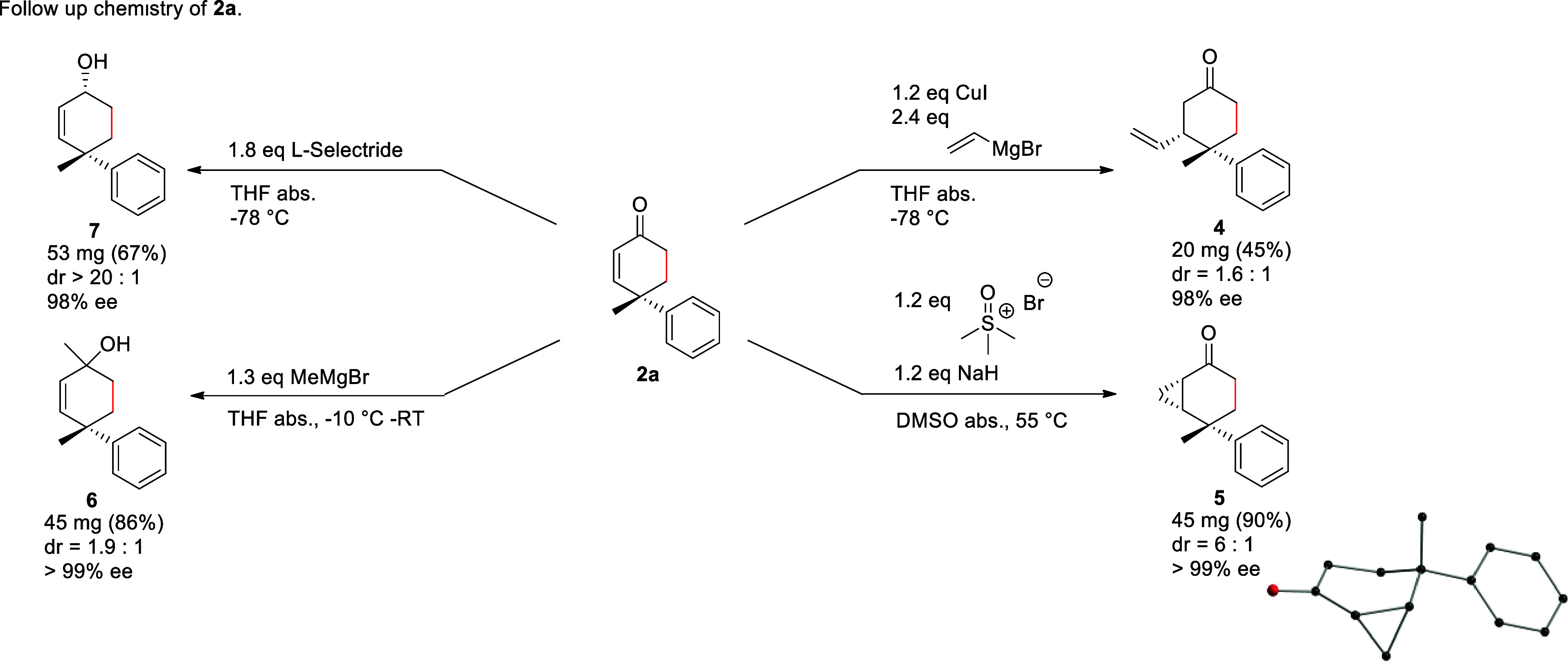
Diversification Reactions with Cyclohexenones **2a**

The synthetic value of these generated chiral cyclohexenones can be leveraged by exploiting the enone moiety for the introduction of a diverse array of structurally different substituents. We used several diversification strategies to increase the complexity of compound **2a** (Scheme 5). The addition of enone **2a** to in situ generated vinyl-cuprate yielded Michael addition product **4** in 45% yield as the 3,4-*cis*-diastereomer (dr >1.6:1). Similarly, a Corey–Chaykovsky reaction generated cyclopropane **5** in 90% yield (dr 6:1). The preference for the *cis*-diastereomer can be explained by an axial attack of the trimethylsulfoxonium ylide reagent at the enone half-chair obtaining a chair-conformation as explained by the Fürst–Plattner rule.^71^ The enantiomeric purity of the quaternary stereocenter could be fully retained. The 1,2-reactivity of enone **2a** was exemplified for further diversification by Grignard addition with MeMgBr producing alcohol **6** in 86% yield (dr 1.9:1). Similarly, **2a** could be reduced with l-selectride to alcohol **7** in 67% yield under nearly full retention of stereoselectivity. Due to the preference of L-selectride to attack in a pseudoequatorial trajectory, **7** was mainly formed as its 1,4-*cis*-diastereomer (dr >20:1).

**Figure 1:**
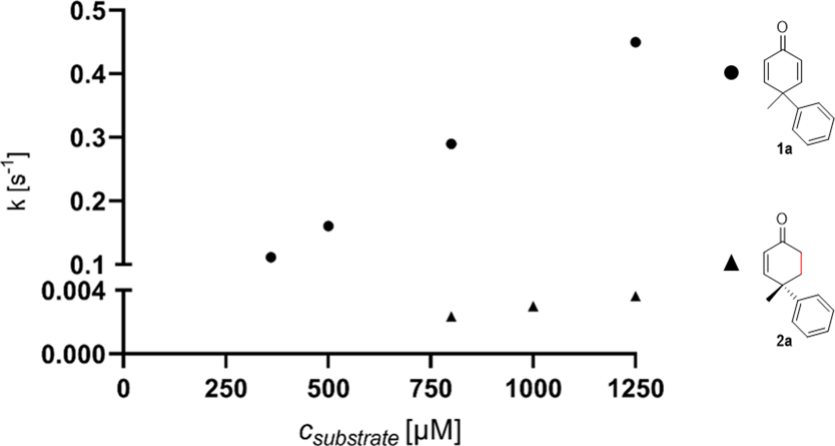
Stopped-flow measurements of dienone **1a** and enone **2a** with YqjM. Measurements were performed against prereduced YqjM under anoxic conditions.

The examples described above highlight the synthetic value and potential of the ene-reductase mediated desymmetric hydrogenation of 4,4-substituted 2,5-cyclohexadienones. In order to gain insight into the observed excellent enantioselectivities of this biocatalytic transformation, we performed stopped-flow measurements for dienone **1a** and its corresponding desymmetrization product **2a**. Both compounds oxidized prereduced YqjM relatively slowly, and no kinetic saturation could be observed in the accessible concentration range (Figure 1). However, dienone **1a** turned out to oxidize the prereduced enzyme more than 120 times faster than enone **2a** [*k*_obs_ (at 800 μM of **1a**): 0.29 s^–1^; *k*_obs_ (at 800 μM of **2a**): 0.0024 s^–1^; Figure 1]. This finding suggests that the binding of **1a**/**2a** to the active site of YqjM favors the reduction of the *pro-S* double bond. Apparently, the active site pocket does not allow for a rotation of the quinone moiety in order to place the *pro-R* double bond near the (reduced) isoalloxazine ring of the flavin cofactor.

**Figure 2:**
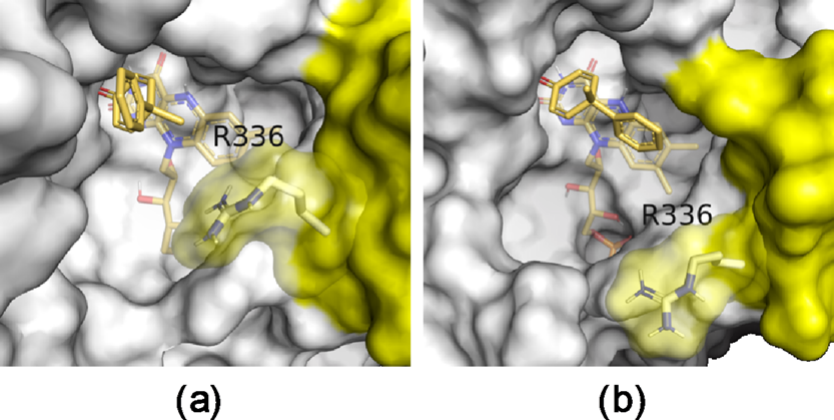
Conformational changes in residue R336 of the second protomer observed during the MD simulations. (a) Docked ligand in the crystal structure of the enzyme shows an “in” conformation of R336 restricting the space in the binding site. (b) MD simulations reveal an “out” conformation of R336, enabling more space in the binding site. The two protomers of the YqjM dimer are shown in gray and yellow nontransparent surface representation. Residue R336 is shown as sticks with carbon atoms colored gray and also has a more transparent surface representation, whereas the substrate and flavin are only shown as sticks with their carbon atoms colored yellow.

In order to rationalize the stereochemical data obtained with the better-performing YqjM, we conducted a computational analysis employing molecular dynamics (MD) simulations and QM/MM calculations. We modeled the structure of the YqjM complex with **1a** using docking and obtained two binding poses of similar energy, in which the carbonyl oxygen atom was hydrogen bonded to the active site histidines H164 and H167 (Figure S7). Binding pose metadynamics was then used to discern the more stable docking pose as starting point for MD simulations. A near-attack conformations (NACs) analysis of the resulting 500 ns MD trajectory revealed a higher preference for the reduction of the *pro-S* double bond than the *pro-R* double bond (Figures S7 and S8). YqjM forms a dimer, with residues from both protein chains lining the active site cavity. In the original crystal structure of YqjM in complex with p-nitrophenol,^72^ the side chain of the arginine residue R336 points into the active site, partially blocking access to the flavin cofactor. During the MD simulations, we observed notable conformational changes in R336, opening up the active site and thereby favoring the *pro-S* binding pose (Figure 2).

**Figure 3:**
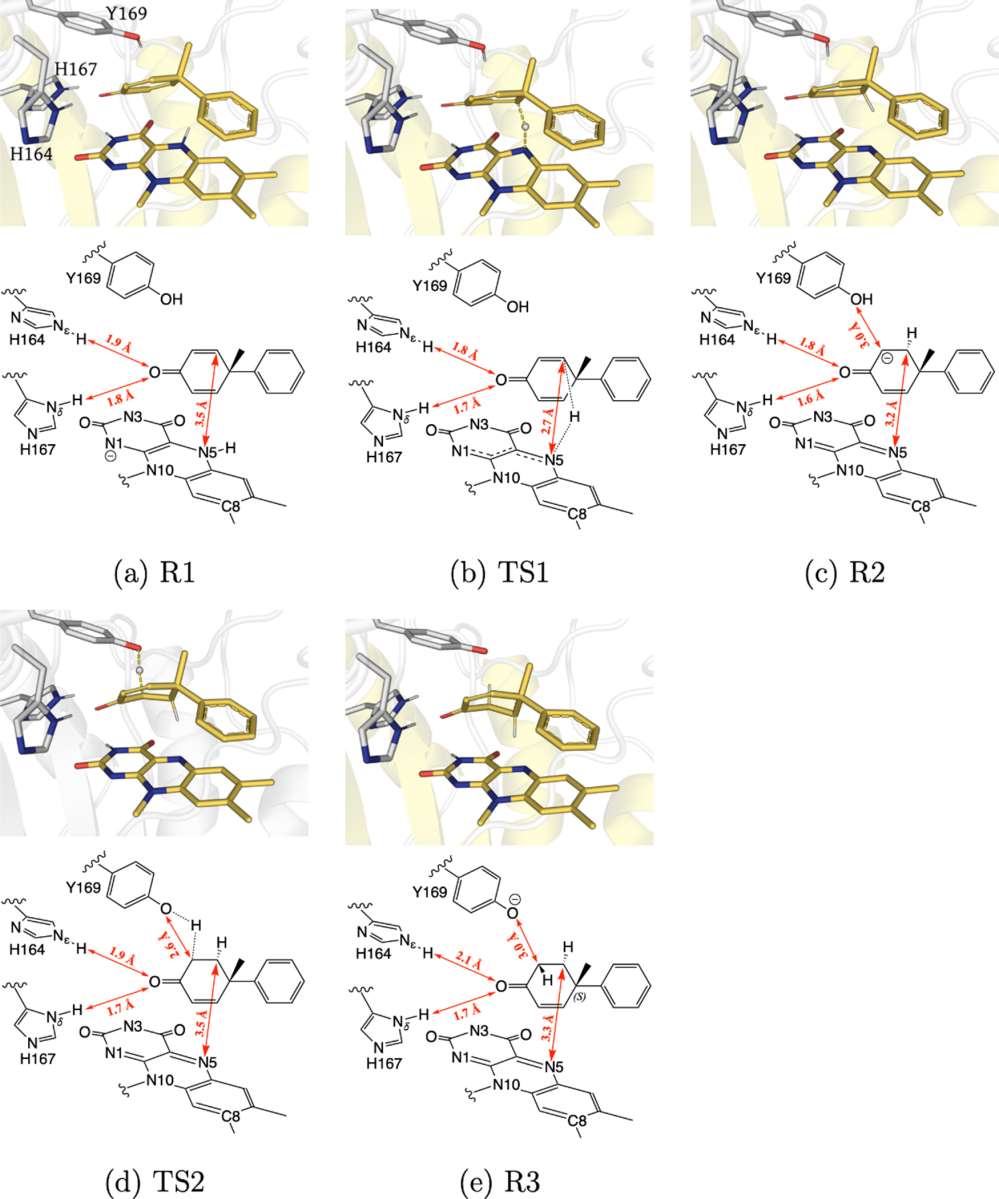
(a–e) (Top) QM/MM geometry optimized molecular structures corresponding to stationary points along the lowest energy reaction coordinate for the reduction of **1a**. TS1 depicts the transition state for the hydride transfer step, and TS2 displays the transition state for the proton transfer step. The substrate and flavin are shown in yellow sticks, and side chains of active site residues are shown in gray sticks. In TS1 and TS2, the transient hydride and proton are also depicted as a gray ball, respectively. (Bottom) Schematic of each configuration showing key distances (red).

Quantum mechanics/molecular mechanics (QM/MM) computations were conducted based on a snapshot of the *pro-*S subpopulation resulting from MD simulations. The QM/MM geometry optimized structures corresponding to stationary points along the lowest energy reaction coordinate for the reduction of the *pro-S* binding pose of **1a** are shown in Figure 3. The QM/MM-optimized binding mode of **1a** is similar to the one observed for typical OYE substrates like α,β-unsaturated ketones. The electron-withdrawing carbonyl group forms hydrogen bonds with two active site histidines (H164 and H167), resulting in the partial polarization of the C=C double bond and its activation for reduction.^73^ Hydride transfer from the reduced flavin to the C-ß of **1a** turned out to be the rate-determining step with a calculated activation energy of 18.1 kcal/mol. The complete energy profile is in accordance with previously reported values^74^ and can be found in Figure S9. In the reactant state (R1) for the hydride transfer step, the distances between the hydride donor atom N5 of the flavin and the *pro-S* C-ß atom and the *pro-R* C-ß atom of **1a** are 3.5 and 5.1 Å, respectively (Figure 3a). Even though the *pro-R* C-ß of **1a** is further apart from the hydride donor N5 atom, this distance difference does not fully explain the excellent enantioselectivity of our biocatalytic desymmetrization. Another decisive factor for the discrimination between both enantiotopic C-ß positions is the (N10–N5–Cß) angle, which is measured at 101° for the *pro-S* C-ß atom and 75° for the *pro-R* C-ß atom. Due to this difference, the hydride can attack the *pro-S* C-ß via a favorable orthogonal trajectory,^75,76^ whereas it has to settle with a smaller angle of attack to reduce the *pro*-*R* C-ß. Another decisive factor for the discrimination between both enantiotopic C-ß positions is the (N10–N5–Cß) angle, which is measured at 101° for the *pro*-*S* C-ß atom and 75° for the *pro-R* C-ß atom. Due to this difference, the hydride can attack the *pro*-*S* C-ß via a favorable orthogonal trajectory, whereas it has to settle with a smaller angle of attack to reduce the *pro-R* C-ß. Similar geometrical constraints were observed in the case of **2a**, where the positioning of the double bond over the flavin is unfavorable for an efficient hydride transfer (Figure S10).

In summary, we have presented the first biocatalytic desymmetrization of 2,5-cyclohexadienones. Due to the strong preference for the *pro-S* orientation of cyclohexadienone substrates in YqjM and OPR3, cyclohexenones carrying quaternary stereocenters could be generated with excellent enantioselectivities (>99% ee), which surpass previously described methods using enantioselective transition-metal catalysis in respect to both enantioselectivity as well as catalytic efficiency (TON). YqjM was even capable of discriminating between sterically similar methyl- and nitrile-substituents with a high degree of stereoinduction (85% ee) for an intermediate, which can function for the formal total synthesis of napelline-type C-20 diterpenoid alkaloids.^68^ This biocatalytic desymmetric hydrogenation using accessible wild type ene-reductases adds a highly stereoselective and environmentally benign biocatalytic method to the synthetic toolbox, delivering quaternary stereocenters.

## Supporting Information Available

Synthetic procedures and corresponding NMR spectra of all synthesized compounds; protocols of the performed analytic and preparative biotransformations; calibrations of educts and products for each biotransformation; summary of observed HPLC recoveries of substrate dienones in biocatalytic screening; chromatograms of chiral HPLC-measurements to support the reported ee; raw data—stopped-flow measurements; structural superposition of the two docking poses of **1a** inside the active site of YqjM; NACs parameters for *pro*-*S* and *pro*-*R* binding poses; NACs analysis of 500 ns MD simulation; overall reaction energies for the reduction of *pro*-*S* double bond of **1a** by YqjM; and structural superimposition of the docked pose of **2a** and the product of the reduction of **1a** obtained from QM/MM computation within the active site of YqjM (PDF)
Crystal data and structure refinement for **2d** (CIF)
Crystal data and structure refinement for **5** (CIF)

## Supplementary Material

cs4c00276_si_001.pdf

cs4c00276_si_002.cif

cs4c00276_si_003.cif

## Abbreviations

DIBAL: diisobutylaluminumhydride
DMSO: dimethyl sulfoxide
FDH: formate dehydrogenase
GC-MS: gas chromatography–mass spectrometry
HMPA: hexamethylphosphoramide
HPLC: high performance liquid chromatography
ee: enantiomeric excess
MD simulation: molecular dynamics simulation
NADH: nicotinamide adeninedinucleotide
OPR3: 12-oxophytodienoate reductase 3 from *Solanum lycopersicum*
YqjM: ene-reductase (old yellow enzyme family) from *Bacillus subtilis*
