# Supplementary material for: Asymmetric Synthesis of Chiral 2-Cyclohexenones with Quaternary Stereocenters via Ene-Reductase Catalyzed Desymmetrization of 2,5-Cyclohexadienones
Source: ACS Catal. 2024 Apr 24;14(9):7256–66. doi: 10.1021/acscatal.4c00276 (PMC11075021; doi:10.1021/acscatal.4c00276)
Supplement: Supplementary file 1 — cs4c00276_si_001.pdf [file cs4c00276_si_001.pdf]

Supporting Information

Asymmetric synthesis of chiral 2-cyclohexenones with quaternary stereocenters via ene-reductase catalyzed desymmetrization of 2,5-cyclohexadienones

Michael Friess,[a] Amit Singh Sahrawat,[b] Bianca Kerschbaumer,[c] Silvia Wallner,[c]

*Ana Torvisco,[d] Roland Fischer,[d] Karl Gruber,[b,e] Peter Macheroux,[c] Rolf Breinbauer[a]**

[a] Institute of Organic Chemistry, Graz University of Technology, Stremayrgasse 9, 8010 Graz, Austria

[b] Institute of Molecular Biosciences, University of Graz, Humboldtstrasse 50, 8010 Graz, Austria

[c] Institute of Biochemistry, Graz University of Technology, Petersgasse 10-12, 8010 Graz, Austria

[d] Institute of Inorganic Chemistry, Graz University of Technology, Stremayrgasse 9, 8010 Graz, Austria

[e] BIOTECHMED Graz, 8010 Graz, Austria

*Corresponding author:
Prof. Rolf Breinbauer: breinbauer@tugraz.at

Table of Contents

1 General information 6

1.1 Chemicals 6

1.1.1 Non-commercial reagents 6

1.1.2 Anhydrous solvents 6

1.2 Analytical methods 8

1.2.1 Thin layer chromatography 8

1.2.2 Flash column chromatography 8

1.2.3 Gas chromatography 8

1.2.4 High performance liquid chromatography 9

1.2.5 Chiral high performance liquid chromatography 9

1.2.6 Nuclear magnetic resonance spectroscopy 9

1.2.7 High resolution mass spectrometry 10

1.2.8 Determination of melting points 10

1.2.9 Determination of optical rotation 11

1.2.10 X-ray Diffraction 11

2 Synthetic procedures 12

2.1 1-Methyl-2,3-dihydro-[1,1'-biphenyl]-4(1*H*)-one (*rac*-**2a**) 12

2.2 1-Methyl-[1,1'-biphenyl]-4(1*H*)-one (**1a**) 13

2.3 General procedure 1 14

2.4 General procedure 2 14

2.5 4'-Fluoro-1-methyl-2,3-dihydro-[1,1'-biphenyl]-4(1*H*)-one (*rac*-**2b**) 15

2.6 4'-Fluoro-1-methyl-[1,1'-biphenyl]-4(1*H*)-one (**1b**) 16

2.7 4'-Chloro-1-methyl-2,3-dihydro-[1,1'-biphenyl]-4(1*H*)-one (*rac*-**2c**) 17

2.8 4'-Chloro-1-methyl-[1,1'-biphenyl]-4(1*H*)-one (**1c**) 18

2.9 4'-Bromo-1-methyl-1,3-dihydro-[1,1'-biphenyl]-4(1*H*)-one (*rac*-**2d**) 18

2.10 4'-Bromo-1-methyl-[1,1'-biphenyl]-4(1*H*)-one (**1d**) 19

2.11 4'-Iodo-1-methyl-2,3-dihydro-[1,1'-biphenyl]-4(1*H*)-one (*rac*-**2e**) 20

2.12 4'-Iodo-1-methyl-[1,1'-biphenyl]-4(1*H*)-one (**1e**) 21

2.13 1-Methyl-4'-nitro-2,3-dihydro-[1,1'-biphenyl]-4(1*H*)-one (*rac*-**2f**) 22

2.14 1-Methyl-4'-nitro-[1,1'-biphenyl]-4(1*H*)-one (**1f**) 23

2.15 1,4'-Dimethyl-2,3-dihydro-[1,1'-biphenyl]-4(1*H*)-one (*rac*-**2g**) 24

2.16 1,4'-Dimethyl-[1,1'-biphenyl]-4(1*H*)-one (**1g**) 25

2.17 4'-Methoxy-1-methyl-2,3-dihydro-[1,1'-biphenyl]-4(1*H*)-one (*rac*-**2h**) 25

2.18 4'-Methoxy-1-methyl-[1,1'-biphenyl]-4(1*H*)-one (**1h**) 26

2.19 (3',4'-Dimethoxy-1-methyl-2,3-dihydro-[1,1'-biphenyl]-4(1*H*)-one (*rac*-**2i**) 27

2.20 3',4'-Dimethoxy-1-methyl-[1,1'-biphenyl]-4(1*H*)-one (**1i**) 28

2.21 1-Ethyl-2,3-dihydro-[1,1'-biphenyl]-4(1*H*)-one (*rac*-**2j**) 29
2.22 1-Ethyl-[1,1'-biphenyl]-4(1*H*)-one (**1j**) 30
2.23 1-Propyl-2,3-dihydro-[1,1'-biphenyl]-4(1*H*)-one (*rac*-**2k**) 30
2.24 1-Propyl-[1,1'-biphenyl]-4(1*H*)-one (**1k**) 31
2.25 3'-Bromo-1-methyl-2,3-dihydro-[1,1'-biphenyl]-4(1*H*)-one (rac-**2l**) 32
2.26 3'-Bromo-1-methyl-[1,1'-biphenyl]-4(1*H*)-one (**1l**) 33
2.27 3'-Iodo-1-methyl-2,3-dihydro-[1,1'-biphenyl]-4(1*H*)-one (*rac*-**2m**) 34
2.28 3'-Iodo-1-methyl-[1,1'-biphenyl]-4(1*H*)-one (**1m**) 35
2.29 1-Methyl-3'-nitro-2,3-dihydro-[1,1'-biphenyl]-4(1*H*)-one (*rac*-**2n**) 36
2.30 1-Methyl-3'-nitro-[1,1'-biphenyl]-4(1*H*)-one (**1n**) 37
2.31 3'-Methoxy-1-methyl-2,3-dihydro-[1,1'-biphenyl]-4(1*H*)-one (*rac*-**2o**) 37
2.32 3'-Methoxy-1-methyl-[1,1'-biphenyl]-4(1*H*)-one (**1o**) 38
2.33 1,2'-Dimethyl-2,3-dihydro-[1,1'-biphenyl]-4(1*H*)-one (*rac*-**2p**) 39
2.34 1,2'-Dimethyl-[1,1'-biphenyl]-4(1*H*)-one (**1p**) 40
2.35 General Procedure 3 41
2.36 2-(2-Methoxyphenyl)propanal (**3q**) 41
2.37 2'-Methoxy-1-methyl-2,3-dihydro-[1,1'-biphenyl]-4(1*H*)-one (*rac*-**2q**) 42
2.38 2'-Methoxy-1-methyl-1[1,1'-biphenyl]-4(1*H*)-one (**1q**) 43
2.39 (2-Chlorophenyl)propanal (**3r**) 44
2.40 2'-Chloro-1-methyl-2,3-dihydro-[1,1'-biphenyl]-4-(1*H*)-one (*rac*-**2r**) 45
2.41 2'-Chloro-1-methyl-[1,1'-biphenyl]-4(1*H*)-one (**1r**) 46
2.42 4-Methyl-4-(naphthalen-2-yl)cyclohex-2-enone (*rac*-**2s**) 46
2.43 4-Methyl-4-(naphthalen-2-yl)cyclohexa-2,5-dienone (**1s**) 47
2.44 1-Methylcyclohexa-2,5-diene-1-carbonitrile (**3t**) 48
2.45 1-Methyl-4-oxocyclohexa-2,5-dienecarbonitrile (**1t**) 49
2.46 1-Methyl-4-oxocyclohex-2-enecarbonitrile (*rac*-**2t**) 50
2.47 (3*S*,4*S*)-4-Methyl-4-phenyl-3-vinylcyclohexanone (**4**) 51
2.48 (1*S*,5*S*,6*R*)-5-Methyl-5-phenylbicyclo[4.1.0]heptan-2-one (**5**) 53
2.49 (1*S*)-1,4-Dimethyl-1,2,3,4-tetrahydro-[1,1'-biphenyl]-4-ol (**6**) 54
2.50 (1*S*,4*R*)-1-Methyl-1,2,3,4-tetrahydro-[1,1'-biphenyl]-4-ol (**7**) 57
3 Biological Section 59
3.1 Expression of OPR3 and YqjM 59
3.2 DNA sequences and translated amino acid sequences of ene-reductases 59
3.3 Preparation of buffers 61
3.4 Preparation of YqjM crude cell lysate 62
3.5 Purification of OPR3 62
3.6 Purification of YqjM 63

3.7 Estimation of enzyme concentrations based on absorption spectra 63
4 Biocatalytic Section 64
4.1 Analytical scale biotransformations 64
4.2 Preparative scale biotransformations 65
4.2.1 (*S*)-1-Methyl-2,3-dihydro-[1,1'-biphenyl]-4(1*H*)-one (**2a**)............................... 65
4.2.2 (*S*)-4'-Fluoro-1-methyl-2,3-dihydro-[1,1'-biphenyl]-4(1*H*)-one (**2b**) 66
4.2.3 (*S*)-4'-Chloro-1-methyl-2,3-dihydro-[1,1'-biphenyl]-4(1*H*)-one (**2c**) 67
4.2.4 (*S*)-4'-Bromo-1-methyl-2,3-dihydro-[1,1'-biphenyl]-4(1*H*)-one (**2d**)............... 68
4.2.5 (*S*)-1-Methyl-2,3-dihydro-[1,1'-biphenyl]-4(1*H*)-one (**2a**) – preparative scale-crude cell lysate with 1.5 eq NADH 69
4.2.6 (*S*)-1-Methyl-2,3-dihydro-[1,1'-biphenyl]-4(1*H*)-one (**2a**) – preparative scale-crude cell lysate with_1.1 eq NADH 71
4.2.7 Optimization – biocatalytic desymmetrization of **1a** with YqjM wt – crude cell lysate plus FDH based cofactor recycling system. 73
4.2.8 (*S*)-1-Methyl-2,3-dihydro-[1,1'-biphenyl]-4(1*H*)-one (**2a**) – preparative scale-crude cell lysate_with FDH cofactor recycling.. 74
4.3 Biocatalytic Screenings 76
4.3.1 Summarized analytical results.. 76
4.3.2 Comparison of analytical methods for reaction monitoring............................. 78
4.3.3 Desymmetrization of **1a** 81
4.3.4 Desymmetrization of **1b** 83
4.3.5 Desymmetrization of **1c** 85
4.3.6 Desymmetrization of **1d** 88
4.3.7 Desymmetrization of **1e** 90
4.3.8 Desymmetrization of **1f** 91
4.3.9 Desymmetrization of **1g** 93
4.3.10 Desymmetrization of **1h** 96
4.3.11 Desymmetrization of **1i** 98
4.3.12 Desymmetrization of **1j** 100
4.3.13 Desymmetrization of **1k** 103
4.3.14 Desymmetrization of **1l** 106
4.3.15 Desymmetrization of **1m** 108
4.3.16 Desymmetrization of **1n** 110
4.3.17 Desymmetrization of **1o** 112
4.3.18 Desymmetrization of **1p** 115
4.3.19 Desymmetrization of **1q** 117
4.3.20 Desymmetrization of **1r** 118
4.3.21 Desymmetrization of **1s** 119
4.3.22 Desymmetrization of **1t** 120

5 X-ray Structures 123
5.1 X-ray Structure of **2d** 123
5.2 X-ray Structure of **5** 125
6 Determination of pre-steady-state kinetics: stopped flow measurements 127
7 Theoretical studies 129
7.1 Protein structure preparation and docking 129
7.2 Binding Pose Metadynamics (BPMD) 130
7.3 MD Simulations 130
7.4 Computation of Near Attack Conformations (NACs) 130
7.5 QM/MM Computations 133
8 References 136
9 NMR-spectra 139

1 General information

If reactions were performed under inert conditions, e.g. exclusion of water, oxygen or both, all experiments were carried out using established Schlenk techniques. Solvents were dried with common methods and afterwards stored under inert gas atmosphere (argon) over molecular sieves. In some cases, when explicitly mentioned, dry solvents were received from the mentioned suppliers. All reagents were added in a counterstream of inert gas to keep the inert atmosphere. All reactions were stirred with Teflon-coated magnetic stirring bars.

Molecular sieves (3Å or 4Å) were activated in a round-bottom flask with a gas inlet adapter by heating them carefully in a heating mantle at level 1 for at least 24 h under high vacuum until complete dryness was obtained. These activated molecular sieves were stored at RT under argon atmosphere.

Temperatures were measured externally if not otherwise stated. When working at a temperature of 0 °C, an ice-water bath served as the cooling medium. Lower temperatures were achieved by using an acetone/dry ice cooling bath. Reactions, which were carried out at higher temperatures than RT, were heated in a silicon oil bath on a heating plate (RCT basic IKAMAG® safety control, 0-1500 rpm) equipped with an external temperature controller.

1.1 Chemicals

All commercially available chemicals and solvents were purchased from abcr, Acros Organics, Alfa Aesar, Fisher, Fluka, Honeywell, Merck, Roth, Sigma Aldrich, TCI, VWR and used without further purification, unless otherwise stated.

1.1.1 Non-commercial reagents

<u>Et_3N (anhydrous):</u> Et_3N was dried over Na. It was distilled into an amber 1 L Schlenk bottle and stored over activated 4Å molecular sieves under argon atmosphere.

1.1.2 Anhydrous solvents

<u>Acetonitrile:</u> Anhydrous acetonitrile was purchased from Alfa Aesar in >99.8% purity. It was transferred into an amber 1 L Schlenk bottle and stored over activated 3Å molecular sieves under argon atmosphere.

Dichloromethane: Dichloromethane (stabilized with EtOH) was purchased from Fisher, dried over phosphorus pentoxide, distilled and heated under reflux over CaH_2 for 24 h. It was distilled into an amber 1 L Schlenk bottle and stored over 4Å molecular sieves under argon atmosphere.

Diethylether: Diethylether was purchased from VWR, dried through an aluminium oxide column under inert conditions and heated under reflux over CaH_2 for 24 h. It was distilled into an amber 1 L Schlenk bottle and stored over 4Å molecular sieves under argon atmosphere.

N,N-Dimethylformamide: Anhydrous *N,N*-dimethylformamide was purchased from Sigma Aldrich in 99.8% purity. It was transferred into an amber 1 L Schlenk bottle and stored over activated 4Å molecular sieves under argon atmosphere.

1,4-Dioxane: Anhydrous 1,4-dioxane was purchased from Sigma Aldrich in 99.8% purity. It was transferred into an amber 1 L Schlenk bottle and stored over activated 4Å molecular sieves under argon atmosphere.

Methanol: Methanol was purchased from Fisher and heated under reflux over Mg and I_2 for 24 h. It was distilled into an amber 1 L Schlenk bottle and stored over activated 3Å molecular sieves under argon atmosphere.

n-Pentane: Anhydrous *n*-pentane was purchased from Alfa Aesar in >99.8% purity. It was transferred into an amber 1 L Schlenk bottle and stored over activated 4Å molecular sieves under argon atmosphere.

2-Propanol: Anhydrous 2-propanol was purchased from Sigma Aldrich in 99.5% purity. It was transferred into an amber 1 L Schlenk bottle and stored over activated 3Å molecular sieves under argon atmosphere.

Tetrahydrofuran: Tetrahydrofuran was purchased from VWR and heated under reflux over Na until benzophenone indicated dryness (intense blue color). It was distilled into an amber 1 L Schlenk bottle and stored over 4Å molecular sieves under argon atmosphere.

Toluene: Toluene was purchased from Fisher and dried through an aluminium oxide column under inert conditions. It was filled into an amber 1 L Schlenk bottle and stored over activated 4Å molecular sieves under argon atmosphere.

1.2 Analytical methods

1.2.1 Thin layer chromatography

Analytical thin layer chromatography (TLC) was carried out on Merck TLC silica gel aluminum sheets (silica gel 60, F254, 20 x 20 cm). All separated compounds were visualized by UV light (λ = 254 nm and/or λ = 366 nm) and by the listed staining reagents followed by development in heat.

$KMnO_4$: 3.0 g $KMnO_4$ and 20 g K_2CO_3 were dissolved in 300 mL H_2O and afterwards 5.0 mL 5% aq. NaOH were added.

CAM: 50 g $(NH_4)_6Mo_7O_{24}$, 2.0 g $Ce(SO_4)_2$ and 50 mL conc. H_2SO_4 were dissolved in 400 mL water.

1.2.2 Flash column chromatography

Flash column chromatography was performed on silica gel 60 from Acros Organics with particle sizes between 35 µm and 70 µm. Depending on the problem of separation, a 30 to 100-fold excess of silica gel was used with respect to the dry amount of crude material. The dimension of the column was adjusted to the required amount of silica gel and formed a pad between 10 cm and 30 cm. In general, the silica gel was mixed with the eluent and the column was equilibrated. Subsequently, the crude material was dissolved in the eluent and loaded onto the top of the silica gel and the mobile phase was forced through the column using a rubber bulb pump. The volume of each collected fraction was adjusted between 20% and 40% of the silica gel volume.

1.2.3 Gas chromatography

GC-MS analyses were performed on an Agilent Technologies 7890A GC system equipped with a 5975C mass selective detector (inert MSD with Triple Axis Detector system) by electron-impact ionization (EI) with a potential of E = 70 eV. Herein, the samples were separated depending on their boiling point and polarity. The desired crude materials or pure compounds were dissolved, and the solutions were injected by employing the autosampler 7683B in a split mode 1/20 (inlet temperature: 280 °C; injection volume: 0.1 µL). Separations were carried out on an Agilent Technologies J&W GC HP-5MS capillary column ((5%-phenyl)methylpolysiloxane, 30 m x 0.2 mm x 0.25 µm) with a constant helium flow rate (He 5.0 (Air Liquide), 1.085 mL·min^{-1}, average velocity: 41.6 cm·s^{-1}). A general gradient temperature method was used:

MT_50S: initial temperature: 50 °C for 1 min; linear increase to 300 °C (40 °C·min^{-1}); hold for 5 min; 1 min post-run at 300 °C; detecting range: 50.0-550.0 amu; solvent delay: 2.60 min.

1.2.4 High performance liquid chromatography

Analytical HPLC-MS measurements were performed on an Agilent Technologies 1200 Series system (G1379 Degasser, G1312 Binary Pump, G1367C HiP ALS SL Autosampler, G1330B FC/ALS Thermostat, G1316B TCC SL column compartment, G1365C MWD SL multiple wavelength detector (deuterium lamp, 190-400 nm)) equipped with a single quadrupole LCMS detector “6120 LC/MS” using electrospray ionization source (ESI in positive and negative mode). Separations were carried out on a reversed phase Agilent Poroshell 120 SB-C18 (100 x 3.0 mm, 2.7 µm) column equipped with a Merck LiChroCART® 4-4 pre-column, or a reversed phase Agilent Poroshell 120 EC-C18 (100 x 3.0 mm, 2.7 µm) column equipped with a Merck LiChroCART® 4-4 pre-column. The following method was used:
FAST_POROSHELL 120_005_TFA_2_100: 0-0.5 min: 98% H_2O (0.05% TFA) and 2% ACN, 0.5-10 min: linear gradient to 100% ACN, 10-12.5 min: 100% ACN; 0.7 mL/min; 40 °C.

1.2.5 Chiral high performance liquid chromatography

Chiral HPLC measurements were performed on a Hewlett Packard 1100 Series system (G1322A Degasser, G1311A Quat Pump, G1313A HiP ALS SL Autosampler, G1318A TCC SL column compartment, G1315A MWD SL multiple wavelength detector (deuterium lamp, 190-400 nm)). Separations were carried out on a CHIRACEL OJ-H column. Utilized separation methods are stated for each separation problem.

1.2.6 Nuclear magnetic resonance spectroscopy

NMR spectra were recorded on a Bruker Avance III 300 spectrometer (^{1}H: 300.36 MHz; ^{13}C: 75.53 MHz) with autosampler, a Varian Unity Inova 500 spectrometer (^{1}H: 499.87 MHz; ^{13}C: 125.69 MHz, ^{19}F: 470.53 MHz, ^{31}P: 202.35 MHz).

Chemical shifts δ are referenced to the residual proton and carbon signal of the deuterated solvent ($CDCl_3$: δ = 7.26 ppm (^{1}H), 77.16 ppm (^{13}C); CD_3OD: δ = 3.31 ppm (^{1}H), 49.00 ppm (^{13}C); DMSO-d_6: δ = 2.50 ppm (^{1}H), 39.52 ppm (^{13}C); D_2O: δ = 4.79 ppm (^{1}H); CD_3CN: δ = 1.94 ppm (^{1}H), 118.26 ppm / 1.32 ppm (^{13}C); CD_2Cl_2: δ = 5.32 ppm (^{1}H), 54.00 ppm (^{13}C); C_6D_6: δ = 7.16 ppm (^{1}H), 128.06 ppm (^{13}C)).[1] Chemical shifts δ are given in ppm (parts per million) and coupling constants *J* in Hz (Hertz). If necessary, 1D spectra (APT and NOESY) as well as 2D spectra (H,H-COSY, HSQC, HMBC) were recorded for the identification and confirmation of the structure. Signal multiplicities are abbreviated as s (singlet), bs (broad singlet), d (doublet), t (triplet), q (quartet), quint (quintet), m (multiplet), dd (doublet of

doublets), td (triplet of doublets), dt (doublet of triplets), and qd (quartet of doublets). Deuterated solvents for nuclear resonance spectroscopy were purchased from euriso-top®.

1.2.7 High resolution mass spectrometry

High-resolution mass spectra were recorded using the following mass spectrometers: direct inlet (DI) EI spectra were recorded on a Waters Micromass GCT Premier system with an electron ionization (DI-EI) source (70 eV). MALDI spectra were recorded on a Micromass® MALDI micro MX™ spectrometer. Dithranol (1,8-dihydroxy-9,10-dihydroanthracen-9-one) or α-cyano-4-hydroxycinnamic acid served as matrix and PEG as internal standard. HR-ESI-MS measurements were performed using the following procedure: sample spectra were acquired by data-dependent high-resolution tandem mass spectrometry on a QExactive Focus (Thermo Fisher Scientific, Germany). The electrospray ionization potential was set to +3.5 or -3.0 kV, the sheath gas flow was set to 20 L/min, and an auxiliary gas flow of 5 L/min was used. Samples were diluted with an appropriate solvent (methanol or chloroform) and 1 µL was injected on a SeQuant® ZIC®-pHILIC HPLC column (Merck, 100 × 2.1 mm; 5 µm; 100Å; peek coated; equipped with a guard column) The separation solvent (A: MeCN, B: 25 mM NH_4HCO_3; RP: A: 0.1% HCOOH, B: 0.1% HCOOH in CH_3CN) was delivered through an Ultimate 3000 HPLC system (Thermo Fisher Scientific, Germany) with a flow rate of 100 µL/min and appropriate gradients were used for proper sample elution.
Acetonitrile (ACN) HiPerSolv CHROMANORM® for HPLC-Supergradient was obtained from VWR Chemicals, methanol Optima® LC/MS Grade from Fisher Chemicals, formic acid Suprapur® and ammonium hydrogen carbonate for LC-MS LiChropur were purchased from Merck, chloroform Plus for HPLC from Sigma-Aldrich, and H_2O was obtained from a Milli-Q® Advantage A10 water purification system (Merck).

1.2.8 Determination of melting points

Melting points were determined on a Mel-Temp® melting point apparatus from Electrothermal with an integrated microscopical support. They were measured in open capillary tubes with a mercury-in-glass thermometer and were not corrected.

1.2.9 Determination of optical rotation

Optical rotation measurements were performed on a Schmidt+Haensch VariPol polarimeter. All samples were measured at the D-line of the sodium light (λ = 589 nm) in a 5 cm cell. Concentrations are given in g/100 mL.

1.2.10 X-ray Diffraction

Instrument 1 (used for crystal structure determination of **2d**):

For single crystal X-ray diffractometry all suitable crystals were covered with a layer of silicone oil. A single crystal was selected, mounted on a glass rod on a copper pin, and placed in the cold N_2 stream provided by an Oxford Cryosystems cryometer (T = 100 K), if not otherwise stated. XRD data collection was performed on a Bruker APEX II diffractometer with use of Mo Kα radiation (λ = 0.71073 Å) from an IµS microsource and a CCD area detector. Empirical absorption corrections were applied using SADABS.[2] The structures were solved with use of either direct methods or the Patterson option in SHELXS. Structure refinement was carried out using SHELXL.[3] CIF files were edited, validated and formatted with the program OLEX2.[4] The space group assignments and structural solutions were evaluated using PLATON.[5] All non-hydrogen atoms were refined anisotropically. All hydrogen atoms were placed in calculated positions corresponding to standard bond lengths and angles using riding models.

Instrument 2 (used for crystal structure determination of **5**):
Data were collected using an XtaLAB Synergy, Dualflex, HyPix-Arc 100 diffractometer operating at T = 100.0(6) K. Data were measured using ω scans of 0.5° per frame for 4.5 s using Cu Kα radiation. The diffraction pattern was indexed and the total number of runs and images was based on the strategy calculation from the program CrysAlisPro (Rigaku, V1.171.43.92a, 2023) The maximum resolution that was achieved was Θ = 80.800° (0.78 Å). The diffraction pattern was indexed and the total number of runs and images was based on the strategy calculation from the program CrysAlisPro (Rigaku, V1.171.43.92a, 2023) and the unit cell was refined using CrysAlisPro (Rigaku, V1.171.43.92a, 2023) on 78695 reflections, 80% of the observed reflections. Data reduction, scaling and absorption corrections were performed using CrysAlisPro (Rigaku, V1.171.43.92a, 2023). The final completeness is 100.00 % out to 80.800° in Θ. A multi-scan absorption correction was performed using CrysAlisPro 1.171.43.92a (Rigaku Oxford Diffraction, 2023) using spherical harmonics, implemented in SCALE3 ABSPACK scaling algorithm. The absorption coefficient µ of this material is 0.567 mm-1 at

this wavelength (λ = 1.542Å) and the minimum and maximum transmissions are 0.411 and 1.000. The structure was solved and the space group $P2_12_12_1$ (# 19) determined by the XT structure solution program[6] using Intrinsic Phasing and refined by Least Squares using version 2018/3 of XL.[7] All non-hydrogen atoms were refined anisotropically. Hydrogen atom positions were calculated geometrically and refined using the riding model. Hydrogen atom positions were calculated geometrically and refined using the riding model.

2 Synthetic procedures

2.1 1-Methyl-2,3-dihydro-[1,1'-biphenyl]-4(1*H*)-one (*rac*-2a)

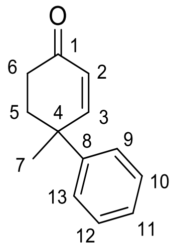

In a 250 mL round-bottom flask a solution of 1.26 mL (1.05 g, 15 mmol, 1 eq) methylvinylketone and 1.97 g (14.7 mmol, 0.98 eq) 2-phenylpropionaldehyde in 54 mL TBME was prepared. The solution was cooled in an ice/water bath. Then, a solution of 0.36 g KOH in 3.9 mL EtOH was added dropwise. The resulting solution was allowed to warm to RT and was stirred overnight at this temperature. Full conversion was indicated by GC-MS. The reaction was quenched by the addition of 1M HCl (15 mL). After phase separation the organic layer was washed with water (15 mL) and brine (15 mL). Then the organic layers were dried over Na_2SO_4 and filtered through a pad of celite. Removal of the solvent under reduced pressure yielded 3.5 g crude product, which was purified via column chromatography [700 mL silica (8x16 cm), CH/EtOAc 25/1 - CH/EtOAc 15/1, fraction size: 170 mL].

$C_{13}H_{14}O$ [186.25 g/mol]; (CAS: 17429-36-6)

Yield 563 mg (3 mmol, 20%); yellow oil

GC-MS 6.07 min [186 (50%), 171 (22%), 144 (80%), 129 (100%)]

^{1}H-NMR (300.36 MHz, $CDCl_3$) δ = 7.21 (m, 5H, H-9 – H-13), 6.84 (d, $^3J_{HH}$ = 10.2 Hz, 1H, H-3), 6.03 (d, $^3J_{HH}$ = 10.2 Hz, 1H, H-2), 2.38-2.00 (m, 4H, H-5, H-6), 1.47 (s, 3H, H-7) ppm.

^{13}**C-NMR** (75.53 MHz, $CDCl_3$) δ = 199.6 (C-1), 157.3 (C-3), 145.4 (C-8), 128.8 (C-10, C-12), 128.7 (C-2), 126.9 (C-11), 126.3 (C-9, C-13), 40.7 (C-4), 38.2 (C-5), 34.8 (C-6), 27.7 (C-7) ppm.

The recorded spectra are in accordance with those reported in the literature.[8]

2.2 1-Methyl-[1,1'-biphenyl]-4(1*H*)-one (1a)

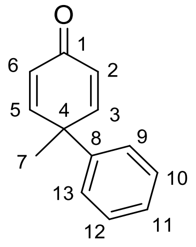

In a flame dried 10 mL Schlenk flask a solution of 150 mg (0.8 mmol, 1 eq) **2a** in 2 mL 1,4-dioxane abs. was prepared. 236 mg (1.04 mmol, 1.3 eq) DDQ were added and the resulting orange solution was stirred at RT overnight. Full conversion was indicated by GC-MS. For workup the reaction mixture was filtered through a pad of celite, which was then rinsed with cyclohexane. The filtrate was washed with 1M NaOH (20 mL) and H_2O (20 mL). The organic layer was dried over Na_2SO_4 and the solvent was removed under reduced pressure. The resulting crude product was purified via column chromatography [50 mL silica, CH/EtOAc 10/1, fraction size: 10 mL].

$C_{13}H_{12}O$ [184.24 g/mol]; (CAS: 28937-18-0)

Yield 69 mg (0.38 mmol, 47%); colourless solid

mp 51-53 °C

R_f 0.22 (CH/EtOAc 9/1, $KMnO_4$)

GC-MS 6.14 min [184 (70%), 156 (100%), 141 (100%), 115 (85%)]

^{1}**H-NMR** (300.36 MHz, $CDCl_3$): δ = 7.43-7.22 (m, 5H, H-9 - H-13), 6.92 (d, $^3J_{HH}$ = 10.0 Hz, 2H, H-3, H-5), 6.28 (d, $^3J_{HH}$ = 10.0 Hz, 2H, H-2, H-6), 1.69 (s, 3H, H-7) ppm.

^{13}**C-NMR** (75.53 MHz, $CDCl_3$,): δ = 186.1 (C-1), 155.5 (C-3, C-5), 140.1 (C-8), 129.1 (C-10, C-12), 127.7 (C-11), 127.2 (C-2, C-6), 126.5 (C-9, C-13), 45.2 (C-3), 24.0 (C-7) ppm.

The recorded spectra are in accordance with those reported in the literature.[8]

2.3 General procedure 1

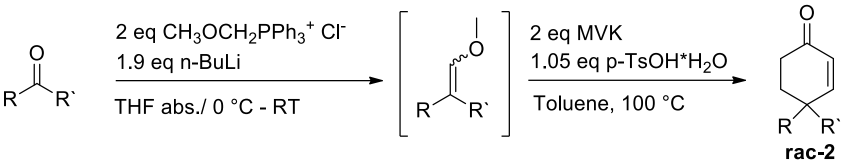

In a flame dried 100 mL Schlenk flask a solution of 6.86 g (20 mmol, 2 eq) methoxymethyltriphenylphosphonium chloride in 45 mL THF abs. was prepared and cooled in an ice/water bath. 7.6 mL (19 mmol, 1.9 eq) n-BuLi (2.5M in THF) were added dropwise. The resulting red solution was stirred at 0 °C for 1 h. Then the corresponding ketone (10 mmol, 1 eq) was added. The reaction mixture was stirred overnight. After GC-MS indicated full conversion, 10 g silica were added and the crude product was adsorbed onto it by removal of the solvent under reduced pressure. Purification of the adsorbed crude product via column chromatography yielded the corresponding vinyl ethers as diastereomeric mixtures. Due to the very apolar nature of most prepared vinylethers, also formed PPh_3 was found in these vinylethers.

For the cyclization step a 0.3M solution of the vinylether in toluene was prepared in an inertized 100 mL Schlenk flask. 1.05 eq p-toluenesulfonic acid monohydrate, as well as 2 eq freshly distilled methyl vinylketone were added to this solution. The resulting reaction mixture was stirred at 100 °C overnight. After full conversion was achieved, the reaction mixture was quenched by the addition of $NaHCO_3$ sat. (4 mL/mmol substrate). The resulting mixture was extracted with EtOAc (3 x 5mL/mmol). The combined organic layers were dried over Na_2SO_4 and filtered through a pad of celite. The resulting crude product was purified via column chromatography furnishing the corresponding cyclohexenone.

2.4 General procedure 2

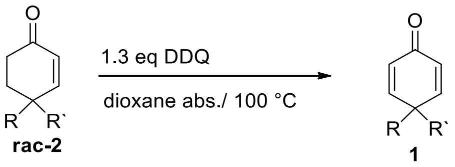

For the desaturation, a 0.4M solution of cyclohexenone in 1,4-dioxane abs. was prepared in an inertized Schlenk flask. After addition of 1.3 eq DDQ the reaction mixture was stirred overnight at 100 °C. The reaction was monitored via GC-MS. For some substrates, addition of another 0.3 eq DDQ and additional reaction time were required to achieve full conversion. For work up, the reaction mixture was cooled to RT, diluted with EtOAc and filtered through a pad of celite. The filtrate was washed with 1M NaOH (25 mL/mmol substrate) and H_2O (25 mL/mmol substrate). The combined organic layers were dried over Na_2SO_4 and again filtered through a pad of celite. After removal of the solvent under reduced pressure, the obtained crude product was purified via column chromatography.

2.5 4'-Fluoro-1-methyl-2,3-dihydro-[1,1'-biphenyl]-4(1*H*)-one (*rac*-2b)

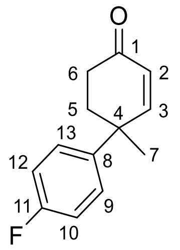

Compound ***rac*-2b** was prepared according to General Procedure 1 with 1.25 g (10 mmol, 1 eq) 4'-fluoroacetophenone as starting material. The vinylether was purified via column chromatography [500 mL silica, CH/EtOAc 70/1 – CH/EtOAc 30/1, fraction size: 100 mL].

Vinylether:

Yield 914 mg (8% w/w PPh_3, 841 mg, 5.1 mmol, 51%); yellow liquid

R_f 0.52 (CH/EtOAc 40/1, $KMnO_4$)

GC-MS 4.88 min 4.70 min [166 (100%), 151 (20%), 123 (93%), 103 (63%)]

Following General Procedure 1, 874 mg (804 mg, 4.8 mmol, 1 eq) vinylether (contains 8% w/w PPh_3) were cyclized. The product was purified via column chromatography [450 mL silica, CH/EtOAc 8/1 – CH/EtOAc 6/1, fraction size: 100 mL].

$C_{13}H_{13}FO$ [204.24 g/mol]; (CAS: 2378808-97-8)

Yield 471 mg (2.3 mmol, 48%)

R_f 0.30 (CH/EtOAc 4/1, $KMnO_4$)

GC-MS 6.06 min [204 (32%), 189 (23%), 162 (59%), 147 (100%)]

^{1}H-NMR (300.36 MHz, $CDCl_3$): δ = 7.35-7.22 (m, 2H, H-9, H-13), 7.10-6.95 (m, 2H, H-10, H-12), 6.90 (d, $^{3}J_{HH}$ = 10.2 Hz, 1H, H-3), 6.12 (d, $^{3}J_{HH}$ = 10.2 Hz, 1H, H-2), 2.48-2.34 (m, 1H, H-6), 2.33-2.23 (m, 1H, H-6), 2.23-2.06 (m, 2H, H-5), 1.55 (s, 3H, H-7) ppm.

^{13}C-NMR (75.53 MHz, $CDCl_3$,): δ = 199.3 (C-1), 161.7 (d, $^{1}J_{CF}$ = 245.82 Hz, C-11), 156.8 (C-3), 141.1 (d, $^{4}J_{CF}$ = 3.2 Hz, C-8), 128.8 (C-2), 127.9 (d, $^{3}J_{CF}$ = 7.9 Hz, C-9, C-13), 115.5 (d, $^{2}J_{CF}$ = 21.2 Hz, C-10, C-12), 40.3 (C-4), 38.3 (C-5), 34.7 (C-6), 27.9 (C-7) ppm.

^{19}F-NMR (376.38 MHz, $CDCl_3$): δ = -116.06 ppm.

The recorded spectra are in accordance with those reported in the literature.[8]

2.6 4'-Fluoro-1-methyl-[1,1'-biphenyl]-4(1*H*)-one (1b)

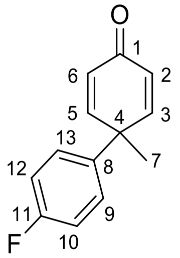

Compound **1b** was prepared according to General Procedure 2 with 435 mg (2.72 mmol, 1 eq) ***rac*-2b** as starting material. The product was purified via column chromatography (100 mL silica, CH/EtOAc 6/1- CH/EtOAc 4/1, fraction size: 20 mL).

**$C_{13}H_{11}FO$**	[202.23 g/mol]; (CAS: 2378808-59-2)
**Yield**	304 mg (1 mmol, 37%); orange solid
**mp**	47 °C
**R_f**	0.23 (CH/EtOAc 4/1, $KMnO_4$)
**GC-MS**	6.12 min [202 (52%), 174 (100%), 159 (100%), 133 (65%), 109 (31%)]

^{1}H-NMR (300.36 MHz, $CDCl_3$): δ = 7.25 (t, $^{3}J_{HH}$ = 6.9 Hz, 2H, H-9, H-13), 7.02 (t, $^{3}J_{HH}$ = 8.5 Hz, 2H, H-10, H-12), 6.87 (d, $^{3}J_{HH}$ = 9.9 Hz, 2H, H-3, H-5), 6.26 (d, $^{3}J_{HH}$ = 9.9 Hz , 2H, H-2, H-6), 1.67 (s, 3H, H-7) ppm.

^{13}C-NMR (75.53 MHz, $CDCl_3$,): δ = 185.8 (C-1), 162.2 (d, $^{1}J_{CF}$ = 247.2 Hz, C-11), 155.2 (C-3, C-5), 135.8 (d, $^{4}J_{CF}$ = 3.18 Hz, C-8), 128.2 (d, $^{3}J_{CF}$ = 8.1 Hz, C-9, C-13), 127.2 (C-2, C-6), 116.0 (d, $^{2}J_{CF}$ = 21.4 Hz, C-10, C-12), 44.6 (C-4), 24.2 (C-7) ppm.

^{19}F-NMR (376.38 MHz, $CDCl_3$): δ = -114.59 ppm.

The recorded spectra are in accordance with those reported in the literature.[8]

2.7 4'-Chloro-1-methyl-2,3-dihydro-[1,1'-biphenyl]-4(1*H*)-one (*rac*-2c)

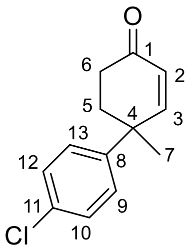

Compound ***rac*-2c** was prepared according to General Procedure 1 with 1.55 g (10 mmol, 1 eq) 4'-chlorocetophenone as starting material. The vinylether was purified via column chromatography [500 mL silica, CH/EtOAc 70/1 – CH/EtOAc 30/1, fraction size: 100 mL].

Vinylether:

Yield 1.66 g (29% w/w PPh_3, 1.18 g, 6.5 mmol, 65%); yellowish liquid

R_f 0.50 (CH/EtOAc 50/1, $KMnO_4$)

GC-MS 5.40 min [182 (100%), 167 (28%), 139 (95%), 108 (100%)]

Following General Procedure 1, 1.62 g (1.15 g, 6.3 mmol, 1 eq) vinylether (contains 29% w/w PPh_3) were cyclized. The product was purified via column chromatography [450 mL silica, CH/EtOAc 8/1 – CH/EtOAc 6/1, fraction size: 100 mL].

$C_{13}H_{13}ClO$ [220.70 g/mol]; (CAS: 75854-92-1)

Yield 990 mg (4.49 mmol, 71%); orange oil

R_f 0.60 (CH/EtOAc 3/1, $KMnO_4$)

GC-MS: 6.14 min [220 (41%), 205 (25%), 178 (75%), 163 (23%), 148 (100%) 148 (68%)]

^{1}H-NMR (300.36 MHz, $CDCl_3$): δ = 7.37-7.19 (m, 4H, H-9, H-10, H-12, H-13), 6.89 (d, $^3J_{HH}$ = 10.2 Hz, 1H, H-3), 6.13 (d, $^3J_{HH}$ = 10.2 Hz, 1H, H-2), 2.47-2.34 (m, 1H, H-6), 2.33-2.23 (m, 1H, H-6), 2.23-2.07 (m, 2H, H-5), 1.54 (s, 3H, H-7) ppm.

^{13}C-NMR (75.53 MHz, $CDCl_3$,): δ = 199.1 (C-1), 156.4 (C-3), 144.0 (C-8), 132.9 (C-11), 129.0 (C-2), 128.9 (C-9, C-13/ C-10, C-12), 127.8 (C-9, C-13/ C-10, C-12), 40.4 (C-4), 38.2 (C-5), 34.6 (C-6), 27.7 (C-7) ppm.

The recorded spectra are in accordance with those reported in the literature.[8]

2.8 4'-Chloro-1-methyl-[1,1'-biphenyl]-4(1*H*)-one (1c)

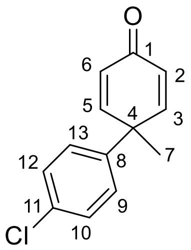

Compound **1c** was synthesized according to General Procedure 2 with 940 mg (4.26 mmol, 1 eq) ***rac*-2c** as starting material. The obtained crude product was purified via column chromatography [250 mL silica, CH/EtOAc 5/1, fraction size: 75 mL].

$C_{13}H_{11}ClO$ [218.68 g/mol]; (CAS: 2243282-31-5)

Yield 431 mg (1.97 mmol, 43%); orange solid

mp 40-44 °C

R_f 0.30 (CH/EtOAc 5/1, $KMnO_4$)

GC-MS 6.73 min [218 (43%), 190 (100%), 175 (52%), 155 (87%)]

^{1}H-NMR (300.36 MHz, $CDCl_3$): δ = 7.31 (d, $^3J_{HH}$ = 8.6 Hz, 2H, H-10, H-12), 7.23 (d, $^3J_{HH}$ = 8.7 Hz, 2H, H-9, H-13), 6.87 (d, $^3J_{HH}$ = 10.0 Hz, 2H, H-3, H-5), 6.28 (d, $^3J_{HH}$ = 10.0 Hz, 2H, H-2, H-6), 1.54 (s, 3H, H-7) ppm.

^{13}C-NMR (75.53 MHz, $CDCl_3$,): δ = 185.7 (C-1), 154.9 (C-3, C-5), 138.7 (C-8), 133.8 (C-11), 129.3 (C-10, C-12), 128.0 (C-9, C-13), 127.4 (C-2), 44.7 (C-4), 24.1 (C-7) ppm.

The recorded spectra are in accordance with those reported in the literature.[8]

2.9 4'-Bromo-1-methyl-1,3-dihydro-[1,1'-biphenyl]-4(1*H*)-one (*rac*-2d)

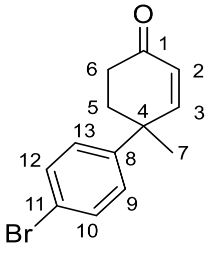

Compound ***rac*-2d** was prepared according to General Procedure 1 with 1.99 g (10 mmol, 1 eq) 4'-bromoacetophenone as starting material. The vinylether was purified via column chromatography [500 mL silica, CH – CH/EtOAc 10/1, fraction size: 100 mL].

Vinylether:

Yield 1.05 g (26% w/w PPh_3, 777 mg, 3.4 mmol, 34%); clourless liquid

R_f 0.45 (CH/EtOAc 50/1, $KMnO_4$)

GC-MS 5.71 min [228, 226 (60%), 132 (100%), 104 (40%)]

Following General Procedure 1, 1.0 g (0.74 g, 3.25 mmol, 1 eq) vinylether (contains 26% w/w PPh_3) was cyclized. The product was purified via column chromatography [400 mL silica, CH/EtOAc 9/1 – CH/EtOAc 5/1, fraction size: 75 mL].

$C_{13}H_{13}BrO$ [265.15 g/mol]; (CAS: 1643566-45-3)

Yield 683 mg (2.57 mmol, 79%); orange oil

R_f 0.38 (CH/EtOAc 4/1, $KMnO_4$)

GC-MS 6.96 min [266/264 (55%), 238/236 (33%), 224/222 (44%), 143 (95%), 128 (100%)]

^{1}H-NMR (300.36 MHz, $CDCl_3$): δ = 7.47 (d, $^3J_{HH}$ = 8.5 Hz, 2H, H-10, H-12), 7.21 (d, $^3J_{HH}$ = 8.5 Hz, 2H, H-9, H-13), 6.88 (d, $^3J_{HH}$ = 10.1 Hz, 1H, H-3), 6.13 (d, $^3J_{HH}$ = 10.2 Hz, 1H, H-2), 2.48-2.34 (m, 1H, H-6), 2.33-2.23 (m, 1H, H-6), 2.23-2.06 (m, 2H, H-5), 1.54 (s, 3H, H-7) ppm.

^{13}C-NMR (75.53 MHz, $CDCl_3$,): δ = 199.1 (C-1), 156.3 (C-3), 144.5 (C-8), 131.8 (C-10, C-12), 129.0 (C-2), 128.1 (C-9, C-13), 120.9 (C-11), 40.5 (C-4), 38.2 (C-5), 34.6 (C-6), 27.7 (C-7) ppm.

The recorded spectra are in accordance with those reported in the literature.[8]

2.10 4’-Bromo-1-methyl-[1,1’-biphenyl]-4(1*H*)-one (1d)

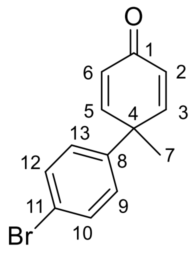

Compound **1d** was prepared according to General Procedure 2 with 638 mg (2.4 mmol, 1 eq) ***rac*-2d** as starting material. The product was purified via column chromatography [50 mL silica, CH/EtOAc 6/1, fraction size: 10 mL].

$C_{13}H_{11}BrO$ [263.13 g/mol]; (CAS: 1643566-29-3)

Yield 342 mg (1.3 mmol, 54%); orange solid

mp 55-56 °C

TLC 0.54 (CH/EtOAc 3/1, $KMnO_4$)

GC-MS 6.94 min [264/262 (43%), 236/234 (100%), 221/219 (32%), 155 (86%), 139 (65%), 128 (50%)]

^{1}H-NMR (300.36 MHz, $CDCl_3$): δ = 7.47 (d, $^3J_{HH}$ = 8.5 Hz, 2H, H-10, H-12), 7.17 (d, $^3J_{HH}$ = 8.5 Hz, 2H, H-9, H-13), 6.86 (d, $^3J_{HH}$ = 10.0 Hz, 2H, H-3, H-5), 6.28 (d, $^3J_{HH}$ = 10.0 Hz , 2H, H-2, H-6), 1.67 (s, 3H, H-7) ppm.

^{13}C-NMR (75.53 MHz, $CDCl_3$,): δ = 185.7 (C-1), 154.7 (C-3, C-5), 132.2 (C-10, C-12), 128.3 (C-9, C-13), 127.4 (C-2, C-6), 121.9 (C-11), 44.7 (C-4), 24.0 (C-7) ppm.

The recorded spectra are in accordance with those reported in the literature.[8]

2.11 4'-Iodo-1-methyl-2,3-dihydro-[1,1'-biphenyl]-4(1*H*)-one (*rac*-2e)

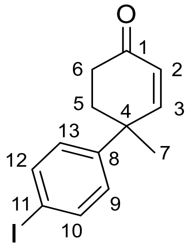

Compound ***rac*-2e** was prepared according to General Procedure 1 with 2.46 g (10 mmol, 1 eq) 4'-iodoacetophenone as starting material. The vinylether was purified via column chromatography [500 mL silica, CH/EtOAc 80/1 – CH/EtOAc 20/1, fraction size: 100 mL].

Vinylether:

Yield 1.48 g (6% w/w PPh_3, 1.39 g, 5.1 mmol, 51%); orange oil

R_f 0.41 (CH/EtOAc 50/1, $KMnO_4$)

GC-MS: 6.09 min [274 (100%), 132 (59%), 104 (15%)]

Following General Procedure 1, 1.48 g (1.39 g, 5.1 mmol, 1 eq) vinylether (contains 6% w/w PPh_3) were cyclized. The product was purified via column chromatography [400 mL silica, CH/EtOAc 12/1 – CH/EtOAc 8/1, fraction size: 80 mL].

$C_{13}H_{13}IO$ [312.15 g/mol]

Yield 655 mg (2.1 mmol, 41%); yellowish solid

mp 62-65 °C

R$_f$ 0.46 (CH/EtOAc 4/1, $KMnO_4$)

GC-MS 7.30 min [312 (100%), 297 (30%), 270 (59%), 143 (48%), 128 (78%)]

HR-MS (ESD) calc m/z for $C_{13}H_{13}IO$ [M+H$^+$]: 313.00893, found: 313.00895

^{1}H-NMR (300.36 MHz, $CDCl_3$): δ = 7.67 (d, $^3J_{HH}$ = 8.5 Hz, 2H, H-10, H-12), 7.08 (d, $^3J_{HH}$ = 8.5 Hz, 2H, H-9, H-13), 6.87 (d, $^3J_{HH}$ = 10.1 Hz, 1H, H-3), 6.12 (d, $^3J_{HH}$ = 10.2 Hz, 1H, H-2), 2.47-2.33 (m, 1H, H-6), 2.33-2.22 (m, 1H, H-6), 2.22-2.05 (m, 2H, H-5), 1.53 (s, 3H, H-7) ppm.

^{13}C-NMR (75.53 MHz, $CDCl_3$,): δ = 199.1 (C-1), 156.3 (C-3), 145.2 (C-8), 137.8 (C-10, C-12), 129.0 (C-2), 128.4 (C-9, C-13), 92.4 (C-11), 40.6 (C-4), 38.1 (C-5), 34.6 (C-6), 27.6 (C-7) ppm.

2.12 4'-Iodo-1-methyl-[1,1'-biphenyl]-4(1*H*)-one (1e)

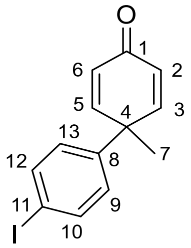

Compound **1e** was prepared according to General Procedure 2 with 610 mg (1.95 mmol, 1 eq) ***rac*-2e** as starting material. The product was purified via column chromatography [150 mL silica, CH/EtOAc 10/1 – CH/EtOAc 1/1, fraction size: 20 mL].

$C_{13}H_{11}IO$ [310.13 g/mol]

Yield 346 mg (1.1 mmol, 57%); yellowish solid

mp 74-75 °C

R$_f$ 0.3 (CH/EtOAc 4/1, $KMnO_4$)

GC-MS 7.35 min [310 (47%), 282 (100%), 267 (26%), 139 (31%)]

HR-MS (ESD) calc m/z for $C_{13}H_{11}IO$ [M+H$^+$]: 310.9933, found: 310.9933

^{1}H-NMR (300.36 MHz, $CDCl_3$): δ = 7.67 (d, $^3J_{HH}$ = 8.4 Hz, 2H, H-10, H-12), 7.04 (d, $^3J_{HH}$ = 8.4 Hz, 2H, H-9, H-13), 6.86 (d, $^3J_{HH}$ = 10.0 Hz, 2H, H-3, H-5), 6.28 (d, $^3J_{HH}$ = 10.0 Hz , 2H, H-2, H-6), 1.66 (s, 3H, H-7) ppm.

^{13}C-NMR (75.53 MHz, $CDCl_3$,): δ = 185.8 (C-1), 154.7 (C-3, C-5), 140.0 (C-8), 138.2 (C-10, C-12), 128.5 (C-9, C-13), 127.5 (C-2, C-6), 93.4 (C-11), 44.8 (C-4), 23.9 (C-7) ppm.

2.13 1-Methyl-4'-nitro-2,3-dihydro-[1,1'-biphenyl]-4(1*H*)-one (*rac*-2f)

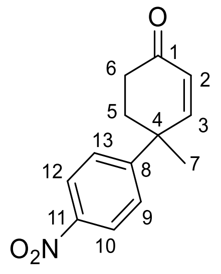

Compound ***rac*-2f** was prepared according to General Procedure 1 with 1.65 g (10 mmol, 1 eq) 4'-nitroacetophenone as starting material. The vinylether was purified via column chromatography [500 mL silica, CH/EtOAc 40/1 – CH/EtOAc 4/1, fraction size: 100 mL].

Vinylether:

Yield 1.0 g (5.2 mmol, 52%); yellow solid

R_f 0.37 (CH/EtOAc 9/1, $KMnO_4$)

GC-MS 6.29 min [193 (100%), 163 (18%), 132 (45%), 103 (39%)]

Following General Procedure 1, 948 mg (4.1 mmol, 1 eq) vinylether were cyclized. The product was purified via column chromatography [500 mL silica, CH/EtOAc 6/1 – CH/EtOAc 2/1, fraction size: 100 mL].

$C_{13}H_{13}NO_3$ [231.25 g/mol]

Yield 824 mg (3.56 mmol, 87%); brownish solid

mp 74-78 °C

R_f 0.38 (CH/EtOAc 2/1, $KMnO_4$)

GC-MS: 7.44 min [231 (32%), 189 (100%), 172 (45%), 142 (69%), 128 (100%)]

HR-MS (ESD) calc m/z for $C_{13}H_{13}NO_3$ [$M+H^+$]: 232.09737, found: 232.09738

^{1}H-NMR (300.36 MHz, $CDCl_3$): δ = 8.21 (d, $^3J_{HH}$ = 8.7 Hz, 2H, H-10, H-12), 7.52 (d, $^3J_{HH}$ = 8.7 Hz, 2H, H-9, H-13), 6.92 (d, $^3J_{HH}$ = 10.2 Hz, 1H, H-3), 6.18 (d, $^3J_{HH}$ = 10.2 Hz, 1H, H-2), 2.53-2.36 (m, 1H, H-6), 2.33-2.15 (m, 3H, H-5, H-6), 1.61 (s, 3H, H-7) ppm.

^{13}C-NMR (75.53 MHz, $CDCl_3$,): δ = 198.4 (C-1), 155.0 (C-3), 153.4 (C-8), 146.9 (C-11), 129.5 (C-2), 127.4 (C-9, C-13), 124.0 (C-10, C-12), 41.1 (C-4), 38.1 (C-5), 34.5 (C-6), 27.6 (C-7) ppm.

2.14 1-Methyl-4'-nitro-[1,1'-biphenyl]-4(1*H*)-one (1f)

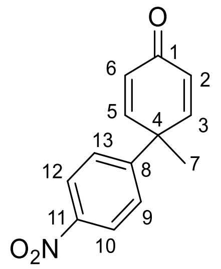

Compound **1f** was prepared according to General Procedure 2 with 771 mg (3.1 mmol, 1 eq) ***rac*-2f** as starting material. The product was purified via column chromatography [100 mL silica, CH/EtOAc 5/1- CH/EtOAc 2/1, fraction size: 20 mL].

$C_{13}H_{11}NO_3$ [229.24 g/mol]

Yield 256 mg (1.12 mmol, 33%); colourless solid

mp 113-115 °C

R_f 0.31 (CH/EtOAc 2/1, $KMnO_4$)

GC-MS 7.46 min [229 (26%), 201 (100%), 153 (37%), 128 (48%)]

HR-MS (ESD) calc m/z for $C_{13}H_{11}NO_3$ [$M+H^+$]: 230.08172, found: 230.08173

^{1}H-NMR (300.36 MHz, $CDCl_3$): δ = 8.20 (d, $^3J_{HH}$ = 8.7 Hz, 2H, H-10, H-12), 7.49 (d, $^3J_{HH}$ = 8.7 Hz, 2H, H-9, H-13), 6.88 (d, $^3J_{HH}$ = 10.0 Hz, 2H, H-3, H-5), 6.35 (d, $^3J_{HH}$ = 9.9 Hz , 2H, H-2, H-6), 1.75 (s, 3H, H-7) ppm.

^{13}C-NMR (75.53 MHz, $CDCl_3$,): δ = 185.2 (C-1), 153.5 (C-3, C-5), 147.7 (C-8), 147.5 (C-11), 128.1 (C-2, C-6), 127.6 (C-9, C-13), 124.3 (C-10, C-12), 45.1 (C-4), 24.2 (C-7) ppm.

2.15 1,4'-Dimethyl-2,3-dihydro-[1,1'-biphenyl]-4(1*H*)-one (*rac*-2g)

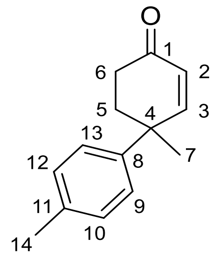

Compound ***rac*-2g** was prepared according to General Procedure 1 with 1.34 g (10 mmol, 1 eq) 4'-methylacetophenone as starting material. The vinylether was purified via column chromatography [500 mL silica, CH/EtOAc 80/1 – CH/EtOAc 20/1, fraction size: 100 mL].

Vinylether:

Yield 1.33 g (14% w/w PPh_3, 1.14 g, 7.1 mmol, 71%); yellow liquid

R_f 0.46 (CH/EtOAc 50/1, $KMnO_4$)

GC-MS 5.07 min [162 (100%), 147 (38%), 119 (74%), 91 (27%)]

Following General Procedure 1, 1.30 g (1.12 g, 6.9 mmol, 1 eq) vinylether (contains 14% w/w PPh_3) were cyclized. The product was purified via column chromatography [500 mL silica, CH/EtOAc 20/1 – CH/EtOAc 10/1, fraction size: 100 mL].

$C_{14}H_{16}O$ [200.28 g/mol]; (CAS: 138371-74-1)

Yield 678 mg (3.39 mmol, 49%); yellow liquid

R_f 0.29 (CH/EtOAc 9/1, $KMnO_4$)

GC-MS 6.36 min [200 (85%), 185 (69%), 158 (65%), 143 (100%), 128 (65%)]

^{1}H-NMR (300.36 MHz, $CDCl_3$): δ = 7.23 (d, $^3J_{HH}$ = 8.2 Hz, 2H, H-9, H-13), 7.16 (d, $^3J_{HH}$ = 8.1 Hz, 2H, H-10, H-12), 6.92 (d, $^3J_{HH}$ = 10.1 Hz, 1H, H-3), 6.11 (d, $^3J_{HH}$ = 10.1 Hz, 1H, H-2), 2.46-2.27 (m, 2H, H-6), 2.34 (s, 3H, H-14), 2.28-2.19 (m, 1H, H-5), 2.19-2.06 (m, 1H, H-5), 1.54 (s, 3H, H-7) ppm.

^{13}C-NMR (75.53 MHz, $CDCl_3$,): δ = 199.7 (C-1), 157.5 (C-3), 142.4 (C-8), 136.5 (C-11), 129.4 (C-10, C-12), 128.6 (C-2), 126.2 (C-9, C-13), 40.4 (C-4), 38.2 (C-5), 34.8 (C-6), 27.8 (C-7), 21.0 (C-14) ppm.

The recorded spectra are in accordance with those reported in the literature.[8]

2.16 1,4’-Dimethyl-[1,1’-biphenyl]-4(1*H*)-one (1g)

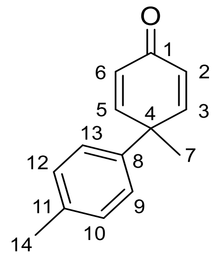

Compound **1g** was prepared according to General Procedure 2 with 670 mg (3.38 mmol, 1 eq) ***rac*-2g** as starting material. The product was purified via column chromatography [200 mL silica, CH/EEtOAc 10/1 – CH/EtOAc 6/1, fraction size: 20 mL].

$C_{14}H_{14}O$ [198.27 g/mol]; (CAS: 1914981-56-8)

Yield 389 mg (1.96 mmol, 58%); orange solid

mp 62-64 °C

R_f 0.38 (CH/EtOAc 4/1, $KMnO_4$)

GC-MS 6.44 min [198 (55%), 170 (76%), 155 (100%), 128 (34%)]

^{1}H-NMR (300.36 MHz, $CDCl_3$): δ = 7.09-6.95 (m, 4H, H-9, H-10, H-12, H-13), 6.76 (d, $^3J_{HH}$ = 9.9 Hz, 2H, H-3, H-5), 6.12 (d, $^3J_{HH}$ = 9.9 Hz, 2H, H-2, H-6), 2.19 (s, 3H, H-14), 1.51 (s, 3H, H-7) ppm.

^{13}C-NMR (75.53 MHz, $CDCl_3$,): δ = 186.1 (C-1), 155.8 (C-3, C-5), 137.5 (C-8), 137.0 (C-11), 129.8 (C-10, C-12), 127.0 (C-2, C-6), 126.4 (C-9, C-13), 44.8 (C-4), 23.9 (C-7), 21.1 (C-14) ppm.

The recorded spectra are in accordance with those reported in the literature.[8]

2.17 4’-Methoxy-1-methyl-2,3-dihydro-[1,1’-biphenyl]-4(1*H*)-one (*rac*-2h)

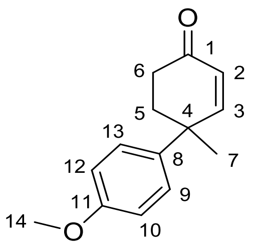

Compound ***rac*-2h** was prepared according to General Procedure 1 with 1.50 g (10 mmol, 1 eq) 4’-methoxyacetophenone as starting material. The vinylether was purified via column chromatography [500 mL silica, CH/EtOAc 100/1 – CH/EtOAc 40/1, fraction size: 100 mL].

Vinylether:

Yield 877 mg (16% w/w PPh_3, 737 mg, 4.1 mmol, 41%); yellow oil

R_f 0.17 (CH/EtOAc 50/1, CAM)

GC-MS 5.58 min [178 (100%), 163 (55%), 135 (85%), 91 (30%)]

Following General Procedure 1, 830 mg (714 mg, 4.0 mmol, 1 eq) vinylether (contains 16% w/w PPh_3) were cyclized. The product was purified via column chromatography [500 mL silica, CH/EtOAc 8/1 – CH/EtOAc 6/1, fraction size: 80 mL].

$C_{14}H_{16}O_2$ [216.28 g/mol]; (CAS: 99432-86-7)

Yield 423 mg (1.96 mmol, 49%); orange oil

R_f 0.29 (CH/EtOAc 9/1, $KMnO_4$)

GC-MS: 6.80 min [216 (71%), 201 (100%), 173 (45%), 145 (32%), 115 (29%)]

^{1}H-NMR (300.36 MHz, $CDCl_3$): δ = 7.26 (d, $^3J_{HH}$ = 8.5 Hz, 2H, H-9, H-13), 6.97-6.80 (m, 3H, H-3, H-10, H-12), 6.11 (d, $^3J_{HH}$ = 10.1 Hz, 1H, H-2), 3.81 (s, 3H, H-14), 2.47-2.28 (m, 2H, H-6), 2.28-2.03 (m, 2H, H-5), 1.54 (s, 3H, H-7) ppm.

^{13}C-NMR (75.53 MHz, $CDCl_3$,): δ = 199.7 (C-1), 158.4 (C-11), 157.5 (C-3), 136.5 (C-11), 137.3 (C-8), 130.0 (C-2), 127.4 (C-9, C-13), 114.1 (C-10, C-12), 55.4 (C-14), 40.1 (C-4), 38.2 (C-5), 34.8 (C-6), 27.9 (C-7) ppm.

The recorded spectra are in accordance with those reported in the literature.[8]

2.18 4'-Methoxy-1-methyl-[1,1'-biphenyl]-4(1*H*)-one (1h)

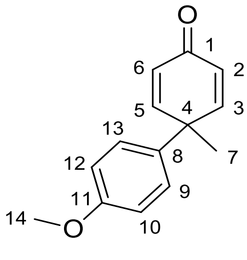

Compound **1h** was prepared according to General Procedure 2 with 360 mg (3.38 mmol, 1 eq) ***rac*-2h** as starting material. The product was purified via column chromatography [50 mL silica, CH/EtOAc 10/1 – CH/EtOAc 8/1, fraction size: 10 mL].

$C_{19}H_{14}O_2$ [214.26 g/mol]; (CAS: 1643566-27-1)

Yield 78 mg (0.36 mmol, 22%); orange solid

mp 49 °C

R_f 0.29 (CH/EtOAc 4/1, CAM)

GC-MS: 6.87 min [214 (68%), 186 (87%), 171 (100%), 128 (71%)]

^{1}H-NMR (300.36 MHz, $CDCl_3$): δ = 7.21 (d, $^3J_{HH}$ = 8.7 Hz, 2H, H-9, H-13), 6.98-6.80 (m, 4H, H-3, H-5, H-10, H-12), 6.25 (d, $^3J_{HH}$ = 9.9 Hz, 2H, H-2, H-6), 3.79 (s, 3H, H-14), 1.66 (s, 3H, H-7) ppm.

^{13}C-NMR (75.53 MHz, $CDCl_3$,): δ = 186.1 (C-1), 159.1 (C-11), 155.9 (C-3, C-5), 131.7 (C-8), 127.6 (C-9, C-13), 126.8 (C-2, C-6), 114.5 (C-10, C-12), 55.4 (C-14), 44.5 (C-4), 24.0 (C-7) ppm.

The recorded spectra are in accordance with those reported in the literature.[8]

2.19 (3',4'-Dimethoxy-1-methyl-2,3-dihydro-[1,1'-biphenyl]-4(1*H*)-one (*rac*-2i)

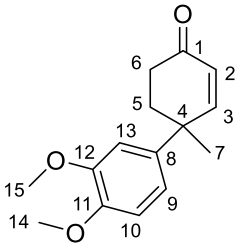

Compound ***rac*-2i** was prepared according to General Procedure 1 with 1.80 g (10 mmol, 1 eq) 1-(3,4-dimethoxyphenyl)ethan-1-one as starting material. The vinylether was purified via column chromatography [500 mL silica, , CH/EtOAc 20/1 – CH/EtOAc 6/1, fraction size: 100 mL].

Vinylether:

Yield 1.53 g (7.34 mmol, 73%); yellow oil

R_f 0.19 (CH/EtOAc 9/1, CAM)

GC-MS 6.13 min [208 (100%), 193 (50%), 165 (37%)]

Following General Procedure 1, 1.47 g (7.04 mmol, 1 eq) vinylether were cyclized. The product was purified via column chromatography [500 mL silica, CH/EtOAc 8/1 – CH/EtOAc 1/1, fraction size: 100 mL].

$C_{15}H_{18}O_3$ [246.3 g/mol]; (CAS: 2378809-04-0)

Yield 622 mg (2.53 mmol, 36%); orange oil

R$_f$ 0.26 (CH/EtOAc 4/1, CAM)

GC-MS 7.19 min [281 (30%), 246 (78%), 231 (78%), 207 (100%)]

^{1}H-NMR (300.36 MHz, $CDCl_3$): δ = 6.91 (d, $^3J_{HH}$ = 10.3 Hz, 1H, H-3), 6.87-6.78 (m, 3H, H-9, H-10, H-13), 6.11 (d, $^3J_{HH}$ = 10.2 Hz, 1H, H-2), 3.87 (s, 6H, H-14, H-15), 2.47-2.29 (m, 2H, H-6), 2.29-2.18 (m, 1H, H-5), 2.18-2.05 (m, 1H, H-5), 1.55 (s, 3H, H-7) ppm.

^{13}C-NMR (75.53 MHz, $CDCl_3$,): δ = 199.7 (C-1), 157.3 (C-3), 149.2 (C-12), 148.1 (C-11), 137.9 (C-8), 128.6 (C-2), 118.6 (C-9), 111.2 (C-10), 109.9 (C-13), 56.1 (C-14/C-15), 56.1 (C-14/C-15), 40.4 (C-4), 38.4 (C-5), 34.8 (C-6), 27.8 (C-7) ppm.

The recorded spectra are in accordance with with those reported in the literature.[8]

2.20 3',4'-Dimethoxy-1-methyl-[1,1'-biphenyl]-4(1*H*)-one (1i)

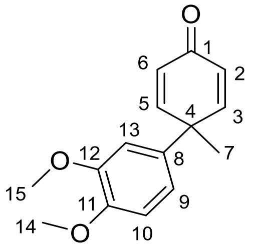

Compound **1i** was prepared according to General Procedure 2 with 596 mg (2.41 mmol, 1 eq) ***rac*-2i** as starting material. The product was purified via column chromatography [100 mL silica, CH/EtOAc 5/1 – CH/EtOAc 1/1, fraction size: 20 mL].

$C_{15}H_{16}O_3$ [244.29 g/mol]; (CAS: 2378808-61-6)

Yield 263 mg (1.08 mmol, 45%); orange solid

mp 65-69 °C

R$_f$ 0.30 (CH/EtOAc 3/1, $KMnO_4$)

GC-MS 7.26 min [244 (100%), 229 (72%), 201 (55%), 115 (52%)]

^{1}H-NMR (300.36 MHz, $CDCl_3$): δ = 6.98-6.80 (m, 4H, H-3, H-5, H-9, H-10), 6.72 (s, 1H, H-13), 6.36 (d, $^3J_{HH}$ = 9.9 Hz, 2H, H-2, H-6), 3.86 (s, 3H, H-14/H-15), 3.83 (s, 3H, H-14/H-15), 1.66 (s, 3H, H-7) ppm.

^{13}C-NMR (75.53 MHz, $CDCl_3$,): δ = 186.1 (C-1), 155.8 (C-3, C-5), 149.3 (C-12), 148.7 (C-11), 132.2 (C-8), 126.9 (C-2, C-6), 118.5 (C-9), 111.5 (C-10), 110.0 (C-13), 56.1 (C-14/C-15), 56.1 (C-14/C-15), 44.8 (C-4), 24.0 (C-7) ppm.

The recorded spectra are in accordance with those reported in the literature.[8]

2.21 1-Ethyl-2,3-dihydro-[1,1'-biphenyl]-4(1*H*)-one (*rac*-2j)

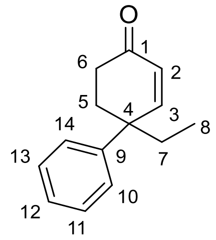

Compound ***rac*-2j** was prepared according to General Procedure 1 with 1.34 g (10 mmol, 1 eq) propiophenone as starting material. The vinylether was purified via column chromatography [500 mL silica, CH/EtOAc 80/1 – CH/EtOAc 40/1, fraction size: 100 mL].

Vinylether:

Yield 1.47 g (23% w/w PPh_3, 1.13 g, 7.0 mmol, 70%); yellow liquid

R_f 0.32 (CH/EtOAc 50/1, $KMnO_4$)

GC-MS 4.85 min [162 (100%), 147 (80%), 115 (47%), 91 (66%)]

Following General Procedure 1, 1.41 g (1.09 g, 6.7 mmol, 1 eq) vinylether (contains 23% w/w PPh_3) were cyclized. The product was purified via column chromatography [400 silica, CH/EtOAc 12/1 – CH/EtOAc 5/1, fraction size: 80 ml].

$C_{14}H_{16}O$ [200.28 g/mol]; (CAS: 1643566-41-9)

Yield 829 mg (4.14 mmol, 62%); orange oil

R_f 0.37 (CH/EtOAc 5/1, CAM)

GC-MS 6.35 min [200 (26%), 171 (100%), 143 (45%), 128 (61%)]

^{1}H-NMR (300.36 MHz, $CDCl_3$): δ = 7.41-7.16 (m, 5H, H-10 – H-14), 7.11 (d, $^3J_{HH}$ = 10.3 Hz, 1H, H-3), 6.17 (d, $^3J_{HH}$ = 10.3 Hz, 1H, H-2), 2.42-2.26 (m, 1H, H-6), 2.26-2.11 (m, 3H, H-5, H-6), 2.01-1.76 (m, 2H, H-7), 0.81 (t, $^3J_{HH}$ = 10.3 Hz, 3H, H-8) ppm.

^{13}C-NMR (75.53 MHz, $CDCl_3$,): δ = 199.9 (C-1), 155.7 (C-3), 143.5 (C-9), 129.7 (C-12), 128.7 (C-11, C-13), 126.9 (C-10, C-14), 126.7 (C-2), 44.4 (C-4), 35.7 (C-5), 34.7 (C-7), 34.4 (C-6), 8.8 (C-8) ppm.

The recorded spectra are in accordance with those reported in the literature.[8]

2.22 1-Ethyl-[1,1'-biphenyl]-4(1*H*)-one (1j)

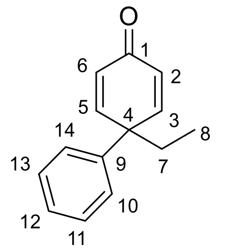

Compound **1j** was prepared according to General Procedure 2 with 763 mg (3.8 mmol, 1 eq) ***rac*-2j** as starting material. The product was purified via column chromatography [100 mL silica, CH/EtOAc 16/1- CH/EtOAc 12/1, fraction size: 20 mL].

$C_{14}H_{14}O$ [198.27 g/mol]; (CAS: 1643566-25-9)

Yield 307 mg (1.55 mmol, 41%), orange solid

mp 44 °C

R_f 0.40 (CH/EtOAc 4/1, CAM)

GC-MS 6.35 min [198 (45%), 169 (95%), 155 (100%), 115 (95%)]

^{1}H-NMR (300.36 MHz, $CDCl_3$): δ = 7.41-7.21 (m, 5H, H-10 – H-14), 6.88 (d, $^3J_{HH}$ = 10.1 Hz, 2H, H-3, H-5), 6.37 (d, $^3J_{HH}$ = 10.1 Hz, 2H, H-2, H-6), 2.17 (q, $^3J_{HH}$ = 7.4 Hz, 2H, H-7), 0.90 (t, $^3J_{HH}$ = 7.4 Hz , 3H, H-8) ppm.

^{13}C-NMR (75.53 MHz, $CDCl_3$,): δ = 186.4 (C-1), 154.3 (C-3, C-5), 140.2 (C-9), 129.1 (C-11, C-13), 129.0 (C-2, C-6), 127.7 (C-12), 126.8 (C-10, C-14), 49.7 (C-4), 30.3 (C-7), 9.1 (C-8) ppm.

The recorded spectra are in accordance with those reported in the literature.[8]

2.23 1-Propyl-2,3-dihydro-[1,1'-biphenyl]-4(1*H*)-one (*rac*-2k)

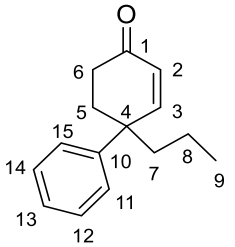

Compound ***rac*-2k** was prepared according to General Procedure 1 with 1.48 g (10 mmol, 1 eq) butyrophenone as starting material. The vinylether was purified via column chromatography [500 mL silica, CH/EtOAc 80/1 – CH/EtOAc 50/1, fraction size: 100 mL].

Vinylether:

Yield 922 g (46% w/w PPh_3, 498 mg, 2.8 mmol, 28%); colourless oil

R_f 0.45 (CH/EtOAc 50/1, $KMnO_4$)

GC-MS: 5.14 min [176 (65%), 147 (100%), 117 (35%), 91 (29%)]

Following General Procedure 1, 872 mg (471 mg, 2.7 mmol, 1 eq) vinylether (contains 46% w/w PPh_3) were cyclized. The product was purified via column chromatography [400 mL, silica, CH/EtOAc 18/1 – CH/EtOAc 10/1, fraction size: 75 mL].

$C_{15}H_{18}O$ [214.31 g/mol]

Yield 339 mg (1.58 mmol, 59%); orange oil

R_f 0.38 (CH/EtOAc 9/1, CAM)

GC-MS 6.57 min [214 (10%), 171 (100%), 143 (42%), 128 (59%)]

HR-MS (ESD) calc m/z for $C_{15}H_{18}O$ $[M+H^+]$: 215.1436, found: 215.1436

^{1}H-NMR (300.36 MHz, $CDCl_3$): δ = 7.42-7.18 (m, 5H, H-11 – H-15), 7.12 (d, $^3J_{HH}$ = 10.2 Hz, 1H, H-3), 6.16 (d, $^3J_{HH}$ = 10.3 Hz, 1H, H-2), 2.44-2.29 (m, 1H, H-6), 2.29-2.11 (m, 3H, H-5, H-6), 1.94-1.70 (m, 2H, H-7), 1.39-1.21 (m, 1H, H-8), 1.21-1.02 (m, 1 H, H-8), 0.88 (t, $^3J_{HH}$ = 7.2 Hz, 3H, H-9) ppm.

^{13}C-NMR (75.53 MHz, $CDCl_3$,): δ = 199.8 (C-1), 156.1 (C-3), 144.0 (C-10), 129.5 (C-2), 128.7 (C-12, C-14), 126.8 (C-11, C-15), 126.7 (C-13), 44.2 (C-4), 44.2 (C-7), 36.1 (C-5), 34.7 (C-6), 17.8 (C-8), 14.7 (C-9) ppm.

2.24 1-Propyl-[1,1'-biphenyl]-4(1*H*)-one (1k)

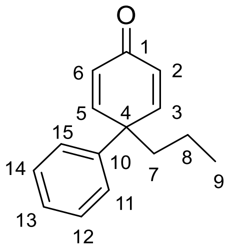

Compound **1k** was prepared according to General Procedure 2 with 284 mg (1.33 mmol, 1eq) ***rac*-2k** as starting material. The product was purified via column chromatography [50 mL silica, CH/EtOAc 20/1 – CH/EtOAc 12/1, fraction size: 10 mL].

$C_{15}H_{16}O$ [212.29 g/mol]

Yield 167 mg (0.78 mmol, 59%); orange oil

R$_f$ 0.32 (CH/EtOAc 9/1, $KMnO_4$)

GC-MS 6.67 min [212 (65%), 170 (100%), 155 (90%), 115 (61%)]

HR-MS (ESD) calc m/z for $C_{15}H_{16}O$ [M+H$^+$]: 213.12794, found: 213.12795

^{1}H-NMR (300.36 MHz, $CDCl_3$): δ = 7.41-7.20 (m, 5H, H-11 – H-15), 6.91 (d, $^3J_{HH}$ = 10.0 Hz, 2H, H-3, H-5), 6.35 (d, $^3J_{HH}$ = 10.0 Hz, 2H, H-2, H-6), 2.15-2.00 (m, 2H, H-7), 1.37-1.20 (m, 2H, H-8), 0.96 (t, $^3J_{HH}$ = 7.3 Hz, 3H, H-9) ppm.

^{13}C-NMR (75.53 MHz, $CDCl_3$,): δ = 186.4 (C-1), 154.6 (C-3, C-5), 140.3 (C-10), 129.1 (C-12, C-14), 128.6 (C-2, C-6), 127.8 (C-13), 126.7 (C-11, C-15), 49.4 (C-4), 39.8 (C-7), 18.1 (C-8), 14.6 (C-9) ppm.

2.25 3'-Bromo-1-methyl-2,3-dihydro-[1,1'-biphenyl]-4(1*H*)-one (rac-2l)

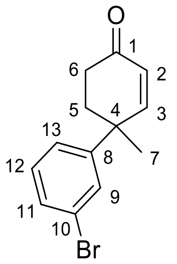

Compound ***rac*-2l** was prepared according to General Procedure 1 with 1.33 mL (1.99 g, 10 mmol, 1 eq) 3'-bromoacetophenone as starting material. The vinylether was purified via column chromatography [500 mL silica, CH/EtOAc 80/1 – CH/EtOAc 50/1, fraction size: 100 mL].
Vinylether:

Yield 1.55 g (20% w/w PPh_3, 1.24 g, 5.5 mmol, 55%); colourless oil

R$_f$ 0.25 (CH/EtOAc 50/1, $KMnO_4$)

GC-MS 5.67 min [228/226 (56%), 132 (100%), 104 (43%)]

Following General Procedure 1, 1.50 g (1.20 g, 5.3 mmol, 1 eq) vinylether (contains 20% w/w PPh_3) were cyclized. The product was purified via column chromatography [450 mL, silica, CH/EtOAc 12/1 – CH/EtOAc 5/1, fraction size: 100 mL].

$C_{13}H_{13}BrO$ [265.15 g/mol]

Yield 884 mg (3.33 mmol, 63%); orange solid

mp 48 °C

R_f 0.25 (CH/EtOAc 5/1, CAM)

GC-MS 6.88 min [266/264 (25%), 240/238 (21%), 224/222 (32%), 143 (100%), 128 (86%)]

HR-MS (ESD) calc m/z for $C_{13}H_{13}BrO$ [M^+]: 264.01498, found: 264.0138

^{1}H-NMR (300.36 MHz, $CDCl_3$): δ = 7.47 (m, 1H, H-9), 7.40 (d, $^3J_{HH}$ = 7.3 Hz, 1H, H-11), 7.31-7.18 (m, 2H, H-12, H-13), 6.89 (d, $^3J_{HH}$ = 10.1 Hz, 1H, H-3), 6.14 (d, $^3J_{HH}$ = 10.2 Hz, 1H, H-2), 2.49-2.35 (m, 1H, H-6), 2.34-2.25 (m, 1H, H-6), 2.25-2.07 (m, 2H, H-5), 1.55 (s, 3H, H-7) ppm.

^{13}C-NMR (75.53 MHz, $CDCl_3$,): δ = 199.1 (C-1), 156.0 (C-3), 148.0 (C-8), 130.3 (C-12), 130.1 (C-11), 129.6 (C-9), 129.1 (C-2), 125.0 (C-13), 123.1 (C-8), 40.7 (C-4), 38.4 (C-5), 34.6 (C-6), 27.7 (C-7) ppm.

2.26 3'-Bromo-1-methyl-[1,1'-biphenyl]-4(1*H*)-one (1l)

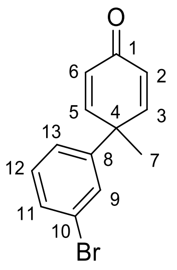

Compound **1l** was prepared according to General Procedure 2 with 796 mg (3.0 mmol, 1eq) ***rac*-2l** as starting material. The product was purified via column chromatography [100 mL silica, CH/EtOAc 10/1 – CH/EtOAc 6/1, fraction size: 20 mL].

$C_{13}H_{11}BrO$ [263.13 g/mol]

Yield 307 mg (1.16 mmol, 39%); orange solid

mp 49-51 °C

R_f 0.25 (CH/EtOAc 4/1, CAM)

GC-MS 6.88 min [264/262 (38%), 236/234 (94%), 183 (30%), 155 (100%), 139.0 (61%)]

HR-MS (ESD) calc m/z for $C_{13}H_{11}BrO$ [M^+]: 261.9993, found: 261.9989

^{1}H-NMR (300.36 MHz, $CDCl_3$): δ = 7.46-7.37 (m, 2H, H-9, H-11), 7.25-7.17 (m, 2H, H-12, H-13), 6.87 (d, $^3J_{HH}$ = 10.0 Hz, 2H, H-3, H-5), 6.29 (d, $^3J_{HH}$ = 10.0 Hz, 2H, H-2, H-6), 1.67 (s, 3H, H-7) ppm.

^{13}**C-NMR** (75.53 MHz, $CDCl_3$,): δ = 185.7 (C-1), 154.5 (C-3, C-5), 142.5 (C-8), 130.9 (C-11), 130.6 (C-12), 129.7 (C-9), 127.6 (C-2, C-6), 125.2 (C-13), 123.3 (C-10), 44.9 (C-4), 24.0 (C-7) ppm.

2.27 3'-Iodo-1-methyl-2,3-dihydro-[1,1'-biphenyl]-4(1*H*)-one (*rac*-2m)

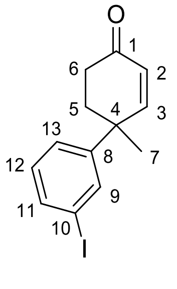

Compound ***rac*-2m** was prepared according to General Procedure 1 with 1.40 mL (2.46 g, 10 mmol, 1 eq) 3'-iodoacetophenone as starting material. The vinylether was purified via column chromatography [500 mL silica, CH/EtOAc 100/1 – CH/EtOAc 60/1, fraction size: 100 mL].
Vinylether:

Yield 1.40 g (22% w/w PPh_3, 1.09 g, 4.0 mmol, 40%); yellow oil

R_f 0.33 (CH/EtOAc 50/1, CAM)

GC-MS 6.02 min [274 (100%), 132 (89%), 104 (37%)]

Following General Procedure 1, 1.35 g (1.05 g, 3.8 mmol, 1 eq) vinylether (contains 22% w/w PPh_3) were cyclized. The product was purified via column chromatography [400 mL silica, CH/EtOAc 10/1 – CH/EtOAc 8/1, fraction size: 80 mL].

$C_{13}H_{13}IO$ [312.15 g/mol]

Yield 720 mg (2.3 mmol, 61%); orange oil

R_f 0.45 (CH/EtOAc 4/1, CAM)

GC-MS 7.18 min [312 (52%), 270 (45%), 143 (79%), 128 (100%)]

HR-MS (ESD) calc m/z for $C_{13}H_{14}IO$ [M+H$^+$]: 313.00893, found: 313.00695

^{1}**H-NMR** (300.36 MHz, $CDCl_3$): δ = 7.66 (s, 1H, H-9), 7.60 (d, $^3J_{HH}$ = 7.8 Hz, 1H, H-11), 7.30 (d, $^3J_{HH}$ = 7.7 Hz, 1H, H-13), 7.08 (t, $^3J_{HH}$ = 7.8 Hz, 1H, H-12), 6.87 (d, $^3J_{HH}$ = 10.1 Hz, 1H, H-3), 6.13 (d, $^3J_{HH}$ = 10.2 Hz, 1H, H-2), 2.47-2.33 (m, 1H, H-6), 2.33-2.24 (m, 1H, H-6), 2.24-2.05 (m, 2H, H-5), 1.53 (s, 3H, H-7) ppm.

^{13}C-NMR (75.53 MHz, $CDCl_3$,): δ = 199.1 (C-1), 156.1 (C-3), 148.0 (C-8), 136.1 (C-11), 135.5 (C-9), 130.5 (C-12), 129.1 (C-2), 125.6 (C-13), 95.1 (C-10), 40.6 (C-4), 38.1 (C-5), 34.6 (C-6), 27.6 (C-7) ppm.

2.28 3'-Iodo-1-methyl-[1,1'-biphenyl]-4(1*H*)-one (1m)

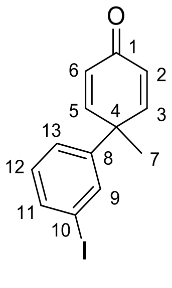

Compound **1m** was prepared according to General Procedure 2 with 633 mg (2.0 mmol, 1eq) ***rac*-2m** as starting material. The product was purified via column chromatography [50 mL silica, CH/EtOAc 10/1 – CH/EtOAc 8/1, fraction size: 10 mL].

**$C_{13}H_{11}IO$**	[310.13 g/mol]
**Yield**	396 mg (1.28 mmol, 63%); orange solid
**mp**	68 °C
**R_f**	0.33 (CH/EtOAc 4/1, CAM)
**GC-MS**	7.25 min [310 (48%), 282 (100%), 267 (19%), 183 (29%), 155 (42%), 139 (42%)]
**HR-MS** (ESD)	calc m/z for $C_{13}H_{11}IO$ [$M+H^+$]: 310.9933, found: 310.9933

^{1}H-NMR (300.36 MHz, $CDCl_3$): δ = 7.67-7.57 (m, 2H, H-9, H-11), 7.27 (d, $^3J_{HH}$ = 7.5 Hz, 1H, H-13), 7.09 (t, $^3J_{HH}$ = 8.0 Hz, 1H, H-12), 6.88 (d, $^3J_{HH}$ = 10.0 Hz, 2H, H-3, H-5), 6.30 (d, $^3J_{HH}$ = 10.0 Hz, 2H, H-2, H-6), 1.67 (s, 3H, H-7) ppm.

^{13}C-NMR (75.53 MHz, $CDCl_3$,): δ = 185.7 (C-1), 154.6 (C-3, C-5), 142.6 (C-8), 136.9 (C-9/C-11), 135.6 (C-9/C-11), 130.8 (C-12), 127.5 (C-2, C-6), 125.9 (C-13), 95.1 (C-10), 44.8 (C-4), 24.0 (C-7) ppm.

2.29 1-Methyl-3'-nitro-2,3-dihydro-[1,1'-biphenyl]-4(1*H*)-one (*rac*-2n)

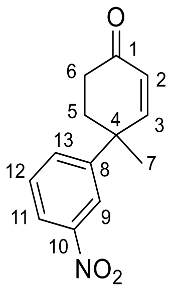

Compound ***rac*-2n** was prepared according to General Procedure 1 with 1.65 g (10 mmol, 1 eq) 3'-nitroacetophenone as starting material. The vinylether was purified via column chromatography [500 mL silica, CH/EtOAc 20/1 – CH/EtOAc 16/1, 100 mL fraction size].

Vinylether:

Yield 1.03 g (5.3 mmol, 53%); yellow oil

R_f 0.43 (CH/EtOAc 50/1, $KMnO_4$)

GC-MS 6.12 min [193 (100%), 132 (42%), 103 (34%), 77 (28%)]

Following General Procedure 1, 983 mg (983 mg, 5 mmol, 1 eq) vinylether were cyclized. The product was purified via column chromatography [400 mL silica, CH/EtOAc 4/1 – CH/EtOAc 3/1, fraction size: 75 mL].

$C_{13}H_{13}NO_3$ [231.25 g/mol]

Yield 405 mg (1.75 mmol, 35%); brown solid

R_f 0.36 (CH/EtOAc 2/1, CAM)

GC-MS 7.29 min [231 (21%), 207 (79%), 189 (100%), 172 (36%), 128 (79%)]

HR-MS (ESD) calc m/z for $C_{13}H_{13}NO_3$ [M+H$^+$]: 232.09737, found: 232.09738

^{1}H-NMR (300.36 MHz, $CDCl_3$): δ = 8.23 (s, 1H, H-9), 8.14 (d, $^3J_{HH}$ = 8.0 Hz, 1H, H-11), 7.68 (d, $^3J_{HH}$ = 7.7 Hz, 1H, H-13), 7.54 (t, $^3J_{HH}$ = 8.0 Hz, 1H, H-12), 6.93 (d, $^3J_{HH}$ = 10.2 Hz, 1H, H-3), 6.19 (d, $^3J_{HH}$ = 10.2 Hz, 1H, H-2), 2.54-2.38 (m, 1H, H-6), 2.37-2.12 (m, 3H, H-5, H-6), 1.62 (s, 3H, H-7) ppm.

^{13}C-NMR (75.53 MHz, $CDCl_3$,): δ = 198.5 (C-1), 155.2 (C-3), 148.7 (C-10), 147.9 (C-8), 132.6 (C-13), 129.8 (C-12), 129.6 (C-2), 122.2 (C-11), 121.3 (C-9), 40.9 (C-4), 38.1 (C-5), 34.5 (C-6), 27.6 (C-7) ppm.

2.30 1-Methyl-3'-nitro-[1,1'-biphenyl]-4(1*H*)-one (1n)

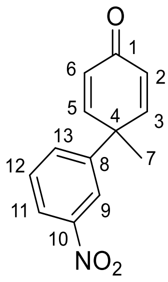

Compound **1n** was prepared according to General Procedure 2 with 336 mg (1.45 mmol, 1 eq) ***rac*-2n** as starting material. The product was purified via column chromatography [50 mL silica, CH/EtOAc 4/1-CH/EtOAc 3/1, fraction size: 10 mL].

$C_{13}H_{11}NO_3$ [229.24 g/mol]

Yield 185 mg (0.81 mmol, 56%); orange solid

mp 100-102 °C

R_f 0.30 (CH/EtOAc 2/1, CAM)

GC-MS 7.32 min [229 (22%), 201 (100%), 153 (45%), 128 (45%)]

HR-MS (ESD) calc m/z for $C_{13}H_{11}NO_3$ [M+H$^+$]: 230.08172, found: 230.08173

^{1}H-NMR (300.36 MHz, $CDCl_3$): δ = 8.14 (s, 1H, H-9), 8.09 (d, $^3J_{HH}$ = 7.1 Hz, 1H, H-11), 7.56 (d, $^3J_{HH}$ = 7.8 Hz, 1H, H-13), 7.48 (t, $^3J_{HH}$ = 7.9 Hz, 1H, H-12), 6.84 (d, $^3J_{HH}$ = 9.9 Hz, 2H, H-3, H-5), 6.28 (d, $^3J_{HH}$ = 9.8 Hz, 2H, H-2, H-6), 1.71 (s, 3H, H-7) ppm.

^{13}C-NMR (75.53 MHz, $CDCl_3$,): δ = 185.3 (C-1), 153.6 (C-3, C-5), 148.8 (C-10), 142.7 (C-8), 133.0 (C-13), 130.1 (C-12), 128.1 (C-2, C-6), 122.9 (C-10), 121.4 (C-9), 44.8 (C-4), 24.1 (C-7) ppm.

2.31 3'-Methoxy-1-methyl-2,3-dihydro-[1,1'-biphenyl]-4(1*H*)-one (*rac*-2o)

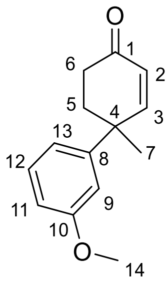

Compound ***rac*-2o** was prepared according to General Procedure 1 with 1.40 mL (1.50 g, 10 mmol, 1 eq) 3'-methoxyacetophenone as starting material. The vinylether was purified via

column chromatography [500 mL silica, CH/EtOAc 80/1 – CH/EtOAc 40/1, fraction size: 100 mL].

Vinylether:

**Yield**	1.60 g (22% w/w PPh_3, 1.25 g, 7.0 mmol, 70%); yellow oil
**R_f**	0.25 (CH/EtOAc 50/1, CAM)
**GC-MS**	5.51 min [178 (100%), 163 (20%), 135 (50%), 105 (27%)]

Following General Procedure 1, 1.52 g (1.19 g, 6.7 mmol, 1 eq) vinylether (contains 22% w/w PPh_3) were cyclized. The product was purified via column chromatography [400 mL silica, CH/EtOAc 6/1, fraction size: 75 mL].

**$C_{14}H_{16}O_2$**	[216.28 g/mol]
**Yield**	847 mg (3.92 mmol, 59%); brown oil
**R_f**	0.43 (CH/EtOAc 4/1, CAM)
**GC-MS**	6.69 min [216 (100%), 188 (35%), 159 (69%), 143 (20%), 115 (42%)]
**HR-MS**	calc m/z for $C_{14}H_{16}O_2$ $[M+H^+]$: 217.12285, found: 217.12287

^{1}H-NMR (300.36 MHz, $CDCl_3$): δ = 7.28 (t, $^3J_{HH}$ = 7.9 Hz, 1H, H-12), 6.96-6.84 (m, 3H, H-3, H-9, H-11), 6.80 (d, $^3J_{HH}$ = 8.1 Hz, 1H, H-13), 6.11 (d, $^3J_{HH}$ = 10.2 Hz, 1H, H-2), 3.81 (s, 3H, H-14), 2.42-2.32 (m, 1H, H-6), 2.32-2.20 (m, 2H, H-5, H-6), 2.19-2.03 (m, 1H, H-5), 1.54 (s, 3H, H-7) ppm.

^{13}C-NMR (75.53 MHz, $CDCl_3$,): δ = 199.6 (C-1), 159.9 (C-10), 157.0 (C-3), 147.2 (C-8), 129.7 (C-12), 128.7 (C-2), 118.7 (C-11), 113.1 (C-9), 111.4 (C-13), 55.4 (C-14), 40.7 (C-4), 38.2 (C-5), 34.8 (C-6), 27.8 (C-7) ppm.

2.32 3'-Methoxy-1-methyl-[1,1'-biphenyl]-4(1*H*)-one (1o)

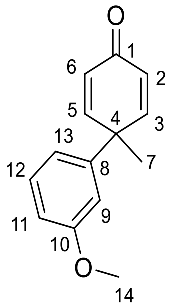

Compound **1o** was prepared according to General Procedure 2 with 742 mg (3.43 mmol, 1 eq) ***rac*-2o** as starting material. The product was purified via column chromatography [100 mL silica, CH/EtOAc 8/1 – CH/EtOAc 5/1, fraction size: 20 mL].

$C_{14}H_{14}O_2$ [214.26 g/mol]; (CAS: 2378808-60-5)

Yield 368 mg (1.7 mmol, 50%); orange solid

mp 68 °C

R_f 0.31 (CH/EtOAc 4/1, $KMnO_4$)

GC-MS: 6.76 min [214 (90%), 186 (100%), 171 (85%), 155 (31%) 128 (66%)]

^{1}H-NMR (300.36 MHz, $CDCl_3$): δ = 7.28 (t, $^3J_{HH}$ = 7.7 Hz, 1H, H-12), 6.97-6.87 (m, 3H, H-3, H-5, H-13), 6.87-6.77 (m, 2H, H-9, H-11), 6.28 (d, $^3J_{HH}$ = 9.8 Hz, 2H, H-2, H-6), 3.80 (s, 3H, H-14), 1.68 (s, 3H, H-7) ppm.

^{13}C-NMR (75.53 MHz, $CDCl_3$,): δ = 186.0 (C-1), 160.1 (C-10), 155.4 (C-3, C-5), 141.7 (C-8), 130.1 (C-12), 127.1 (C-2, C-6), 118.7 (C-13), 113.0 (C-9/C-11), 112.5 (C-9/ C-11), 55.4 (C-14), 45.1 (C-4), 24.0 (C-7) ppm.

The recorded spectra are in accordance with those reported in the literature.[8]

2.33 1,2’-Dimethyl-2,3-dihydro-[1,1’-biphenyl]-4(1*H*)-one (*rac*-2p)

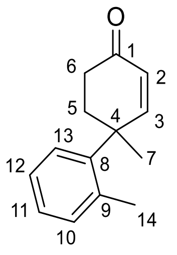

Compound ***rac*-2p** was prepared according to General Procedure 1 with 1.34 g (10 mmol, 1 eq) 2’-methylacetophenone as starting material. The vinylether was purified via column chromatography [500 mL silica, CH/EtOAc 80/1 – CH/EtOAc 20/1, fraction size: 100 mL].

Vinylether:

Yield 1.03 g (25% w/w PPh_3, 0.77 g, 4.7 mmol, 47%); yellow liquid

R_f 0.54 (CH/EtOAc 40/1, $KMnO_4$)

GC-MS 4.74 min [162 (100%), 147 (20%), 130 (37%), 115 (40%)]

Following General Procedure 1, 995 mg (746 mg, 4.6 mmol, 1 eq) vinylether (contains 25% w/w PPh_3) were cyclized. The product was purified via column chromatography [500 mL silica, CH/EtOAc 20/1 – CH/EtOAc 10/1, fraction size:100 mL].

$C_{14}H_{16}O$ [200.28 g/mol]; (CAS: 1914982-01-6)

Yield 296 mg (1.5 mmol, 33%); brown solid

mp 56 °C

R_f 0.51 (CH/EtOAc 4/1, $KMnO_4$)

GC-MS 6.49 min [200 (67%), 185 (15%), 158 (26%), 143 (100%), 129 (63%)]

^{1}H-NMR (300.36 MHz, $CDCl_3$): δ = 7.38-7.30 (m, 1H, H_{Ar}), 7.23-7.13 (m, 3H, H_{Ar}), 7.04 (d, $^3J_{HH}$ = 10.1 Hz, 1H, H-3), 6.05 (d, $^3J_{HH}$ = 10.2 Hz, 1H, H-2), 2.68-2.49 (m, 2H, H-6), 2.49-2.33 (m, 4H, H-5, H-14), 2.06-1.92 (m, 1H, H-5), 1.68 (s, 3H, H-7) ppm.

^{13}C-NMR (75.53 MHz, $CDCl_3$,): δ = 199.2 (C-1), 160.0 (C-3), 143.4 (C-8), 136.3 (C-9), 133.2 (C_{Ar}), 127.3 (C-2), 127.2 (C_{Ar}), 127.0 (C_{Ar}), 126.3 (C_{Ar}), 41.4 (C-4), 35.5 (C-6), 35.0 (C-5), 26.40 (C-14), 22.87 (C-14) ppm.

The recorded spectra are in accordance with those reported in the literature.[9]

2.34 1,2'-Dimethyl-[1,1'-biphenyl]-4(1*H*)-one (1p)

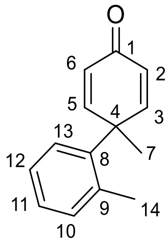

Compound **1p** was prepared according to General Procedure 2 with 257 mg (1.28 mmol, 1 eq) ***rac*-2p** as starting material. The product was purified via column chromatography [100 mL silica, CH/EtOAc 12/1 – CH/EtOAc 6/1, fraction size: 20 mL].

$C_{14}H_{14}O$ [198.27 g/mol]; (CAS: 1914981-58-0)

Yield 63 mg (0.32 mmol, 25%); yellowish solid

mp 118-120 °C

R_f 0.23 (CH/EtOAc 4/1, $KMnO_4$)

GC-MS 6.49 min [198 (100%), 155 (44%), 128 (28%)]

^{1}H-NMR (300.36 MHz, $CDCl_3$): δ = 7.45 (d, $^3J_{HH}$ = 8.7 Hz, 1H, H-13), 7.33-7.19 (m, 2H, H-11, H-12), 7.11 (d, $^3J_{HH}$ = 6.4 Hz, 1H, H-10), 6.91 (d, $^3J_{HH}$ = 10.0 Hz , 2H, H-3, H-5), 6.37 (d, $^3J_{HH}$ = 9.9 Hz , 2H, H-2, H-6), 2.18 (s, 3H, H-14), 1.71 (s, 3H, H-7) ppm.

^{13}C-NMR (75.53 MHz, $CDCl_3$,): δ = 185.7 (C-1), 155.7 (C-3, C-5), 138.6 (C-8), 137.7 (C-9), 132.4 (C-10), 128.2 (C-2, C-6), 128.2 (C-11, C-12), 126.9 (C-11, C-12), 126.2 (C-13), 46.0 (C-4), 27.4 (C-7), 20.3 (C-14) ppm.
The recorded spectra are in accordance with those reported in the literature.[9]

2.35 General Procedure 3

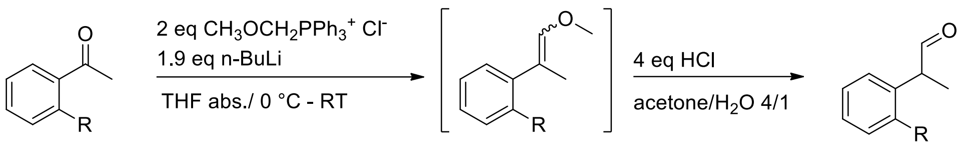

3

In a flame dried 100 mL Schlenk flask a solution of 6.86 g (20 mmol, 2 eq) methoxymethyltriphenylphosphonium chloride in 45 mL THF abs. was prepared and cooled in an ice/water bath. 7.6 mL (19 mmol, 1.9 eq) n-BuLi (2.5M in THF) were added dropwise. The resulting red solution was stirred at 0 °C for 1 h. Then the corresponding ketone (10 mmol, 1 eq) was added. The reaction mixture was stirred overnight. After GC-MS indicated full conversion, 10 g silica were added and the crude product was adsorbed onto silica by removal of the solvent under reduced pressure. Purification of the adsorbed crude product *via* column chromatography yielded the corresponding vinyl ethers as diastereomeric mixtures.
In a round bottom flask a 0.9M solution of the vinyl ether intermediate in a 4/1 acetone/H_2O mixture was prepared. After addition of 4 eq 6M HCl, the reaction mixture was stirred overnight at RT. Full conversion was indicated by GC-MS and the reaction mixture was quenched by the addition of $NaHCO_3$ sat. (3.5 mL/mmol substrate). The resulting mixture was extracted with EtOAc (3x 2 mL/mmol substrate). The combined organic layers were dried over Na_2SO_4 and the solvent was removed under reduced pressure. The obtained crude product was used without further purification.

2.36 2-(2-Methoxyphenyl)propanal (3q)

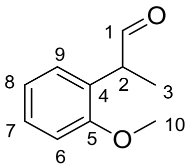

Compound **3q** was prepared according to General Procedure 3 with 1.50 g (10 mmol, 1 eq) 2'-methoxyacetophenone as starting material. The vinylether was purified via column chromatography [500 mL silica, CH/EtOAc 16/1 – CH/EtOAc 6/1, fraction size: 100 mL].

Vinylether:

Yield 899 mg (10% w/w PPh_3, 809 mg, 4.5 mmol, 45%); yellow oil

R_f 0.16 (CH/EtOAc 50/1, CAM)

GC-MS 5.23 min [178 (100%), 163 (12%), 135 (77%), 120 (58%), 91 (58%)]

Following General Procedure 3, 836 mg (746 mg, 4.6 mmol, 1 eq) vinylether (contains 10% w/w PPh_3) were hydrolyzed.

$C_{10}H_{12}O_2$ [164.20 g/mol]

Yield 740 mg (4.5 mmol, 98%), yellow oil

GC-MS 5.05 min [164 (14%), 135 (100%), 120 (7%), 105 (24%)]

^{1}H-NMR (300.36 MHz, $CDCl_3$): δ = 9.67 (s, 1H, H-1), 7.29 (t, $^3J_{HH}$ = 7.1 Hz, 1H, H-7), 7.12 (d, $^3J_{HH}$ = 7.4 Hz, 1H, H-9), 6.97 (t, $^3J_{HH}$ = 7.6 Hz, 1H, H-8), 6.92 (d, $^3J_{HH}$ = 8.2 Hz , 1H, H-6), 3.94-3.71 (m, 4H, H-2, H-10), 1.39 (d, $^3J_{HH}$ = 7.1 Hz, 3H, H-3) ppm.

^{13}C-NMR (75.53 MHz, $CDCl_3$,): δ = 202.1 (C-1), 157.3 (C-5), 129.3 (C-9), 128.8 (C-7), 127.2 (C-4), 121.1 (C-8), 110.9 (C-6), 55.5 (C-10), 47.5 (C-2), 13.6 (C-3) ppm.

2.37 2'-Methoxy-1-methyl-2,3-dihydro-[1,1'-biphenyl]-4(1*H*)-one (*rac*-2q)

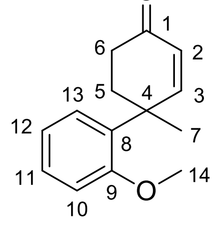

In a 25 mL round-bottom flask a solution of 717 mg (4.37 mmol, 1 eq) **3q** in 6 mL TBME was prepared. The solution was cooled in an ice/water bath. Then 0.37 mL (306 mg, 4.37 mmol, 1 eq) MVK and a solution of 98 mg (1.75 mmol, 0.4 eq) KOH in 1.2 mL EtOH were added. The rersulting solution was allowed to warm to RT and was stirred overnight at this temperature. Full conversion was indicated by GC-MS. For workup the reaction was diluted with EtOAc (20

mL) and washed with 1M HCl (10 mL). The organic layer was washed with H_2O (5 mL) and brine (5 mL). The organic layer was dried over Na_2SO_4. Removal of the solvent under reduced pressure yielded 1.1 g crude product, which was purified *via* column chromatography [200 mL silica, CH/EtOAc 12/1 - CH/EtOAc 8/1, fraction size: 50 mL].

$C_{14}H_{16}O_2$ [216.28 g/mol]; (CAS: 138371-79-6)

Yield 300 mg (1.39 mmol, 32%); yellow oil

GC-MS 6.64 min [216 (100%), 201 (34%), 174 (34%), 159 (84%), 131 (25%), 115 (47%)]

^{1}H-NMR (300.36 MHz, $CDCl_3$) δ = 7.31-7.16 (m, 2H, H-11, H-13), 7.09 (d, $^3J_{HH}$ = 10.2 Hz, 1H, H-3), 6.99-6.83 (m, 2H, H-10, H-12), 7.09 (d, $^3J_{HH}$ = 10.2 Hz, 1H, H-2), 3.83 (s, 3H, H-14), 2.83-2.69 (m, 1H, H-6), 2.53-2.39 (m, 1H, H-5), 2.37-2.22 (m, 1H, H-5), 2.00-1.87 (m, 1H, H-6), 1.60 (s, 3H, H-7) ppm.

^{13}C-NMR (75.53 MHz, $CDCl_3$) δ = 200.2 (C-1), 159.6 (C-3), 158.1 (C-9), 132.9 (C-8), 128.5 (C-11/C-13), 127.6 (C-11/C-13), 126.9 (C-2), 120.6 (C-12), 112.0 (C-10), 55.2 (C-14), 40.3 (C-4), 35.3 (C-5), 34.3 (C-6), 26.1 (C-7) ppm.

The recorded spectra are in accordance with those reported in the literature.[8]

2.38 2'-Methoxy-1-methyl-1[1,1'-biphenyl]-4(1*H*)-one (1q)

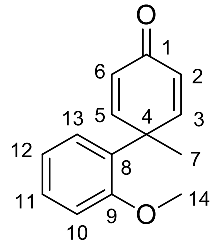

Compound **1q** was prepared according to General Procedure 2 with 271 mg (1.25 mmol, 1 eq) ***rac*-2q** as starting material. The product was purified via column chromatography [100 mL silica, CH/EtOAc 14/1 – CH/EtOAc 4/1, fraction size: 15 mL].

$C_{14}H_{14}O_2$ [214.26 g/mol]; (CAS: 2243282-32-6)

Yield 104 mg (0.49 mmol, 39%); orange solid

mp 140 °C

R_f 0.31 (CH/EtOAc 4/1, CAM)

GC-MS 6.72 min [214 (100%), 186 (14%), 171 (41%), 152 (21%), 128 (41%)]

^{1}H-NMR (300.36 MHz, $CDCl_3$): δ = 7.35-7.19 (m, 2H, H-11, H-13), 7.08 (d, $^3J_{HH}$ = 10.0 Hz 2H, H-3, H-5), 6.95 (t, $^3J_{HH}$ = 7.5 Hz, 1H, H-12), 6.88 (d, $^3J_{HH}$ = 8.0 Hz, 1H, H-10), 6.28 (d, $^3J_{HH}$ = 10.0 Hz , 2H, H-2, H-6), 3.75 (s, 3H, H-14), 1.68 (s, 3H, H-7) ppm.
^{13}C-NMR (75.53 MHz, $CDCl_3$,): δ = 186.4 (C-1), 158.5 (C-9), 155.1 (C-3, C-5), 129.3 (C-11/C-13), 128.9 (C-8), 127.5 (C-11/C-13), 127.2 (C-2, C-6), 121.1 (C-12), 111.9 (C-10), 55.3 (C-14), 44.2 (C-4), 24.8 (C-7) ppm.
The recorded spectra are in accordance with those reported in the literature.[8]

2.39 (2-Chlorophenyl)propanal (3r)

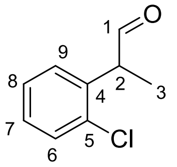

Compound **3r** was prepared according to General Procedure 3 with 1.55 g (10 mmol, 1 eq) 2'-chloroacetophenone as starting material. The vinylether was purified via column chromatography [500 mL silica, CH/EtOAc 80/1 – CH/EtOAc 40/1, fraction size: 100 mL].
Vinylether:

Yield 1.51 g (33% w/w PPh_3, 1.01 g, 5.5 mmol, 55%); yellow liquid
R_f 0.54 (CH/EtOAc 20/1, CAM)
GC-MS 5.05 min [182 (100%), 167 (7%), 139 (46%), 103 (93%)]

Following General Procedure 3, 1.33 g (891 mg, 5.2 mmol, 1 eq) vinylether (contains 33% w/w PPh_3) were hydrolyzed.

C_9H_9ClO [168.62]
Yield 1.27 g (40% w/w PPh_3, 762 mg, 4.5 mmol, 87% crude); yellow liquid
GC-MS 4.86 min [168 (14%), 139 (100%), 103 (71%)]
^{1}H-NMR (300.36 MHz, $CDCl_3$): δ = 9.74 (s, 1H, H-1), 7.46 (d, $^3J_{HH}$ = 5.6 Hz, 1H, H-6), 7.31-7.21 (m, 2H, H-7, H-8), 7.15 (d, $^3J_{HH}$ = 9.1 Hz, 1H, H-9), 4.15 (q, $^3J_{HH}$ = 7.0 Hz, 1H, H-2), 1.45 (d, $^3J_{HH}$ = 7.1 Hz, 3H, H-3) ppm.
^{13}C-NMR (75.53 MHz, $CDCl_3$,): δ = 200.4 (C-1), 136.2 (C-5), 134.5 (C-4), 130.2 (C-6), 129.4 (C-9), 129.0 (C-7), 127.6 (C-8), 49.7 (C-2), 13.9 (C-3) ppm.

2.40 2'-Chloro-1-methyl-2,3-dihydro-[1,1'-biphenyl]-4-(1*H*)-one (*rac*-2r)

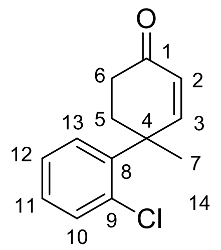

In a 25 mL round-bottom flask a solution of 1.22 g (732 mg, 4.4 mmol, 1 eq) **3r** (contains 40% w/w PPh_3) in 10 mL TBME was prepared. The solution was cooled in an ice bath. Then 0.6 mL (508 mg, 7.25 mmol, 1.6 eq) MVK and a solution of 162 mg (2.89 mmol, 0.4 eq) KOH in 2 mL EtOH were added. The resulting solution was allowed to warm to RT and was stirred overnight. Full conversion was indicated by GC-MS. For workup the reaction was diluted with EtOAc (20 mL) and washed with 1M HCl (8 mL). The organic layer was washed with H_2O (8 mL) and brine (8 mL) and dried over Na_2SO_4. After removal of the solvent under reduced pressure the crude product was purified *via* column chromatography [500 mL silica, CH/EtOAc 12/1 - CH/EtOAc 8/1, fraction size: 100 mL].

**$C_{13}H_{13}ClO$**	[220.69 g/mol]
**Yield**	235 mg (1.06 mmol, 24%); yellow solid
**mp**	90-93 °C
**GC-MS**	6.64 min [220 (14%), 178 (66%), 163 (28%), 143 (100%), 128 (62%)]
**HR-MS**	calc m/z for $C_{13}H_{13}ClO$ [M+H$^+$]: 221.07331, found: 221.07332

^{1}H-NMR (300.36 MHz, $CDCl_3$) δ = 7.43-7.31 (m, 2H, H-Ar), 7.25-7.16 (m, 2H, H-Ar), 7.06 (d, $^3J_{HH}$ = 10.2 Hz, 1H, H-3), 6.07 (d, $^3J_{HH}$ = 10.2 Hz, 1H, H-2), 3.03-2.88 (m, 1H, H-6), 2.59-2.43 (m, 1H, H-5), 2.41-2.24 (m, 1H, H-5), 2.05-1.89 (m, 1H, H-6), 1.72 (s, 3H, H-7) ppm.

^{13}C-NMR (75.53 MHz, $CDCl_3$) δ = 199.2 (C-1), 158.2 (C-3), 141.9 (C-9), 133.6 (C-8), 129.0 (C-Ar), 128.6 (C-Ar), 127.7 (C-2), 127.1 (C-Ar), 41.6 (C-4), 35.0 (C-5), 34.1 (C-6), 26.0 (C-7) ppm.

2.41 2'-Chloro-1-methyl-[1,1'-biphenyl]-4(1*H*)-one (1r)

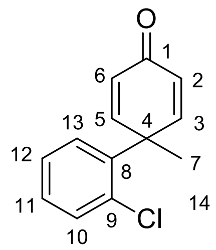

Compound **1r** was prepared according to General Procedure 2 with 144 mg (0.65 mmol, 1 eq) ***rac*-2r** as starting material. The product was purified via column chromatography [50 mL silica, CH/EtOAc 10/1 – CH/EtOAc 4/1, fraction size: 10 mL].

**$C_{13}H_{11}ClO$**	[218.68 g/mol]
**Yield**	53 mg (0.24 mmol, 37%); orange solid
**mp**	115-118 °C
**R_f**	0.27 (CH/EtOAc 4/1, CAM)
**GC-MS**	6.74 min [218 (20%), 198 (100%), 175 (27%), 155 (87%), 139 (33%)]
**HR-MS**	calc m/z for $C_{13}H_{11}ClO$ [M^+]: 218.0498, found: 218.0494

^{1}H-NMR (300.36 MHz, $CDCl_3$): δ = 7.37 (d, $^3J_{HH}$ = 9.0 Hz, 1H, H-10), 7.28-7.11 (m, 3H, H-11, H-12, H-13), 6.86 (d, $^3J_{HH}$ = 10.0 Hz, 2H, H-3, H-5), 6.26 (d, $^3J_{HH}$ = 10.0 Hz , 2H, H-2, H-6), 1.64 (s, 3H, H-7) ppm.

^{13}C-NMR (75.53 MHz, $CDCl_3$,): δ = 185.8 (C-1), 153.7 (C-3, C-5), 137.7 (C-9), 135.0 (C-8), 131.9 (C-Ar), 129.5 (C-Ar), 128.7 (C-2, C-6), 128.3 (C-Ar), 127.6 (C-Ar), 45.6 (C-4), 26.6 (C-7) ppm.

2.42 4-Methyl-4-(naphthalen-2-yl)cyclohex-2-enone (*rac*-2s)

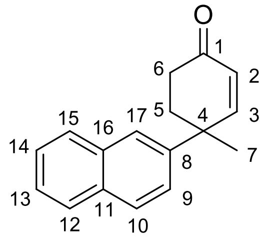

Compound ***rac*-2s** was prepared according to General Procedure 1 with 1.70 g (10 mmol, 1 eq) 2'-naphthylacetophenone as starting material. The vinylether was purified via column chromatography [500 mL silica, CH/EtOAc 60/1 – CH/EtOAc 30/1, fraction size: 100 mL].

Vinylether:

Yield 1.89 g (24% w/w PPh_3, 1.44 g, 7.3 mmol, 73%); yellow liquid

R_f 0.34 (CH/EtOAc 40/1, $KMnO_4$)

GC-MS 6.50 min [198 (100%), 183 (33%), 155 (89%), 128 (10%)]

Following General Procedure 1, 1.84 g (1.40 g, 7.1 mmol, 1 eq) vinylether (contains 24% w/w PPh_3) were cyclized. The product was purified via column chromatography [500 mL silica, CH/EtOAc 25/1 – CH/EtOAc 14/1, fraction size:100 mL].

$C_{17}H_{16}O$ [200.28 g/mol]; (CAS: 153167-72-7)

Yield 1.1 g (4.65 mmol, 65%); brown oil

R_f 0.16 (CH/EtOAc 6/1, $KMnO_4$)

GC-MS: 7.65 min [236 (100%), 221 (62%), 194 (31%), 179 (100%), 152 (33%)]

^{1}H-NMR (300.36 MHz, $CDCl_3$): δ = 7.91-7.76 (m, 3H, H-Ar), 7.72 (s, 1H, H-Ar), 7.56-7.41 (m, 3H, H-Ar), 7.04 (d, $^3J_{HH}$ = 10.1 Hz, 1H, H-3), 6.21 (d, $^3J_{HH}$ = 10.1 Hz, 1H, H-2), 2.49-2.29 (m, 3H, H-5, H-6), 2.29-2.12 (m, 1H, H-5), 1.66 (s, 3H, H-7) ppm.

^{13}C-NMR (75.53 MHz, $CDCl_3$) δ = 199.6 (C-1), 157.1 (C-3), 142.6 (C-8), 133.3 (C-Ar), 132.3 (C-Ar), 128.9 (C-2), 128.7 (C-Ar), 128.1 (C-Ar), 127.6 (C-Ar), 126.5 (C-Ar), 126.2 (C-Ar), 125.1 (C-Ar), 124.4 (C-Ar), 40.9 (C-4), 38.0 (C-5), 34.8 (C-6), 27.8 (C-7) ppm.

The recorded spectra are in accordance with those reported in the literature.[8]

2.43 4-Methyl-4-(naphthalen-2-yl)cyclohexa-2,5-dienone (1s)

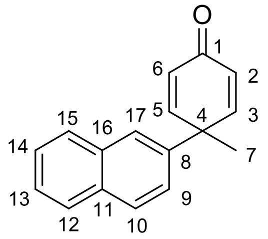

Compound **1s** was prepared according to General Procedure 2 with 1.10 g (4.43 mmol, 1 eq) ***rac*-2s** as starting material. The product was purified via column chromatography [250 mL silica, CH/EtOAc 4/1, fraction size: 75 mL].

$C_{17}H_{14}O$ [234.29 g/mol]; (CAS: 1914981-60-4)

Yield 434 mg (1.85 mmol, 42%); orange solid

mp 78-79 °C

R_f 0.23 (CH/EtOAc 4/1, CAM)

GC-MS 7.73 min [234 (100%), 206 (29%), 191 (29%), 165 (13%)]

^{1}H-NMR (300.36 MHz, $CDCl_3$): δ = 7.89-7.70 (m, 4H, H-Ar), 7.56-7.42 (m, 2H, H-Ar), 7.35 (d, $^3J_{HH}$ = 10.4 Hz, 1H, H-Ar), 6.98 (d, $^3J_{HH}$ = 10.0 Hz , 2H, H-3, H-5), 6.34 (d, $^3J_{HH}$ = 10.0 Hz, 2H, H-2, H-6), 1.81 (s, 3H, H-7) ppm.

^{13}C-NMR (75.53 MHz, $CDCl_3$,): δ = 186.1 (C-1), 155.5 (C-3, C-5), 137.4 (C-8), 133.7 (C-11/C-16), 132.7 (C-11/C-16), 128.8 (C-Ar), 128.1 (C-Ar), 127.7 (C-Ar), 127.4 (C-2, C-6), 126.7 (C-Ar), 126.5 (C-Ar), 125.0 (C-Ar), 124.7 (C-Ar), 45.3 (C-4), 24.0 (C-7) ppm.

The recorded spectra are in accordance with those reported in the literature.[8]

2.44 1-Methylcyclohexa-2,5-diene-1-carbonitrile (3t)

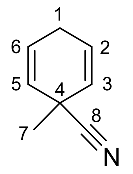

A 250 mL 3-necked round bottom flask equipped with a gas induction tube was inertized and flushed with argon. After immersing this flask into a N_2/DCM cooling bath, 100 mL NH_3 were condensed into the flask. Then 510 mg (73.5 mmol, 2.5 eq) Li were added in pieces to the ammonia, giving an intense blue colour. The mixture was stirred at -78 °C for 30 min in order to ensure complete dissolution of the Li. Then a solution of 3.0 mL (29.4 mmol, 1 eq) benzonitrile in 30 mL THF abs. and a solution of 2.8 mL (29.4 mmol, 1 eq) *tert*-BuOH in 10 mL THF abs. were added in quick succession. The resulting orange solution was kept at -78 °C for 2 h. Then, the formed carbanion was quenched by the addition of 3.7 mL (58.76 mmol, 2 eq) MeI, which led to decoloration of the reaction mixture. The reaction was kept at -78 °C for further 30 min. Then it was quenched by the addition of 12.6 g solid NH_4Cl in portions. The mixture was then allowed to warm to RT overnight, to remove the ammonia. Full conversion was indicated by GC-MS. For workup, brine (15 mL), H_2O (15 mL) and Et_2O (30 mL) were added. After phase separation, the aqueous layer was extracted with Et_2O (3x30 mL). The combined organic layers were washed with brine (15 mL) and dried over Na_2SO_4. The solvent

was carefully removed under reduced pressure. Due to the volatility of the product the crude product contained just 76% w/w of the actual nitrile, while the rest was represented by THF.

C_8H_9O [119.16 g/mol]; (CAS: 109954-32-7)

Yield 3.88 g (2.95 g (76% w/w), 22.2 mmol, 75%); orange oil

GC-MS 3.87 min [119 (17%), 104 (100%), 77 (27%)]

^{1}H-NMR (300.36 MHz, $CDCl_3$): δ = 5.9 (d, $^3J_{HH}$ = 10.2 Hz, 2H, H-2, H-6), 5.7 (d, $^3J_{HH}$ = 10.2 Hz, 2H, H-3, H-5), 2.7-2.6 (m, 2H, H-1), 1.5 (s, 3H, H-7) ppm.

^{13}C-NMR (75.53 MHz, $CDCl_3$,): δ = 126.5 (C-2, C-6), 125.7 (C-3, C-5), 122.5 (C-8), 31.3 (C-4), 28.7 (C-7), 25.5 (C-1) ppm.

2.45 1-Methyl-4-oxocyclohexa-2,5-dienecarbonitrile (1t)

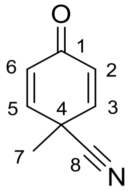

In a 250 mL round bottom flask a solution of 1.94 g (1.48 g, 12.4 mmol, 1 eq) **3t** (contains 24% w/w THF) in a 1/1 mixture of Ac_2O and AcOH (20 mL) was prepared and cooled in an ice bath. Then 20 mL benzene and 6.15 g (62.5 mmol, 5 eq) CrO_3 were added. The reaction mixture was allowed to warm to RT overnight. As full conversion was indicated by GC-MS the reaction was diluted with EtOAc (60 mL) and quenched by the addition of $NaHCO_3$ sat. (60 mL). After phase separation, the aqueous layer was extracted with EtOAc (3x100 mL). The combined organic layers were washed with H_2O (15 mL) and brine (15 mL). Then the organic layers were dried over Na_2SO_4 and the solvent was removed under reduced pressure. The obtained crude product was purified via column chromatography [450 mL silica, CH/EtOAc 4/1, fraction size: 80 mL].

C_8H_7NO [133.15 g/mol]; (CAS: 134837-20-0)

Yield 567 mg (5.0 mmol, 41%); colourless solid

mp 58-60 °C

GC-MS 4.49 min [133 (50%), 105 (100%), 78 (81%)]

^{1}H-NMR (300.36 MHz, $CDCl_3$): δ = 6.86 (d, $^3J_{HH}$ = 9.9 Hz, 2H, H-3, H-5), 6.38 (d, $^3J_{HH}$ = 9.9 Hz, 2H, H-2, H-6), 1.70 (s, 3H, H-7) ppm.

^{13}**C-NMR** (75.53 MHz, $CDCl_3$,): δ = 183.4 (C-1), 144.4 (C-3, C-5), 130.4 (C-2, C-6), 117.4 (C-8), 35.3 (C-4), 25.9 (C-7) ppm.

2.46 1-Methyl-4-oxocyclohex-2-enecarbonitrile (*rac*-2t)

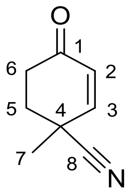

A flame dried 15 mL Schlenk flask was charged with 24 mg (0.13 mmol, 10 mol%) $Cu(OAc)_2$ and 60 mg (0.26 mmol, 20 mol%) PPh_3 and subjected to three vacuum/argon flushing cycles. Then 0.8 mL THF abs. were added and the resulting green solution was stirred for 15 min at RT. After addition of 0.31 mL (262 mg, 1.95 mmol, 1.5 eq) methyldiethoxysilane the reaction mixture turned yellow. Then a solution of 174 mg (1.3 mmol, 1 eq) **1t** in 1.4 mL THF abs. was added and the reaction was warmed to 55 °C. The reaction was stirred at 55 °C overnight, whereupon GC-MS indicated a conversion of 95%. For workup, the reaction was cooled to RT and directly applied onto a silica column [100 mL silica, CH/EtOAc 16/1 – CH/EtOAc 4/1, fraqction size: 15 mL].

C_8H_9NO [135.16]

Yield 54 mg (0.4 mmol, 31%; contains 10% **1t**); yellow oil

GC-MS 4.49 min [135 (18%), 107 (100%), 92 (4%), 68 (34%)]

^{1}**H-NMR** (300.36 MHz, $CDCl_3$): δ = 6.67 (d, $^3J_{HH}$ = 11.2 Hz, 1H, H-3), 6.08 (d, $^3J_{HH}$ = 9.9 Hz, 1H, H-2), 2.79-2.63 (m, 1H, H-6), 2.63-2.36 (m, 2H, H-5, H-6), 2.16-2.00 (m, 1H, H-5), 1.61 (s, 3H, H-7) ppm.

^{13}**C-NMR** (75.53 MHz, $CDCl_3$,): δ = 196.2 (C-1), 146.8 (C-3), 130.7 (C-2), 120.8 (C-8), 34.6 (C-5/C-6), 34.5 (C-5/C-6), 33.1 (C-4), 25.7 (C-7) ppm.

2.47 (3*S*,4*S*)-4-Methyl-4-phenyl-3-vinylcyclohexanone (4)

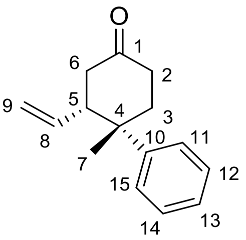

A flame dried Schlenk flask was charged with 49 mg (0.26 mmol, 1.2 eq) CuI and dried under vacuum. Then the CuI was suspended in 0.8 mL THF abs. and cooled to -78 °C using an acetone/dry ice bath. 0.52 mL (0.52 mmol, 2.4 eq) Vinylmagnesiumbromide (1M in THF) were added dropwise. The resulting mixture was stirred at -78 °C for 15 min . Then a solution of 40 mg (0.215 mmol, 1 eq) **2a** in 0.3 mL THF abs. was added dropwise. The reaction was stirred for 4 h and allowed to warm to -20 °C over this period of time. At this point full conversion was achieved as judged by GC-MS. The reaction was quenched by the addition of NH_4Cl sat. (1.5 mL) at -20 °C. The resulting mixture was stirred overnight and allowed to warm up to RT. For workup the reaction was diluted with Et_2O (5 mL) and washed with a 9/1 mixture of NH_4Cl sat./NH_4OH (25%). After phase separation the aqueous layer was extracted with Et_2O (3x5 mL). The combined organic layers were washed with the 9/1 mixture of NH_4Cl sat./NH_4OH (25%) (3x1 mL) and brine (5 mL). Then, the organic layers were dried over Na_2SO_4 and the solvent was removed under reduced pressure. Purification of the crude product was achieved via column chromatography [50 mL silica, CH/EtOAc 20/1-CH/EtOAc 14/1, fraction size: 10 mL].

$C_{15}H_{18}O$ [214.31 g/mol]

Yield 20 mg (0.1 mmol, 45%); colourless oil

GC-MS 6.07 min [214 (10%), 199 (21%), 159 (12%), 118 (100%)]

$[\alpha]_D^{24}$ 18.9 (0.475, EtOH)

HR-MS calc m/z for $C_{15}H_{18}O$ [M^+]: 214.1358, found: 214.1346

^{1}H-NMR (400 MHz, $CDCl_3$): δ = 7.46-7.42 (m, 2H, H-11, H-15), 7.39-7.33 (m, 2H, H-12, H-14), 7.27-7.21 (m, 1H, H-13), 5.67-5.56 (m, 1H, H-8), 5.01 (dt, $^3J_{HH}$ = 10.6 Hz, 1.3 Hz, 1H, H-9), 4.91 (dt, $^3J_{HH}$ = 17.2 Hz, 1.4 Hz, 1H, H-9), 3.12 (q, $^3J_{HH}$ = 7.0 Hz, 1H, H-5), 2.57-2.38 (m, 4H, H-2, H-6), 2.35-2.26 (m, 1H, H-3), 1.93 (dt, $^3J_{HH}$ = 14.0 Hz, 5.8 Hz, 1H, H-3), 1.40 (s, 3H, H-7) ppm.

^{13}C-NMR (100.58 MHz, $CDCl_3$,): δ = 211.3 (C-1), 147.1 (C-10), 137.8 (C-8), 128.7 (C-12, C-14), 126.5 (C-13), 125.9 (C-11, C-15), 116.4 (C-9), 49.0 (C-5), 43.1 (C-6), 40.6 (C-4), 38.6 (C-2), 37.7 (C-3), 20.9 (C-7) ppm.

Chiral HPLC – *rac*-4 (CHIRACEL® OJ-H; Heptane/iPrOH 1/1; 0.7 mL/min):

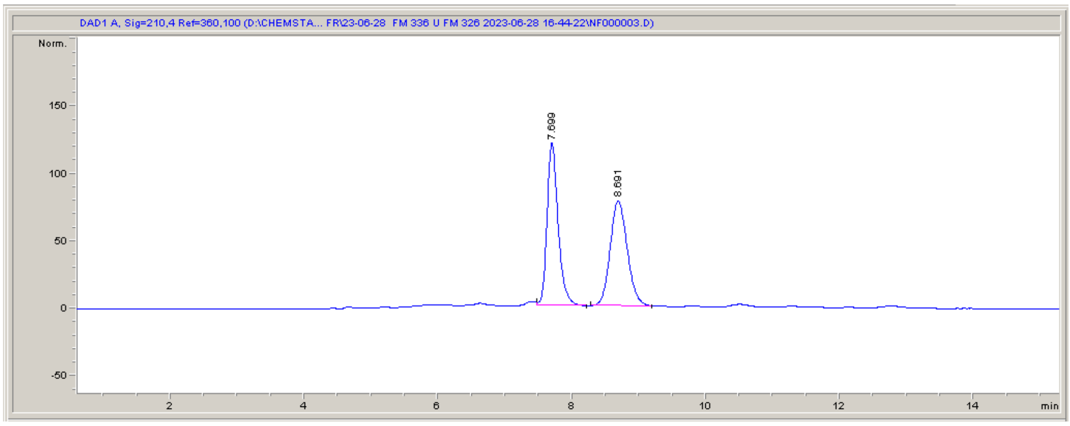

#	Time	Area	Height	Width	Area%	Symmetry
1	7.699	1395.2	120.6	0.175	50.207	0.724
2	8.691	1383.8	78	0.275	49.793	0.861

Chiral HPLC – 4 (CHIRACEL® OJ-H; Heptane/iPrOH 1/1; 0.7 mL/min):

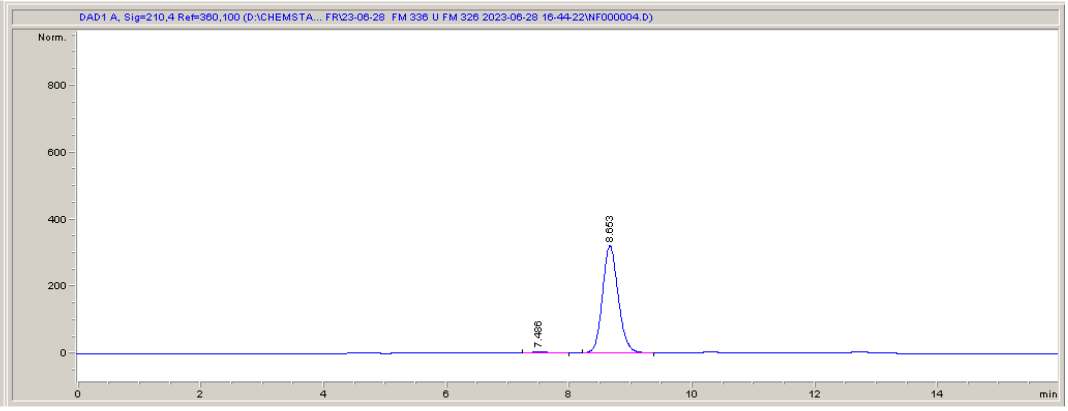

#	Time	Area	Height	Width	Area%	Symmetry
1	7.486	64	4	0.2208	1.107	0.641
2	8.653	5718.2	319.6	0.2767	98.893	0.83

2.48 (1*S*,5*S*,6*R*)-5-Methyl-5-phenylbicyclo[4.1.0]heptan-2-one (5)

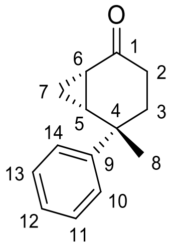

In a flame dried 10 mL Schlenk flask a suspension of 52 mg (0.3 mmol, 1.2 eq) trimethylsulfoxoniumbromide in 0.5 mL DMSO abs. was prepared. After addition of 12 mg (7.2 mg, 0.3 mmol, 1.2 eq) NaH (60% suspension), the reaction mixture was stirred for 1 h at RT. Then a solution of 46 mg (0.25 mmol, 1 eq) **2a** in 0.5 mL DMSO abs. was added dropwise. The reaction was stirred at 55 °C overnight. Full conversion was indicated by GC-MS. For workup the reaction was quenched by the addition of H_2O (3 mL) and extracted with EtOAc (3x10 mL). The combined organic layers were washed with brine (10 mL) and dried over Na_2SO_4. Removal of the solvent under reduced pressure yielded 286 mg of crude product, which was purified via column chromatography [75 mL silica, fraction size: 10 mL, CH/EtOAc 14/1 – CH/EtOAc 4/1].

**$C_{14}H_{16}O$**	[200.28 g/mol]
**Yield**	45 mg (0.22 mmol, 90%), colorless amorphous solid
**R_f**	0.33 (CH/EtOAc 4/1, CAM)
**GC-MS:**	t_R = 6.61 min [200 (19%), 145 (20%), 129 (47%), 104 (100%)]
**$[\alpha]_D^{24}$**	-37.6 (0.425, EtOH)
**HR-MS:**	calc m/z for $C_{14}H_{16}O$ [M^+]: 200.1201, found: 200.1201

^{1}H-NMR (400 MHz, $CDCl_3$): δ = 7.55-7.51 (m, 2H, H-10, H-14), 7.39-7.33 (m, 2H, H-11, H-13), 7.26 (dt, $^3J_{HH}$ = 3.7 Hz, 1.5 Hz, 1H, H-12), 2.44-2.28 (m, 2H, H-2), 2.00-1.93 (m, 1H, H-6), 1.93-1.84 (m, 2H, H-5, H-3), 1.84-1.77 (m, 1H, H-3), 1.51 (s, 3H, H-8), 1.49-1.44 (m, 1H, H-7), 1.43-1.37 (m, 1H, H-7) ppm.

^{13}C-NMR (100.58 MHz, $CDCl_3$,): δ = 208.9 (C-1), 148.9 (C-9), 128.6 (C-11, C-13), 126.5 (C-12), 125.6 (C-10, C-14), 36.0 (C-4), 33.4 (C-2), 30.8 (C-3), 29.4 (C-5), 27.1 (C-8), 26.6 (C-6), 12.2 (C-7) ppm.

Chiral HPLC – *rac*-5 (CHIRACEL® OJ-H; Heptane/iPrOH 1/1; 0.7 mL/min):

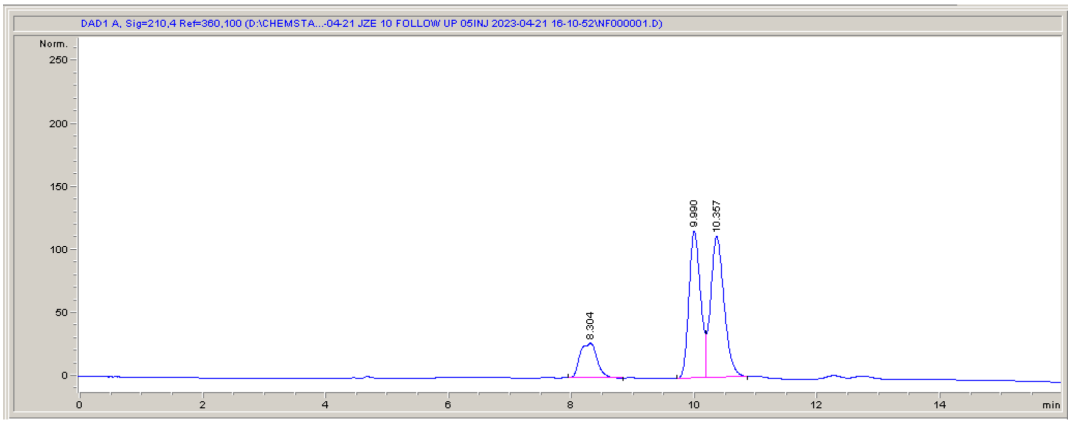

#	Time	Area	Height	Width	Area%	Symmetry
1	8.304	544.4	27.8	0.2645	14.362	1.47
2	9.99	1540.9	116.7	0.2021	40.652	0.825
3	10.357	1705.2	111.8	0.2299	44.986	0.785

Chiral HPLC – 5 (CHIRACEL® OJ-H; Heptane/iPrOH 1/1; 0.7 mL/min):

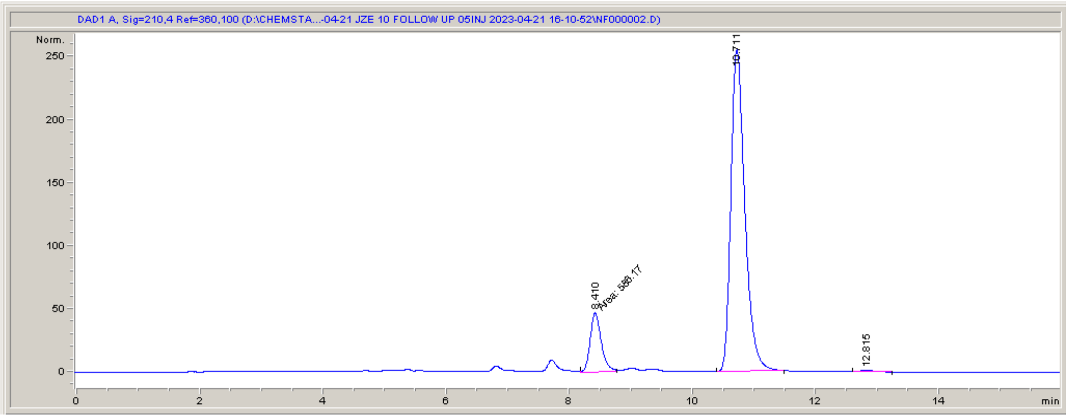

#	Time	Area	Height	Width	Area%	Symmetry
1	8.41	586.2	46.9	0.2082	12.605	0.767
2	10.711	4043.4	255.3	0.2428	86.950	0.672
3	12.815	20.7	1.1	0.2817	0.445	0.762

2.49 (1*S*)-1,4-Dimethyl-1,2,3,4-tetrahydro-[1,1'-biphenyl]-4-ol (6)

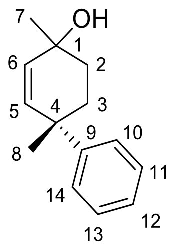

In a flame dried 15 mL Schlenk flask a solution of 49 mg (0.26 mmol, 1 eq) **138** in 0.9 mL THF abs. was prepared. The reaction mixture was cooled to -10 °C using a NaCl/ice bath. Then, 0.11 mL (0.34 mmol, 1.3 eq) MeMgBr (3M in THF) were added dropwise. The reaction was allowed

to slowly warm up to RT and was stirred overnight. Full conversion was indicated by GC-MS. For workup, the reaction was quenched by the addition of NH_4Cl sat. (1 mL). After the addition of EtOAc (10 mL) and H_2O (5 mL) the phases were separated. The aqueous layer was extracted with EtOAc (3x5 mL). The combined organic layers were dried over Na_2SO_4. Removal of the solvent under reduced pressure yielded 117 mg crude product, which was purified via column chromatography [50 mL silica, CH/EtOAc 8/1- CH/EtOAc 6/1, fraction size: 10 mL].

**$C_{14}H_{18}O$**	[202.29 g/mol]
**Yield**	45 mg (0.22 mmol, 86%), colourless solid
**R_f**	0.36 (CH/EtOAc 4/1, CAM)
**GC-MS**	5.88 min [202 (17%), 187 (98%), 169 (100%), 144 (30%), 129 (50%)]
**HR-MS**	calc m/z for $C_{14}H_{18}O$ [M^+]: 202.1358, found: 202.1347
**$[\alpha]_D^{24}$**	-73 (0.575, EtOH)

^{1}H-NMR-Diastereomer 1 (400 MHz, $CDCl_3$): δ = 7.39-7.35 (m, 2H, H-10, H-14), 7.32-7.29 (m, 2H, H-11, H-13), 7.23-7.18 (m, 1H, H-12), 5.83-5.71 (m, 2H, H-5, H-6), 1.91-1.86 (m, 1H, H-3), 1.79-1.71 (m, 1H, H-3), 1.68-1.60 (m, 1H, H-2), 1.54-1.45 (m, 1H, H-2), 1.39 (s, 3H, H-7), 1.35 (s, 3H, H-8) ppm.

^{1}H-NMR-Diastereomer 2 (400 MHz, $CDCl_3$): δ = 7.34-7.32 (m, 2H, H-10, H-14), 7.32-7.29 (m, 2H, H-11, H-13), 7.23-7.18 (m, 1H, H-12), 5.83-5.71 (m, 2H, H-5, H-6), 2.03-1.95 (m, 1H, H-3), 1.79-1.71 (m, 1H, H-3), 1.68-1.60 (m, 1H, H-2), 1.54-1.45 (m, 1H, H-2), 1.43 (s, 3H, H-7), 1.30 (s, 3H, H-8) ppm.

^{13}C-NMR-Diastereomer 1 (100.58 MHz, $CDCl_3$,): δ = 148.7 (C-9), 136.6 (C-6), 133.3 (C-5), 128.3 (C-11, C-13), 126.4 (C-10, C-14), 126.1 (C-12), 68.5 (C-1), 39.8 (C-4), 36.6 (C-3), 35.1 (C-2), 29.0 (C-8), 27.9 (C-7) ppm.

^{13}C-NMR-Diastereomer 2 (100.58 MHz, $CDCl_3$,): δ = 147.8 (C-9), 136.8 (C-6), 133.2 (C-5), 128.3 (C-11, C-13), 126.5 (C-10, C-14), 126.1 (C-12), 67.9 (C-1), 40.0 (C-4), 35.8 (C-3), 34.7 (C-2), 29.4 (C-7/C-8), 29.3 (C-7/C-8) ppm.

Chiral HPLC – *rac*-6 (CHIRACEL® OJ-H; Heptane/i-PrOH 1/1; 0.7 mL/min):

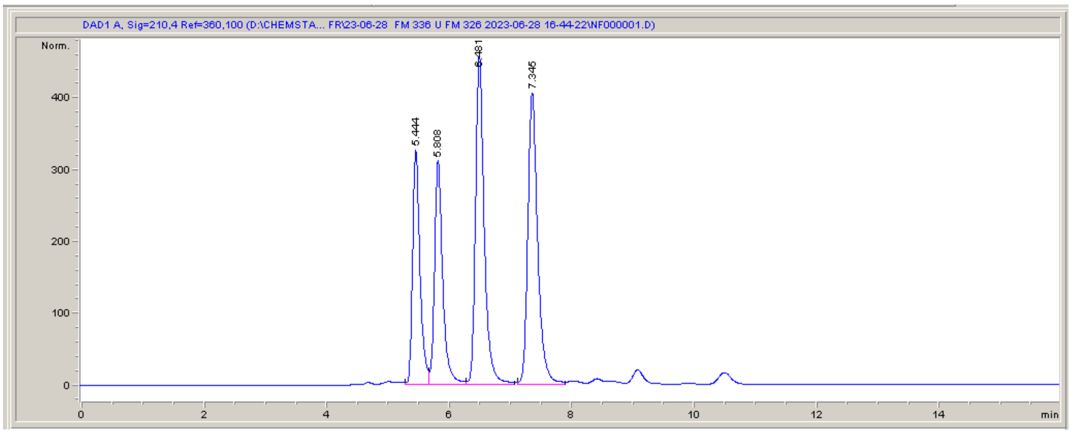

#	Time	Area	Height	Width	Area%	Symmetry
1	5.444	2719.6	327.4	0.1249	18.416	0.687
2	5.808	2890.1	312	0.1381	19.570	0.671
3	6.481	4576.7	458.4	0.1505	30.991	0.694
4	7.345	4581.2	406.3	0.1716	31.022	0.714

Chiral HPLC – 6 (CHIRACEL® OJ-H; Heptane/i-PrOH 1/1; 0.7 mL/min):

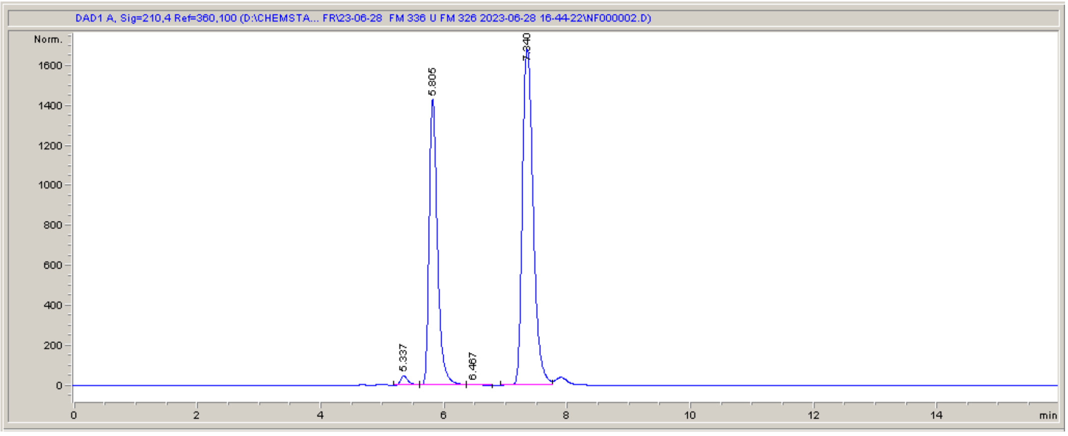

#	Time	Area	Height	Width	Area%	Symmetry
1	5.337	448.4	49.9	0.1309	1.289	0.621
2	5.805	13434.8	1431.9	0.1415	38.629	0.661
3	6.467	100.3	7.1	0.1996	0.288	0.586
4	7.34	20795.4	1681.7	0.1943	59.793	0.717

2.50 (1*S*,4*R*)-1-Methyl-1,2,3,4-tetrahydro-[1,1'-biphenyl]-4-ol (7)

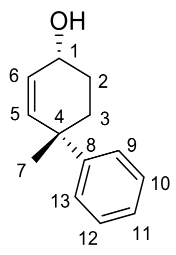

In a flame dried Schlenk flask a solution of 78 mg (0.42 mmol, 1 eq) **2a** in 4.2 mL THF abs. was prepared. The solution was cooled to -78 °C using an acetone/dry ice bath. To this solution 0.76 mL (0.76 mmol, 1.8 eq) L-Selectride (1M in THF) were added dropwise. The reaction was stirred for 1 h at -78 °C. Full conversion was indicated by GC-MS. The mixture was quenched by the addition of NH_4Cl sat. (5 mL) at low temperature. After warming to RT the phases were separated and the aqueous layer was extracted with EtOAc (4x10 mL). The combined organic layers were washed with brine (5 mL) and dried over Na_2SO_4. Removal of the solvent under reduced pressure yielded 300 mg crude product, which was purified via column chromatography [50 mL silica, CH/EtOAc 10/1-CH/EtOAc 6/1, fraction size: 10 mL].

**$C_{13}H_{16}O$**	[188.27 g/mol]
**Yield**	53 mg (0.28 mmol, 67%), colourless oil
**GC-MS**	5.84 min [188 (23%), 170 (19%), 155 (55%), 145 (32%), 129 (58%), 118 (100%), 91 (55%)]
**$[\alpha]_D^{24}$**	-22 (0.455, EtOH)
**HRMS**	calc m/z for $C_{13}H_{16}O$ [M^+]: 188.1201, found: 188.1189

^{1}H-NMR (300.36 MHz, $CDCl_3$): δ = 7.38 (d, $^3J_{HH}$ = 7.4 Hz, 2H, H-9, H-13), 7.31 (t, $^3J_{HH}$ = 7.5 Hz, 2H, H-10, H-12), 7.20 (t, $^3J_{HH}$ = 7.0 Hz, 1H, H-11), 5.95-5.75 (m, 2H, H-5, H-6), 4.26 (bs, 1H, H-1), 2.06-1.94 (m, 1H, H-3), 1.93-1.78 (m, 1H, H-2), 1.77-1.63 (m, 1H, H-3), 1.52-1.42 (m, 1H, H-2), 1.39 (s, 3H, H-7) ppm.

^{13}C-NMR (75.53 MHz, $CDCl_3$,): δ = 148.4 (C-8), 137.8 (C-5), 129.8 (C-6), 128.3 (C-10, C-12), 126.7 (C-9, C-13), 126.1 (C-11), 66.7 (C-1), 39.8 (C-4), 36.3 (C-3), 29.1 (C-2), 29.0 (C-7) ppm.

Chiral HPLC – *rac*-7 (CHIRACEL® OJ-H; Heptane/EtOH 4/1; 0.7 mL/min):

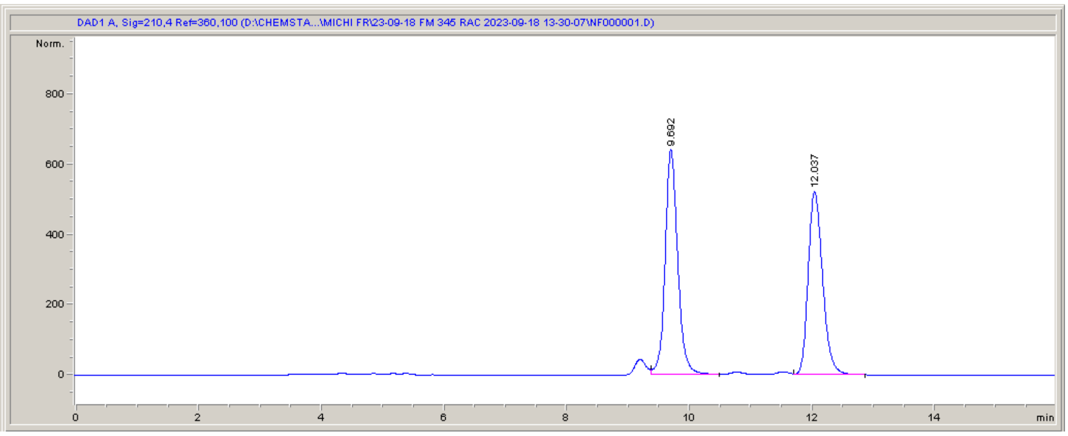

#	Time	Area	Height	Width	Area%	Symmetry
1	9.692	9175	641.1	0.217	51.802	0.801
2	12.037	8536.7	521.7	0.2529	48.198	0.758

Chiral HPLC – 7 (CHIRACEL® OJ-H; Heptane/EtOH 4/1; 0.7 mL/min):

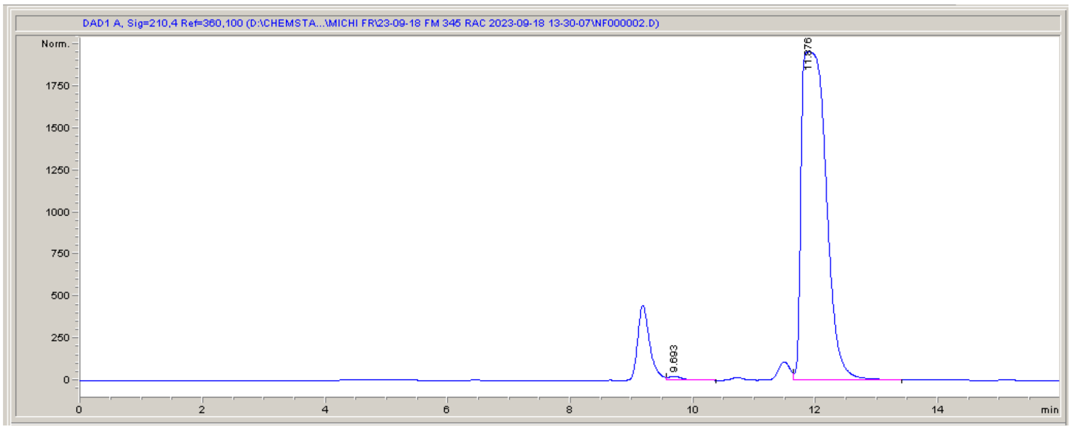

#	Time	Area	Height	Width	Area%	Symmetry
1	9.693	431.3	24.7	0.248	0.782	0.504
2	11.876	54681.9	1951.9	0.3708	99.218	0.358

3 Biological Section

3.1 Expression of OPR3 and YqjM

pET21d-*opr3* and pET21a-*yqjM* plasmids were transformed into *E.coli* Top10 cells for selection. Plasmid DNA from three colonies, each, was isolated and sequenced. Plasmids exhibiting the desired gene were transformed into *E.coli* expression strain BL21-CodonPlus^{TM}-(DE3)-RIL. These strains were then used for generation of appropriate overnight cell cultures. 10 mL of an overnight cell culture was used to inoculate one flask with 800 mL LB medium supplemented with ampicillin (100 µg/mL) and chloramphenicol (20 µg/mL). The cell culture was incubated at 37 °C at 150 rpm until an OD600 of 0.6-0.8 was reached. Then, protein expression was induced by the addition of IPTG (to a final concentration of 0.125 mM) and the cultures were incubated at 20 °C and 150 rpm for 16 h. The cells were harvested by centrifugation at 5000 rpm and 4 °C (for 15 min) and the resulting pellets were stored at -32 °C.

3.2 DNA sequences and translated amino acid sequences of ene-reductases

The gene of OPR3 wild type (*Lycopersiconesculentum*, tomato) with the N-terminal 6x His-tag underlined:

ATGGCTAGC<u>CACCACCACCACCACCAC</u>ATGGCGTCTTCAGCTCAAGATGGAAACA
ATCCCCTTTTCTCTCCTTACAAGATGGGCAAGTTCAATCTATCCCACAGGGTAGTA
TTGGCTCCGATGACAAGGTGCAGAGCACTGAATAATATTCCACAGGCGGCGCTAG
GGGAGTATTACGAGCAGAGAGCGACGGCCGGTGGATTTCTGATCACTGAAGGCA
CTATGATTTCTCCGACTTCAGCTGGGTTTCCTCATGTGCCAGGGATTTTCACAAAG
GAACAAGTAAGGGAATGGAAGAAAATAGTTGATGTAGTGCATGCAAAGGGTGCT
GTCATATTTTGTCAGCTGTGGCATGTTGGTCGTGCATCTCATGAAGTGTATCAACC
TGCTGGAGCTGCACCAATATCATCCACTGAGAAGCCTATATCAAATAGGTGGAGA
ATTCTAATGCCTGATGGAACTCATGGGATTTATCCAAAACCAAGAGCAATTGGAA
CCTATGAGATCTCACAAGTTGTTGAAGATTATCGCAGGTCGGCCTTGAATGCTAT
TGAAGCAGGTTTCGATGGTATTGAAATCCATGGAGCTCACGGTTACTTGATTGAT
CAATTCTTGAAAGATGGGATCAATGACCGGACAGATGAGTATGGTGGATCACTA
GCCAACCGGTGCAAATTCATCACACAGGTGGTTCAAGCAGTAGTCTCAGCAATAG
GAGCTGATCGCGTAGGCGTTAGAGTTTCACCAGCAATAGATCATCTTGATGCCAT
GGACTCTAATCCACTCAGCCTTGGCTTAGCAGTTGTTGAAAGACTAAACAAAATC
CAACTCCATTCTGGTTCCAAGCTTGCCTATCTTCATGTAACACAGCCACGATACGT

AGCATATGGGCAAACTGAAGCAGGCAGACTTGGCAGTGAAGAGGAAGAGGCTCG
TTTAATGAGGACTTTGAGGAACGCGTATCAGGGGACATTCATTTGCAGTGGTGGA
TACACTAGGGAACTAGGAATTGAGGCTGTGGCACAAGGTGATGCTGATCTCGTGT
CATATGGTCGTCTTTTCATCTCTAATCCTGATTTGGTTATGAGAATCAAGCTAAAT
GCACCTCTAAATAAGTATAACAGGAAGACATTCTATACTCAAGATCCAGTTGTGG
GATACACAGATTACCCTTTCCTTCAAGGAAATGGAAGCAATGGACCGTTATCGCG
TCTGTGA

MASHHHHHHMASSAQDGNNPLFSPYKMGKFNLSHRVVLAPMTRCRALNNIPQAAL
GEYYEQRATAGGFLITEGTMISPTSAGFPHVPGIFTKEQVREWKKIVDVVHAKGAVIF
CQLWHVGRASHEVYQPAGAAPISSTEKPISNRWRILMPDGTHGIYPKPRAIGTYEISQ
VVEDYRRSALNAIEAGFDGIEIHGAHGYLIDQFLKDGINDRTDEYGGSLANRCKFITQ
VVQAVVSAIGADRVGVRVSPAIDHLDAMDSNPLSLGLAVVERLNKIQLHSGSKLAYL
HVTQPRYVAYGQTEAGRLGSEEEEARLMRTLRNAYQGTFICSGGYTRELGIEAVAQG
DADLVSYGRLFISNPDLVMRIKLNAPLNKYNRKTFYTQDPVVGYTDYPFLQGNGSNG
PLSR

The gene of YqjM wild type (*Bacillus subtilis*):

ATGGCCAGAAAATTATTTACACCTATTACAATTAAAGATATGACGTTAAAAAACC
GCATTGTCATGTCGCCAATGTGCATGTATTCTTCTCATGAAAAGGACGGAAAATT
AACACCGTTCCACATGGCACATTACATATCGCGCGCAATCGGCCAGGTCGGACTG
ATTATTGTAGAGGCGTCAGCGGTTAACCCTCAAGGACGAATCACTGACCAAGACT
TAGGCATTTGGAGCGACGAGCATATTGAAGGCTTTGCAAAACTGACTGAGCAGGT
CAAAGAACAAGGTTCAAAAATCGGCATTCAGCTTGCCCATGCCGGACGTAAAGC
TGAGCTTGAAGGAGATATCTTCGCTCCATCGGCGATTGCGTTTGACGAACAATCA
GCAACACCTGTAGAAATGTCAGCAGAAAAAGTAAAAGAAACGGTCCAGGAGTTC
AAGCAAGCGGCTGCCCGCGCAAAAGAAGCCGGCTTTGATGTGATTGAAATTCAT
GCGGCGCACGGATATTTAATTCATGAATTTTTGTCTCCGCTTTCCAACCATCGAAC
AGATGAATATGGCGGCTCACCTGAAAACCGCTATCGTTTCTTGAGAGAGATCATT
GATGAAGTCAAACAAGTATGGGACGGTCCTTTATTTGTCCGTGTATCTGCTTCTGA
CTACACTGATAAAGGCTTAGACATTGCCGATCACATCGGTTTTGCAAAATGGATG
AAGGAGCAGGGTGTTGACTTAATTGACTGCAGCTCAGGCGCCCTTGTTCACGCAG
ACATTAACGTATTCCCTGGCTATCAGGTCAGCTTCGCTGAGAAAATCCGTGAACA
GGCGGACATGGCTACTGGTGCCGTCGGCATGATTACAGACGGTTCAATGGCTGAA

GAAATTCTGCAAAACGGACGTGCCGACCTCATCTTTATCGGCAGAGAGCTTTTGC
GGGATCCATTTTTTGCAAGAACTGCTGCGAAACAGCTCAATACAGAGATTCCGGC
CCCTGTTCAATACGAAAGAGGCTGGTAA

MARKLFTPITIKDMTLKNRIVMSPMCMYSSHEKDGKLTPFHMAHYISRAIGQVGLIIV
EASAVNPQGRITDQDLGIWSDEHIEGFAKLTEQVKEQGSKIGIQLAHAGRKAELEGDI
FAPSAIAFDEQSATPVEMSAEKVKETVQEFKQAAARAKEAGFDVIEIHAAHGYLIHEF
LSPLSNHRTDEYGGSPENRYRFLREIIDEVKQVWDGPLFVRVSASDYTDKGLDIADHI
GFAKWMKEQGVDLIDCSSGALVHADINVFPGYQVSFAEKIREQADMATGAVGMITD
GSMAEEILQNGRADLIFIGRELLRDPFFARTAAKQLNTEIPAPVQYERGW

3.3 Preparation of buffers

NaH_2PO_4/Na_2HPO_4 buffer (50 mM, pH 7.5, 150 mM NaCl)

$Na_2HPO_4 \cdot 2H_2O$ (8.29 g, 46.6 mmol), $NaH_2PO_4 \cdot H_2O$ (0.47 g, 3.4 mmol) and NaCl (8.77 g, 150 mmol) were dissolved in 1 L H_2O bidest. Under vigorous stirring H_3PO_4 conc. or NaOH-solution (conc., in H_2O) were added until a calibrated pH-meter indicated pH 7.5. Buffer solution was pressed through a syringe filter.

For buffers additionally containing imidazole, the latter was added prior to the adjustment of the pH.

Buffer A: Tris-HCl buffer (50 mM, pH 7.5)

Tris-HCl (7.88 g, 50 mmol) was dissolved in 1 L H_2O bidest. Under vigorous stirring HCl conc. or NaOH-solution (conc., in H_2O) were added until a calibrated pH-meter indicated pH 7.5. Buffer solution was pressed through a syringe filter.

Buffer B: Tris-HCl buffer (50 mM, pH 7.5, 400 mM KCl)

Tris-HCl (7.88 g, 50 mmol) and KCl (29.8 g, 400 mmol) were dissolved in 1 L H_2O bidest. Under vigorous stirring HCl conc. or NaOH-solution (conc., in H_2O) were added until a calibrated pH-meter indicated pH 7.5. Buffer solution was pressed through a syringe filter.

Buffer C: Tris-HCl buffer (50 mM, pH 7.5, 1.5 M $(NH_4)_2SO_4$)

Tris-HCl (7.88 g, 50 mmol) and $(NH_4)_2SO_4$ (198.2 g, 1.5 mol) were dissolved in 1 L H_2O bidest. Under vigorous stirring HCl conc. or NaOH-solution (conc., in H_2O) were added until a calibrated pH-meter indicated pH 7.5. Buffer solution was pressed through a syringe filter.

3.4 Preparation of YqjM crude cell lysate

For the preparation of the YqjM crude cell lysate purpose harvested cells (Section 3.1) were resuspended in sodium phosphate buffer (50 mM, pH 7.5, 150 mM NaCl). 5 mL buffer were used per g wet cell paste and a spatula tip of FMN was added to the suspension. The suspension was left on ice for 20 min and then cells were lysed by sonication with a Labsonic L sonication probe (B. Braun Biotech, Berlin, Germany) for 10 min (0.5 s pulse, 60% power). The cell lysate was separated from the debris and insoluble fraction by centrifugation at 38500 g for 45 min at 4 °C. The cleared cell lysate was then used for the biotransformations.

3.5 Purification of OPR3

A cell pellet (~ 12 g) (Section 3.1) was resuspended in lysis buffer (50 mM sodium phosphate buffer, pH 7.5, 20 mM imidazole, 150 mM NaCl, 3 mL/g cell pellet), to which phenylmethylsulfonyl fluoride (final concentration 1.3 mM) and lysozyme (final concentration 1 mg/mL) were added. After 20 min of incubation at 4 °C the cells were further lysed by ultra-sonication for 10 min (0.5 s pulse, power 60 %). The sonicated suspension was subsequently centrifuged at 20.000 rpm and 4 °C for 45 min. The supernatant was then loaded onto a Ni-NTA HisTrap FF 5 mL column equilibrated with lysis buffer. After washing with increasing concentrations of imidazole (up to 50 mM), the bound protein was eluted with elution buffer (50 mM sodium phosphate buffer, pH 7.5, 300 mM imidazole, 150 mM NaCl). The yellow fractions containing OPR3 were pooled, concentrated, and dialyzed against sodium phosphate buffer (50 mM, pH 7.5, 150 mM NaCl) at 4 °C overnight. If required, the protein was then again concentrated and aliquots of OPR3 WT were stored at -32 °C until needed. In addition, the purity of the protein fractions was analyzed by SDS-PAGE.

3.6 Purification of YqjM

A cell pellet (10-15 g/ Section 3.1) was resuspended in buffer A (50 mM Tris-HCl, pH 7.5, 4 mL/g cell pellet), to which phenylmethylsulfonyl fluoride (final concentration 1.3 mM) and lysozyme (final concentration 1 mg/mL) were added. After 20 min of incubation at 4 °C the cells were further lysed by ultra-sonication for 10 min (0.5 s pulse, power 60 %). The sonicated suspension was centrifuged at 20.000 rpm and 4 °C for 45 min. The supernatant was then loaded onto an ion exchange chromatography DEAE-Sephacel FF column (20 mL) equilibrated with buffer A. After extensive washing with buffer A, the bound protein was eluted with a linear gradient of 250 mL of buffer A and buffer B (buffer B: 50 mM Tris-HCl, pH 7.5, 400 mM KCl). The yellow fractions containing YqjM were pooled, concentrated, and dialyzed against buffer A at 4 °C overnight. The dialysate was brought to 30% ammonium sulfate saturation and applied to a phenyl-sepharose column (25 mL) freshly equilibrated with buffer C (50 mM Tris-HCl, pH 7.5, 1.5M $(NH_4)_2SO_4$). After washing with buffer C, the bound protein was eluted with a linear gradient of 500 mL buffer C and buffer A. The yellow fractions containing YqjM were again pooled, concentrated, and dialyzed against buffer A at 4 °C overnight. If necessary, the protein solution was again concentrated, before flash freezing aliquots of YqjM with liquid nitrogen and storing them at – 32 °C until further use. To prove the quality of the protein purification SDS-PAGE analysis was applied after each chromatographic step.

3.7 Estimation of enzyme concentrations based on absorption spectra

Enzyme concentrations were photometrically estimated using the molar extinction coefficients of OPR3 and YqjM at their respective absorption maximum (OPR3: 466 nm, YqjM: 455 nm; $\varepsilon_{\lambda max}$ = ~11600 $M^{-1} \cdot cm^{-1}$).[10,11]

All spectra were recorded using a spectrophotometer Specord 200 plus from Analytik Jena. The measurements were carried out in disposable cuvettes (semimicro, dimension 12.5×12.5×45 mm) from Brand.

4 Biocatalytic Section

4.1 Analytical scale biotransformations

All biotransformations of analytical scale were performed in triplicates. Each reaction was carried out in a total volume of 600 µL. Substrate concentration was kept at 10 mM, while 1.5 eq NADH were utilized as stoichiometric reducing agent. To exclude that the limited substrate solubility influences the observed results, the biotransformations were prepared without the use of a mastermix. Setting up biotransformations started with the preparation of a substrate stock solution in DMSO (111.1mM). A 54 µL aliquot of this stock solution was placed in a 1.5 mL PP Eppendorf vial. Then NaPi buffer (50mM NaPi, 150mM NaCl, pH = 7.5; volume of buffer chosen in a way that a final reaction volume of 600 µL was reached) was added. Enzyme was added to the resulting white emulsion. The volume of the added enzyme was chosen in a way that ensured a final ene-reductase concentration of 5 µM. Finally, a 200mM NADH solution in NaPi buffer (45 µL) was added. After careful vortexing, the reactions were incubated in a thermoshaker (300 rpm, 30 °C, 18 h). For workup 100 µL of the reaction mixture were mixed with a solution of TBB (1,3,5-tribromobenzene) in NaPi (500 µL). The resulting samples were then injected into a HPLC-MS system to determine educt as well as product concentrations. The reported results represent the average of a triplicate series of experiments. For quantification external calibrations with TBB as external standard were prepared.

For determination of the stereoselectivity the biotransformations were extracted with EtOAc (2 x 600 µL). The organic layer was dried over Na_2SO_4, followed by solvent removal under reduced pressure. The remainings were taken up in HPLC grade heptane (200 µL). The resulting samples were measured *via* chiral HPLC (CHIRACEL OJ-H column, detection at 210 nm). Enantiomeric purities were derived from the corresponding peak areas. In more complex cases (substrates that gave low conversion), up to five different measurements (e.g. spike experiments) were carried out in order to ensure unambiguous determination of enantioselectivities.

4.2 Preparative scale biotransformations

4.2.1 (*S*)-1-Methyl-2,3-dihydro-[1,1'-biphenyl]-4(1*H*)-one (2a)

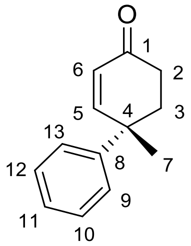

For the large scale biotransformation 300 mg (1.58 mmol) substrate **1a** were used. The biotransformation was distributed over 8 Falcon tubes in equal amounts. Each Falcon tube contained 20 mL of the reaction mixture. To set up the biotransformations a solution of 300 mg (1.58 mmol, 1 eq) **1a** in 14.4 mL DMSO was prepared. Each Falcon tube was charged with 1.8 mL of this stock solution. Then 16.5 mL NaPi buffer, 0.35 mL YqjM wt (726 µM in NaPi) and 1.2 mL of a 200 mM NADH solution in buffer were added to each vial. The biotransformations were incubated in a thermoshaker (24 h, 64 rpm, 27 °C). At this point GC-MS control showed 80% conversion. After addition of further 4 mM NADH and 3 µM enzyme, the biotransformations were incubated for further 24 h, whereafter full conversion was achieved. For workup the biotransformations were pooled together and extracted with EtOAc (3x40 mL). The combined organic layers were washed with brine (15 mL) and dried over Na_2SO_4. The solvent was removed under reduced pressure yielding 800 mg crude product. Purification was achieved via column chromatography [175 mL silica, fraction size: 20 mL, CH/EtOAc 16/1 – CH/EtOAc 6/1].

$C_{13}H_{14}O$ [186.25 g/mol]

Yield 214 mg (1.15 mmol, 72%), colourless oil

$[\alpha]_D^{24}$ -94 (0.5, EtOH)

^{1}H-NMR (300.36 MHz, $CDCl_3$) δ = 7.43-7.17 (m, 5H, H-9 – H-13), 6.95 (d, $^3J_{HH}$ = 10.1 Hz, 1H, H-5), 6.13 (d, $^3J_{HH}$ = 10.2 Hz, 1H, H-6), 2.48-2.08 (m, 4H, H-2, H-3), 1.57 (s, 3H, H-7) ppm.

^{13}C-NMR (75.53 MHz, $CDCl_3$) δ = 199.6 (C-1), 157.2 (C-5), 145.4 (C-8), 128.8 (C-10, C-12), 128.8 (C-6), 126.7 (C-11), 126.2 (C-9, C-13), 40.7 (C-4), 38.3 (C-3), 34.8 (C-2), 27.7 (C-7) ppm.

4.2.2 (*S*)-4'-Fluoro-1-methyl-2,3-dihydro-[1,1'-biphenyl]-4(1*H*)-one (2b)

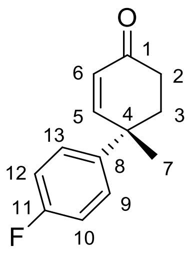

In a Falcon tube a solution of 50 mg (0.25 mmol, 1 eq) **1b** in 2.25 mL DMSO was prepared. After addition of 22.5 mL NaPi a white emulsion was formed, which was intensively vortexed. Then 265 µL (corresponds to 0.02 mol% final concentration) YqjM wt (188 µM in NaPi) and 195 mg (0.275 mmol, 1.1 eq) NADH were added. The reaction was incubated in a thermoshaker (64 rpm, 25 °C) overnight. As reaction control of GC-MS indicated no full conversion, further portions of NADH (2x0.4 eq) were added consecutively. After a total reaction time of 48 h a conversion of 83% was achieved and workup was initiated. For workup the reaction mixture was extracted with EtOAc (3x20 mL) and the combined organic layers were washed with brine (20 mL). The combined organic layers were dried over Na_2SO_4, followed by removal of the solvent under reduced pressure. The obtained crude product (80 mg) was purified via column chromatography [20 mL silica, CH/EtOAc 20/1 – CH/EtOAc 8/1, fraction size: 5 mL].

$C_{13}H_{13}FO$ [204.24 g/mol]

Yield 22 mg (0.11 mmol, 43%), colourless oil

GC-MS 6.06 min [204 (48%), 189 (26%), 162 (72%), 147 (100%)]

$[\alpha]_D^{24}$ -78.8 (0.495, EtOH)

^{1}H-NMR (300.36 MHz, $CDCl_3$): δ = 7.35-7.22 (m, 2H, H-9, H-13), 7.03 (t, $^3J_{HH}$ = 8.6 Hz, 2H, H-10, H-12), 6.90 (d, $^3J_{HH}$ = 10.2 Hz, 1H, H-5), 6.12 (d, $^3J_{HH}$ = 10.2 Hz, 1H, H-6), 2.48-2.34 (m, 1H, H-2), 2.33-2.23 (m, 1H, H-2), 2.23-2.06 (m, 2H, H-3), 1.55 (s, 3H, H-7) ppm.

^{13}C-NMR (75.53 MHz, $CDCl_3$,): δ = 199.3 (C-1), 161.7 (d, $^1J_{CF}$ = 245.82 Hz, C-11), 156.8 (C-5), 141.1 (d, $^4J_{CF}$ = 3.2 Hz, C-8), 128.8 (C-6), 127.9 (d, $^3J_{CF}$ = 7.9 Hz, C-9, C-13), 115.5 (d, $^2J_{CF}$ = 21.2 Hz, C-10, C-12), 40.3 (C-4), 38.4 (C-3), 34.7 (C-2), 27.9 (C-7) ppm.

4.2.3 (*S*)-4’-Chloro-1-methyl-2,3-dihydro-[1,1’-biphenyl]-4(1*H*)-one (2c)

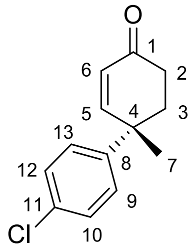

In a Falcon tube a solution of 44 mg (0.2 mmol, 1 eq) **1c** in 2.25 mL DMSO was prepared. After addition of 17.8 mL NaPi a white emulsion was formed, which was intensively vortexed. Then 319 µL (corresponds to 0.03 mol% final concentration) YqjM wt (188 µM in NaPi) and 156 mg (0.22 mmol, 1.1 eq) NADH were added. The reaction was incubated in a thermoshaker (64 rpm, 25 °C) overnight. At this point a conversion of 50% was achieved as judged by GC-MS. Thus, further 0.03 mol% YqjM wt and 1.1 eq of NADH were added. The biotransformation was again incubated overnight. For workup the reaction mixture was extracted with EtOAc (3x20 mL). The combined organic layers were dried over Na_2SO_4, followed by removal of the solvent under reduced pressure. The obtained crude product (78 mg) was purified via column chromatography [25 mL silica, CH/EtOAc 12/1 – CH/EtOAc 10/1, fraction size: 5 mL].

$C_{13}H_{13}ClO$ [220.70 g/mol]

Yield 18 mg (0.08 mmol, 41%), yellowish oil

GC-MS 6.61 min [220 (50%), 192 (50%), 163 (65%), 143 (100%), 128 (58%)]

$[\alpha]_D^{24}$ -103 (0.485, EtOH)

^{1}H-NMR (300.36 MHz, $CDCl_3$): δ = 7.37-7.19 (m, 4H, H-9, H-10, H-12, H-13), 6.89 (d, $^3J_{HH}$ = 10.2 Hz, 1H, H-5), 6.13 (d, $^3J_{HH}$ = 10.2 Hz, 1H, H-6), 2.47-2.34 (m, 1H, H-2), 2.32-2.23 (m, 1H, H-2), 2.22-2.06 (m, 2H, H-3), 1.54 (s, 3H, H-7) ppm.

^{13}C-NMR (75.53 MHz, $CDCl_3$,): δ = 199.2 (C-1), 156.4 (C-5), 144.0 (C-8), 132.9 (C-11), 129.0 (C-6), 127.8 (C-9, C-13/ C-10, C-12), 40.4 (C-4), 38.2 (C-3), 34.6 (C-2), 27.7 (C-7) ppm.

4.2.4 (*S*)-4'-Bromo-1-methyl-2,3-dihydro-[1,1'-biphenyl]-4(1*H*)-one (2d)

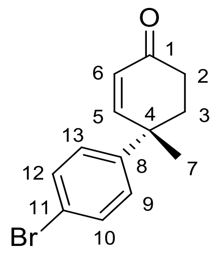

For the large scale biotransformation 79 mg (0.3 mmol, 1 eq) **1d** were used. The biotransformation was distributed over 2 Falcon tubes in equal amounts. Each Falcon tube contained 15 mL of the reaction mixture. To set up the biotransformations a solution of 79 mg (0.3 mmol, 1 eq) **1d** in 2.7 mL DMSO was prepared. Each Falcon tube was charged with 1.35 mL of this stock solution. Then 12.5 mL NaPi buffer, 275 µL YqjM wt (188 µM in NaPi) and 0.9 mL of a 200 mM NADH solution in NaPi were added to each Falcon tube. The biotransformations were incubated in a thermoshaker (24 h, 64 rpm, 27 °C). At this point GC-MS control showed a 15% conversion. Through the addition of further portions of enzyme (+0.03 + 0.05 + 0.02 mol%) and NADH (+1.2 eq +1.2 eq +1.2 eq), the conversion could be driven to 35%. For workup the biotransformations were pooled together and extracted with EtOAc (3x80 mL). The combined organic layers were washed with brine (10 mL) and dried over Na_2SO_4. The solvent was removed under reduced pressure yielding 425 mg crude product. Purification was achieved via column chromatography [160 mL silica, CH/EtOAc 20/1 – CH/EtOAc 10/1, fraction size: 10 mL].

**$C_{13}H_{13}BrO$**	[265.15]
**Yield**	17 mg (0.06 mmol, 21%), colourless solid
**mp**	93-95 °C
**GC-MS**	6.96 min [264.0/266.0 (55%), 236.0/238.0 (33%), 222.0/224.0 (44%), 143.1 (95%), 128.1 (100%)]
**$[\alpha]_D^{24}$**	-99.2 (0.665; EtOH)

^{1}H-NMR (300.36 MHz, $CDCl_3$): δ = 7.47 (d, $^3J_{HH}$ = 8.5 Hz, 2H, H-10, H-12), 7.21 (d, $^3J_{HH}$ = 8.5 Hz, 2H, H-9, H-13), 6.88 (d, $^3J_{HH}$ = 10.1 Hz, 1H, H-5), 6.13 (d, $^3J_{HH}$ = 10.2 Hz, 1H, H-6), 2.48-2.34 (m, 1H, H-2), 2.33-2.23 (m, 1H, H-2), 2.23-2.06 (m, 2H, H-3), 1.54 (s, 3H, H-7) ppm.

^{13}**C-NMR** (75.53 MHz, $CDCl_3$,): δ = 199.1 (C-1), 156.3 (C-5), 144.5 (C-8), 131.8 (C-10, C-12), 129.0 (C-6), 128.1 (C-9, C-13), 120.9 (C-11), 40.5 (C-4), 38.2 (C-3), 34.6 (C-2), 27.7 (C-7) ppm.

4.2.5 (*S*)-1-Methyl-2,3-dihydro-[1,1'-biphenyl]-4(1*H*)-one (2a) – preparative scale-crude cell lysate with 1.5 eq NADH

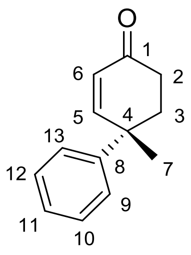

For the large scale biotransformation of **1a** using crude cell lysate of YqjM wt 24 biotransformations were set up in parallel. 35 mg (0.19 mmol, 1 eq) **1a** were used as substrate and distributed between the 24 individual biotransformations. For setting up the individual biotransformations a stock solution of **1a** (1.47 mg **1a** dissolved in 72 µL DMSO) were placed in an Eppendorf tube. Then 401 µL NaPi (50 mM, 150 mM NaCl, pH = 7.5), 267 µL crude cell lysate (1 g cell pellet/5 mL NaPi) and 60 µL of a NADH stock solution (200 mM in NaPi) were added. After gentle vortexing the resulting biotransformations were incubated (450 rpm, 30 °C, 18 h). After full conversion was indicated by GC-MS the individual biotransformations were pooled together and extracted with EtOAc (3 x 25 mL). The combined organic layers were washed with brine (5 mL) and dried over Na_2SO_4. The solvent was removed under reduced pressure. Purification was achieved via column chromatography [50 mL silica, CH/EtOAc 18/1 – CH/EtOAc 8/1, fraction size: 10 mL].

$C_{13}H_{14}O$ [186.25 g/mol]

Yield 23 mg (0.12 mmol, 63%; contains 15% cyclohexanone), colourless oil

$[\alpha]_D^{24}$ -87 (0.45, EtOH)

^{1}**H-NMR** (300.36 MHz, $CDCl_3$) δ = 7.43-7.17 (m, 5H, H-9 – H-13), 6.95 (d, $^{3}J_{HH}$ = 10.1 Hz, 1H, H-5), 6.13 (d, $^{3}J_{HH}$ = 10.2 Hz, 1H, H-6), 2.48-2.08 (m, 4H, H-2, H-3), 1.57 (s, 3H, H-7) ppm.

^{13}**C-NMR** (75.53 MHz, $CDCl_3$) δ = 199.6 (C-1), 157.2 (C-5), 145.4 (C-8), 128.8 (C-10, C-12), 128.8 (C-6), 126.7 (C-11), 126.2 (C-9, C-13), 40.7 (C-4), 38.3 (C-3), 34.8 (C-2), 27.7 (C-7) ppm.

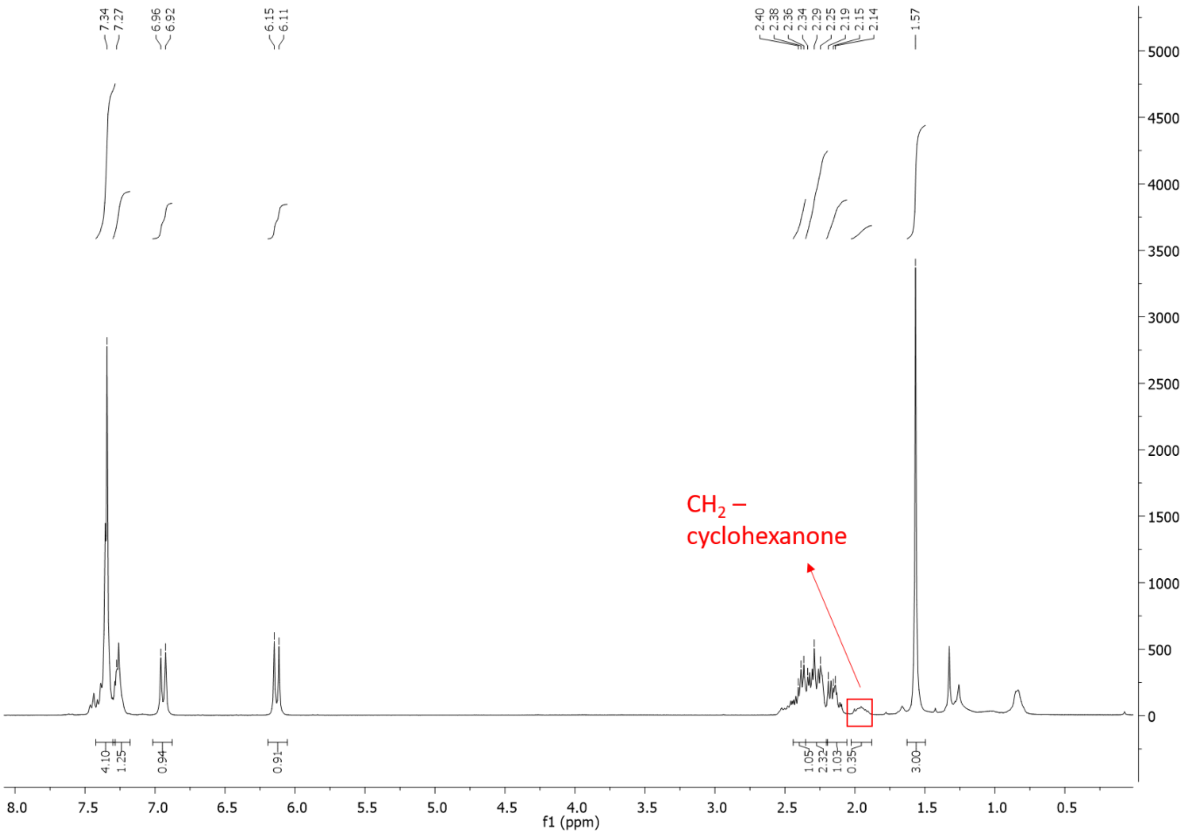

Figure S1. ^{1}H-NMR of **2a** isolated from YqjM cell crude lysate based biotransformation (15 mM NADH). Signal referring to the contained cyclohexanone (overreduction) highlighted in red.

Chiral HPLC (racemic reference (***rac*-2a**) shown in Section 4.3.3):

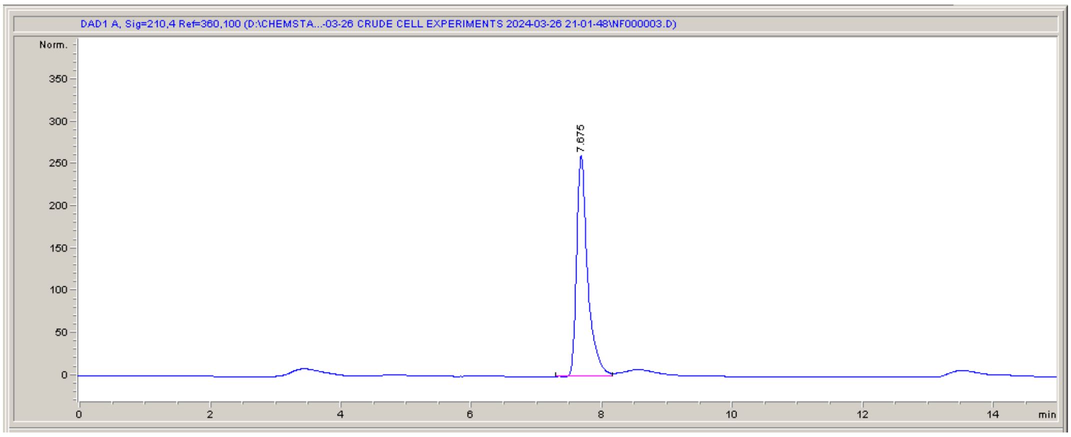

#	Time	Area	Height	Width	Area%	Symmetry
1	7.675	3089.6	261.2	0.174	100.000	0.614

4.2.6 (*S*)-1-Methyl-2,3-dihydro-[1,1'-biphenyl]-4(1*H*)-one (2a) – preparative scale-crude cell lysate with_1.1 eq NADH

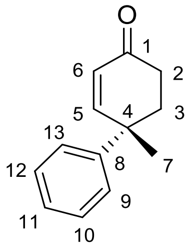

For the large scale biotransformation of **1a** using crude cell lysate of YqjM wt 24 biotransformations were set up in parallel. 35 mg (0.19 mmol, 1 eq) **1a** were used as substrate and distributed between the 24 individual biotransformations. For setting up the individual biotransformations a stock solution of **1a** (1.47 mg **1a** dissolved in 72 µL DMSO) was placed in an Eppendorf tube. Then 417 µL NaPi (50 mM, 150 mM NaCl, pH = 7.5), 267 µL crude cell lysate (1 g cell pellet/5 mL NaPi) and 44 µL of a NADH stock solution (200 mM in NaPi) were added. After gentle vortexing the resulting biotransformations were incubated (450 rpm, 30 °C, 18 h). After full conversion was indicated by GC-MS the individual biotransformations were pooled together and extracted with EtOAc (3 x 25 mL). The combined organic layers were washed with brine (5 mL) and dried over Na_2SO_4. The solvent was removed under reduced pressure. Purification was achieved via column chromatography [50 mL silica, CH/EtOAc 18/1 – CH/EtOAc 8/1, fraction size: 10 mL].

$C_{13}H_{14}O$ [186.25 g/mol]

Yield 20 mg (0.11 mmol, 58%; contains 8% cyclohexanone), colourless oil

$[\alpha]_D^{24}$ -91 (0.6, EtOH)

^{1}H-NMR (300.36 MHz, $CDCl_3$) δ = 7.43-7.17 (m, 5H, H-9 – H-13), 6.95 (d, $^3J_{HH}$ = 10.1 Hz, 1H, H-5), 6.13 (d, $^3J_{HH}$ = 10.2 Hz, 1H, H-6), 2.48-2.08 (m, 4H, H-2, H-3), 1.57 (s, 3H, H-7) ppm.

^{13}C-NMR (75.53 MHz, $CDCl_3$) δ = 199.6 (C-1), 157.2 (C-5), 145.4 (C-8), 128.8 (C-10, C-12), 128.8 (C-6), 126.7 (C-11), 126.2 (C-9, C-13), 40.7 (C-4), 38.3 (C-3), 34.8 (C-2), 27.7 (C-7) ppm.

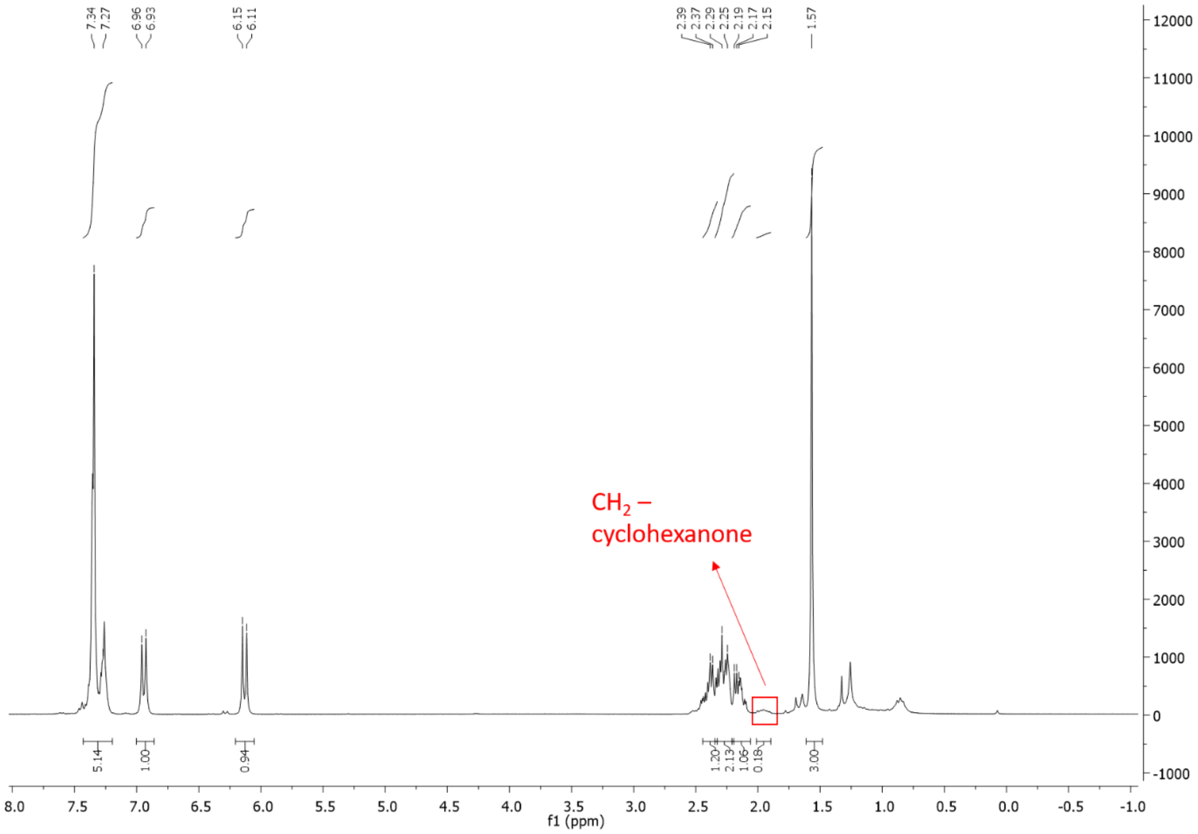

Figure S2. ^{1}H-NMR of **2a** isolated from YqjM cell crude lysate based biotransformation (11 mM NADH). Signal referring to the contained cyclohexanone (overreduction) highlighted in red.

Chiral HPLC (racemic reference (***rac*-2a**) shown in Section 4.3.3):

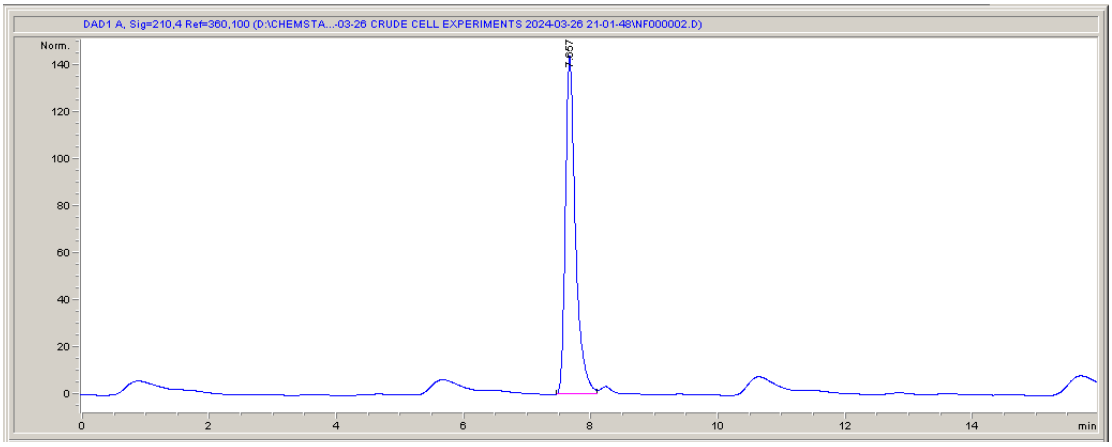

#	Time	Area	Height	Width	Area%	Symmetry
1	7.657	1608.2	144.2	0.1681	100.000	0.65

4.2.7 Optimization – biocatalytic desymmetrization of 1a with YqjM wt – crude cell lysate plus FDH based cofactor recycling system.

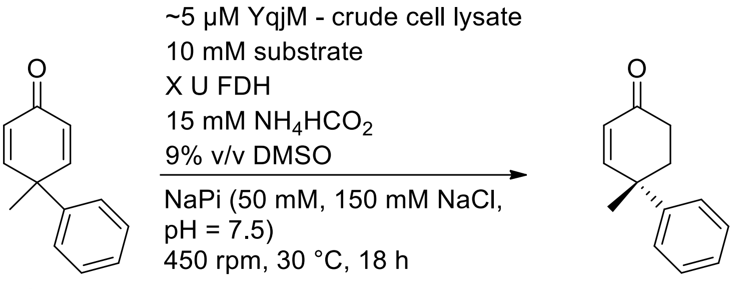

To establish a combination of utilizing crude cell lysate with a FDH based cofactor recycling system for our biocatalytic desymmetrization we screened various FDH loadings. Samples for this screening were prepared as follows (Table S1).

Table S1. Composition of FDH – screening reactions.

Component	2 U FDH	5 U FDH	10 U FDH	20 U FDH
Stock – **1a** (1.1 mg/54 µL DMSO)	54 µL	54 µL	54 µL	54 µL
Stock – NH_4HCO_2 (57 mg /10 mL NaPi)	100 µL	100 µL	100 µL	100 µL
Stock – FDH (25 mg FDH/ 100 µL NaPi; or dilution* thereof)	100 µL 1/5 dilution*	100 µL 1/2 dilution*	100 µL	200 µL
NaPi	146 µL	146 µL	146 µL	46 µL
Crude cell lysate	200 µL	200 µL	200 µL	200 µL

All test reactions were incubated for 18 h (450 rpm, 30 °C). To evaluate the biocatalytic test reactions, they were extracted with EtOAc. The organic layer was dried over Na_2SO_4 and the resulting extract was measured by GC-MS.
The obtained conversions are listed in Table S2.

Table S2. Screening – FDH loading for crude lysate – FDH cofactor recycling based biocatalytic desymmetrization.

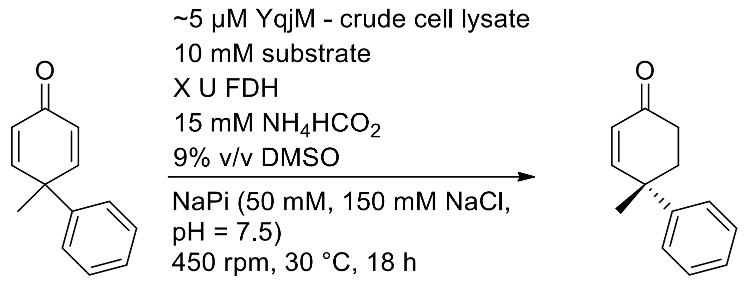

1a **2a**

FDH - loading	GC-MS conversion
2 U	21%
5 U	44%
10 U	90%
20 U	99% (Contains elevated levels of cyclohexanone)

4.2.8 (*S*)-1-Methyl-2,3-dihydro-[1,1'-biphenyl]-4(1*H*)-one (2a) – preparative scale-crude cell lysate_with FDH cofactor recycling

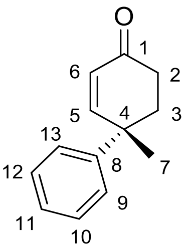

For the preparative scale biotransformation of **1a** using crude cell lysate of YqjM wt in combination with FDH (10 U) based cofactor recycling, 28 biotransformations were set up in parallel. 31 mg (0.17 mmol, 1 eq) **1a** were used as substrate and distributed between the 28 individual biotransformations. For setting up the individual biotransformations a stock solution of **1a** (1.1 mg **1a** dissolved in 54 µL DMSO) was placed in an Eppendorf tube. Then 100 µL NH_4HCO_2 stock (57 mg NH_4HCO_2/ 10 mL NaPi), 100 µL FDH-stock (25 mg/100 µL NaPi), 146 µL NaPi (50 mM, 150 mM NaCl, pH = 7.5) and 200 µL YqjM-crude cell lysate (1 g cell pellet/5 mL NaPi) were added. After gentle vortexing the resulting biotransformations were incubated (450 rpm, 30 °C, 18 h). After full conversion was indicated by GC-MS the individual biotransformations were pooled together and extracted with EtOAc (3 x 25 mL). The combined

organic layers were washed with brine (5 mL) and dried over Na_2SO_4. The solvent was removed under reduced pressure. Purification was achieved via column chromatography [50 mL silica, CH/EtOAc 18/1 – CH/EtOAc 8/1, fraction size: 10 mL].

$C_{13}H_{14}O$ [186.25 g/mol]

Yield 15 mg (0.08 mmol, 48%; contains 12% cyclohexanone), colourless oil

$[\alpha]_D^{24}$ -90 (0.5, EtOH)

^{1}H-NMR (300.36 MHz, $CDCl_3$) δ = 7.43-7.17 (m, 5H, H-9 – H-13), 6.95 (d, $^3J_{HH}$ = 10.1 Hz, 1H, H-5), 6.13 (d, $^3J_{HH}$ = 10.2 Hz, 1H, H-6), 2.48-2.08 (m, 4H, H-2, H-3), 1.57 (s, 3H, H-7) ppm.

^{13}C-NMR (75.53 MHz, $CDCl_3$) δ = 199.6 (C-1), 157.2 (C-5), 145.4 (C-8), 128.8 (C-10, C-12), 128.8 (C-6), 126.7 (C-11), 126.2 (C-9, C-13), 40.7 (C-4), 38.3 (C-3), 34.8 (C-2), 27.7 (C-7) ppm.

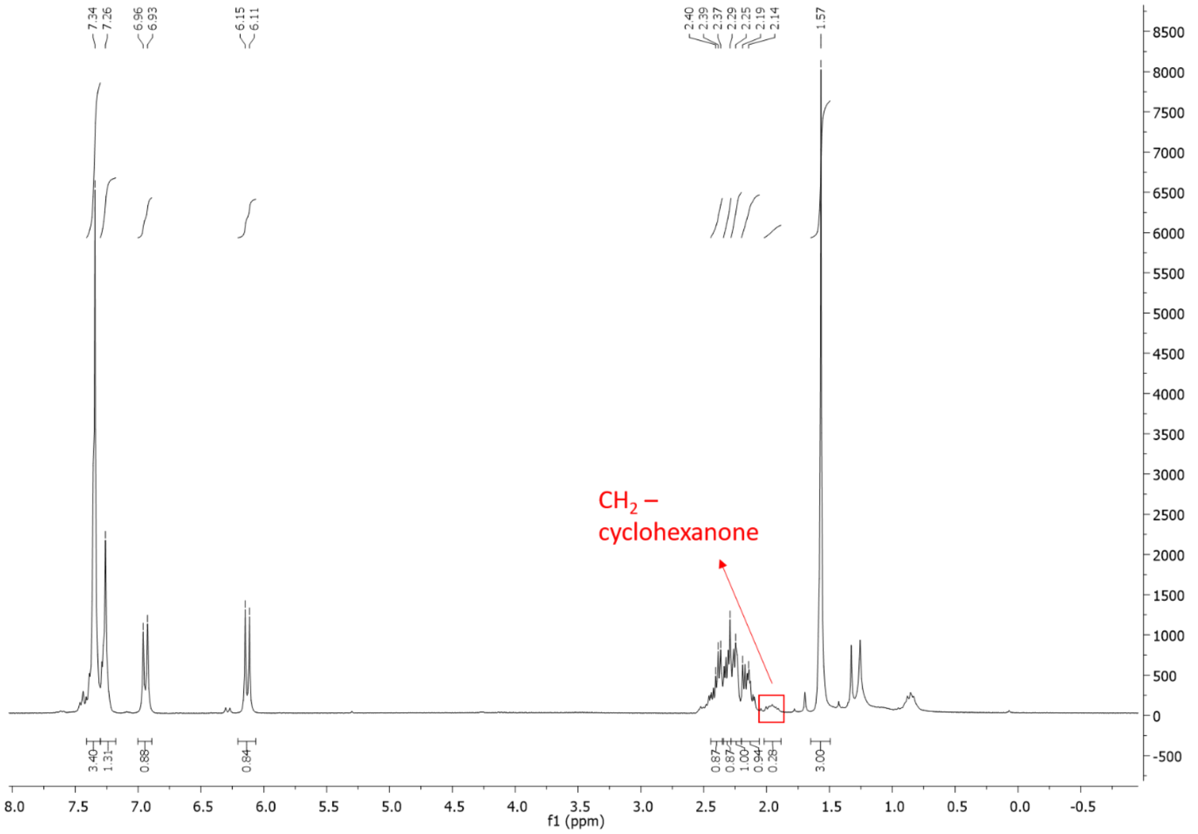

Figure S3. ^{1}H-NMR of **2a** isolated from YqjM cell crude lysate based biotransformation in combination with FDH cofactor recycling. Signal referring to the contained cyclohexanone (overreduction) highlighted in red.

Chiral HPLC (racemic reference (***rac*-2a**) shown in Section 4.3.3):

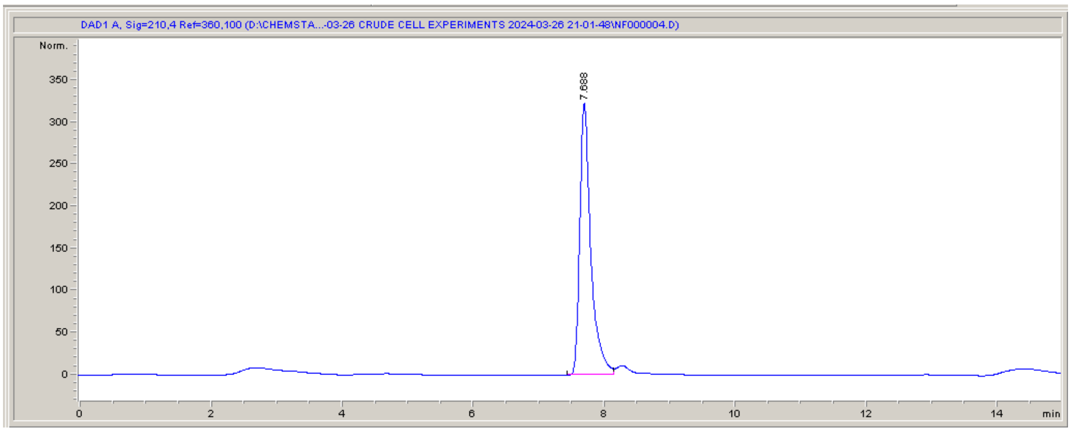

#	Time	Area	Height	Width	Area%	Symmetry
1	7.688	3778.9	322.7	0.1746	100.000	0.604

4.3 Biocatalytic Screenings

4.3.1 Summarized analytical results

HPLC-MS yields of recovered substrates from the biocatalytic screening are summarized in Table S3. Amounts of cyclohexadienones (educt) and cyclohexenones (product) were determined via external calibrations using TBB as external standard. To quantify the amount of generated cyclohexanones the corresponding HPLC-signals at 210 nm were used. For this purpose the calibration curve of 4-methyl-4-phenylcyclohexanone (Section 4.3.3) was adapted for each substrate, based on the relationships of the corresponding cyclohexenone calibration curves to the calibration curve of **2a** (serving as benchmark).

Table S3. Summary of analytical results for the performed biotransformations.

Substrate		Enzyme	Cyclohexa-dienoneone (Educt) [%]	Cyclohexenone [%]	Cylohexanone (Overreduction product) [%]	Mass-Balance [%]
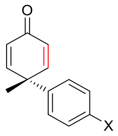	**1a** X: H	OPR3	10	62	13	85
		YqjM	5	78	8	91
	**1b** X: F	OPR3	2	76	4	82
		YqjM	2	63	2	67
	**1c** X: Cl	OPR3	16	70	9	95
		YqjM	4	31	2	37
	**1d** X: Br	OPR3	40	27	11	78
		YqjM	27	33	3	63
	**1e** X: I	OPR3	60	12	1	73
		YqjM	47	12	<1	60
	**1f** X: NO_2	OPR3	78	14	<1	93
		YqjM	56	35	<1	92
	**1g** X: Me	OPR3	35	44	6	85
		YqjM	21	62	2	85
	**1h** X: OMe	OPR3	36	44	6	86
		YqjM	10	47	2	59
**1i**	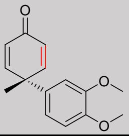	OPR3	37[1]	54[1]	9[1]	100[1]
		YqjM	2[1]	92[1]	6[1]	100[1]
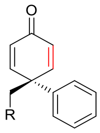	**1j** R: Me	OPR3	20	48	3	71
		YqjM	12	54	3	69
	**1k** R: Et	OPR3	59	11	<1	71
		YqjM	44	19	3	66
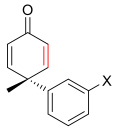	**1l** X: Br	OPR3	8	44	8	60
		YqjM	4	40	<1	45
	**1m** X: I	OPR3	21	44	3	68
		YqjM	7	15	n.d.	22
	**1n** X: NO_2	OPR3	35	48	1	84
		YqjM	66	32	1	99
	**1o** X: OMe	OPR3	12	53	12	77
		YqjM	8	60	8	76
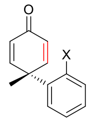	**1p** X: Me	OPR3	91	2	n.d.	93
		YqjM	88	8	n.d.	96
	**1q** X: OMe	OPR3	91	7	n.d.	98
		YqjM	81	17	n.d.	98
	**1r** X: Cl	OPR3	83	6	n.d.	89
		YqjM	85	10	n.d.	95

[1] Quantities normalized relative to a mass balance of 100% in order to account for mass balances exceeding 100% due to experimental error.

**1s** 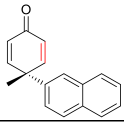	OPR3	>99	<1	n.d.	99
	YqjM	>99	<1	n.d.	99
**1t** 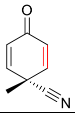	OPR3	33	57	n.d	90
	YqjM	15[2]	85[2]	n.d	100[2]

4.3.2 Comparison of analytical methods for reaction monitoring

For the reaction monitoring we used the following Standard Analytical Procedure:

Standard Analytical Procedure used in this work:	After finished incubation of our biotransformations, 100 µL of each sample were transferred into a separate vial equipped with 500 µL TBB containing DMSO (42 mg TBB/100 mL DMSO as external standard). Due to the emulsive nature of our biotransformations (result of limited substrate solubility), we intensively vortexed the biotransformations just seconds before taking the 100 µL samples thereof. This should ensure that those 100 µL samples were as representative for the overall biotransformation composition as possible. After adding this sample to the DMSO based TBB solution, a homogenous solution was obtained, which was analyzed by HPLC-MS.

In order to rule out that a systematic error was introduced by working with emulsion samples, the following Alternative Analytical Procedure was developed.

Alternative Analytical Procedure used to evaluate the standard analytical procedure:	To the biotransformation (400 µL total volume) containing Eppendorf tubes, 200 µL DMSO were added, followed by vortexing. Due to this step homogenous solutions were obtained. From these solutions, 100 µL samples were transferred into second Eppendorf tubes equipped with 500 µL of a DMSO based TBB solution (42 mg TBB/100 mL DMSO). The resulting solution was then analyzed by HPLC-MS.

[2] Quantities normalized relative to a mass balance of 100% in order to account for mass balances exceeding 100% due to experimental error.

The calibration curves of **1a** and **2a** generated according to the Alternative Analytical Procedure are shown in Figure S4.

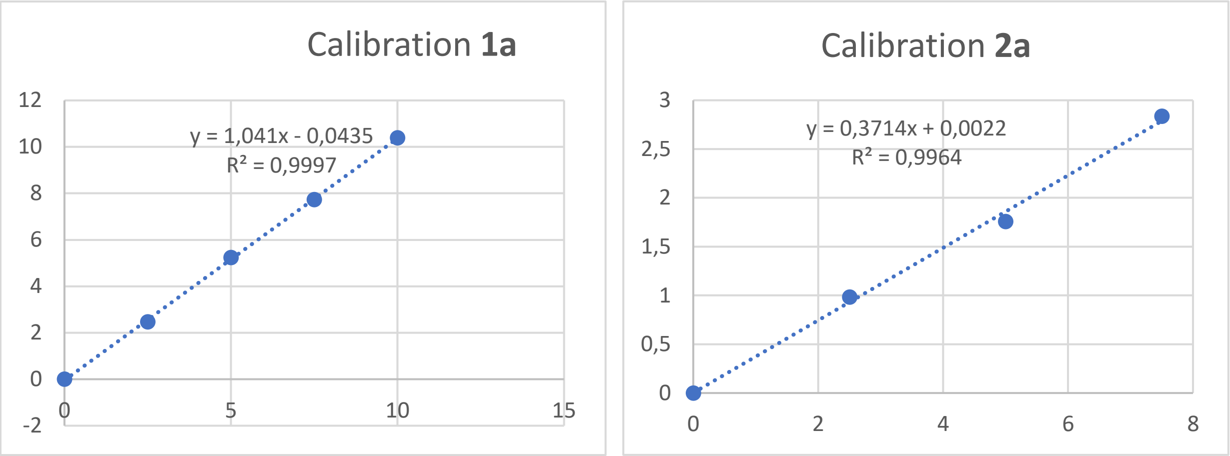

Figure S4. Calibration curves for **1a** and **2a** for the Alternative Analytical Procedure.

New calibration and workup of the Alternative Analytical Procedure were validated by analyzing a sample with known concentrations of **1a** and **2a** (Table S4).

Table S4. Validation of the Alternative Analytical Procedure.

	Actual Concentration	Measured Concentration
**1a**	5 mM	4.78 mM
**2a**	3.75 mM	3.74 mM

Next, the newly established Alternative Analytical Procedure was compared with the Standard Analytical Procedure. For this purpose biotransformations of **1a** with YqjM wt as well as OPR3 wt as biocatalysts were performed (400 µL scale for Alternative Analytical Procedure) and analyzed by the Alternative Analytical Procedure. The results were then compared to the previously obtained results (obtained via Standard Analytical Procedure) (Table S5).

Table S5. Standard vs Alternative Analytical Procedure.

	**Alternative Anal. Proc.**		**Standard Anal. Proc.**	
Biotransformation	**1a**	**2a**	**1a**	**2a**
OPR3	12%	55%	10%	62%
YqjM	3%	79%	4%	78%

The results obtained with the two analytical procedures indicate good consistency between these two methods.

In addition, a series of incubation experiments were performed to investigate a potential instability of substrate **1a** under the standard biocatalytic conditions. These experiments were set up in Eppendorf tubes according to Table S6. First a stock solution of **1a** in DMSO was prepared. Then NaPi and in case of sample **I-Enz** an enzyme solution were added, giving a final volume of 400 µL. Each sample contained **1a** in an initial concentration of 10 mM.

Table S6. Composition - incubation experiments.

Experiment	Stock **-1a**[3] [µL]	NaPi [µL]	YqjM wt [µL]
**I**	36	364	0
**I-Enz**	36	353	11

Each incubation experiment was prepared 4 times in order to obtain two time points (analyzed 1 h and 3 h after preparation) in duplicates (a and b for each time point). After the corresponding time points the incubation experiments were analyzed by the Alternative Analytical Procedure (described on page 78). The obtained results are summarized in Table S7.

Table S7. Results – Incubation experiments.

	1 h	3h
Experiment	**1a** [mM]	**1a** [mM]
**I a**	9.39	8.55
**I b**	9.33	8.59
**I-Enz a**	9.44	8.70
**I-Enz b**	9.39	8.53

The incubation experiments shown above clearly demonstrated a decrease of the amount of **1a** with increasing incubation time. Within each set of duplicates the observed values for the individual experiments match rather closely. Furthermore, it could be shown that the enzyme presence did not have a significant incluence on the amount of **1a** found by HPLC-MS.
The ultimate cause for the lost substrate within the performed biotransformations could not be identified as no defined side products could be observed with either GC-MS or HPLC-MS.

[3] Resulting in an overall substrate concentration of 10 mM in the final incubation experiments.

4.3.3 Desymmetrization of 1a

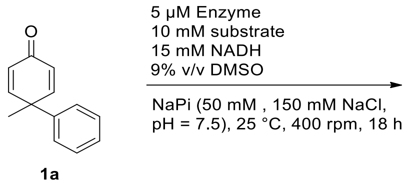

Enzyme	HPLC yield – **2a** (%)	HPLC recovery – **1a** (%)	ee – **2a** (%)
OPR3	62	10	99
YqjM	78	5	>99

Calibration – ***rac*-2a**:

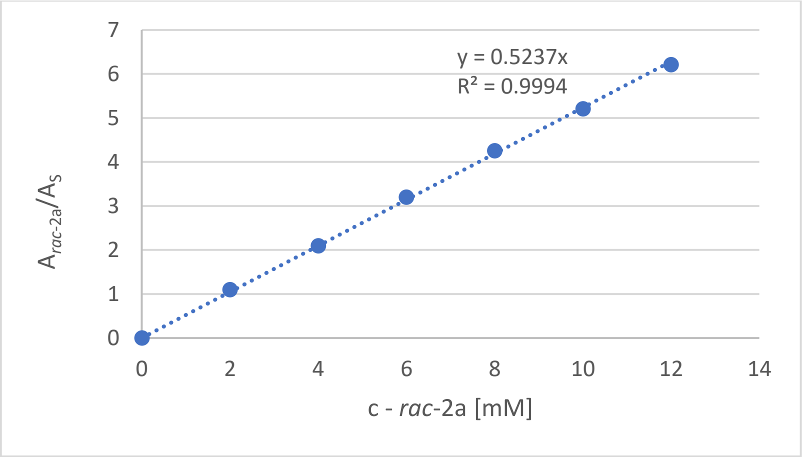

Calibration – **1a**:

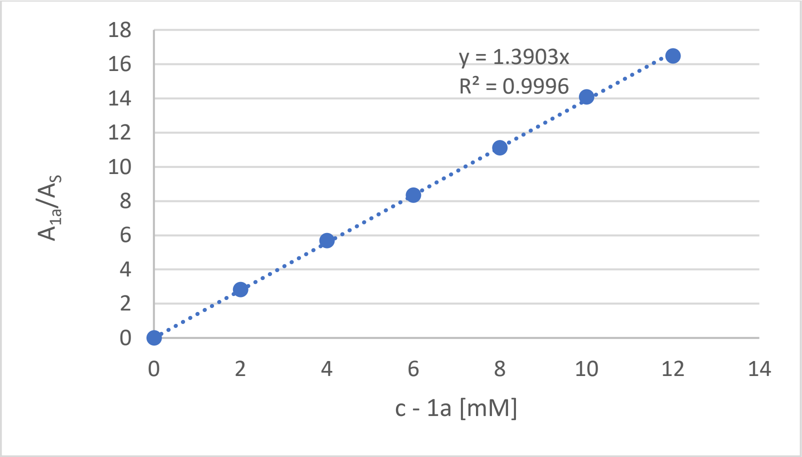

Calibration – **4-methyl-4-phenylcyclohexanone** (210 nm):

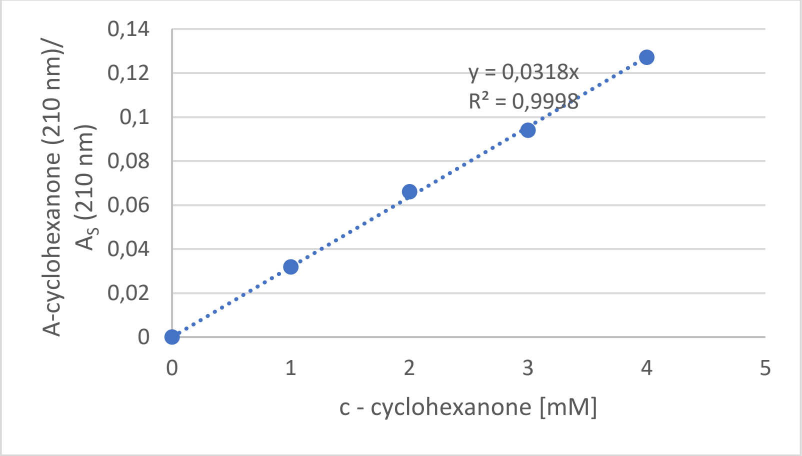

Chiral HPLC – ***rac*-2a:**

(CHIRACEL® OJ-H; Heptane/i-PrOH 1/1; 0.7 mL/min; 25 °C)

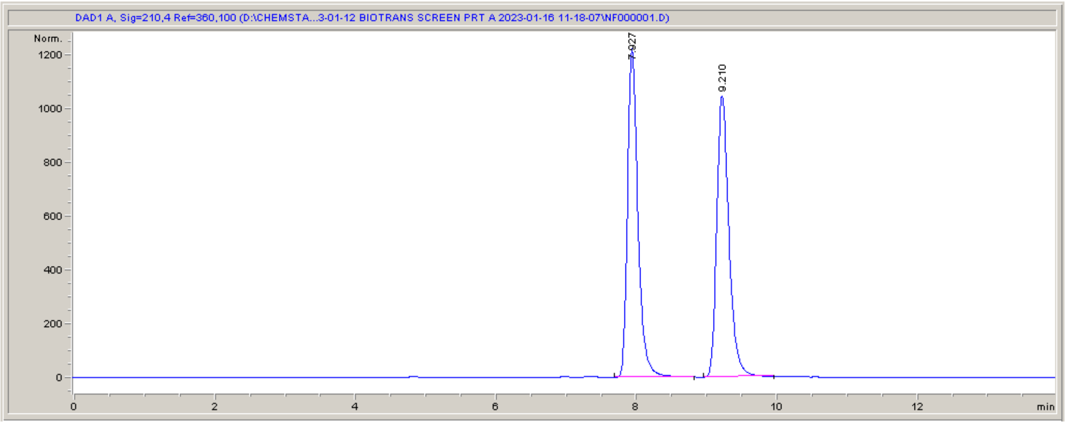

#	Time	Area	Height	Width	Area%	Symmetry
1	7.927	12672	1224.9	0.1587	50.089	0.693
2	9.21	12627.2	1047.3	0.1847	49.911	0.71

Chiral HPLC – biotransformation **1a**_OPR3:

(CHIRACEL® OJ-H; Heptane/i-PrOH 1/1; 0.7 mL/min; 25 °C)

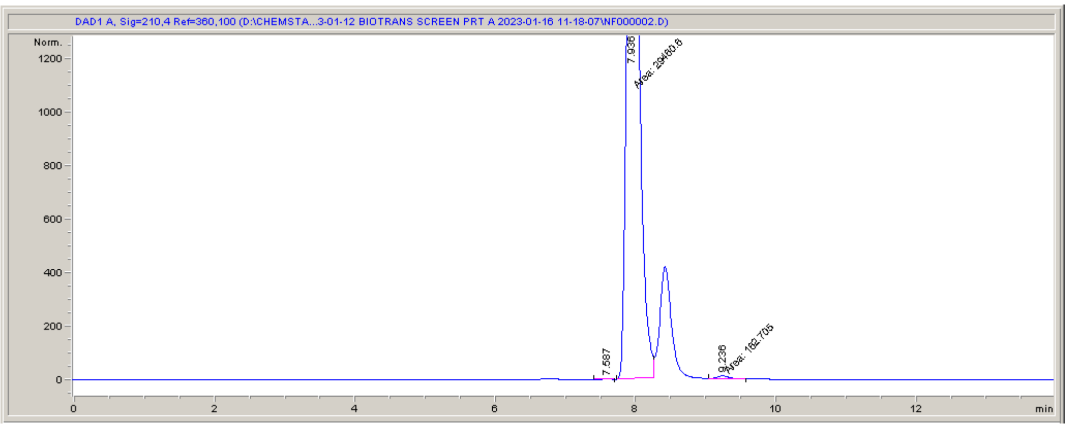

#	Time	Area	Height	Width	Area%	Symmetry
1	7.587	22.7	2.8	0.1296	0.076	0.988
2	7.936	29460.6	2314.6	0.2121	99.375	0.657
3	9.236	162.7	12.4	0.2193	0.549	0.905

Chiral HPLC – biotransformation **1a**_YqjM:

(CHIRACEL® OJ-H; Heptane/i-PrOH 1/1; 0.7 mL/min; 25 °C)

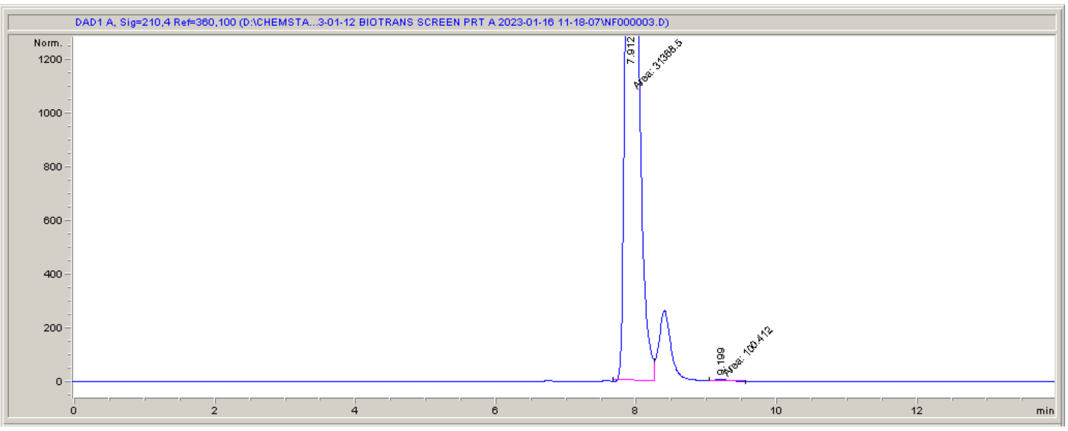

#	Time	Area	Height	Width	Area%	Symmetry
1	7.912	31368.5	2354.3	0.2221	99.681	0.618
2	9.199	100.4	7.2	0.2335	0.319	0.88

4.3.4 Desymmetrization of 1b

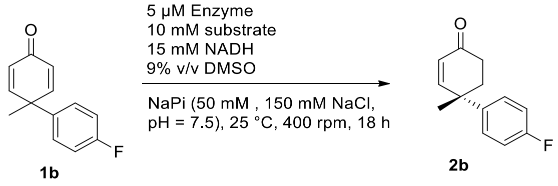

Enzyme	HPLC yield – **2b** (%)	HPLC recovery – **1b** (%)	ee – **2b** (%)
OPR3	76	2	>99
YqjM	63	2	>99

Calibration – ***rac*-2b:**

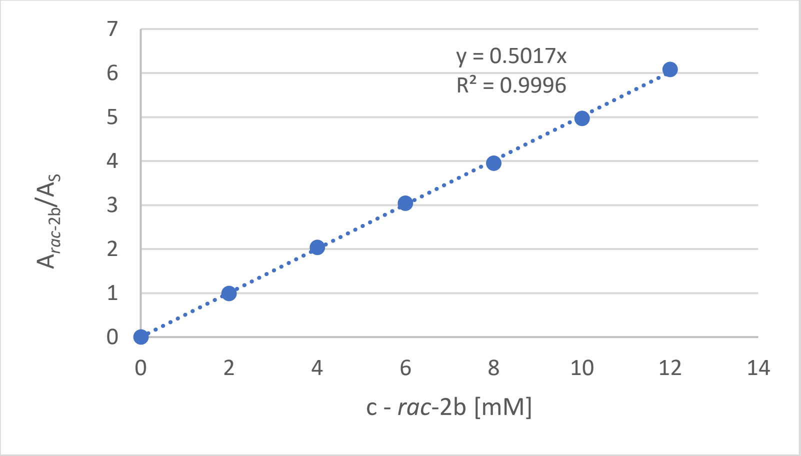

Calibration – **1b:**

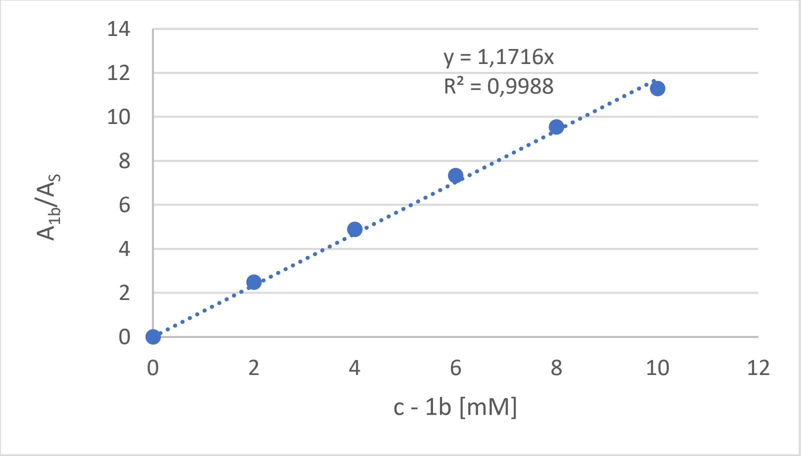

Chiral HPLC – ***rac*-2b:**

(CHIRACEL® OJ-H; Heptane/i-PrOH 2/1; 0.7 mL/min; 25 °C)

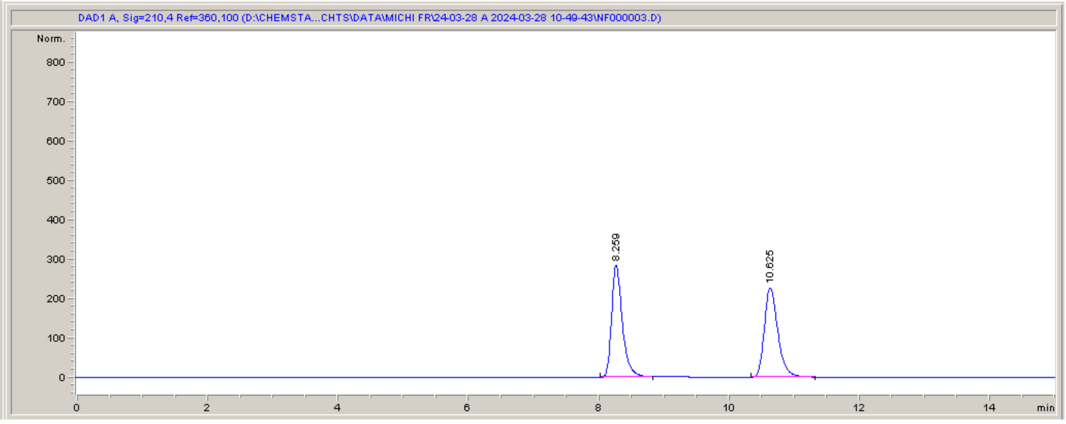

#	Time	Area	Height	Width	Area%	Symmetry
1	8.259	3286.1	284.6	0.1747	50.156	0.66
2	10.625	3265.6	226.7	0.22	49.844	0.716

Chiral HPLC – biotransformation **1b**_OPR3:

(Heptane/i-PrOH 2/1; 0.7 mL/min; 25 °C)

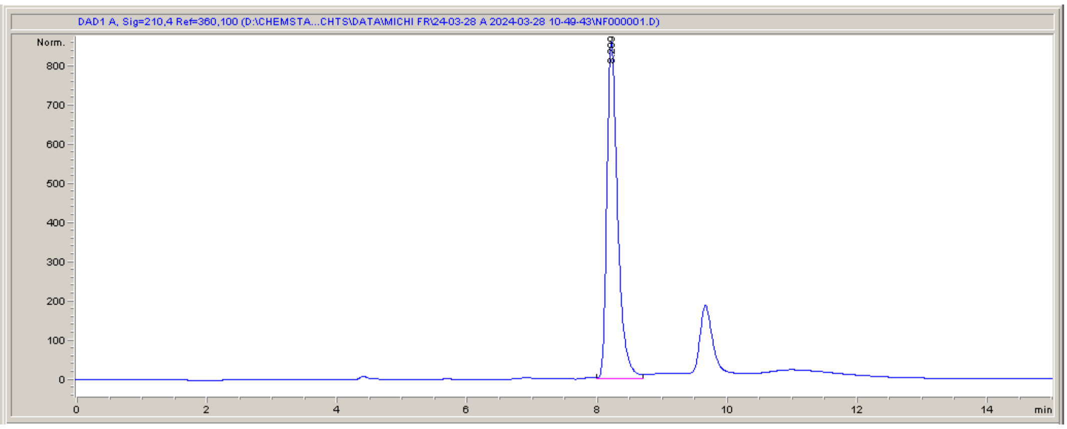

#	Time	Area	Height	Width	Area%	Symmetry
1	8.209	9965.4	859.9	0.1752	100.000	0.634

Chiral HPLC – biotransformation **1b**_YqjM:

(CHIRACEL® OJ-H; Heptane/i-PrOH 2/1; 0.7 mL/min; 25 °C)

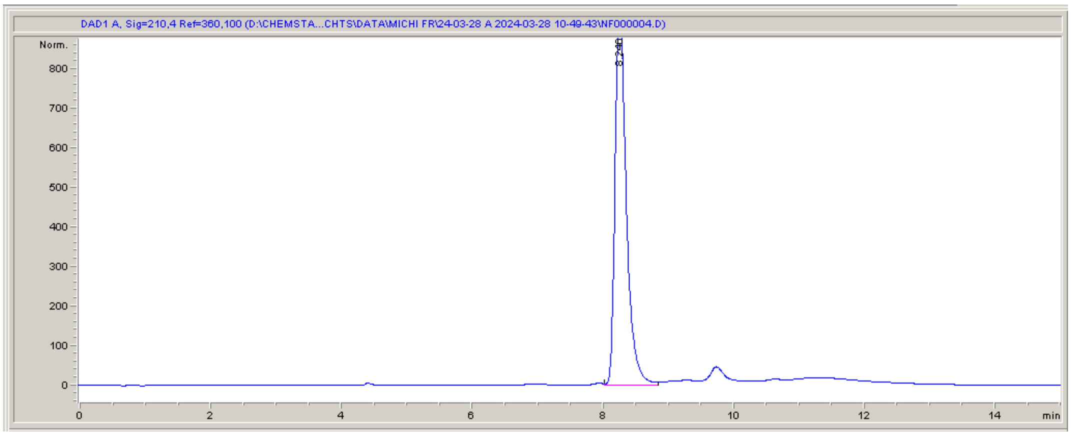

#	Time	Area	Height	Width	Area%	Symmetry
1	8.246	11582.2	981.6	0.1777	100.000	0.612

4.3.5 Desymmetrization of 1c

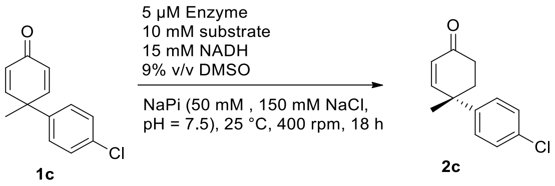

Enzyme	HPLC yield – **2c** (%)	HPLC recovery – **1c** (%)	ee – **2c** (%)
OPR3	70	16	>99
YqjM	31	4	>99

Calibration ***rac*-2c:**

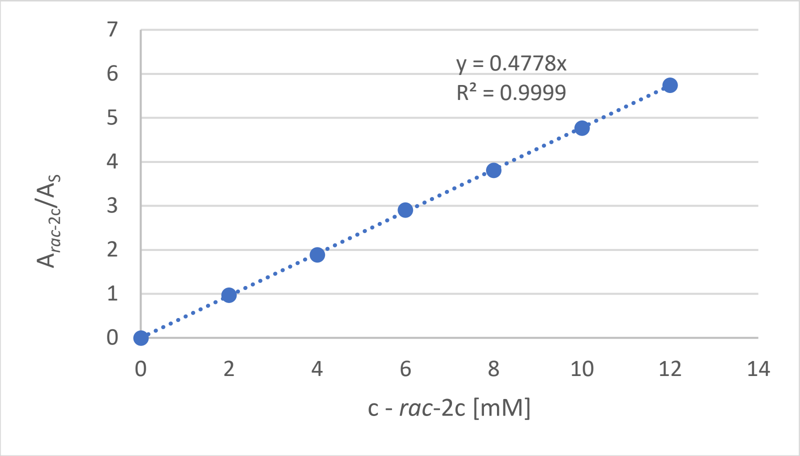

Calibration **1c:**

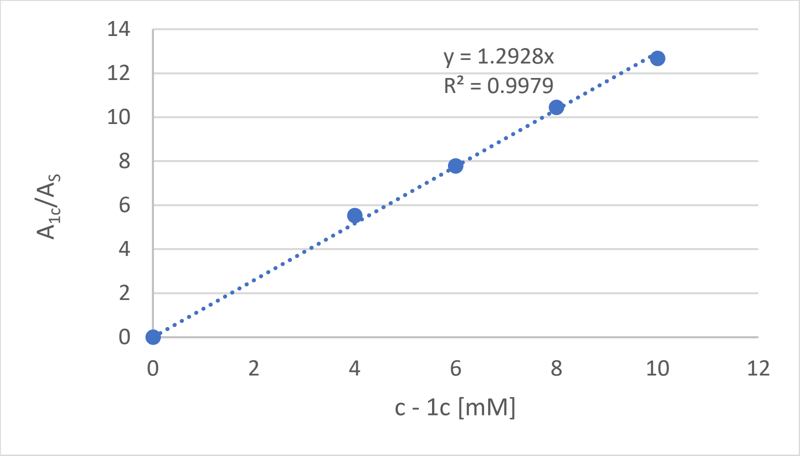

Chiral HPLC ***rac*-2c:**

(CHIRACEL® OJ-H; Heptane/i-PrOH 1/1; 0.7 mL/min; 25 °C)

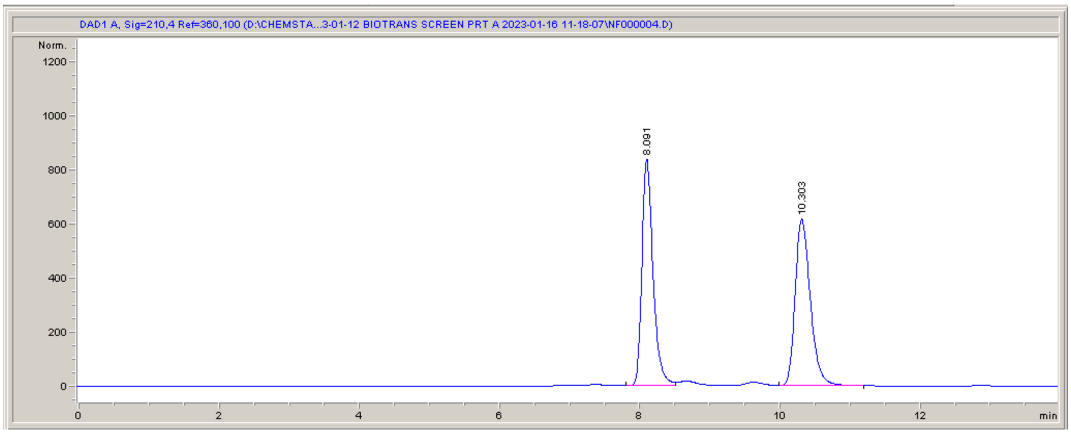

#	Time	Area	Height	Width	Area%	Symmetry
1	8.091	9306.3	840.5	0.1672	49.963	0.697
2	10.303	9319.9	617	0.2322	50.037	0.718

Chiral HPLC – biotransformation **1c**_OPR3:

(CHIRACEL® OJ-H; Heptane/i-PrOH 1/1; 0.7 mL/min; 25 °C)

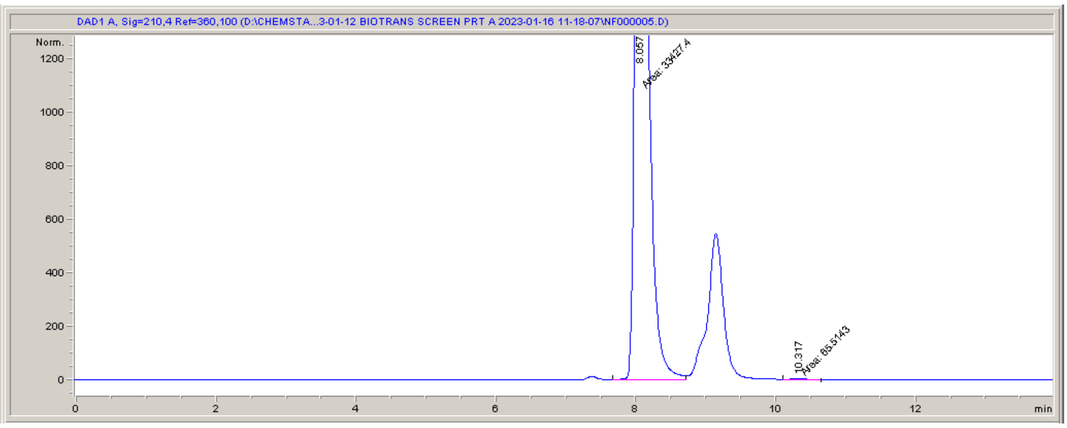

#	Time	Area	Height	Width	Area%	Symmetry
1	8.057	33427.4	2357.1	0.2364	99.804	0.603
2	10.317	65.5	4	0.2713	0.196	0.889

Chiral HPLC – biotransformation **1c**_YqjM:

(CHIRACEL® OJ-H; Heptane/i-PrOH 1/1; 0.7 mL/min; 25 °C)

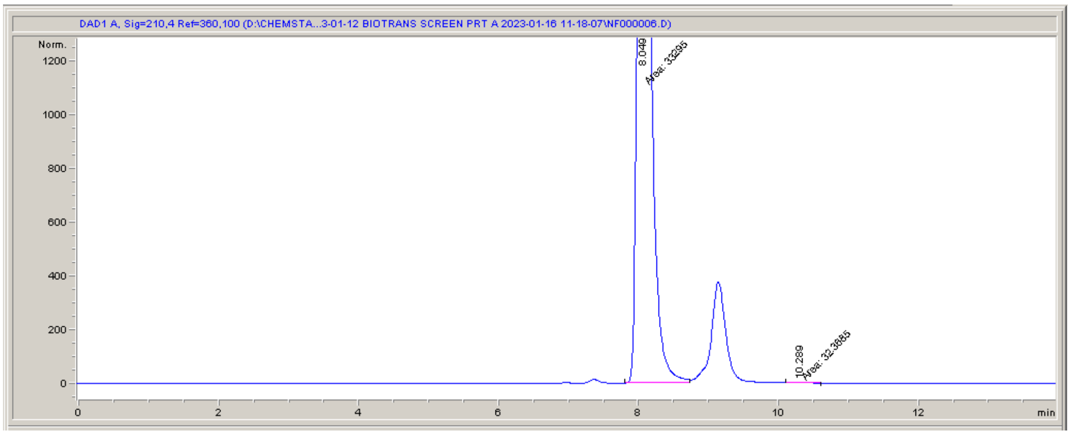

#	Time	Area	Height	Width	Area%	Symmetry
1	8.049	33295	2354.7	0.2357	99.903	0.602
2	10.289	32.4	1.9	0.2821	0.097	0.866

4.3.6 Desymmetrization of 1d

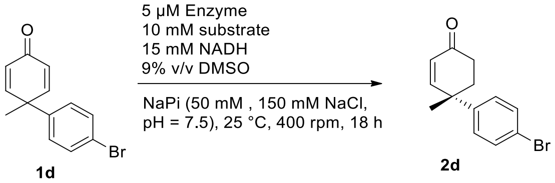

Enzyme	HPLC yield – **2d** (%)	HPLC recovery – **1d** (%)	ee – **2d** (%)
OPR3	27	40	99
YqjM	33	27	>99

Calibration – ***rac*-2d**:

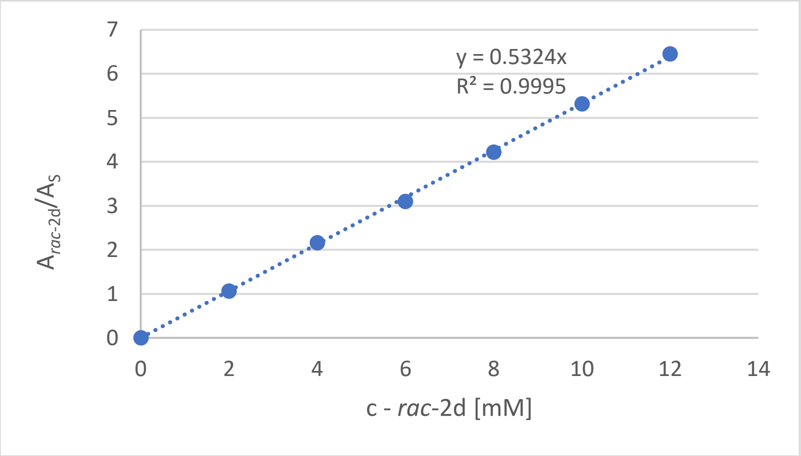

Calibration – **1d:**

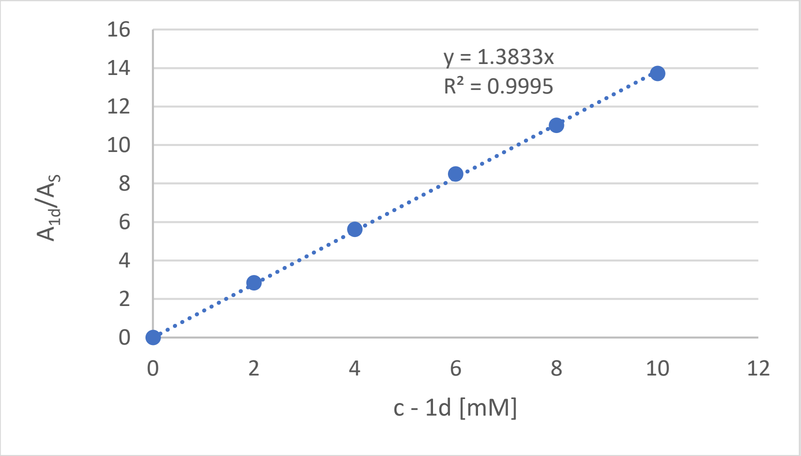

Chiral HPLC – ***rac*-2d:**

(CHIRACEL® OJ-H; Heptane/i-PrOH 1/1; 0.7 mL/min; 25 °C)

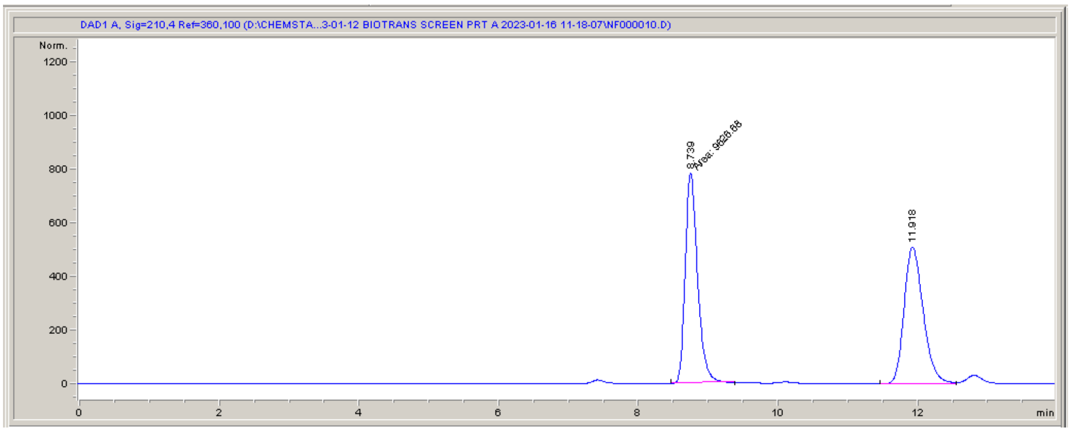

#	Time	Area	Height	Width	Area%	Symmetry
1	8.739	9626.7	784.3	0.2046	49.686	0.73
2	11.918	9748.3	510.5	0.2948	50.314	0.742

Chiral HPLC – biotransformation **1d**_OPR3:

(CHIRACEL® OJ-H; Heptane/i-PrOH 1/1; 0.7 mL/min; 25 °C)

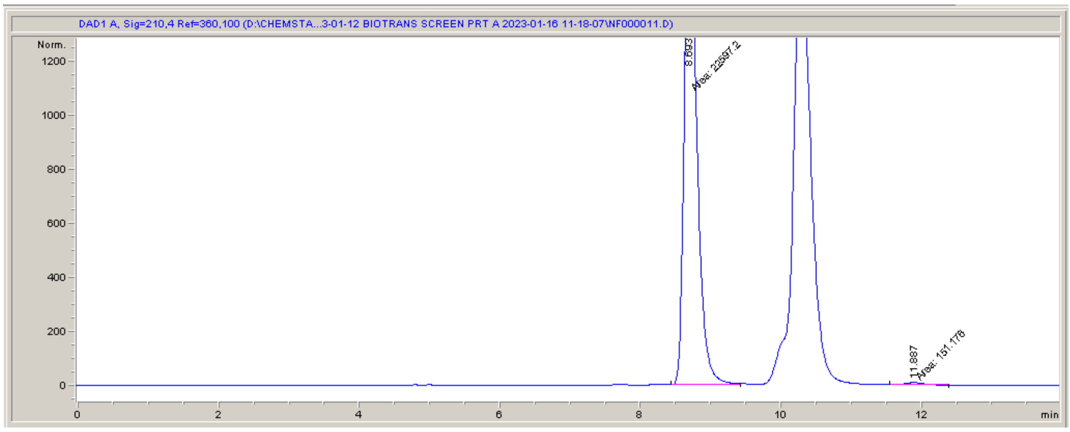

#	Time	Area	Height	Width	Area%	Symmetry
1	8.693	22597.2	1758	0.2142	99.335	0.676
2	11.887	151.2	8.3	0.3029	0.665	0.801

Chiral HPLC – biotransformation **1d**_YqjM:

(CHIRACEL® OJ-H; Heptane/i-PrOH 1/1; 0.7 mL/min; 25 °C)

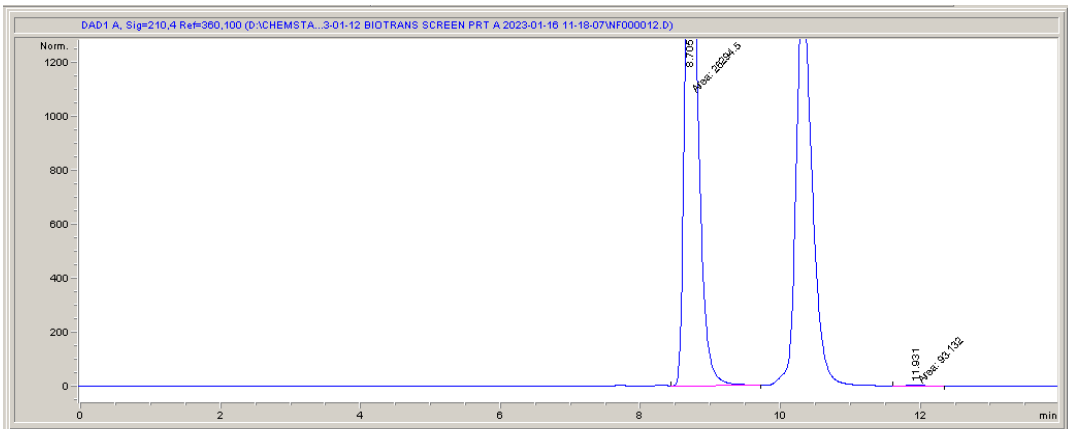

#	Time	Area	Height	Width	Area%	Symmetry
1	8.705	26294.5	1979.6	0.2214	99.647	0.658
2	11.931	93.1	4.1	0.3752	0.353	0.775

4.3.7 Desymmetrization of 1e

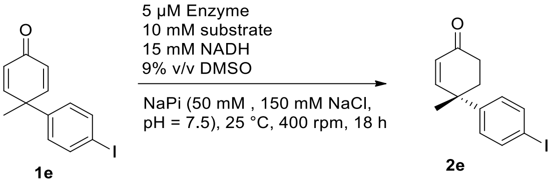

Enzyme	HPLC yield – **2e** (%)	HPLC recovery – **1e** (%)	ee – **2e** (%)
OPR3	12	60	n.d.
YqjM	12	47	n.d.

Calibration – ***rac*-2e:**

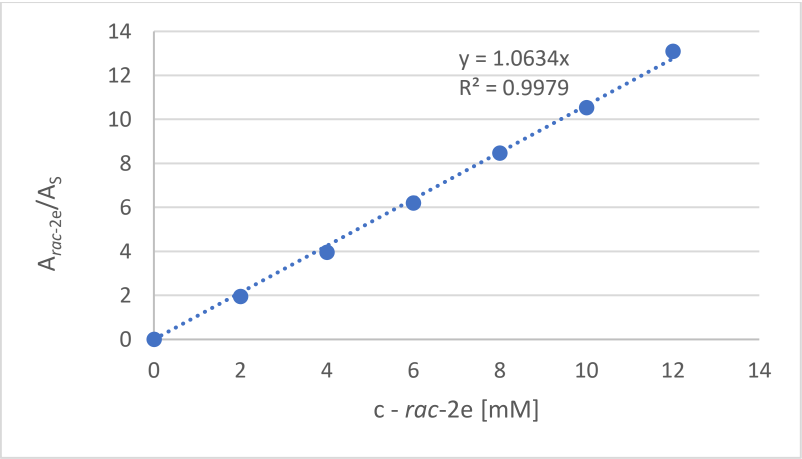

Calibration – **1e:**

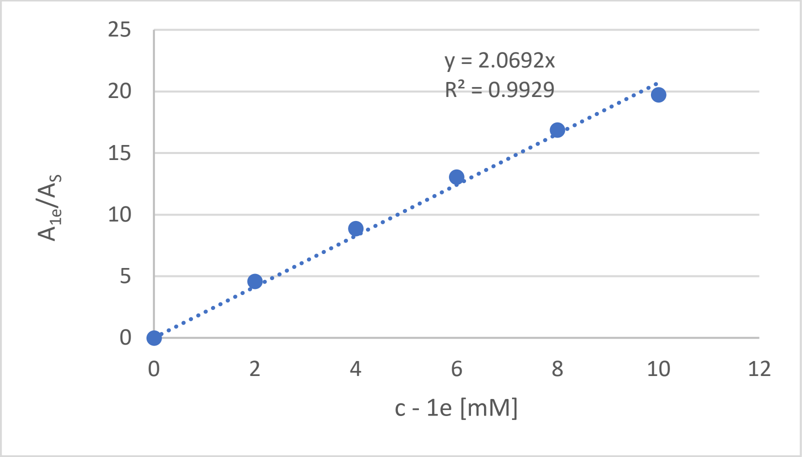

4.3.8 Desymmetrization of 1f

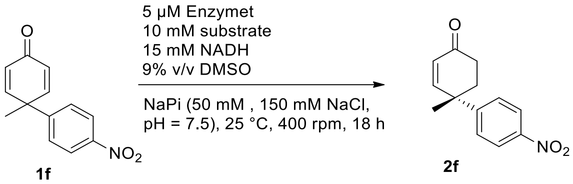

Enzyme	HPLC yield – **2f** (%)	HPLC recovery – **1f** (%)	ee – **2f** (%)
OPR3	14	78	86
YqjM	35	56	94

Calibration – ***rac*-2f:**

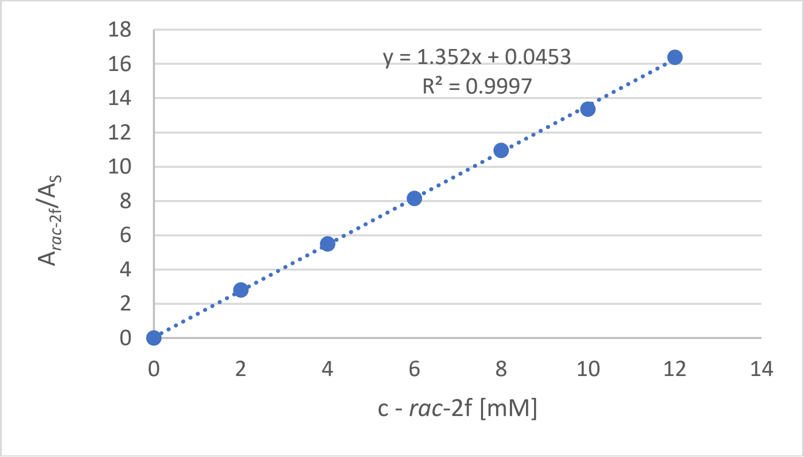

Calibration – **1f:**

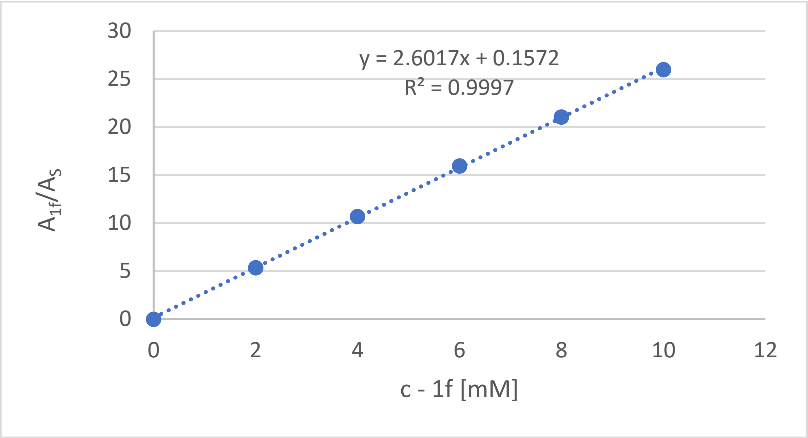

Chiral HPLC – ***rac*-2f:**
(CHIRACEL® OJ-H; Heptane/EtOH 4/1; 0.7 mL/min; 25 °C)

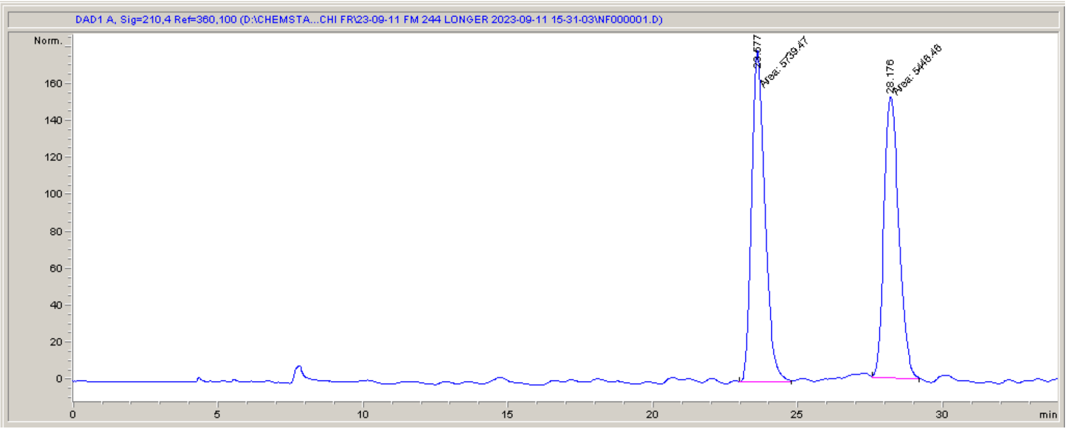

#	Time	Area	Height	Width	Area%	Symmetry
1	23.577	5739.5	179.1	0.534	51.310	0.727
2	28.176	5446.5	152	0.5973	48.690	0.77

Chiral HPLC – biotransformation **1f**_OPR3:

(CHIRACEL® OJ-H; Heptane/EtOH 4/1; 0.7 mL/min; 25 °C)

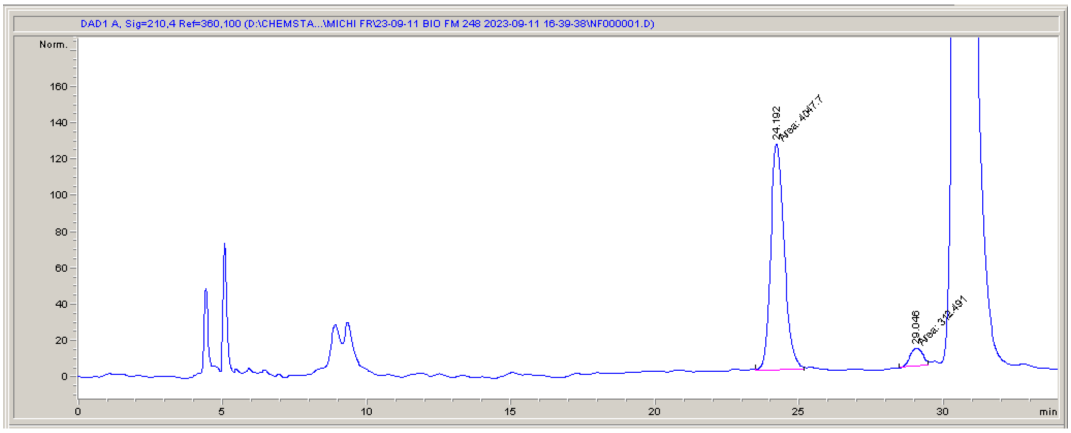

#	Time	Area	Height	Width	Area%	Symmetry
1	24.192	4047.7	124.5	0.5417	92.833	0.767
2	29.046	312.5	10.1	0.5166	7.167	1.034

Chiral HPLC – biotransformation **1f**_YqjM:

(CHIRACEL® OJ-H; Heptane/EtOH 4/1; 0.7 mL/min; 25 °C)

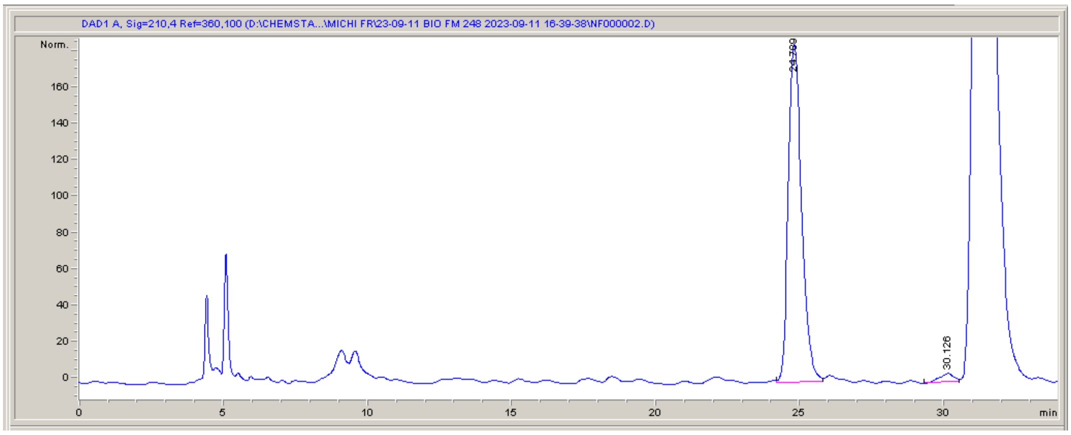

#	Time	Area	Height	Width	Area%	Symmetry
1	24.789	6009.7	185.8	0.5014	96.870	0.708
2	30.126	194.2	5.1	0.496	3.130	1.394

4.3.9 Desymmetrization of 1g

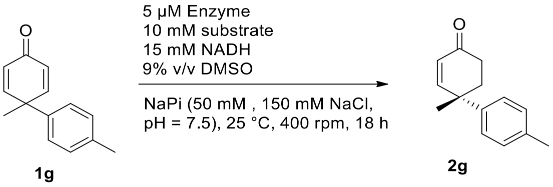

Enzyme	HPLC yield – **2g** (%)	HPLC recovery – **1g** (%)	ee – **2g** (%)
OPR3	44	35	>99
YqjM	62	21	>99

Calibration – ***rac*-2g:**

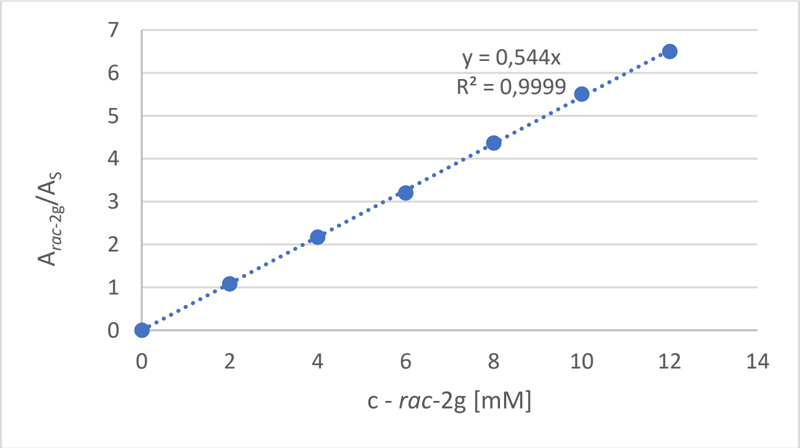

Calibration – **1g:**

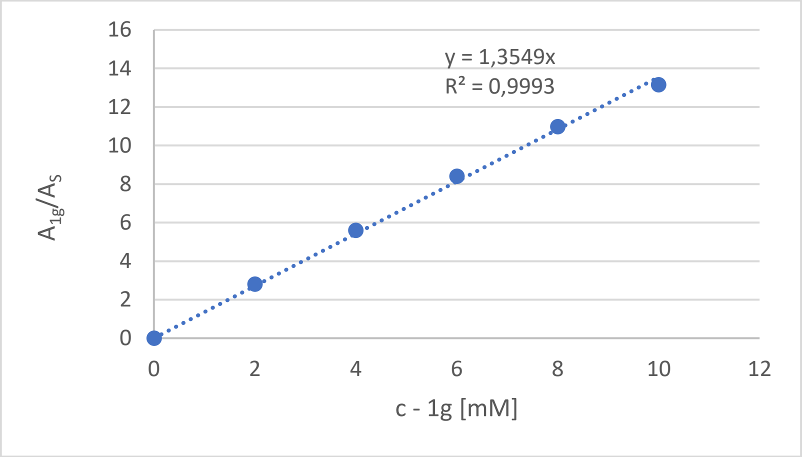

Chiral HPLC – ***rac*-2g:**
(CHIRACEL® OJ-H; Heptane/i-PrOH 4/1; 0.7 mL/min; 25 °C)

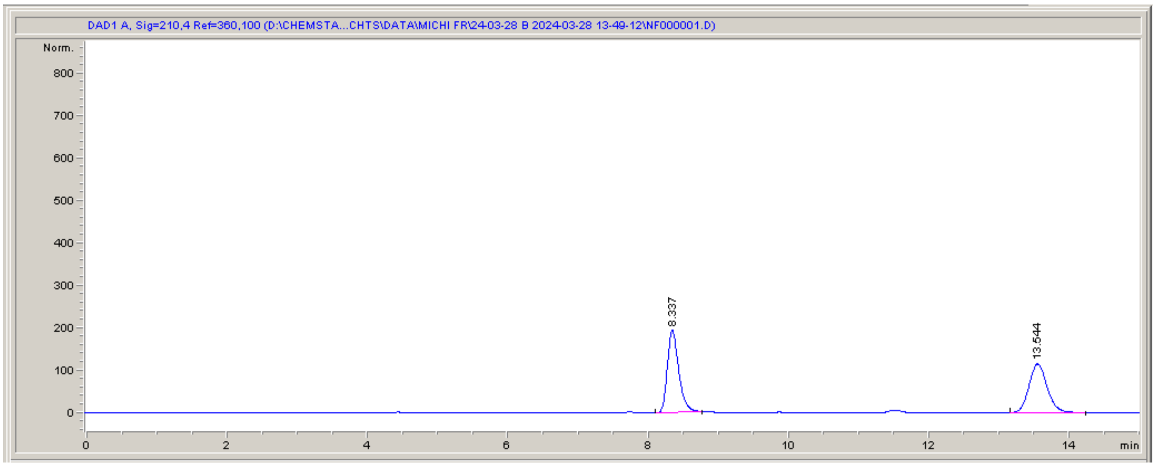

#	Time	Area	Height	Width	Area%	Symmetry
1	8.337	2223.2	194.4	0.1734	51.421	0.698
2	13.544	2100.3	115.2	0.2808	48.579	0.851

Chiral HPLC – biotransformation **1g**_OPR3:

(CHIRACEL® OJ-H; Heptane/i-PrOH 4/1; 0.7 mL/min; 25 °C)

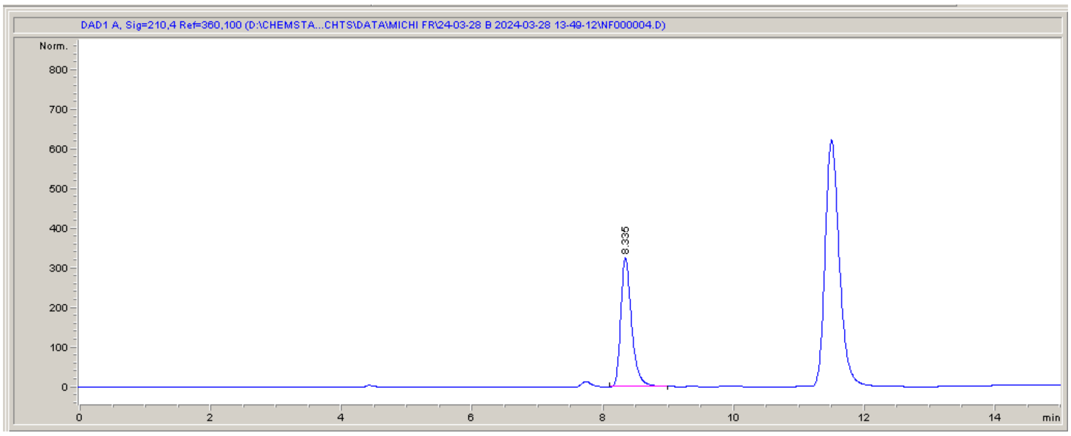

#	Time	Area	Height	Width	Area%	Symmetry
1	8.335	3768.4	325.5	0.1751	100.000	0.677

Chiral HPLC – biotransformation **1g**_YqjM:

(CHIRACEL® OJ-H; Heptane/i-PrOH 4/1; 0.7 mL/min; 25 °C)

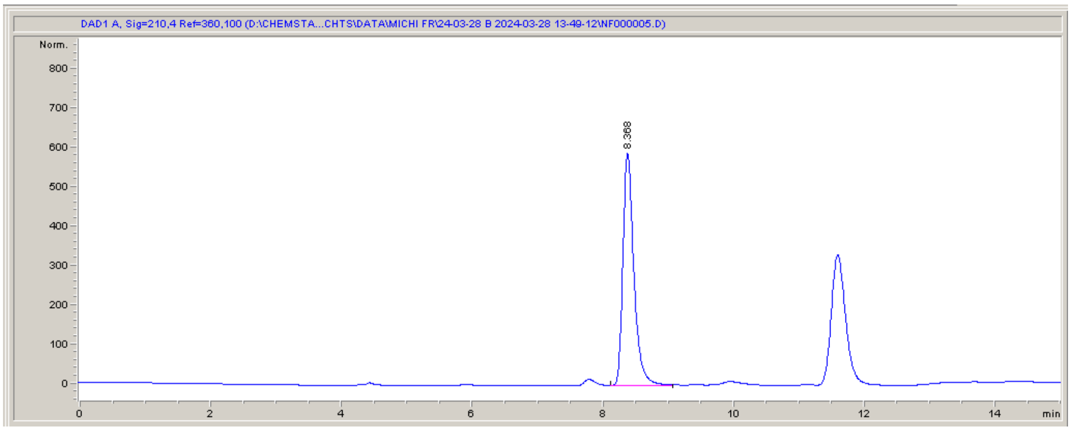

#	Time	Area	Height	Width	Area%	Symmetry
1	8.368	6936.2	591	0.1769	100.000	0.663

4.3.10 Desymmetrization of 1h

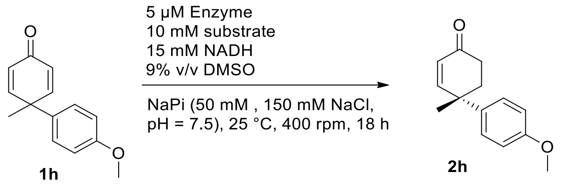

Enzyme	HPLC yield – **2h** (%)	HPLC recovery – **1h** (%)	ee – **2h** (%)
OPR3	44	36	99
YqjM	47	10	>99

Calibration – ***rac*-2h:**

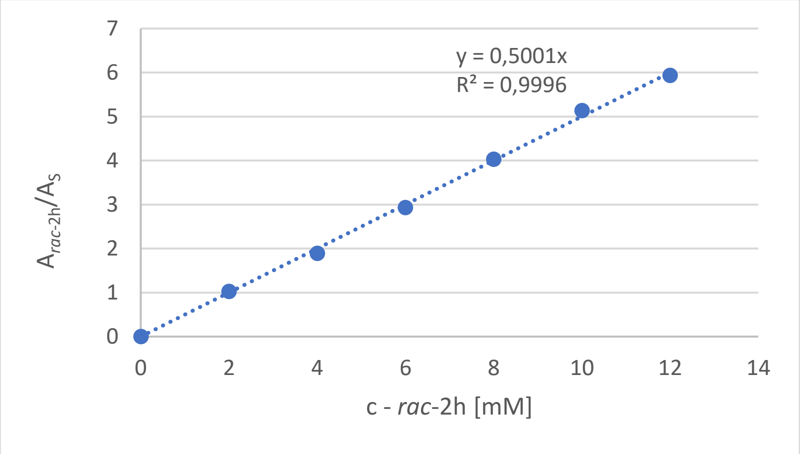

Calibration – **1h:**

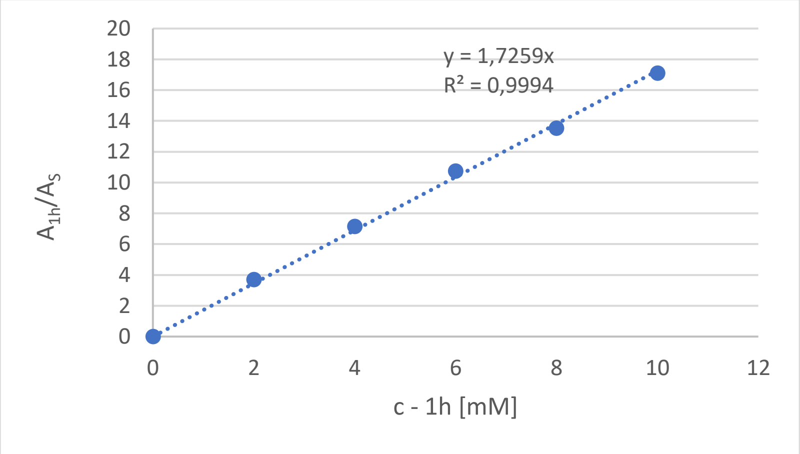

Chiral HPLC – ***rac*-2h:**
(CHIRACEL® OJ-H; Heptane/i-PrOH 1/1; 0.7 mL/min; 25 °C)

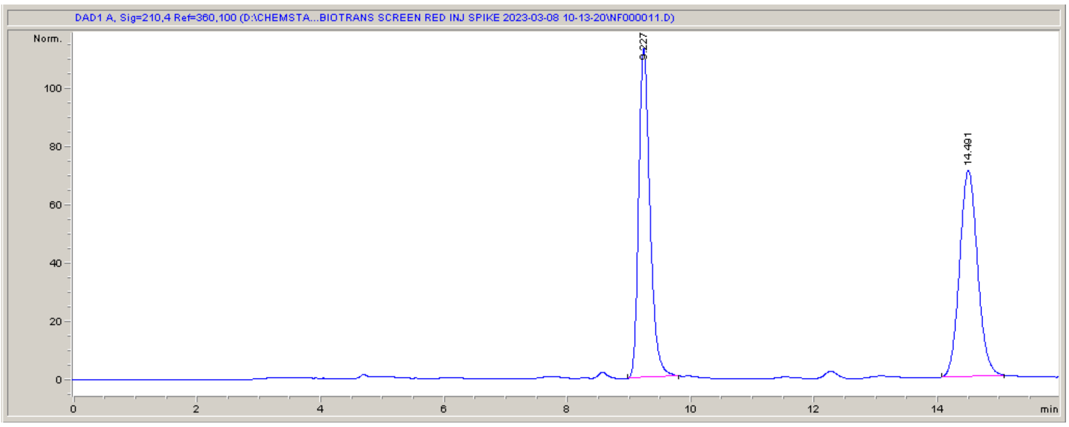

#	Time	Area	Height	Width	Area%	Symmetry
1	9.227	1434.5	113	0.1921	49.961	0.73
2	14.491	1436.7	70.9	0.3125	50.039	0.902

Chiral HPLC – biotransformation **1h**_OPR3:

(CHIRACEL® OJ-H; Heptane/i-PrOH 1/1; 0.7 mL/min; 25 °C)

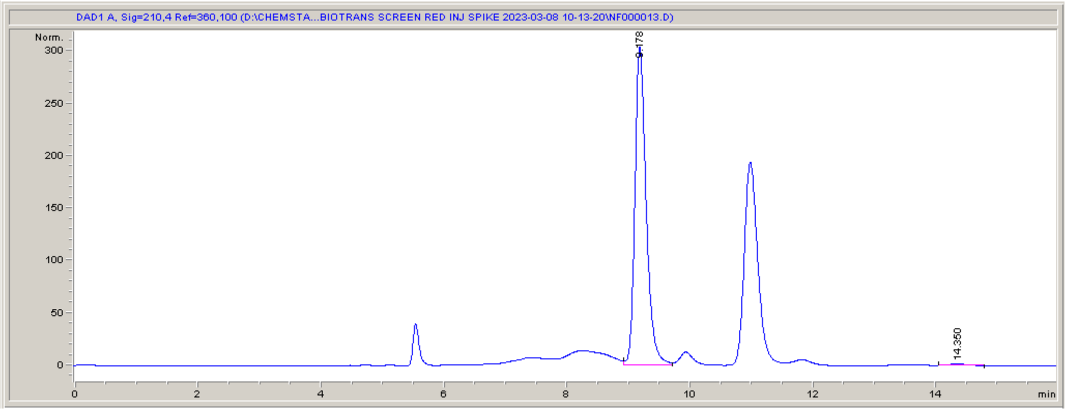

#	Time	Area	Height	Width	Area%	Symmetry
1	9.178	3881.2	303.8	0.193	99.342	0.714
2	14.35	25.7	1.4	0.2399	0.658	0.945

Chiral HPLC – biotransformation **1h**_YqjM:

(CHIRACEL® OJ-H; Heptane/i-PrOH 1/1; 0.7 mL/min; 25 °C)

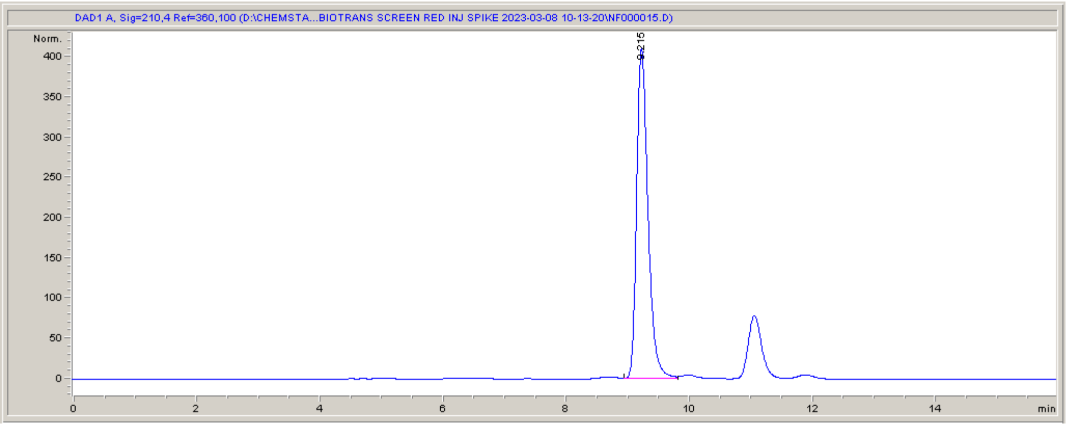

#	Time	Area	Height	Width	Area%	Symmetry
1	9.215	5259.1	410.8	0.1954	100.000	0.696

4.3.11 Desymmetrization of 1i

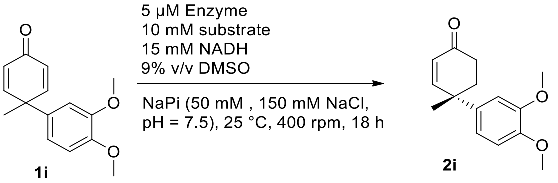

Enzyme	HPLC yield – **2i** (%)	HPLC recovery – **1i** (%)	ee – **2i** (%)
OPR3	63	42	>99
YqjM	97	2	>99

Calibration – ***rac*-2i:**
(Heptane/i-PrOH 1/1; 0.7 mL/min; 25 °C)

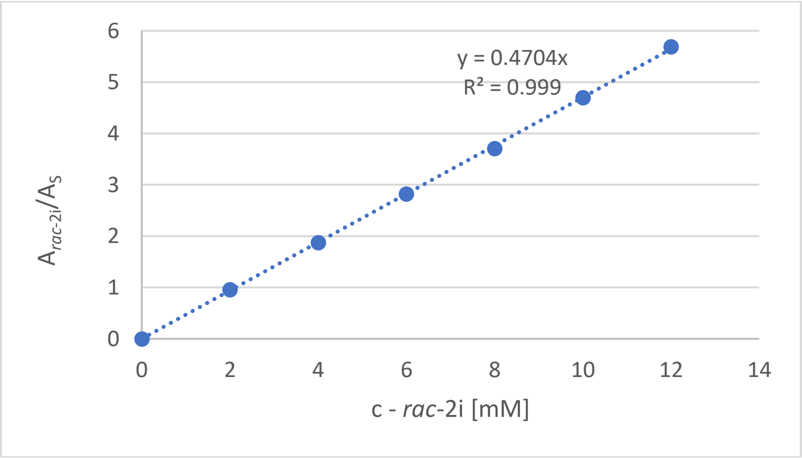

Calibration – **1i:**
(Heptane/i-PrOH 1/1; 0.7 mL/min; 25 °C)

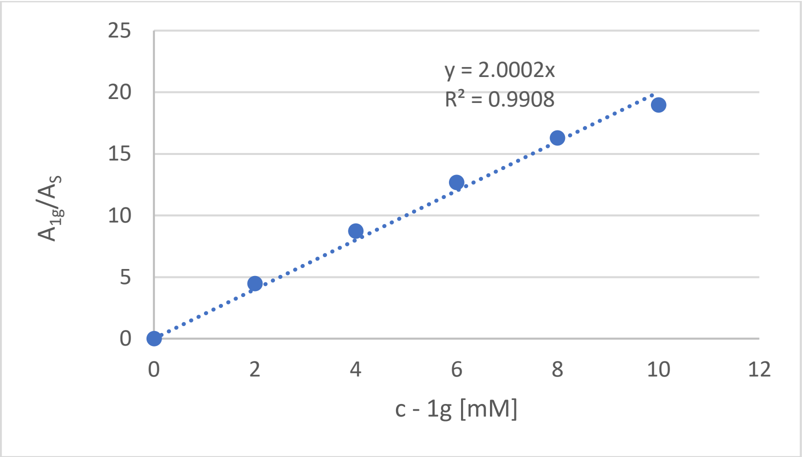

Chiral HPLC – ***rac*-2i:**
(CHIRACEL® OJ-H; Heptane/i-PrOH 1/1; 0.7 mL/min; 25 °C)

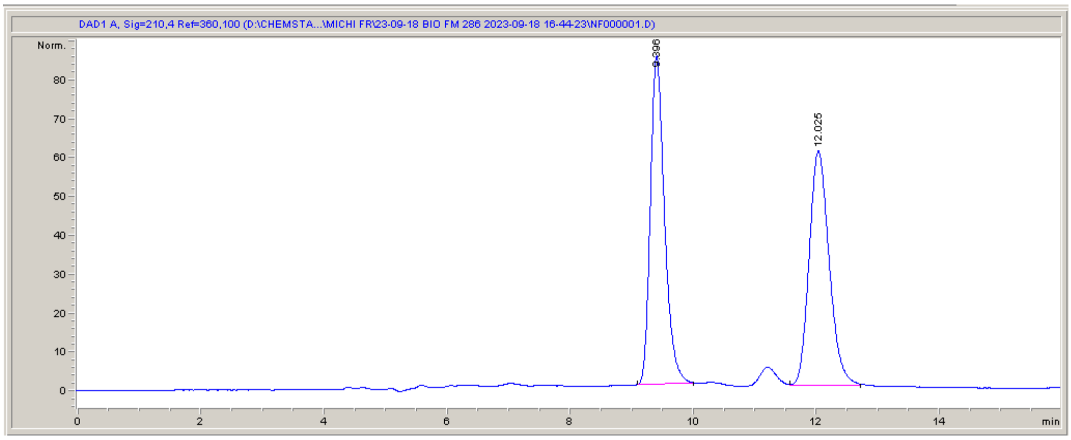

#	Time	Area	Height	Width	Area%	Symmetry
1	9.396	1359	84.6	0.2455	49.897	0.726
2	12.025	1364.6	60.3	0.3499	50.103	0.806

Chiral HPLC – biotransformation **1i**_OPR3:

(CHIRACEL® OJ-H; Heptane/i-PrOH 1/1; 0.7 mL/min; 25 °C)

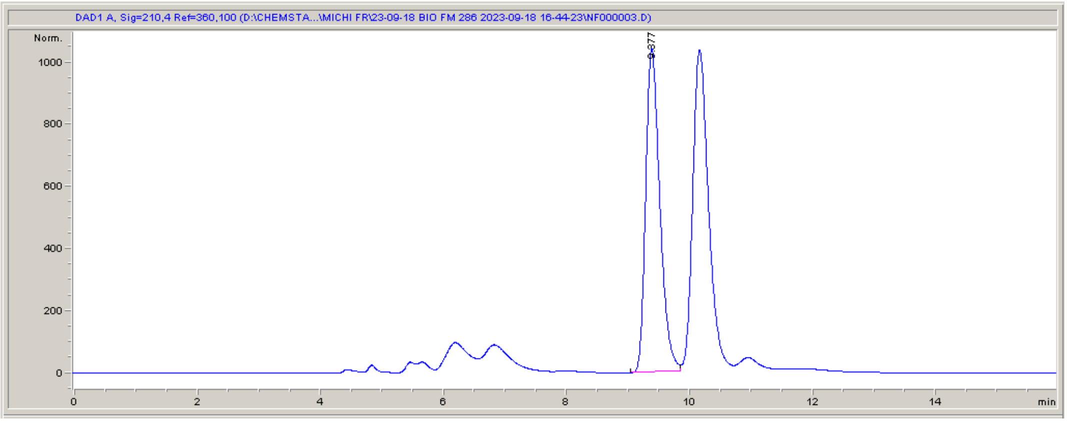

#	Time	Area	Height	Width	Area%	Symmetry
1	9.377	16763.3	1043.6	0.2454	100.000	0.687

Chiral HPLC – biotransformation **1i**_YqjM:

(CHIRACEL® OJ-H; Heptane/i-PrOH 1/1; 0.7 mL/min; 25 °C)

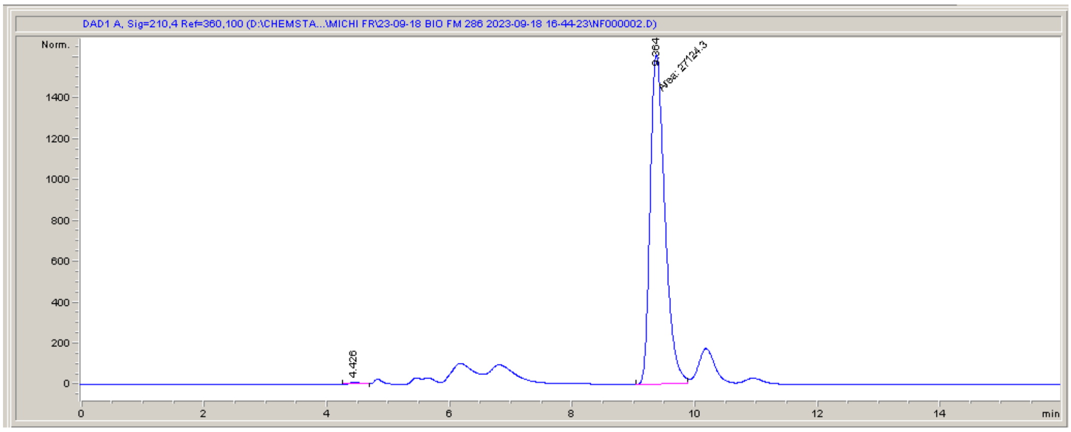

#	Time	Area	Height	Width	Area%	Symmetry
1	4.426	147.2	11.6	0.1804	0.540	0.434
2	9.364	27124.3	1615.8	0.2798	99.460	0.671

4.3.12 Desymmetrization of 1j

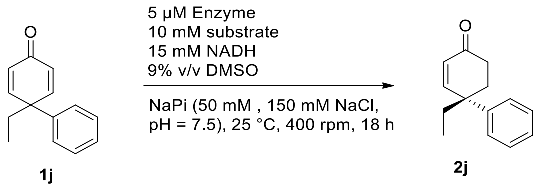

Enzyme	HPLC yield – **2j** (%)	HPLC recovery – **1j** (%)	ee – **2j** (%)
OPR3	48	20	98
YqjM	54	12	>99

Calibration – ***rac*-2j:**

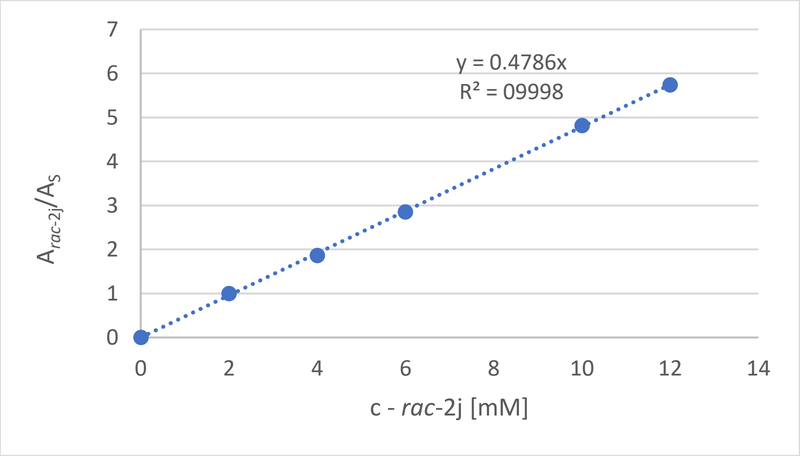

Calibration – **1j:**

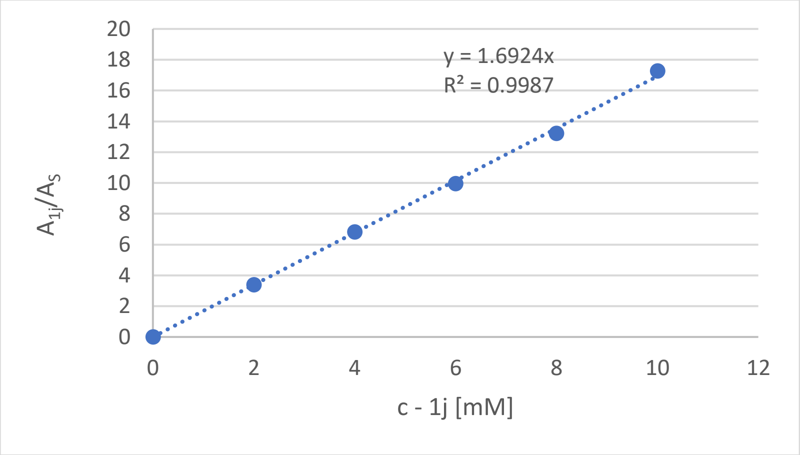

Chiral HPLC – ***rac*-2j:**
(CHIRACEL® OJ-H; Heptane/EtOH 8/1; 0.7 mL/min; 25 °C)

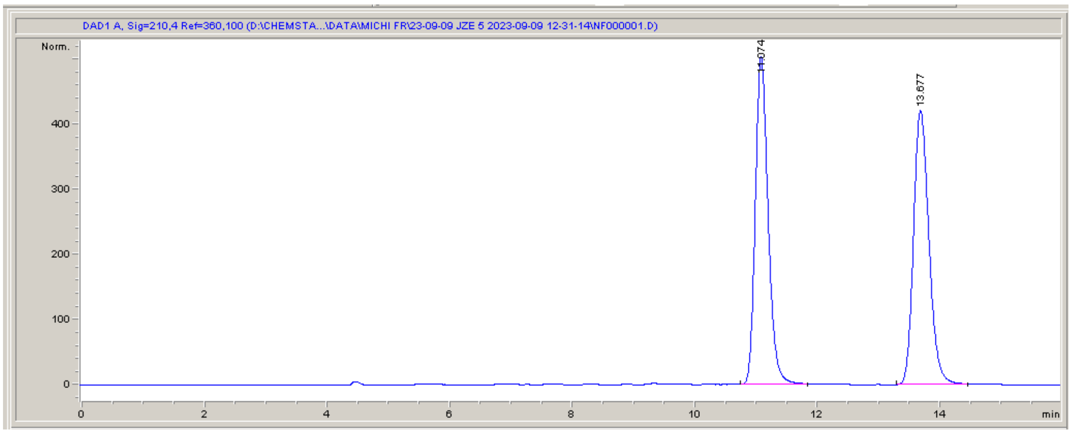

#	Time	Area	Height	Width	Area%	Symmetry
1	11.074	7333.1	503.8	0.2258	50.008	0.77
2	13.677	7330.8	420.8	0.2692	49.992	0.785

Chiral HPLC – biotransformation **1j**_OPR3:

(CHIRACEL® OJ-H; Heptane/EtOH 8/1; 0.7 mL/min; 25 °C)

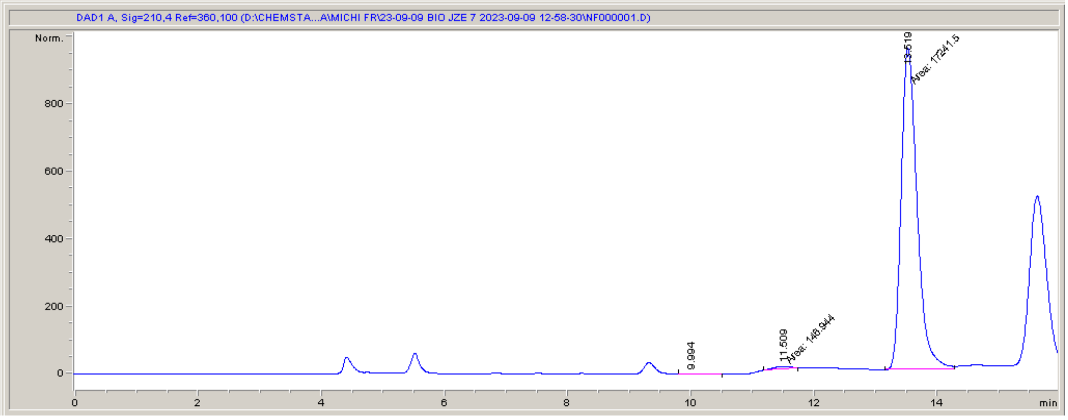

#	Time	Area	Height	Width	Area%	Symmetry
1	9.994	18.4	1.2	0.2135	0.106	0.831
2	11.509	146.9	7.5	0.3258	0.844	1.808
3	13.519	17241.5	953.9	0.3012	99.050	0.704

Chiral HPLC – biotransformation **1j**_YqjM:

(CHIRACEL® OJ-H; Heptane/EtOH 8/1; 0.7 mL/min; 25 °C)

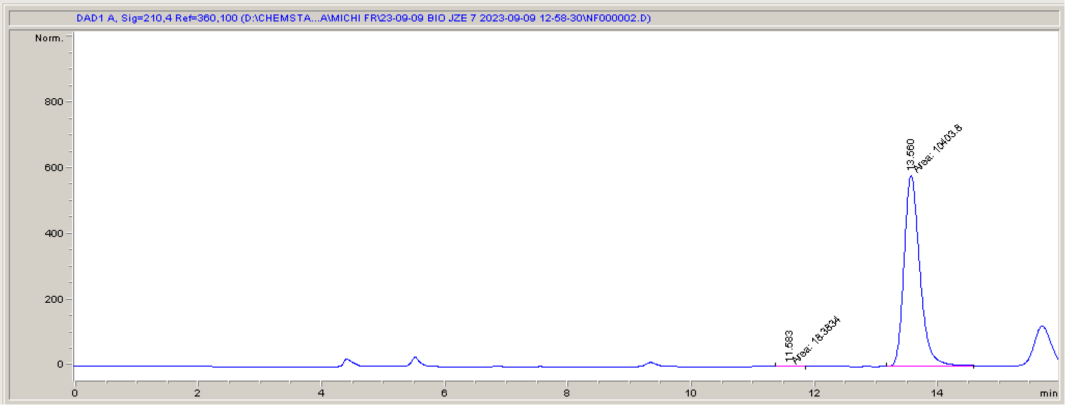

#	Time	Area	Height	Width	Area%	Symmetry
1	11.583	18.4	1.2	0.253	0.176	0.619
2	13.56	10403.8	581.6	0.2981	99.824	0.747

4.3.13 Desymmetrization of 1k

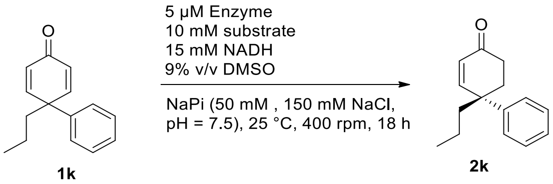

Enzyme	HPLC yield – **2k** (%)	HPLC recovery – **1k** (%)	ee – **2k** (%)
OPR3	11	59	60
YqjM	19	44	86

Calibration – ***rac*-2k:**

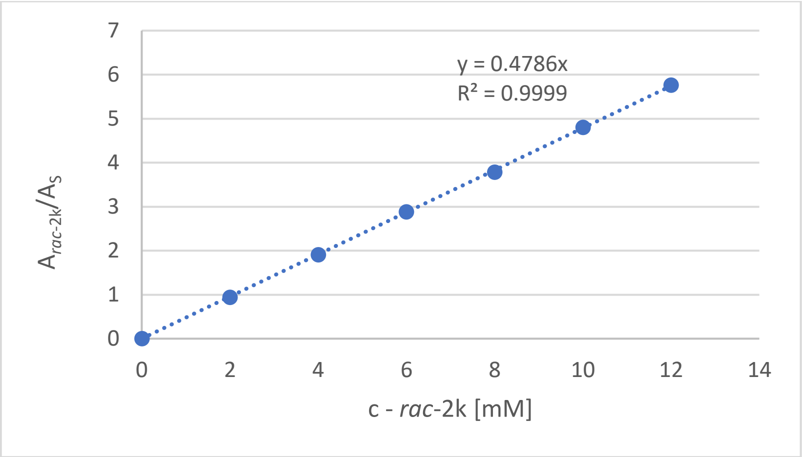

Calibration – **1k:**

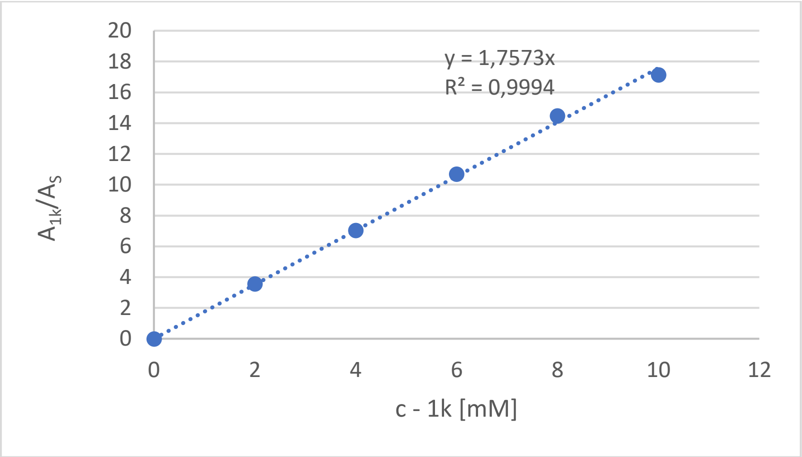

Chiral HPLC – ***rac*-2k:**
(CHIRACEL® OJ-H; Heptane/i-PrOH 8/1; 0.7 mL/min; 25 °C)

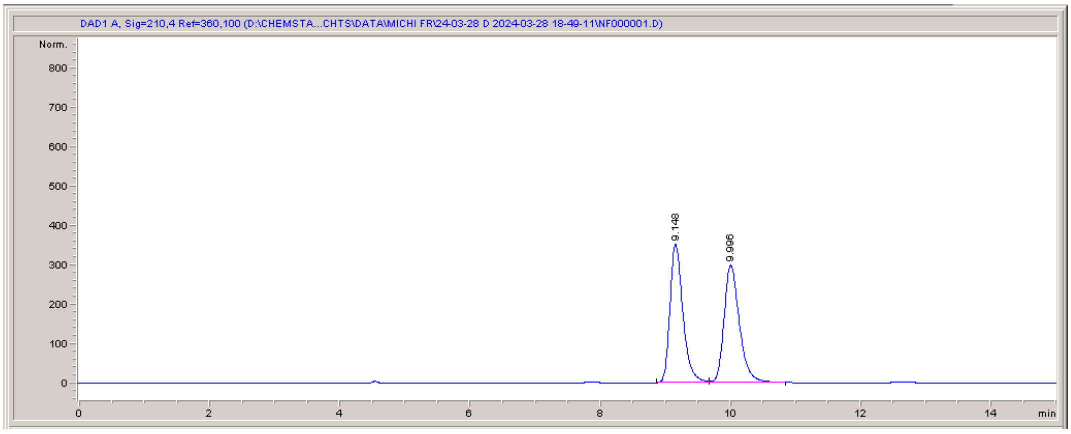

#	Time	Area	Height	Width	Area%	Symmetry
1	9.148	4887.2	351.4	0.2123	49.834	0.7
2	9.996	4919.8	298.1	0.2505	50.166	0.718

Chiral HPLC – biotransformation **1k**_OPR3:

(CHIRACEL® OJ-H; Heptane/i-PrOH 8/1; 0.7 mL/min; 25 °C)

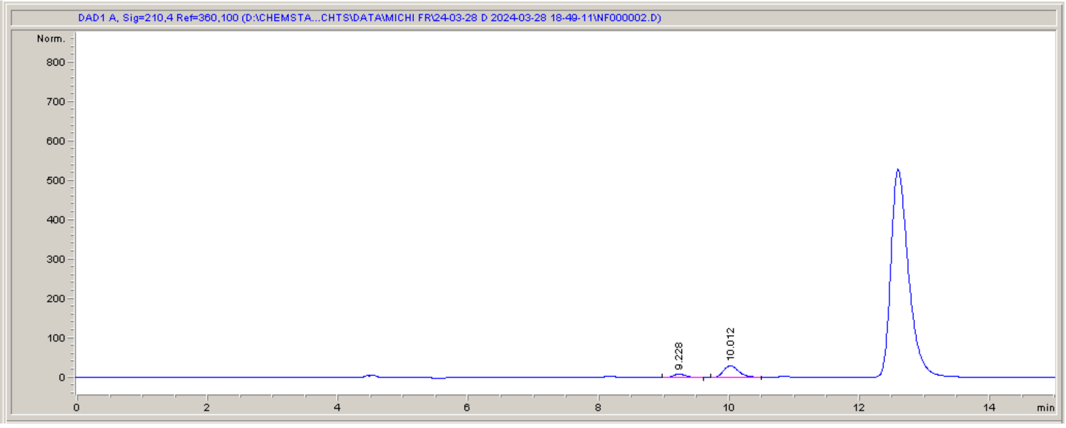

#	Time	Area	Height	Width	Area%	Symmetry
1	9.228	124.5	8.9	0.2113	19.840	0.823
2	10.012	503.1	30.4	0.2511	80.160	0.749

Chiral HPLC – biotransformation **1k**_YqjM:

(CHIRACEL® OJ-H; Heptane/i-PrOH 8/1; 0.7 mL/min; 25 °C)

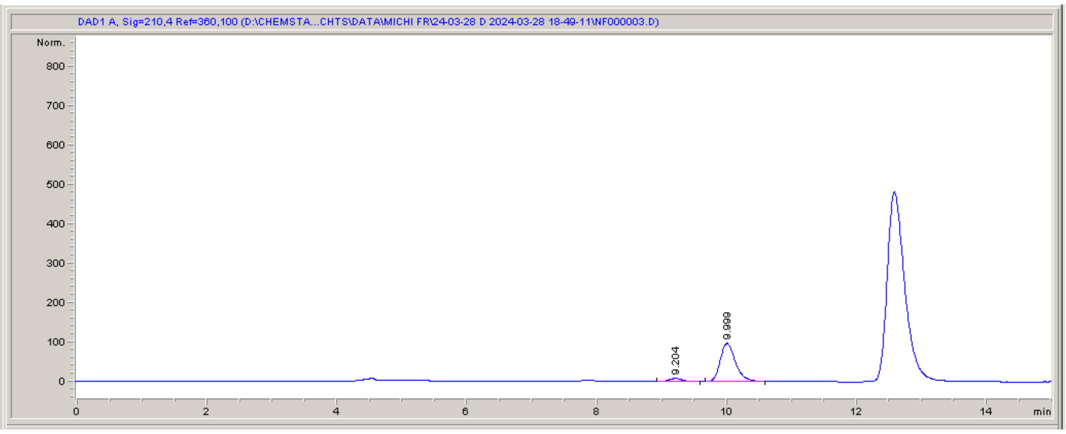

#	Time	Area	Height	Width	Area%	Symmetry
1	9.204	116	8	0.2257	6.794	0.852
2	9.999	1591.9	97.3	0.2489	93.206	0.747

4.3.14 Desymmetrization of 1l

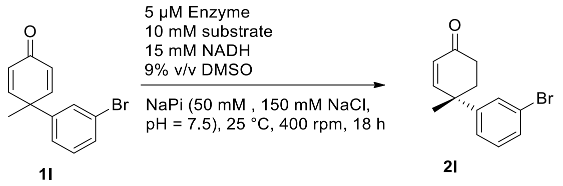

Enzyme	HPLC yield – **2l** (%)	HPLC recovery – **1l** (%)	ee – **2l** (%)
OPR3	44	8	>99
YqjM	40	4	>99

Calibration – ***rac*-2l:**

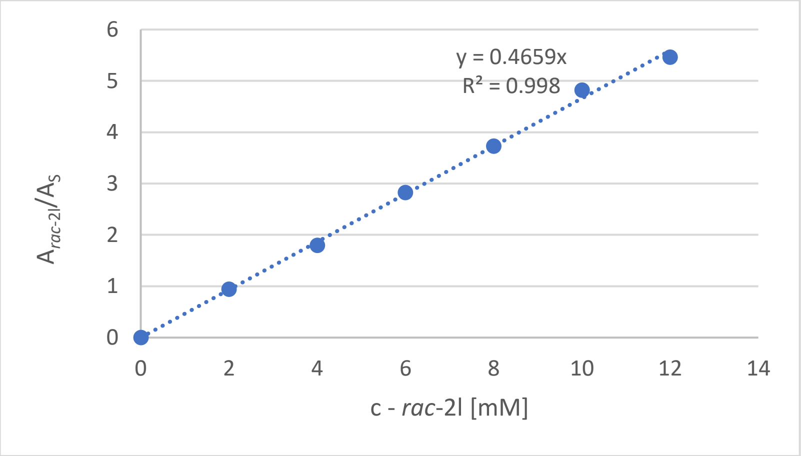

Calibration – **1l:**

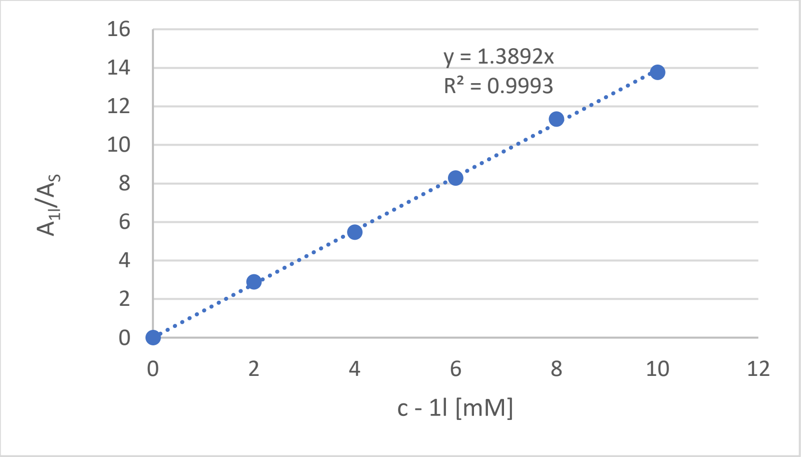

Chiral HPLC – ***rac*-2l:**
(CHIRACEL® OJ-H; Heptane/i-PrOH 1/1; 0.7 mL/min; 25 °C)

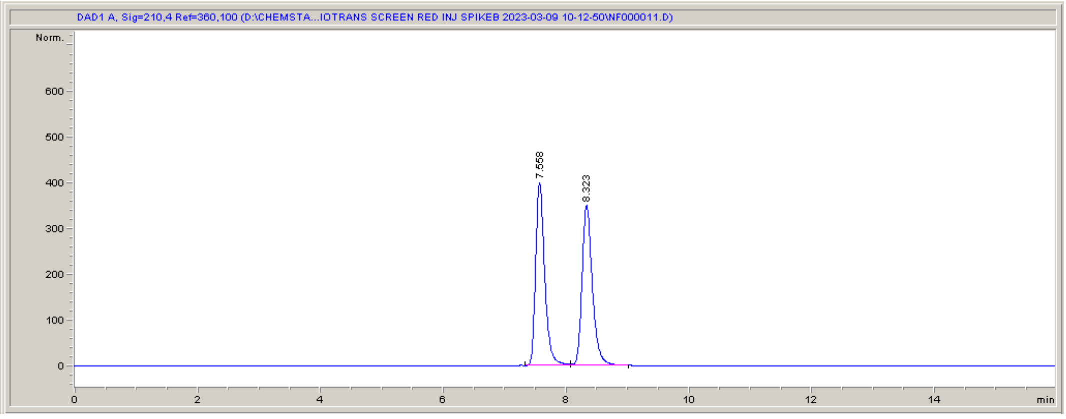

#	Time	Area	Height	Width	Area%	Symmetry
1	7.558	4107	399.8	0.1538	49.870	0.699
2	8.323	4128.3	349.9	0.1796	50.130	0.719

Chiral HPLC – biotransformation **1l**_OPR3:

(CHIRACEL® OJ-H; Heptane/i-PrOH 1/1; 0.7 mL/min; 25 °C)

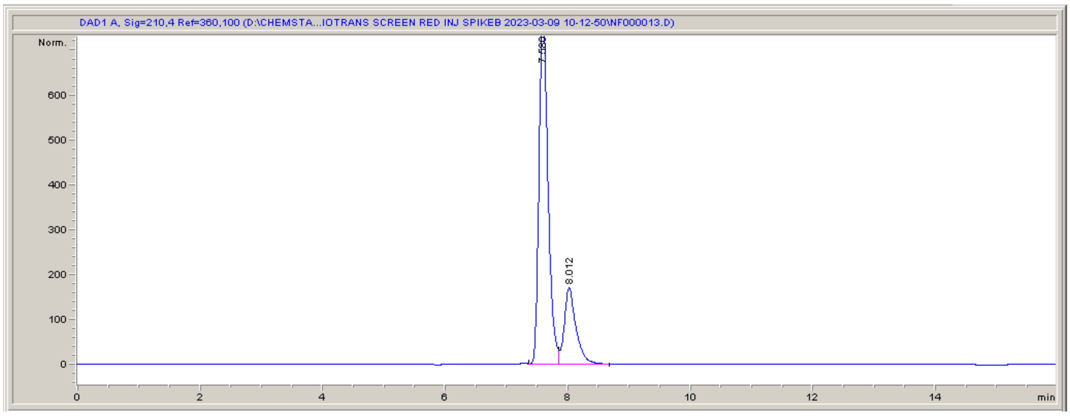

#	Time	Area	Height	Width	Area%	Symmetry
1	7.586	7984.3	786	0.1545	77.925	0.713
2	8.012	2261.8	170.3	0.193	22.075	0.616

Chiral HPLC – biotransformation **1l**_YqjM:

(CHIRACEL® OJ-H; Heptane/i-PrOH 1/1; 0.7 mL/min; 25 °C)

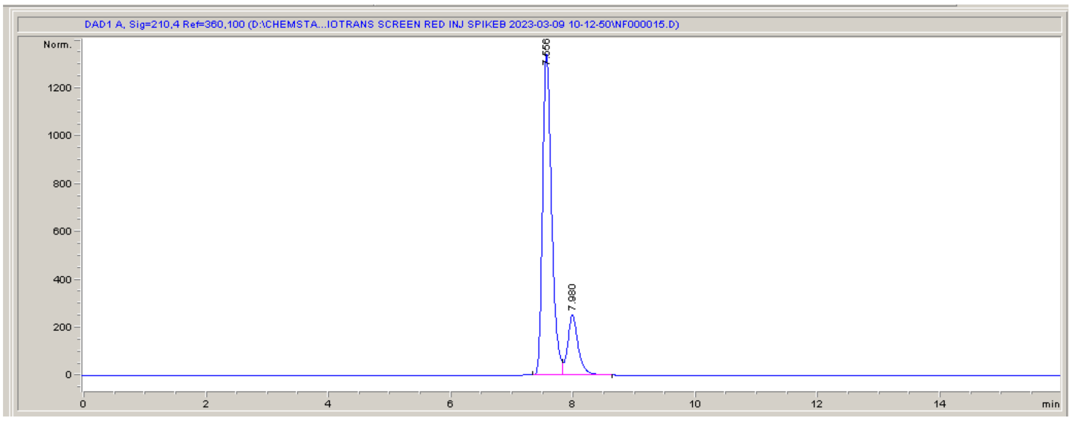

#	Time	Area	Height	Width	Area%	Symmetry
1	7.556	13799.2	1342	0.156	81.982	0.698
2	7.98	3032.7	254	0.1773	18.018	0.727

4.3.15 Desymmetrization of 1m

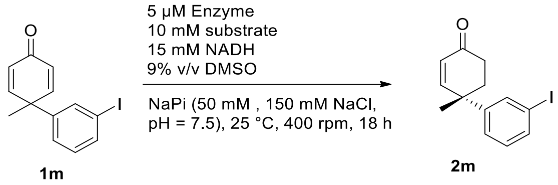

Enzyme	HPLC yield – **2m** (%)	HPLC recovery – **1m** (%)	ee – **2m** (%)
OPR3	44	21	>99
YqjM	15	7	>99

Calibration – ***rac*-2m:**

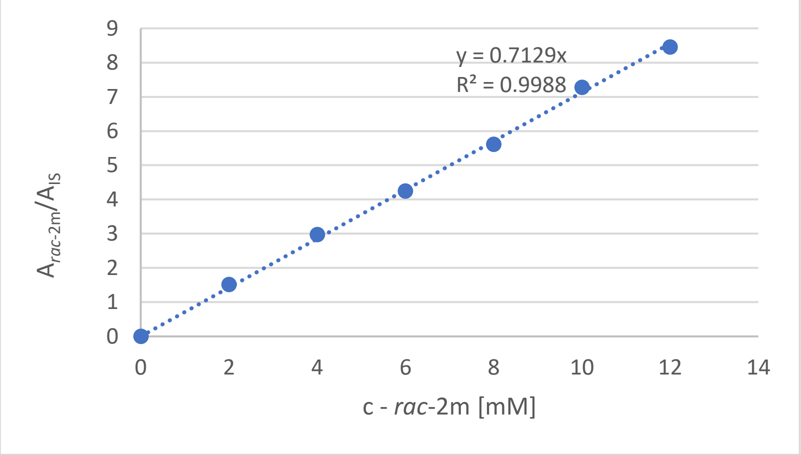

Calibration – **1m:**

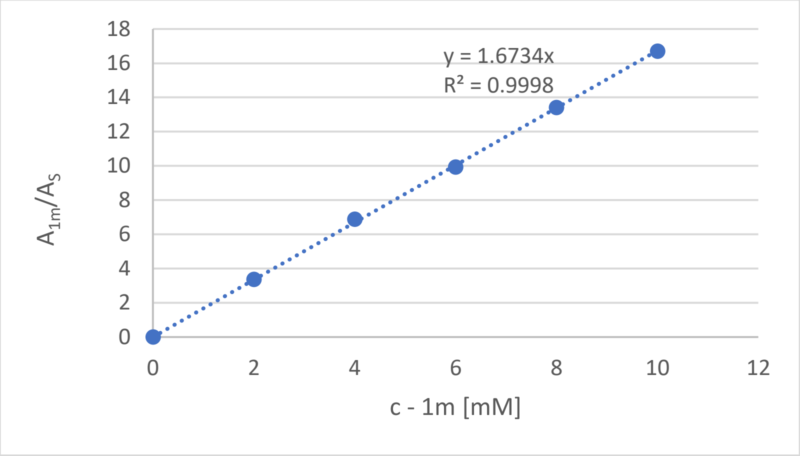

Chiral HPLC – ***rac*-2m:**
(CHIRACEL® OJ-H; Heptane/i-PrOH 4/1; 0.7 mL/min; 25 °C)

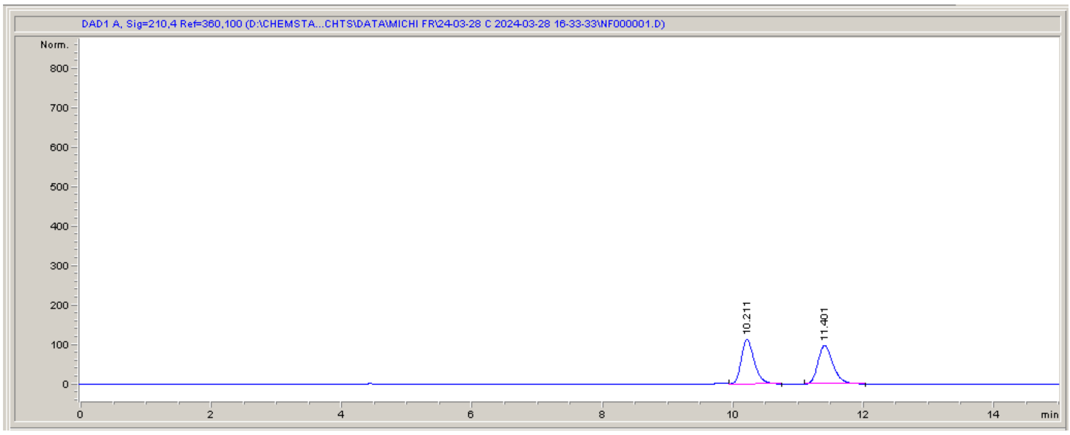

#	Time	Area	Height	Width	Area%	Symmetry
1	10.211	1574.4	113.4	0.212	49.900	0.751
2	11.401	1580.7	97.4	0.2494	50.100	0.736

Chiral HPLC – biotransformation **1m**_OPR3:

(CHIRACEL® OJ-H; Heptane/i-PrOH 4/1; 0.7 mL/min; 25 °C)

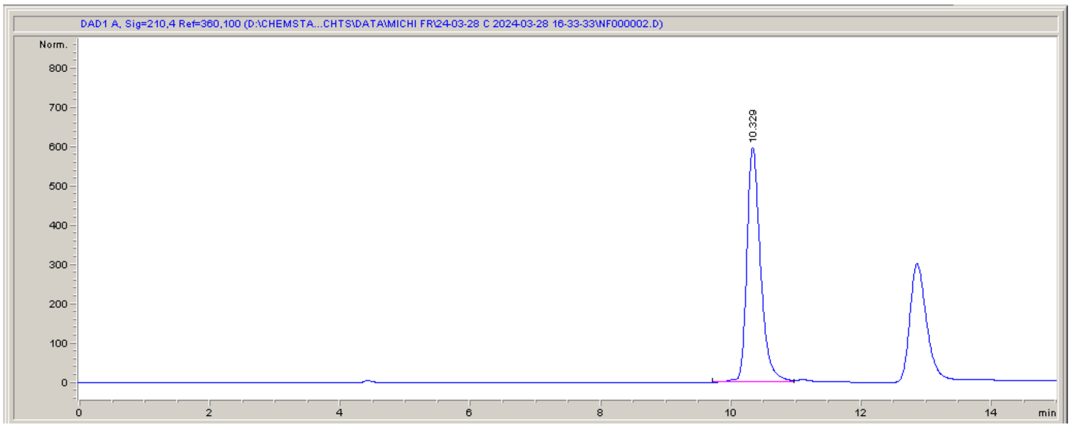

#	Time	Area	Height	Width	Area%	Symmetry
1	10.329	8742.2	598.3	0.2225	100.000	0.696

Chiral HPLC – biotransformation **1m**_YqjM:

(CHIRACEL® OJ-H; Heptane/i-PrOH 4/1; 0.7 mL/min; 25 °C)

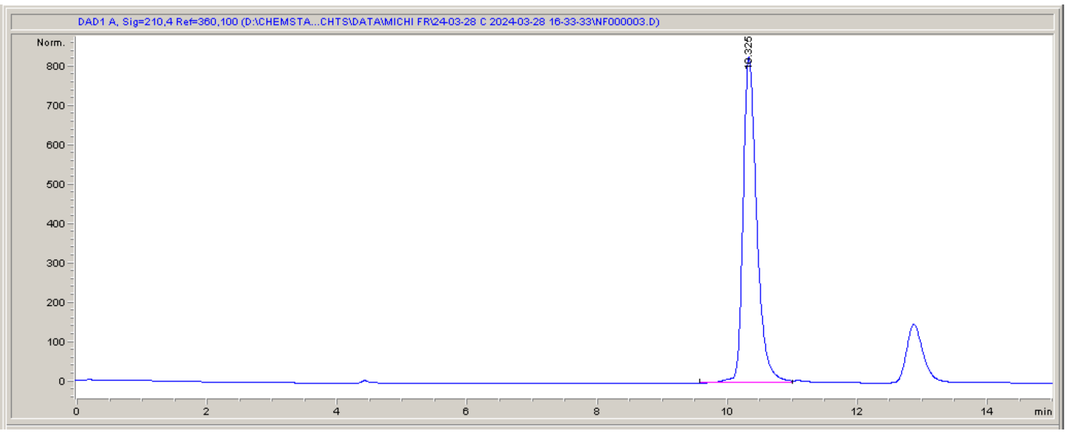

#	Time	Area	Height	Width	Area%	Symmetry
1	10.325	12182.3	827.6	0.2237	100.000	0.69

4.3.16 Desymmetrization of 1n

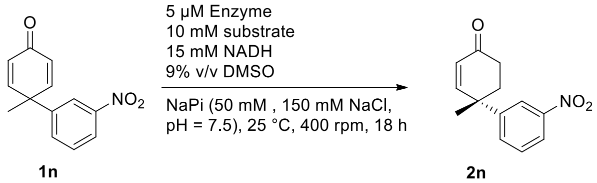

Enzyme	HPLC yield – **2n** (%)	HPLC recovery – **1n** (%)	ee – **2n** (%)
OPR3	48	35	75
YqjM	32	66	79

Calibration – **rac-2n:**

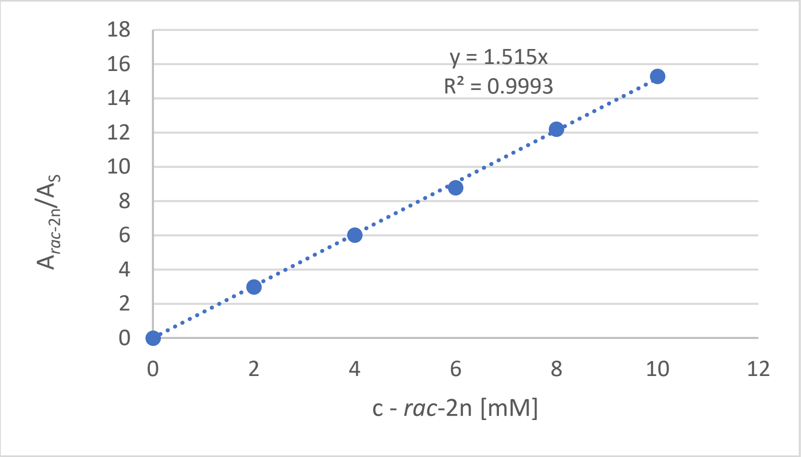

Calibration – **1n:**

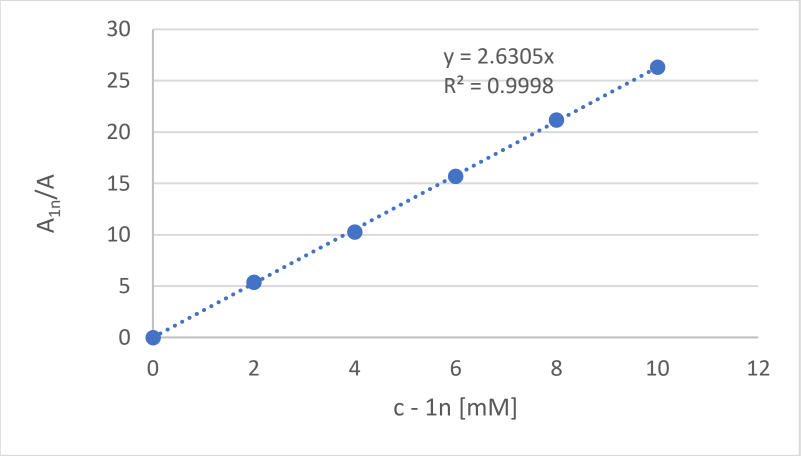

Chiral HPLC – ***rac*-2n:**
(CHIRACEL® OJ-H; Heptane/EtOH 4/1; 0.7 mL/min; 25 °C)

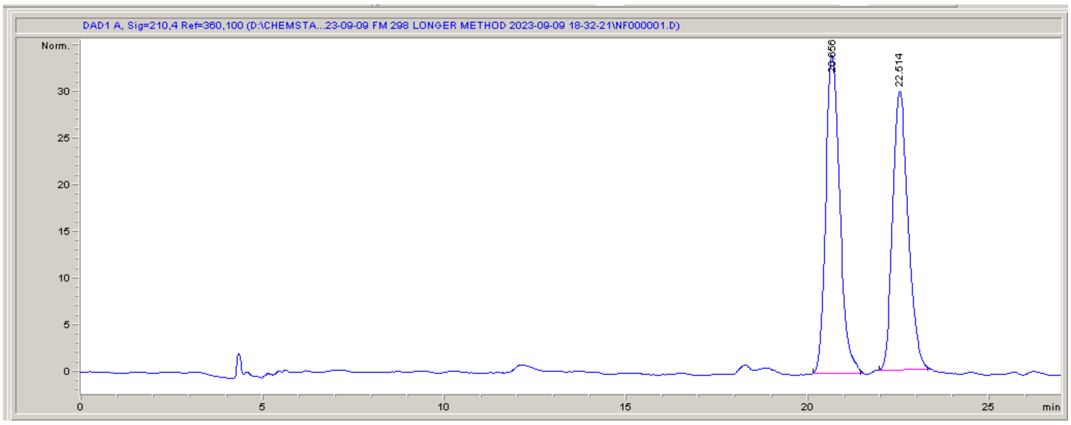

#	Time	Area	Height	Width	Area%	Symmetry
1	20.656	921.8	34.1	0.4152	50.955	0.788
2	22.514	887.2	29.9	0.4621	49.045	0.77

Chiral HPLC – biotransformation **1n**_OPR3:

(CHIRACEL® OJ-H; Heptane/EtOH 4/1; 0.7 mL/min; 25 °C)

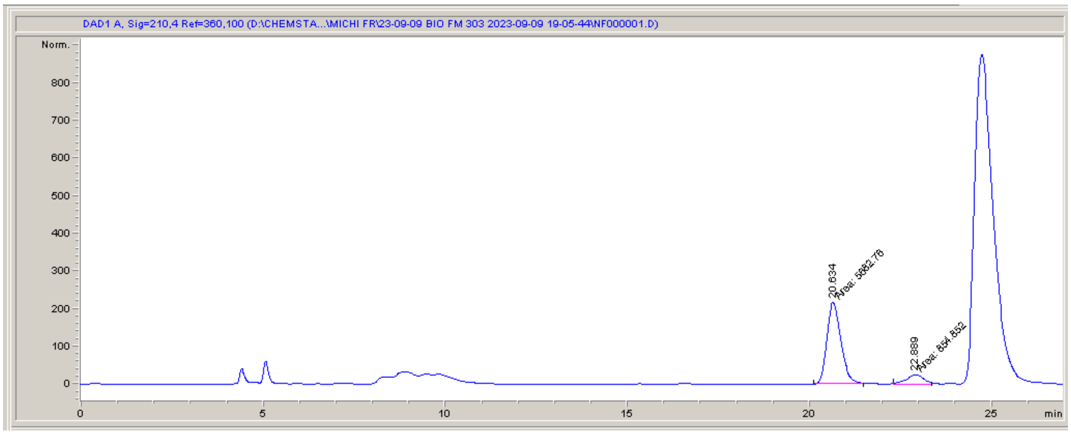

#	Time	Area	Height	Width	Area%	Symmetry
1	20.634	5682.8	215.5	0.4396	86.924	0.772
2	22.889	854.9	25.3	0.5627	13.076	1.1

Chiral HPLC – biotransformation **1n**_YqjM:

(CHIRACEL® OJ-H; Heptane/EtOH 4/1; 0.7 mL/min; 25 °C)

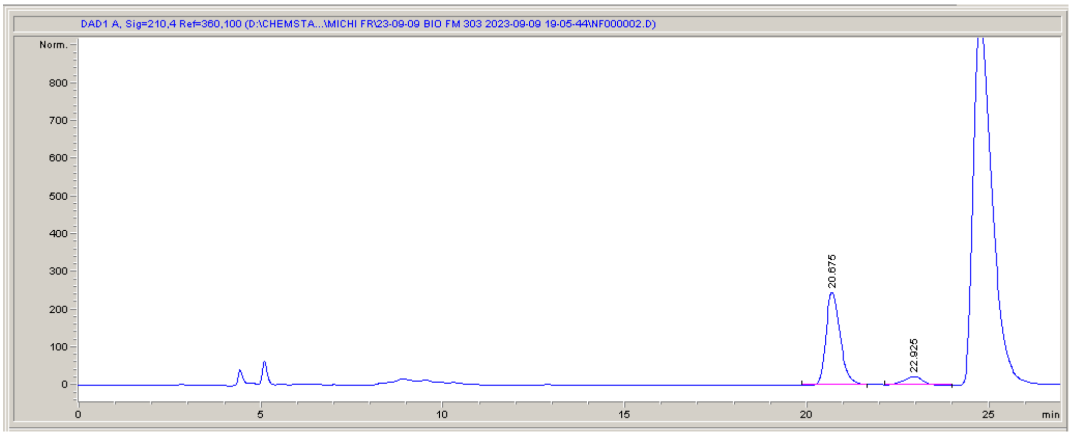

#	Time	Area	Height	Width	Area%	Symmetry
1	20.675	6624.2	245.8	0.4185	89.477	0.732
2	22.925	779	22.5	0.5145	10.523	1.272

4.3.17 Desymmetrization of 1o

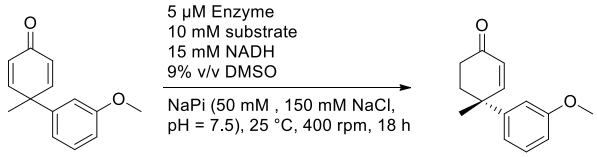

1o **2o**

Enzyme	HPLC yield – **2o** (%)	HPLC recovery – **1o** (%)	ee – **2o** (%)
OPR3	53	12	>99
YqjM	60	8	>99

Calibration – ***rac*-2o:**

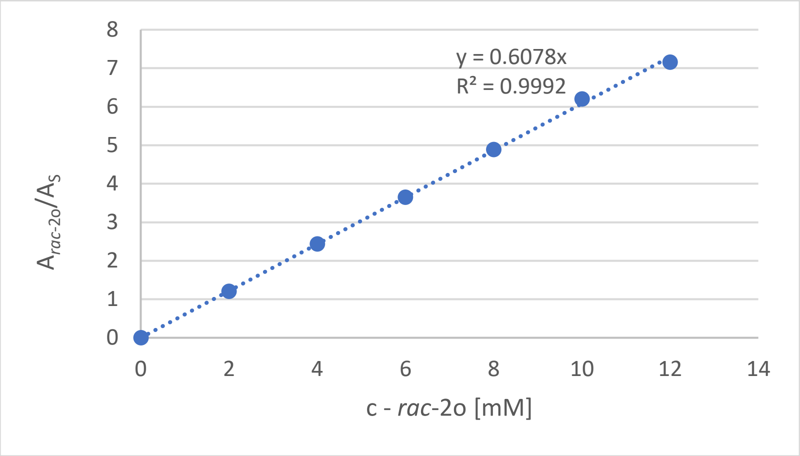

Calibration – **1o:**

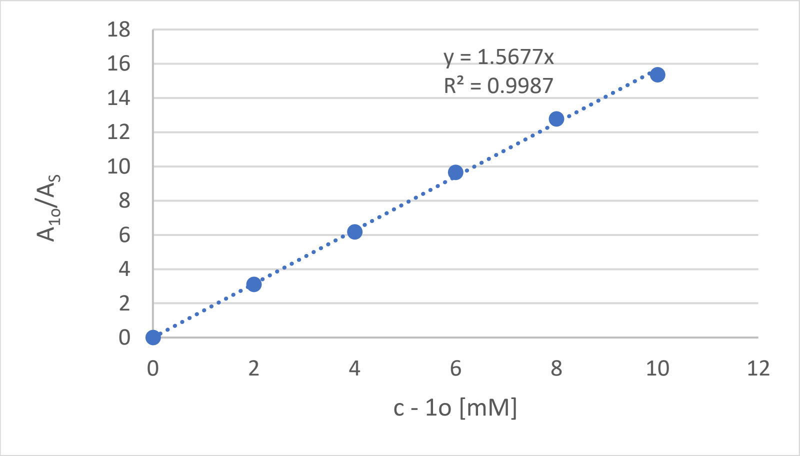

Chiral HPLC – ***rac*-2o**
(CHIRACEL® OJ-H; Heptane/i-PrOH 4/1; 0.7 mL/min)

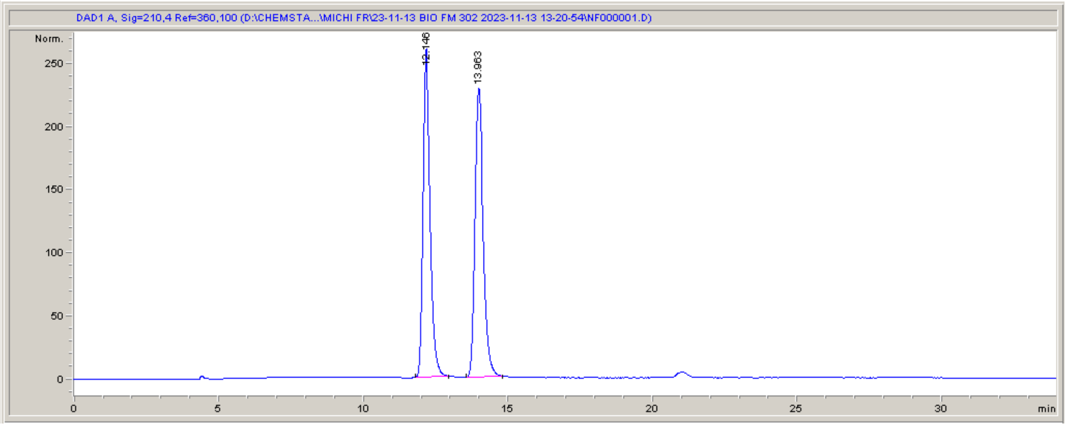

#	Time	Area	Height	Width	Area%	Symmetry
1	12.146	4539.2	260.3	0.2675	50.052	0.72
2	13.963	4529.7	228.4	0.3033	49.948	0.717

Chiral HPLC – biotransformation **1o**_OPR3:

(CHIRACEL® OJ-H; Heptane/i-PrOH 4/1; 0.7 mL/min)

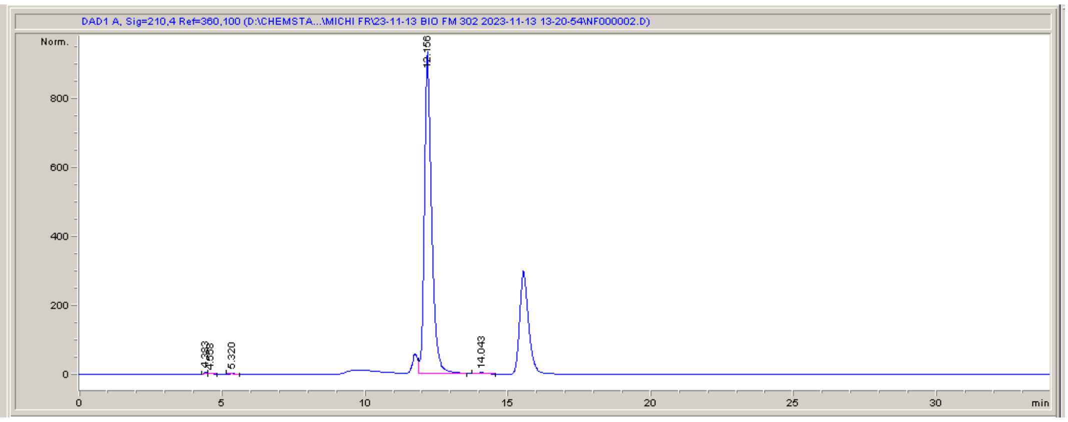

#	Time	Area	Height	Width	Area%	Symmetry
1	4.383	50.6	6.6	0.1173	0.292	0.615
2	4.558	28.1	2.8	0.139	0.162	0.435
3	5.32	31.1	3.1	0.1558	0.180	0.668
4	12.156	17113.3	934.9	0.2775	98.988	0.648
5	14.043	65.2	3.5	0.2855	0.377	0.757

Chiral HPLC – biotransformation **1o**_YqjM:

(CHIRACEL® OJ-H; Heptane/i-PrOH 4/1; 0.7 mL/min)

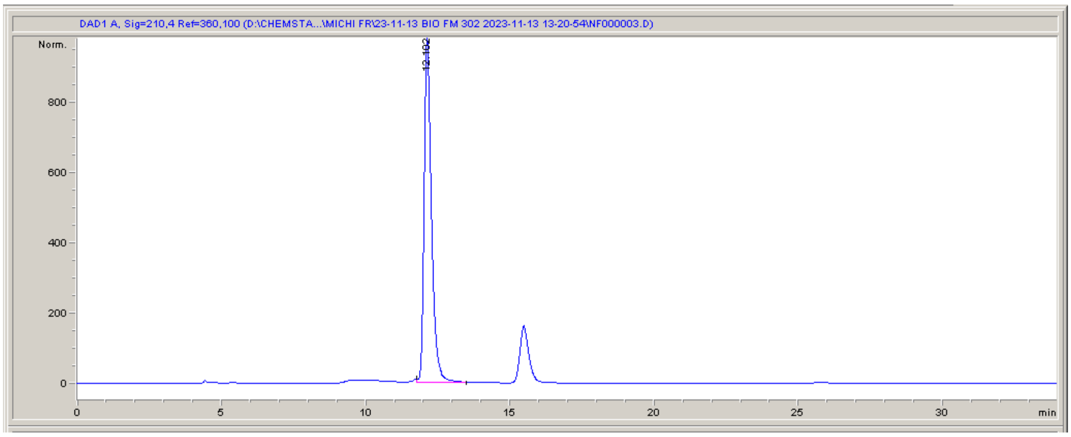

#	Time	Area	Height	Width	Area%	Symmetry
1	12.102	18322.7	1012.8	0.2771	100.000	0.635

4.3.18 Desymmetrization of 1p

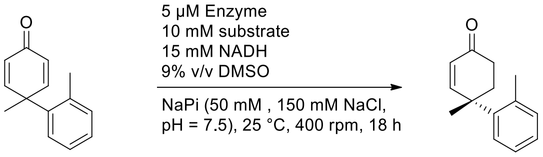

1p **2p**

Enzyme	HPLC yield – **2p** (%)	HPLC recovery – **1p** (%)	ee – **2p** (%)
OPR3	2	91	83
YqjM	8	88	94

Calibration – ***rac*-2p:**

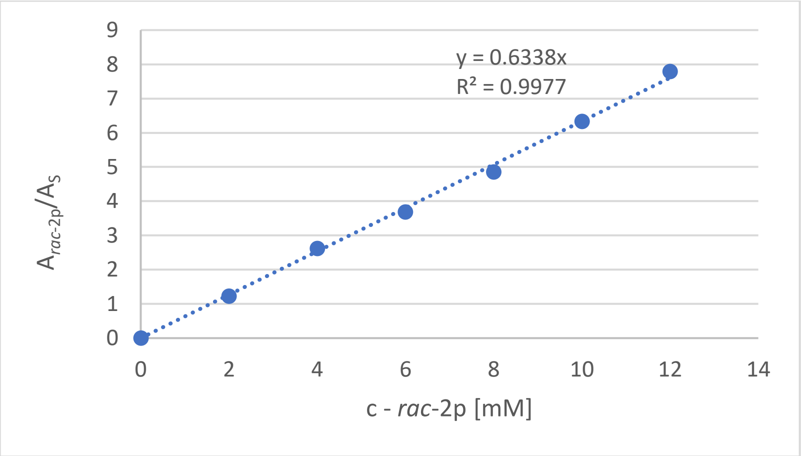

Calibration – **1p:**

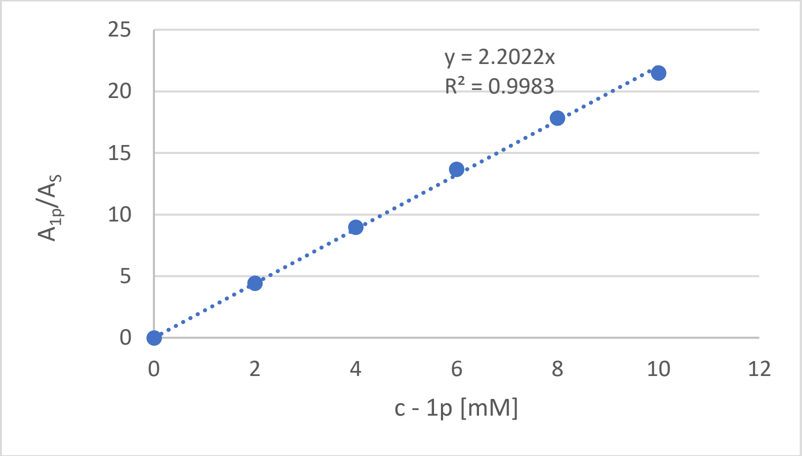

Chiral HPLC – ***rac*-2p:**
(CHIRACEL® OJ-H; Heptane/i-PrOH 1/1; 0.7 mL/min; 25 °C)

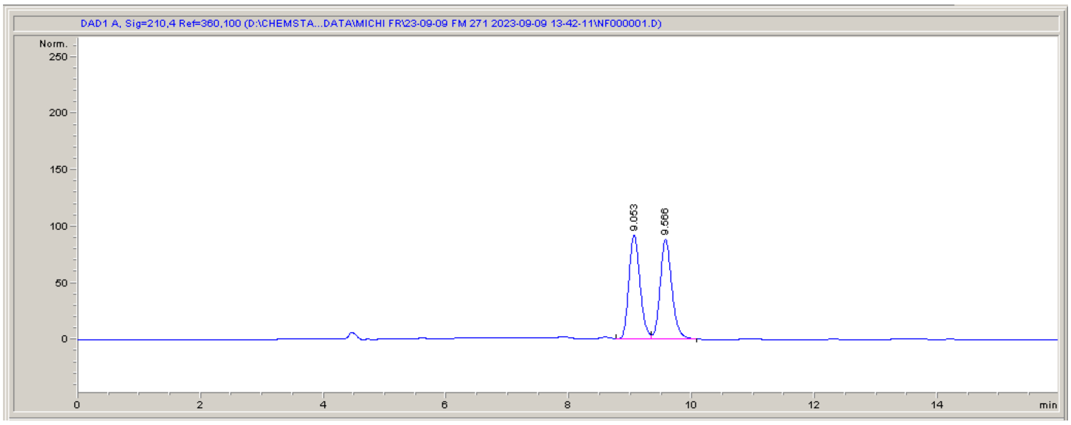

#	Time	Area	Height	Width	Area%	Symmetry
1	9.053	1151.6	91.7	0.1944	49.415	0.823
2	9.566	1178.9	88.5	0.2055	50.585	0.807

Chiral HPLC – biotransformation **1p**_OPR3:

(CHIRACEL® OJ-H; Heptane/i-PrOH 1/1; 0.7 mL/min; 25 °C)

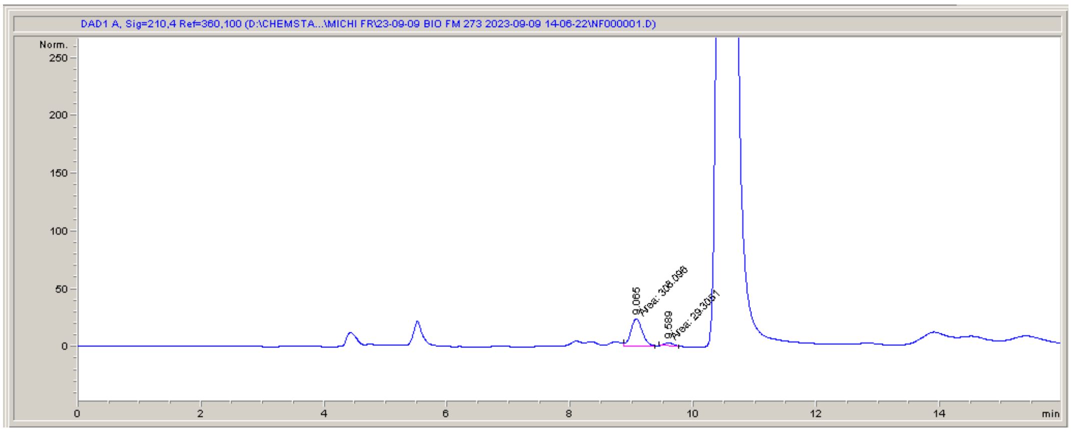

#	Time	Area	Height	Width	Area%	Symmetry
1	9.065	308.1	24	0.2143	91.314	0.876
2	9.589	29.3	2.7	0.1814	8.686	0.774

Chiral HPLC – biotransformation **1p**_YqjM:

(CHIRACEL® OJ-H; Heptane/i-PrOH 1/1; 0.7 mL/min; 25 °C)

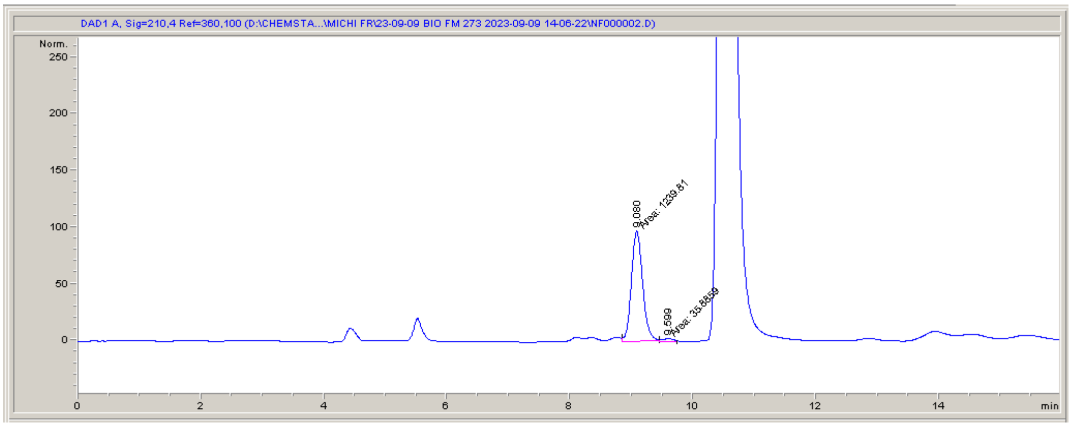

#	Time	Area	Height	Width	Area%	Symmetry
1	9.08	1239.8	97.6	0.2118	97.187	0.841
2	9.599	35.9	2.9	0.209	2.813	1.027

4.3.19 Desymmetrization of 1q

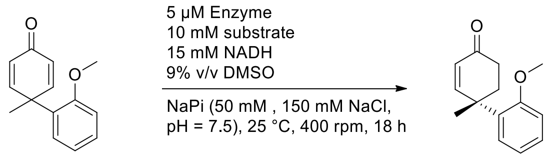

1q **2q**

Enzyme	HPLC yield – **2q** (%)	HPLC recovery – **1q** (%)	ee – **2q** (%)
OPR3	7	91	n.d.
YqjM	17	81	n.d.

Calibration – ***rac*-2q:**

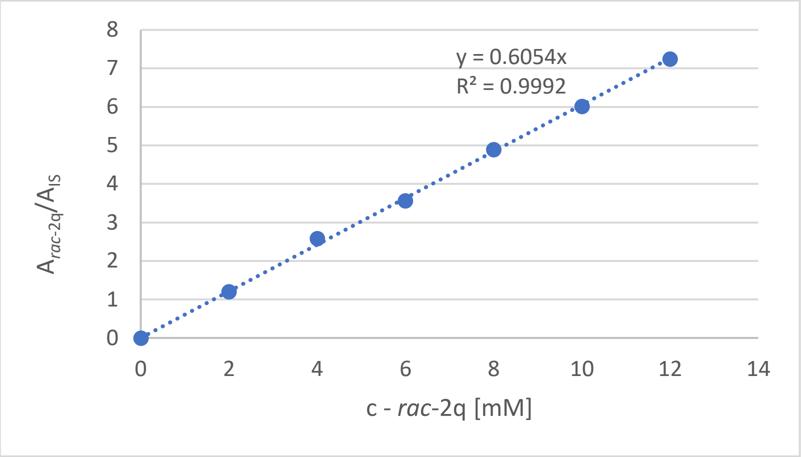

Calibration – **1q:**

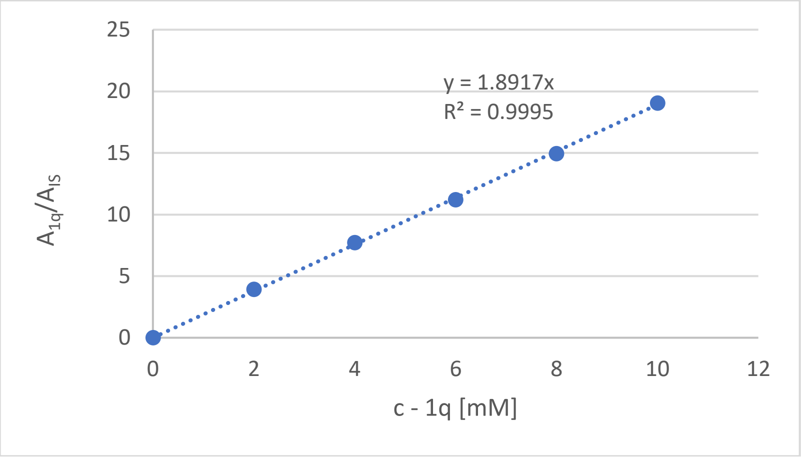

4.3.20 Desymmetrization of 1r

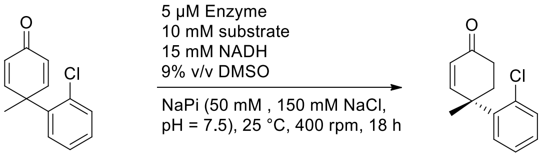

1r **2r**

Enzyme	HPLC yield – **2r** (%)	HPLC recovery – **1r** (%)	ee – **2r** (%)
OPR3	6	83	n.d.
YqjM	10	85	n.d.

Calibration – ***rac*-2r:**

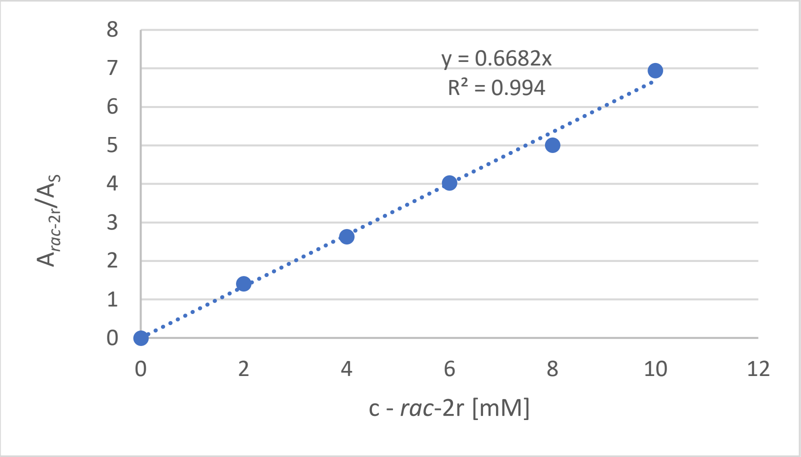

Calibration – **1r:**

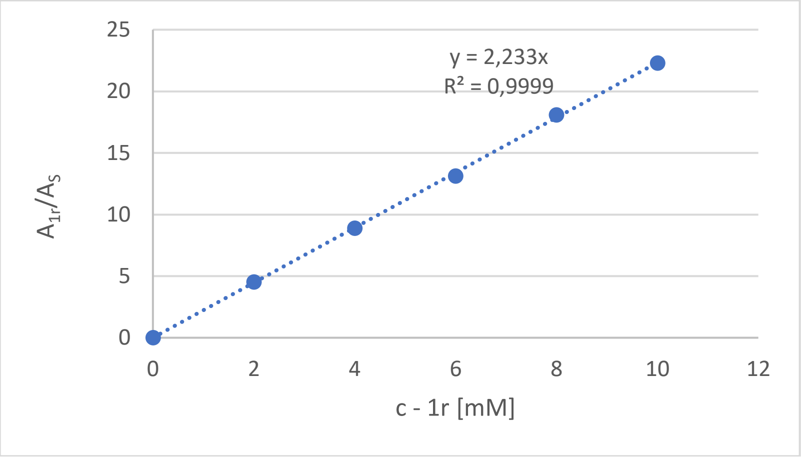

4.3.21 Desymmetrization of 1s

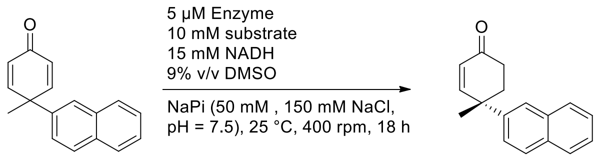

1s **2s**

Enzyme	GC convers. – **2s** (%)	HPLC recovery – **1s** (%)	ee – **2s** (%)
OPR3	<1%	/	n.d
YqjM	<1%	/	n.d

4.3.22 Desymmetrization of 1t

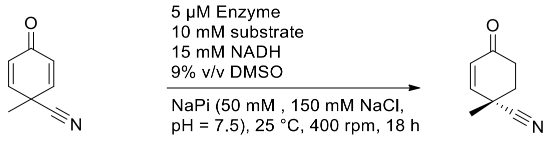

1t **2t**

Enzyme	HPLC yield – **2t** (%)	HPLC recovery – **1t** (%)	ee – **2t** (%)
OPR3	57	33	6
YqjM	94	16	85

Calibration – ***rac*-2t:**

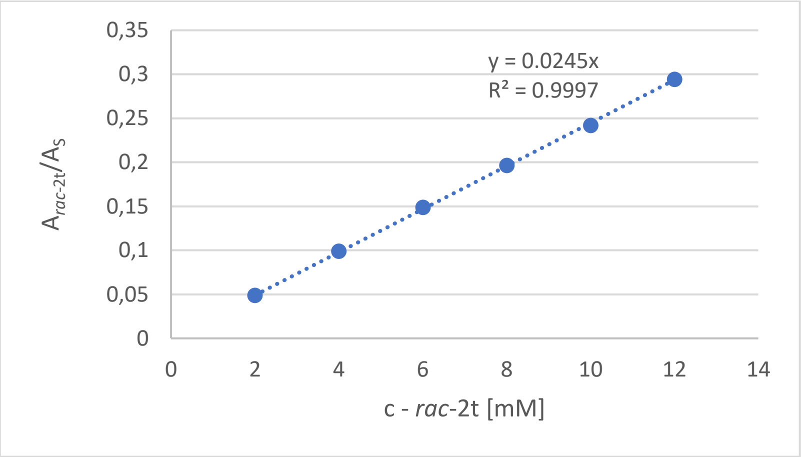

Calibration – **1t:**

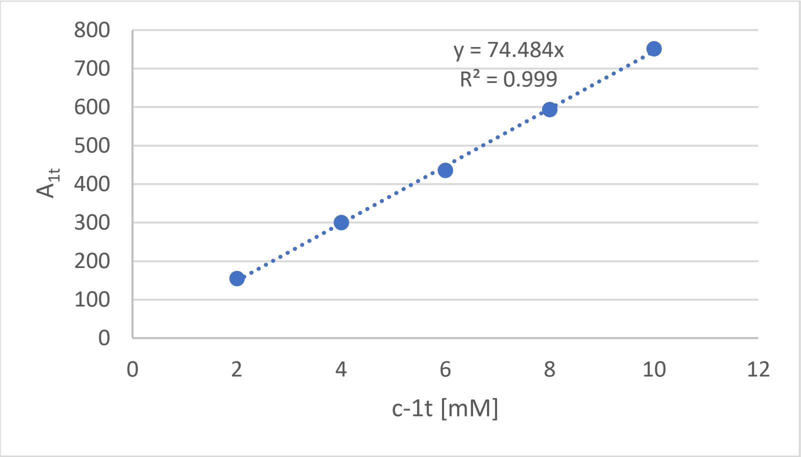

Chiral HPLC – ***rac*-2t:**
(CHIRACEL® OJ-H; Heptane/i-PrOH 4/1; 0.7 mL/min; 25 °C)

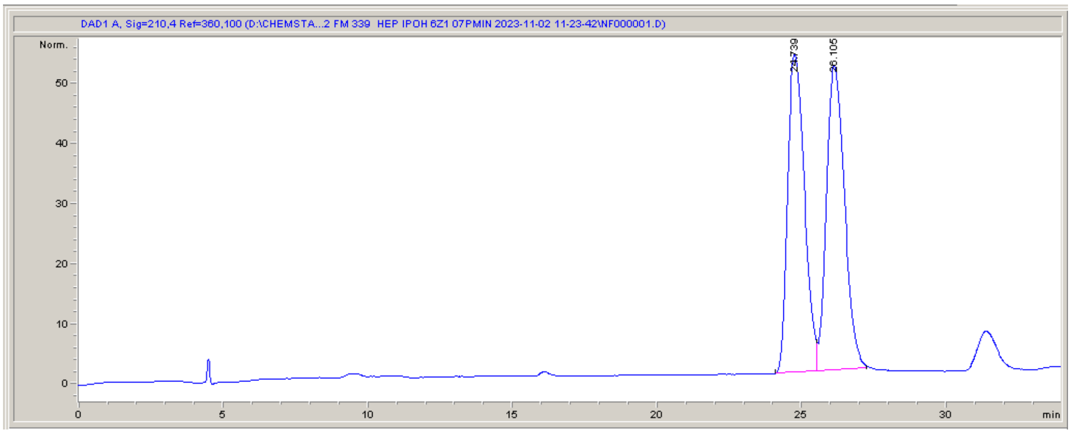

#	Time	Area	Height	Width	Area%	Symmetry
1	24.739	2147.2	52.8	0.6321	49.445	0.697
2	26.105	2195.4	50.7	0.6736	50.555	0.723

Chiral HPLC – biotransformation **1t**_OPR3:

(CHIRACEL® OJ-H; Heptane/i-PrOH 4/1; 0.7 mL/min; 25 °C)

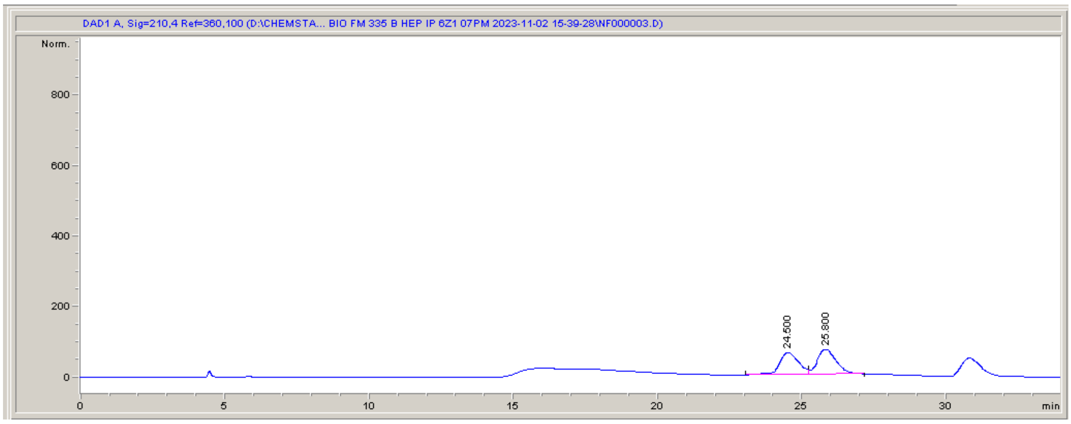

#	Time	Area	Height	Width	Area%	Symmetry
1	24.5	2912.6	62.5	0.7041	47.214	0.815
2	25.8	3256.4	70.7	0.7115	52.786	0.702

Chiral HPLC – biotransformation **1t**_YqjM:

(CHIRACEL® OJ-H; Heptane/i-PrOH 4/1; 0.7 mL/min; 25 °C)

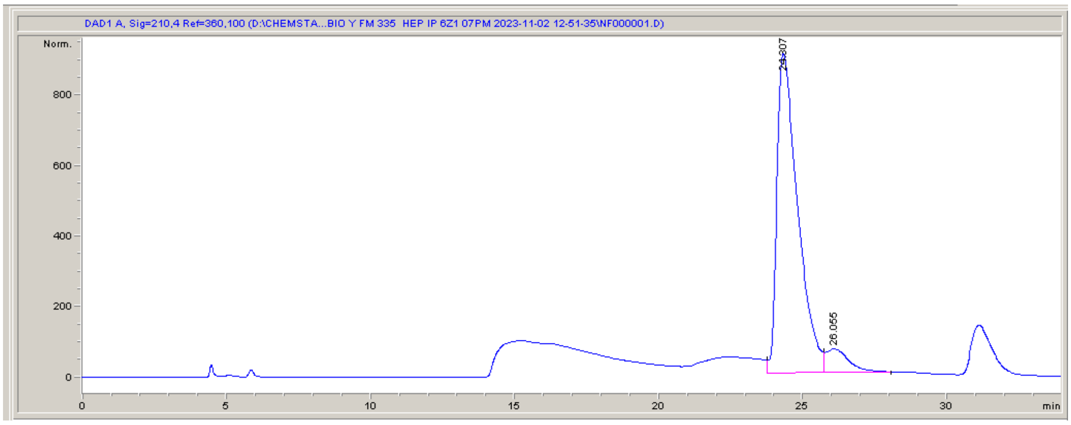

#	Time	Area	Height	Width	Area%	Symmetry
1	24.307	45757.5	907.5	0.7342	92.281	0.41
2	26.055	3827.3	67.9	0.7936	7.719	0.445

5 X-ray Structures

5.1 X-ray Structure of 2d

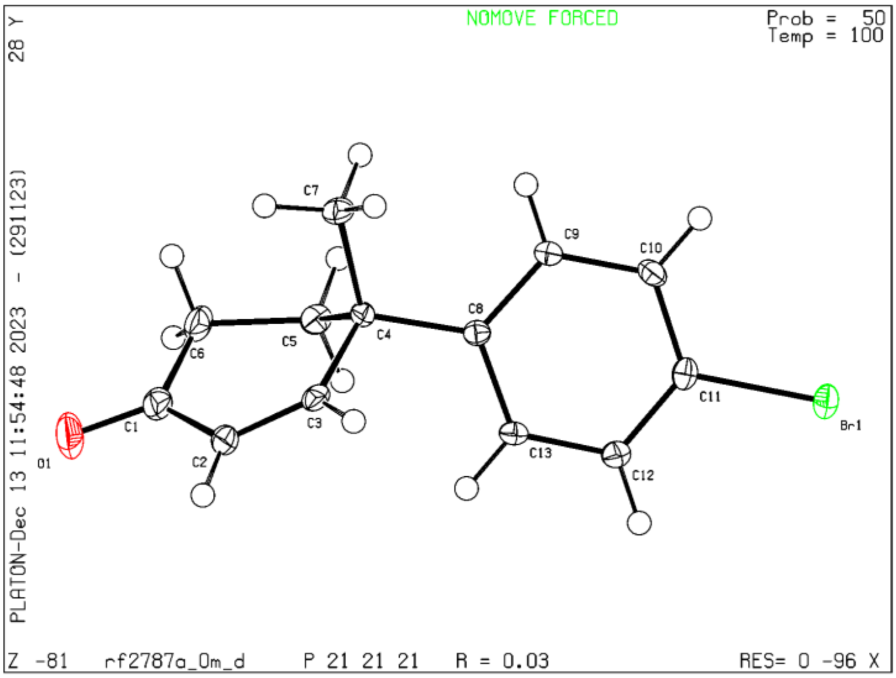

Table S8. Crystal data and structure refinement for 2d.

Identification code	RF2787a_0m_d
Empirical formula	$C_{13}H_{13}BrO$
Formula weight	265.14
Temperature/K	99.96
Crystal system	orthorhombic
Space group	$P2_12_12_1$
a/Å	5.9931(4)
b/Å	7.2804(5)
c/Å	25.6891(18)
α/°	90
β/°	90
γ/°	90
Volume/Å^3	1120.87(13)
Z	4
ρ_{calc}g/cm^3	1.571
μ/mm^{-1}	3.636
F(000)	536
Crystal size/mm^3	0.29 × 0.16 × 0.04
Radiation	MoKα (λ = 0.71073)
Θ range for data collection/°	3.172 to 30.529
Index ranges	$-8 \leq h \leq 8, -10 \leq k \leq 10, -36 \leq l \leq 36$
Reflections collected	84464
Independent reflections	3445 [R_{int} = 0.0628, R_{sigma} = 0.0218]

Data/restraints/parameters	3445/0/137
Goodness-of-fit on F^2	1.127
Final R indexes [I>=2σ (I)]	R_1 = 0.0286, wR_2 = 0.0751
Final R indexes [all data]	R_1 = 0.0307, wR_2 = 0.0761
Largest diff. peak/hole / e $Å^{-3}$	0.78/-0.83
Flack parameter	0.004(4)
**CCDC**	**2324913**

5.2 X-ray Structure of 5

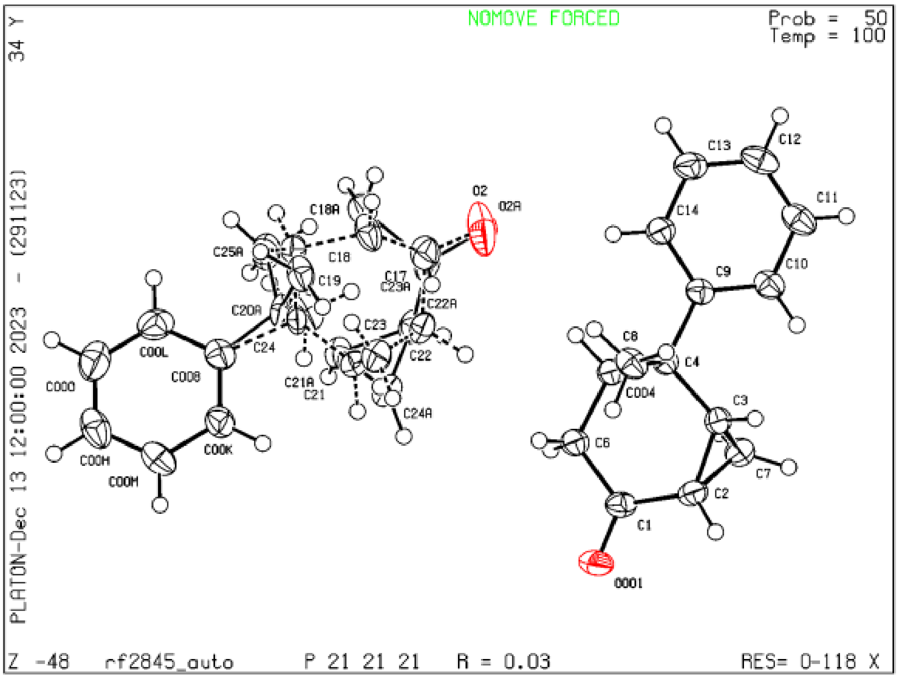

Table S9 Crystal data and structure refinement for 5.

Identification code	RF2845_auto
Empirical formula	$C_{14}H_{16}O$
Formula weight	200.27
Temperature/K	100
Crystal system	orthorhombic
Space group	$P2_12_12_1$
Flack Parameter	0.00(4)
a/Å	7.02879(4)
b/Å	11.82858(6)
c/Å	26.67886(14)
α/°	90
β/°	90
γ/°	90
Volume/Å^3	2218.10(2)
Z	8
ρ_{calc}g/cm^3	1.200
μ/mm^{-1}	0.567
F(000)	864
Crystal size/mm^3	0.20 × 0.08 × 0.05
Radiation	CuKα (λ = 1.54184)
Θ range for data collection/°	3.313 to 80.800

Index ranges	$-8 \le h \le 7$, $-15 \le k \le 15$, $-33 \le l \le 34$
Reflections collected	98661
Independent reflections	4842 [Rint = 0.0491, Rsigma = 0.0121]
Data/restraints/parameters	4842/0/356
Goodness-of-fit on F^2	1.047
Final R indexes [I>=2σ (I)]	$R_1 = 0.0326$, $wR_2 = 0.0886$
Largest diff. peak/hole/ e $Å^{-3}$	0.13/-0.16
**CCDC**	**2324914**

6 Determination of pre-steady-state kinetics: stopped flow measurements

The apparent kinetic parameters of the oxidative half-reaction were determined using a stopped-flow device (SF-61DX2, TgK Scientific, Bath, UK) under anoxic conditions (O_2 = 7–9 ppm) in a glove box (Belle Technology, Weymouth, UK) at 25 °C. To provide anoxic conditions, the reaction (50 mM NaPi, 150 mM NaCl, pH 7.5) and substrate buffer (50 mM NaPi,150 mM NaCl, 9% v/v DMSO, pH 7.5) were flushed and incubated with nitrogen in the glove box overnight. YqjM, pre-weighed NADH, and the substrate (dissolved in substrate buffer to a final concentration of 2.5 mM) were incubated in the glove box for at least 30 min or until the O_2 level reached < 9 ppm and diluted to the desired concentration directly in the glove box. A KinetaScanT diode array detector (MG-6560, Hi-Tech Scientific, Wiltshire, UK) was used to monitor spectral changes of the flavin cofactor at a wavelength of 450 nm, and collected data were analyzed using the Kinetic Studio software (version 4.01, TgK Scientific, Bath, UK). For the oxidative rates, 40 µM enzyme were pre-reduced using sub-stoichiometric amounts of NADH and shot against increasing substrate concentrations. The resulting increase in absorbance at 450 nm, which is due to the reoxidation of the reduced flavin, was used to determine the kinetic parameters. All measurements were performed in triplicates. The apparent rates of oxidation (k_{ox}) were visualized using the GraphPad Prism software (v8.4.3, GraphPad Software, San Diego, CA, USA).

The obtained raw data of the above described stopped flow measurements are disclosed in Table S10.

Table S10. Oxidative rates (OR) of YqjM using different concentrations of **1a** and **2a**, respectively. Measurements were performed in triplicates. SD= standard deviation.

C [µM]	OR-**1a** [µmol/s]			**SD** [µmol/s]	OR-**2a** [µmol/s]			**SD** [µmol/s]
120	0.02982	0.03061	0.02805	0.001311	n.a	n.a	n.a	n.a
200	0.05578	0.05891	0.05736	0.001565	n.a	n.a	n.a	n.a
280	0.08157	0.08258	0.08115	0.000735	n.a	n.a	n.a	n.a
360	0.11009	0.11363	0.11141	0.001789	n.a	n.a	n.a	n.a
500	0.15631	0.16204	0.16391	0.00396	n.a	n.a	n.a	n.a
800	0.2853	0.28599	0.29876	0.00758	0.0024	0.00246	0.00222	0.000125
1000	n.a	n.a	n.a	n.a	0.003	0.00298	0.00304	3.06E-05
1250	0.44978	0.44831	0.45155	0.001622	0.00356	0.00377	0.00358	0.000116

In Figure S5 a representative dataset for the detrmination of the reported oxidative rates is depicted.

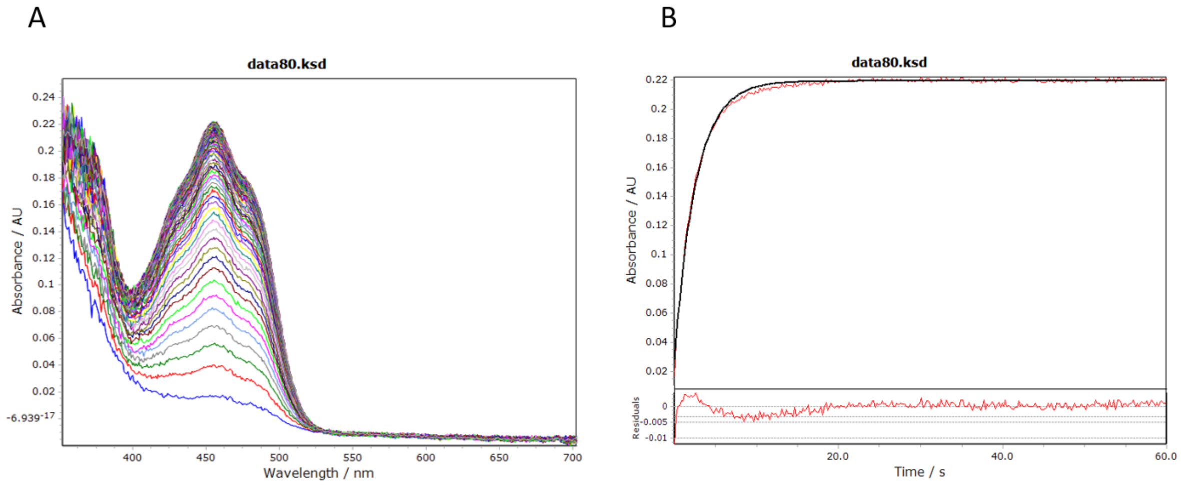

Figure S5. Representative dataset of YqjM oxidation using 1250 µM of **1a**. A) shows the spectral changes of reduced FMN until full oxidation level. B) shows the respective fit of the data shown in A.

7 Theoretical studies

7.1 Protein structure preparation and docking

The crystal structure of YqjM (PDB ID: 1Z44)[12] was downloaded, and all the ligands except the flavin cofactor were deleted. In the crystal, YqjM is a homodimer, where residue R336 of chain B interacts with the active site of chain A and vice versa. Hence, both chains were considered in this study. Further, the structure was prepared using the protein preparation wizard[13] in the Maestro program. Specifically, the two active site histidines (His-164 and His-167) were modelled as neutral and singly protonated at the ε- and δ-nitrogen atoms, respectively.

Comparing our substrate to a typical OYE substrate like 2-cyclohexen-1-one, it is essential that the carbonyl group of our substrate forms hydrogen bonds with the active site histidines. To explore this binding orientation, we have performed molecular docking of **1a** inside the active site of YqjM, revealing two promising poses where the carbonyl group of **1a** formed hydrogen bonds with the active site histidines and had similar binding energies. Docking was performed using Glide software.[14] The centre of the receptor grid was placed on the centre of mass (isoalloxazine) of the flavin cofactor in chain A, followed by the extra precision Glide docking protocol.[15]

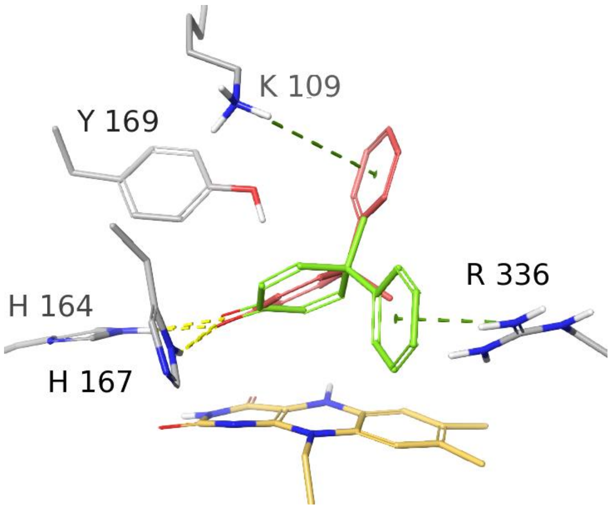

Figure S6. Structural superposition 129 of the two docking poses of **1a** inside the active site of YqjM.

The two docking poses of the **1a** are shown in red and green sticks. Flavin and the side chains of active site residues are shown in yellow and grey sticks, respectively. The yellow dotted line represents the hydrogen bond whereas the green dotted line depicts the cation-pi interaction.

7.2 Binding Pose Metadynamics (BPMD)

Binding pose metadynamics (BPMD) was utilized to discern the most stable binding pose among the two promising docking poses described above. The BPMD protocol has been developed to assess the stability of ligands in a binding pocket.[16,17] It is a variant of metadynamics simulations, where the root-mean-square deviation (RMSD) of bound ligand heavy atoms measures its stability in the binding pocket. A loosely bound ligand will result in a higher RMSD compared to tightly bound ligands occupying the same binding site. The chosen pose, exhibiting the lowest root mean square deviation (RMSD) from its initial position, was subjected to a 500 ns molecular dynamics (MD) simulation.

7.3 MD Simulations

The chosen stable pose then undergoes an energy minimization step only for the hydrogen atoms, followed by solvation with a buffer of at least 10 Å surrounding the protein and neutralization by adding a respective number of ions.

For equilibration, we run 50 ps NVT at 10 K and 50 ps NVT at 300 K, followed by 100ps NPT at 300 K with restraining solute-heavy atoms. Finally, a short 10 ps NVT without any restraints at 300 K. The equilibrated system was then simulated for 500 ns at 300 K. The program Desmond[18] was used with the OPLS4[19] all-atom force-field. A time step of 1 fs was used throughout the simulations, along with the Nose–Hoover[20] thermostat and Martyna–Tobias–Klein[21] barostat, with relaxation times of 1.0 and 2.0 ps, respectively. The particle mesh Ewald[22] method was used to treat long-range interactions, and a nonbonded cutoff of 9.0 Å was used for short-range interactions. Throughout the simulations, we have applied harmonic restraints to keep the substrate's carbonyl group within the hydrogen bonding distance from the two active site histidines.

7.4 Computation of Near Attack Conformations (NACs)

Near attack conformations (NACs) represent subpopulations of substrate that approach the configuration of the relevant transition state, often defined in terms of distances and angles.[23] Figure S6 shows the characterization of NACs for our productive binding mode based on the typical OYE catalytic mechanism.[24,25] Basically, we have considered three parameters for computing NACs for each of the double bonds. First, the distance between the hydride receiving

C-β atom and the hydride donating N5 atom of the flavin should be less than 3.6 Å. Second, the distance between the proton acceptor C-α atom and the proton donor oxygen atom of Y169 should be less than 3.6 Å, and the last one is the angle between the N1 of flavin, N5 of flavin and then hydride accepting C-β should be within 90-110 degrees. The described geometrical parameters for NACs have been averaged over production runs of 500 ns (in total 50K frames).

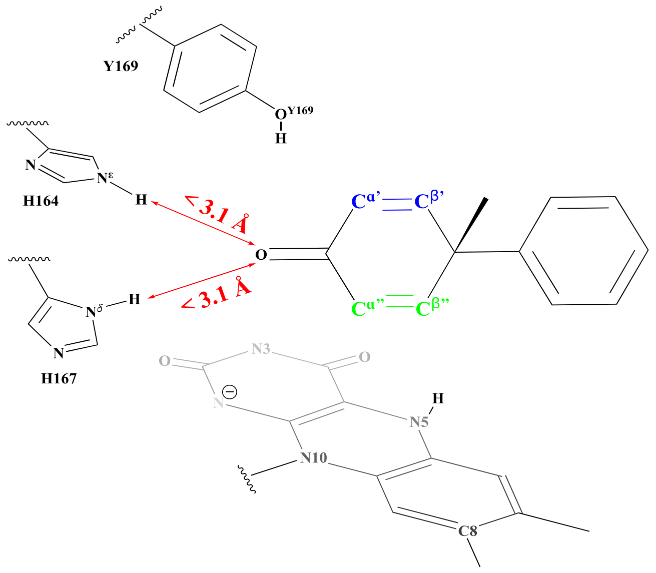

Near Attack Conformations (NACs) for "pro-S" binding pose	Near Attack Conformations (NACs) for "pro-R" binding pose
distance ($C^{\beta'}$-N5) < 3.6 Å<br>distance ($C^{\alpha'}$-O^{Y169}) < 3.6 Å<br>angle (N10-N5-$C^{\beta'}$) > 89 and < 111 degrees	distance ($C^{\beta''}$-N5) < 3.6 Å<br>distance ($C^{\alpha''}$-O^{Y169}) < 3.6 Å<br>angle (N10-N5-$C^{\beta''}$) > 89 and < 111 degrees

Figure S7. Near Attack Conformations (NACs) parameters for pro-*S* and pro-*R* binding poses.

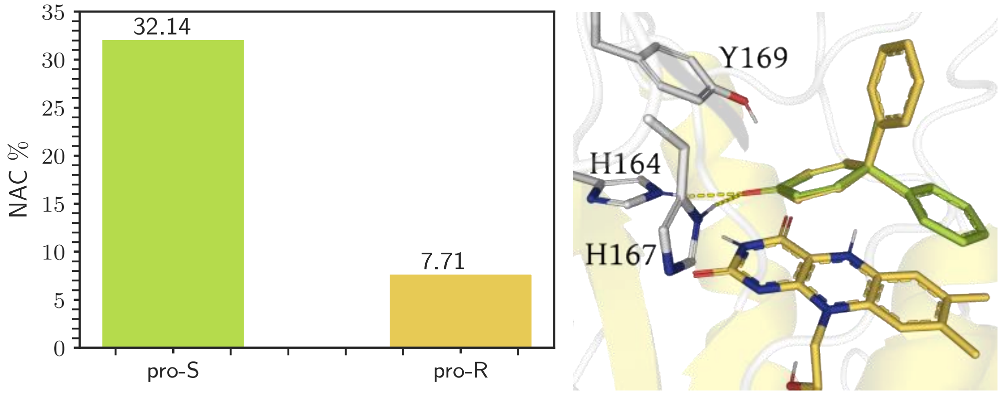

(a) (b)

Figure S8. NACs analysis of 500ns MD simulation

a) Percentage of NACs for pro-S and pro-R binding poses of the substrate. b) Snapshot representing the pro-S (in green sticks) and pro-R (in yellow sticks) binding poses of the substrate within the active site of YqjM obtained from the respective NACs subpopulation corresponding to each pose. Enzyme residues and the flavin are also shown as stick representations; the carbon atoms of the former are coloured grey, whereas the latter has its carbon atoms coloured yellow. Non-polar hydrogens and the main chain atoms are not shown for clarity.

7.5 QM/MM Computations

A random snapshot has been obtained from the *pro-S* NACs subpopulation. Solvent molecules not within 5 Å of any protein atom were removed, and the remaining atomic coordinates were used as input for Q-Site[26] (the QM/MM module of the Schrödinger package), where the QM region consists of side-chains of H164, H167 and Y169, substrate and the lumiflavin (LuF), bearing a net charge of -1. QM computation has been performed using the (UB3LYP-D3[27,28]/6-31G(d)[29]) QM method, basis set, and MM potential (OPLS-2005[30]). Dispersion corrections were added using D3 formalism.[31] MM atoms more than 5 Å away from the QM region were also fixed. Hydrogen atoms were used to cap covalent bonds partitioned at the QM/MM boundaries. From there, transition states were modelled using the quadratic synchronous transit (QST) method and further confirmed by the presence of a negative frequency corresponding to the selected reaction coordinates. All the structural figures have been prepared and rendered in Pymol (PyMOL Molecular Graphics System, Version 2.0 Schrödinger, LLC). The calculated energy values include a zero-point energy correction, enthalpy, and entropy contribution.

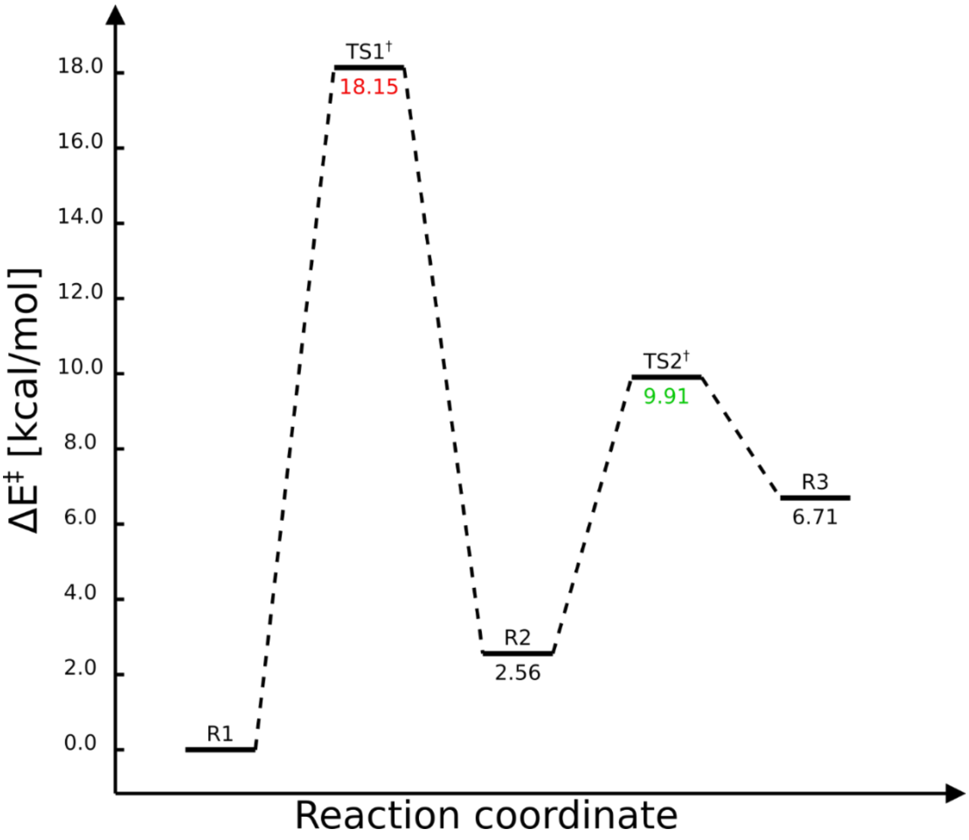

Figure S9. Overall reaction energies for the reduction of pro-*S* double bond of **1a** by YqjM.

TS1 depicts the transition state for the hydride transfer step, and TS2 displays the transition state for the proton transfer step. Energies are calculated relative to the reactant complex at the UB3LYP-D3/6-31G(d)/OPLS2005 level.

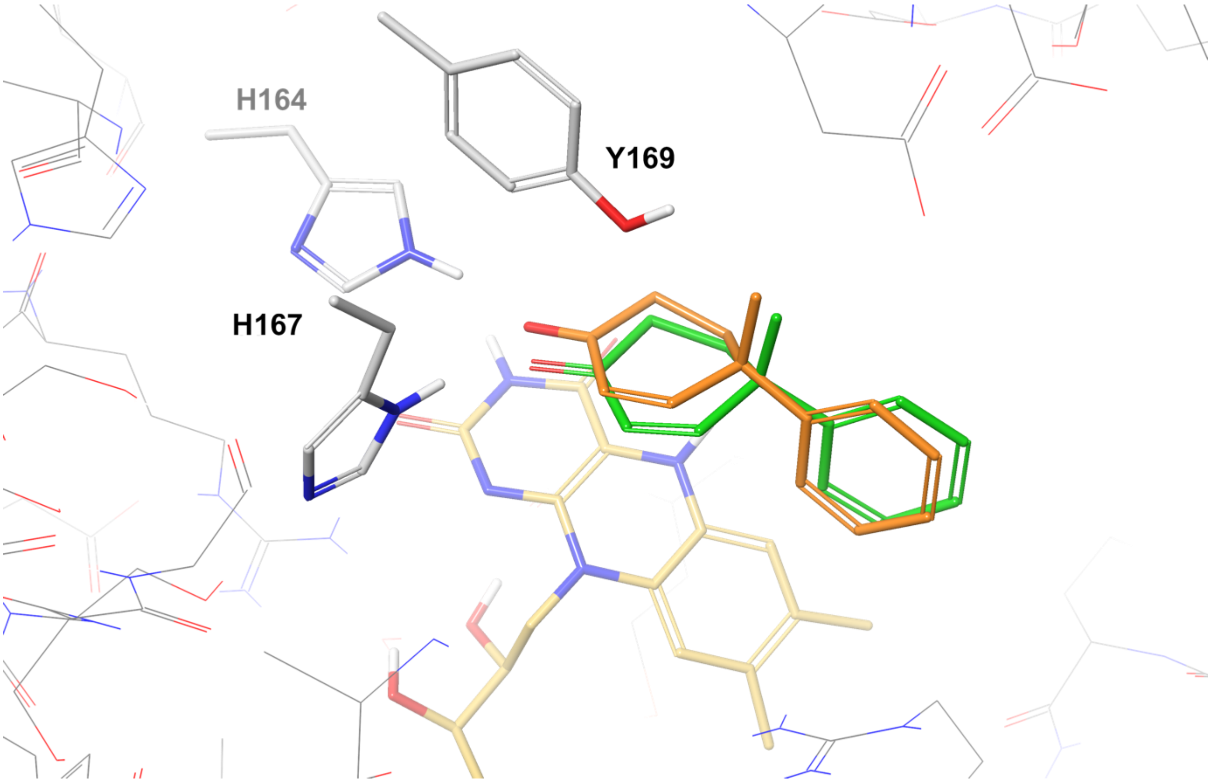

Figure S10. Structural superimposition of the docked pose of **2a** (green) and the product of the reduction of **1a** obtained from QM/MM computation (orange) within the active site of YqjM.

Important active site residues and the flavin cofactor are also shown as stick representations with grey and yellow carbon atoms, respectively. More distant amino acids are shown as lines. Non-polar hydrogens and the main chain atoms are omitted for clarity.

8 References

(1) Gottlieb, H. E.; Kotlyar, V.; Nudelman, A. NMR Chemical Shifts of Common Laboratory Solvents as Trace Impurities. *J. Org. Chem.* **1997**, *62* (21), 7512–7515.

(2) SADABS Version 2.0 Siemens Area Detector Correction, Universität Göttingen, Göttingen **2003**; Sheldrick.

(3) G.M. Sheldrick, GM SHELXS97 and SHELXL97, Universität Göttingen, Göttingen **2002.**

(4) Dolomanov, O. V.; Bourhis, L. J.; Gildea, R. J.; Howard, J. A. K.; Puschmann, H. OLEX2 : a complete structure solution, refinement and analysis program. *J Appl. Cryst.* **2009**, *42* (2), 339–341.

(5) Spek, A. L. Structure validation in chemical crystallography. *Acta crystallogr.* **2009**, *65* (Pt 2), 148–155.

(6) Sheldrick, G. M. SHELXT – Integrated space-group and crystal-structure determination. *Acta. Crystallogr.* **2015**, *71*, 3–8.

(7) Sheldrick, G. M. A short history of SHELX. *Acta. Crystallogr.* **2008**, *64* (1), 112-122.

(8) You, C.; Li, X.; Gong, Q.; Wen, J.; Zhang, X. Nickel-Catalyzed Desymmetric Hydrogenation of Cyclohexadienones: An Efficient Approach to All-Carbon Quaternary Stereocenters. *J. Am. Chem. Soc.* **2019**, *141* (37), 14560–14564.

(9) Naganawa, Y.; Kawagishi, M.; Ito, J.-I.; Nishiyama, H. Asymmetric Induction at Remote Quaternary Centers of Cyclohexadienones by Rhodium-Catalyzed Conjugate Hydrosilylation. *Angew. Chem. Int. Ed.* **2016**, *55* (24), 6873–6876.

(10) Fitzpatrick, T. B.; Amrhein, N.; Macheroux, P. Characterization of YqjM, an Old Yellow Enzyme homolog from Bacillus subtilis involved in the oxidative stress response. *J. Biol. Chem.* **2003**, *278* (22), 19891–19897.

(11) Strassner, J.; Fürholz, A.; Macheroux, P.; Amrhein, N.; Schaller, A. A homolog of old yellow enzyme in tomato. Spectral properties and substrate specificity of the recombinant protein. *J. Biol. Chem.* **1999**, *274* (49), 35067–35073.

(12) Kitzing, K.; Fitzpatrick, T. B.; Wilken, C.; Sawa, J.; Bourenkov, G. P.; Macheroux, P.; Clausen, T. The 1.3 A crystal structure of the flavoprotein YqjM reveals a novel class of Old Yellow Enzymes. *J. Biol. Chem.* **2005**, *280* (30), 27904–27913.

(13) Sastry, G. M.; Adzhigirey, M.; Day, T.; Annabhimoju, R.; Sherman, W. Protein and ligand preparation: parameters, protocols, and influence on virtual screening enrichments. *J. Comput. aided Mol.* **2013**, *27* (3), 221–234.

(14) Halgren, T. A.; Murphy, R. B.; Friesner, R. A.; Beard, H. S.; Frye, L. L.; Pollard, W. T.; Banks, J. L. Glide: a new approach for rapid, accurate docking and scoring. 2. Enrichment factors in database screening. *J. Med. Chem.* **2004**, *47* (7), 1750–1759.

(15) Friesner, R. A.; Murphy, R. B.; Repasky, M. P.; Frye, L. L.; Greenwood, J. R.; Halgren, T. A.; Sanschagrin, P. C.; Mainz, D. T. Extra precision glide: docking and scoring incorporating a model of hydrophobic enclosure for protein-ligand complexes. *J. Med. Chem.* **2006**, *49* (21), 6177–6196.

(16) Clark, A. J.; Tiwary, P.; Borrelli, K.; Feng, S.; Miller, E. B.; Abel, R.; Friesner, R. A.; Berne, B. J. Prediction of Protein-Ligand Binding Poses via a Combination of Induced Fit Docking and Metadynamics Simulations. *J. Chem. Theory Comput.* **2016**, *12* (6), 2990–2998.

(17) Fusani, L.; Palmer, D. S.; Somers, D. O.; Wall, I. D. Exploring Ligand Stability in Protein Crystal Structures Using Binding Pose Metadynamics. *J. Chem. Inf. Model.* **2020**, *60* (3), 1528–1539.

(18) Sievers, F.; Wilm, A.; Dineen, D.; Gibson, T. J.; Karplus, K.; Li, W.; Lopez, R.; McWilliam, H.; Remmert, M.; Söding, J.; Thompson, J. D.; Higgins, D. G. Fast, scalable generation of high-quality protein multiple sequence alignments using Clustal Omega. *Mol. Sys. Biol.* **2011**, *7*, 539.

(19) Lu, C.; Wu, C.; Ghoreishi, D.; Chen, W.; Wang, L.; Damm, W.; Ross, G. A.; Dahlgren, M. K.; Russell, E.; Bargen, C. D. von; Abel, R.; Friesner, R. A.; Harder, E. D. OPLS4: Improving Force Field Accuracy on Challenging Regimes of Chemical Space. *J. Chem. Theory Comput.* **2021**, *17* (7), 4291–4300.

(20) Hoover, W. G. Canonical dynamics: Equilibrium phase-space distributions. *PRA* **1985**, *31* (3), 1695–1697.

(21) Martyna, G. J.; Tobias, D. J.; Klein, M. L. Constant pressure molecular dynamics algorithms. *J. Chem. Phys.* **1994**, *101* (5), 4177–4189.

(22) Darden, T.; York, D.; Pedersen, L. Particle mesh Ewald: An N ·log(N) method for Ewald sums in large systems. *J. Chem. Phys.* **1993**, *98* (12), 10089–10092.

(23) Sadiq, S. K.; Coveney, P. V. Computing the role of near attack conformations in an enzyme-catalyzed nucleophilic bimolecular reaction. *J. Chem. Theory Comput.* **2015**, *11* (1), 316–324.

(24) Breukelaar, W. B.; Polidori, N.; Singh, A.; Daniel, B.; Glueck, S. M.; Gruber, K.; Kroutil, W. Mechanistic Insights into the Ene-Reductase-Catalyzed Promiscuous Reduction of Oximes to Amines. *ACS Catal.* **2023**, *13* (4), 2610–2618.

(25) Fraaije, M. W.; Mattevi, A. Flavoenzymes: diverse catalysts with recurrent features. *TIBS* **2000**, *25* (3), 126–132.

(26) Murphy, R. B.; Philipp, D. M.; Friesner, R. A. A mixed quantum mechanics/molecular mechanics (QM/MM) method for large-scale modeling of chemistry in protein environments. *J. Comput. Chem.* **2000**, *21* (16), 1442–1457.

(27) Lee, C.; Yang, W.; Parr, R. G. Development of the Colle-Salvetti correlation-energy formula into a functional of the electron density. *Phys. Rev. B. Condens.* **1988**, *37* (2), 785–789.

(28) Becke, A. D. A new mixing of Hartree–Fock and local density-functional theories. *J. Chem. Phys.* **1993**, *98* (2), 1372–1377.

(29) Hariharan, P. C.; Pople, J. A. The influence of polarization functions on molecular orbital hydrogenation energies. *Theoret. Chim. Acta* **1973**, *28* (3), 213–222.

(30) Shivakumar, D.; Harder, E.; Damm, W.; Friesner, R. A.; Sherman, W. Improving the Prediction of Absolute Solvation Free Energies Using the Next Generation OPLS Force Field. *J. Chem. Theory Comput.* **2012**, *8* (8), 2553–2558.

(31) Grimme, S.; Antony, J.; Ehrlich, S.; Krieg, H. A consistent and accurate *ab initio* parametrization of density functional dispersion correction (DFT-D) for the 94 elements H-Pu. *J. Chem. Phys..* **2010**, *132*, 154104.

9 NMR-spectra

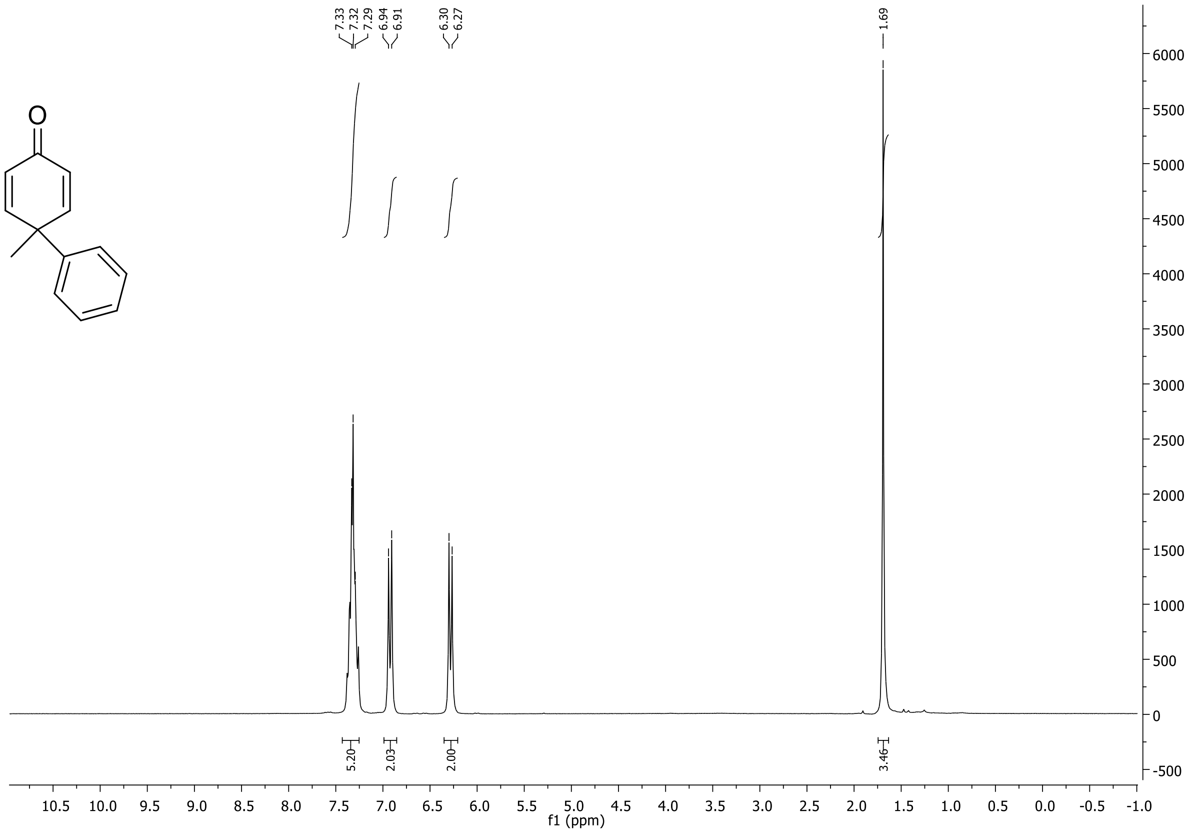

Figure S11. ^{1}H-NMR (300.36 MHz, $CDCl_3$) – **1-Methyl-[1,1'-biphenyl]-4(1*H*)-one** (**1a**).

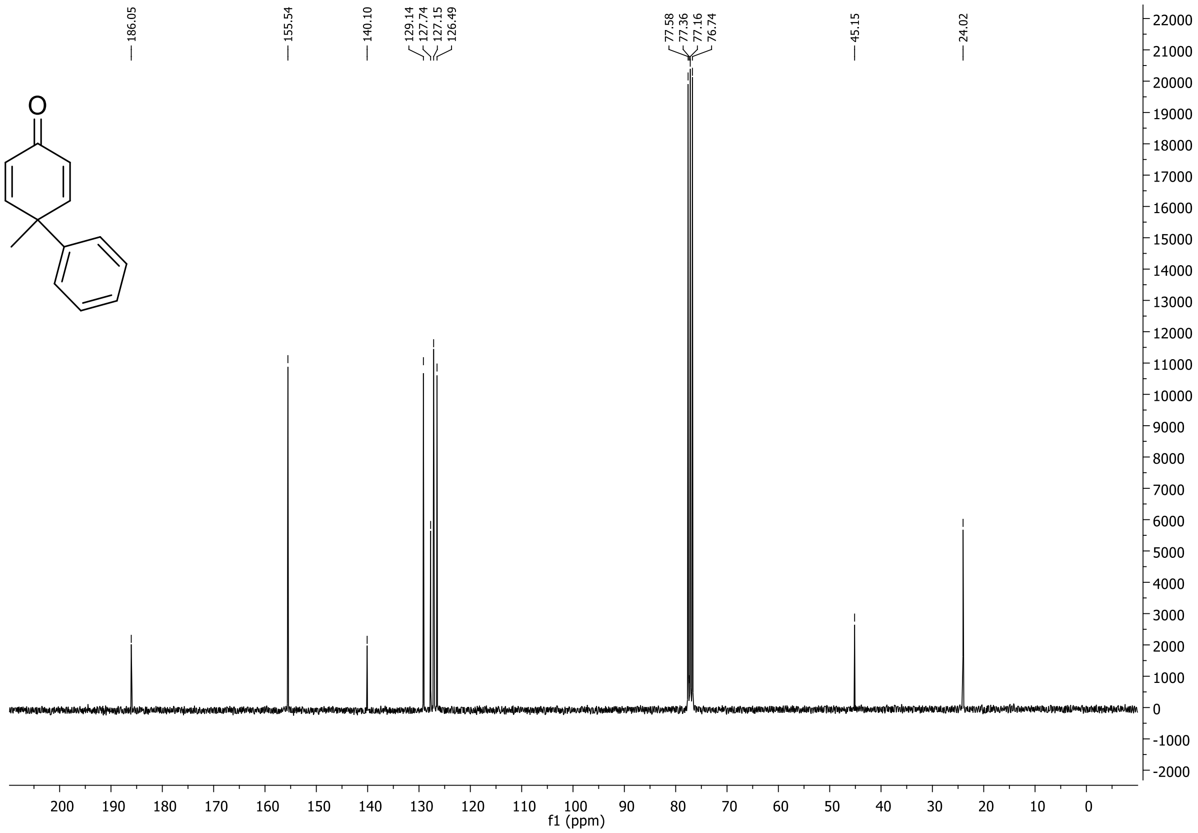

Figure S12. ^{13}C-NMR (75.53 MHz, $CDCl_3$) – **1-Methyl-[1,1'-biphenyl]-4(1*H*)-one** (**1a**).

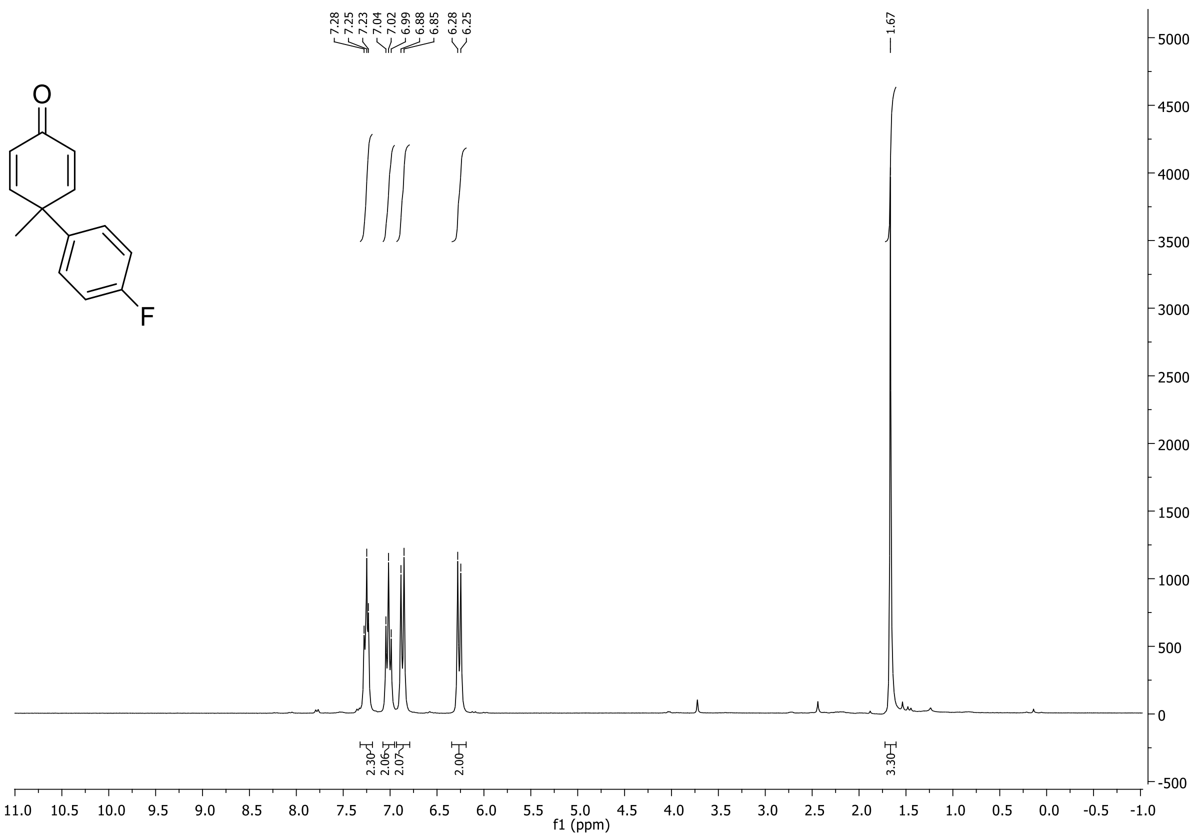

Figure S13. ^{1}H-NMR (300.36 MHz, $CDCl_3$) – **4'-Fluoro-1-methyl-[1,1'-biphenyl]-4(1*H*)-one (1b)**.

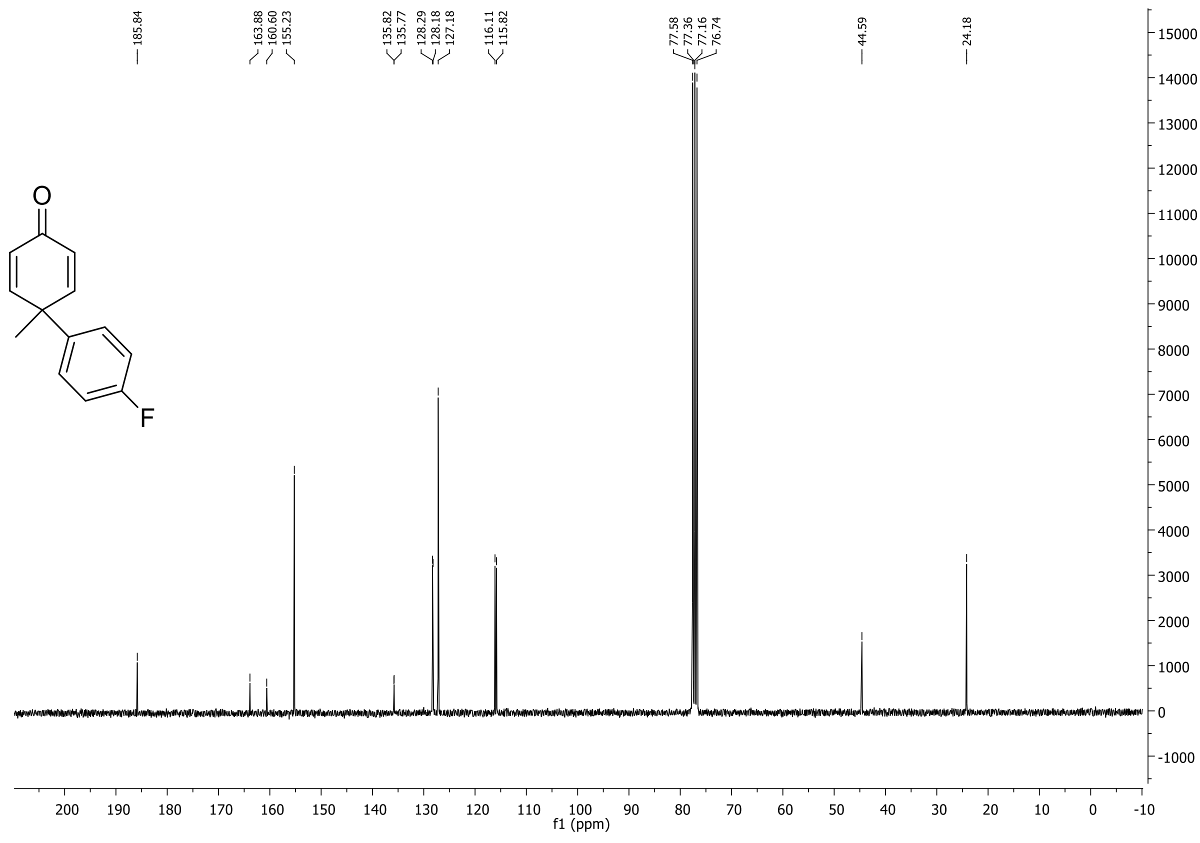

Figure S14. ^{13}C-NMR (75.53 MHz, $CDCl_3$) – **4'-Fluoro-1-methyl-[1,1'-biphenyl]-4(1*H*)-one (1b)**.

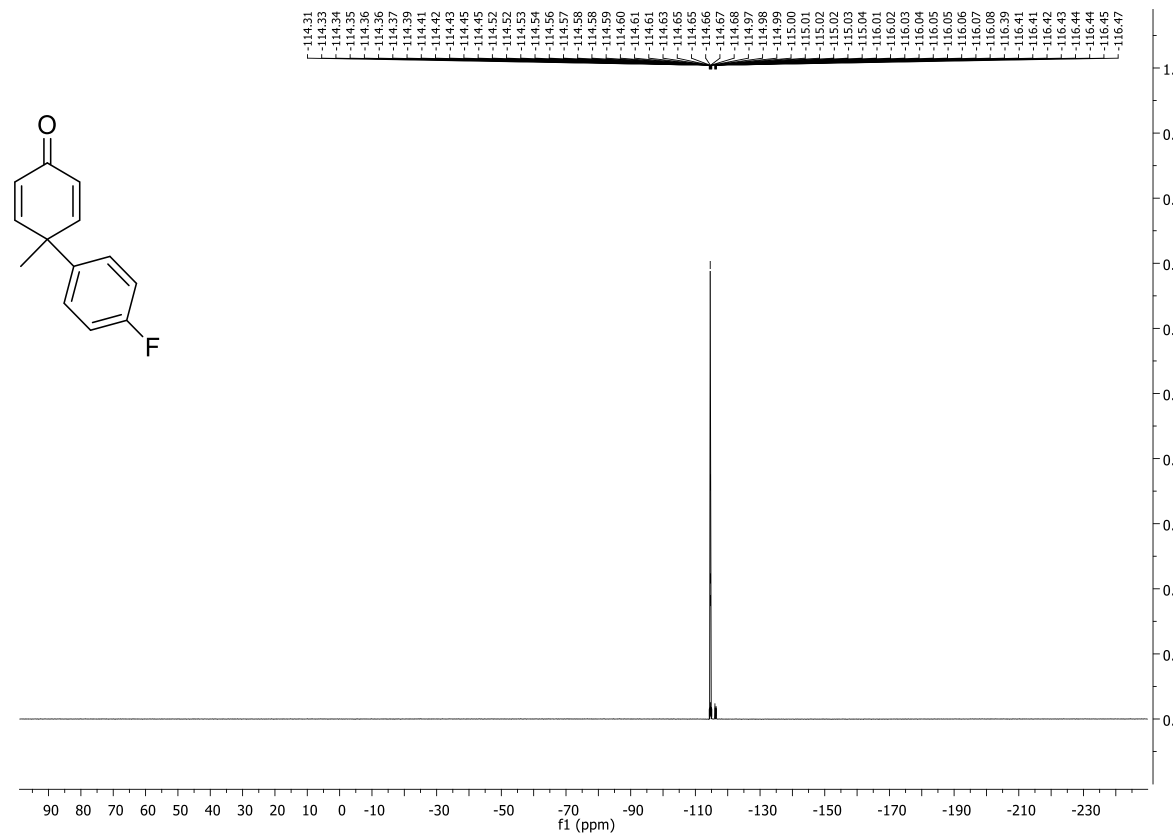

Figure S15. ^{19}F-NMR (376.17 MHz, $CDCl_3$) – **4’-Fluoro-1-methyl-[1,1’-biphenyl]-4(1*H*)-one (1b)**.

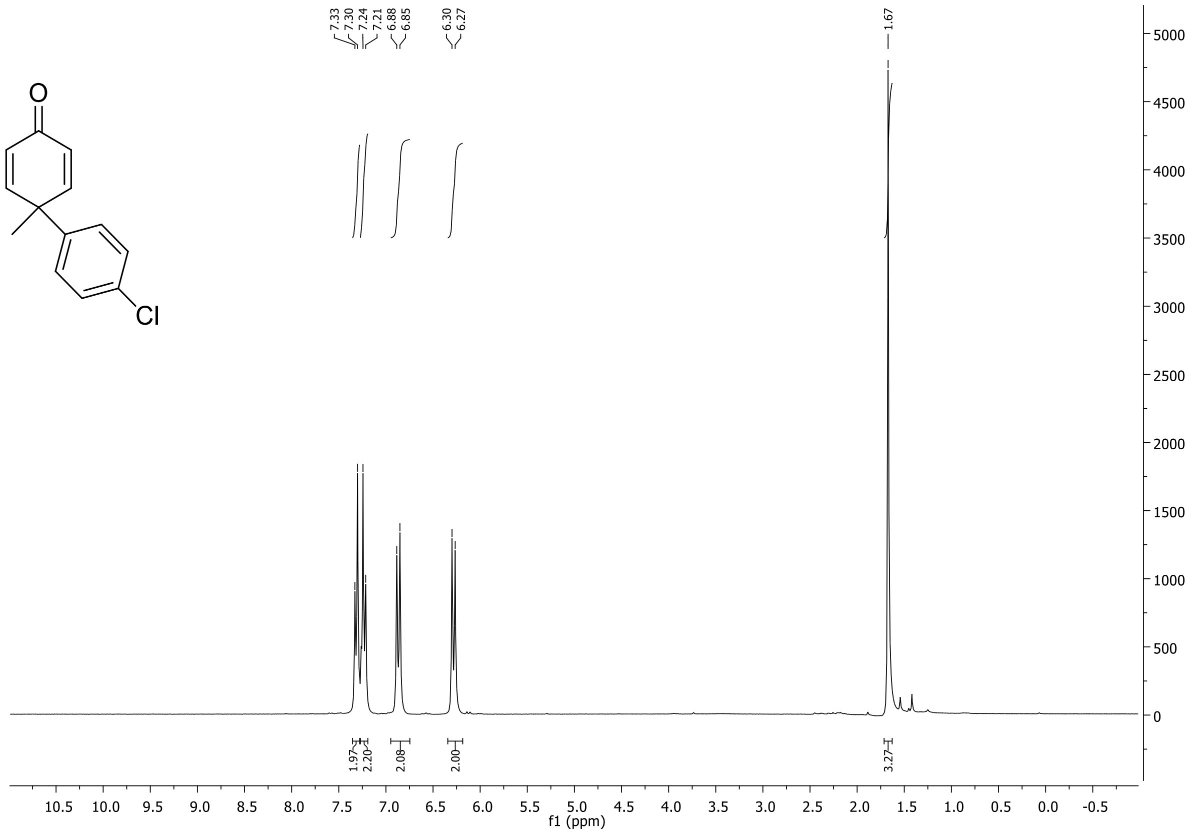

Figure S16. ^{1}H-NMR (300.36 MHz, $CDCl_3$) – **4’-Chloro-1-methyl-[1,1’-biphenyl]-4(1*H*)-one (1c)**.

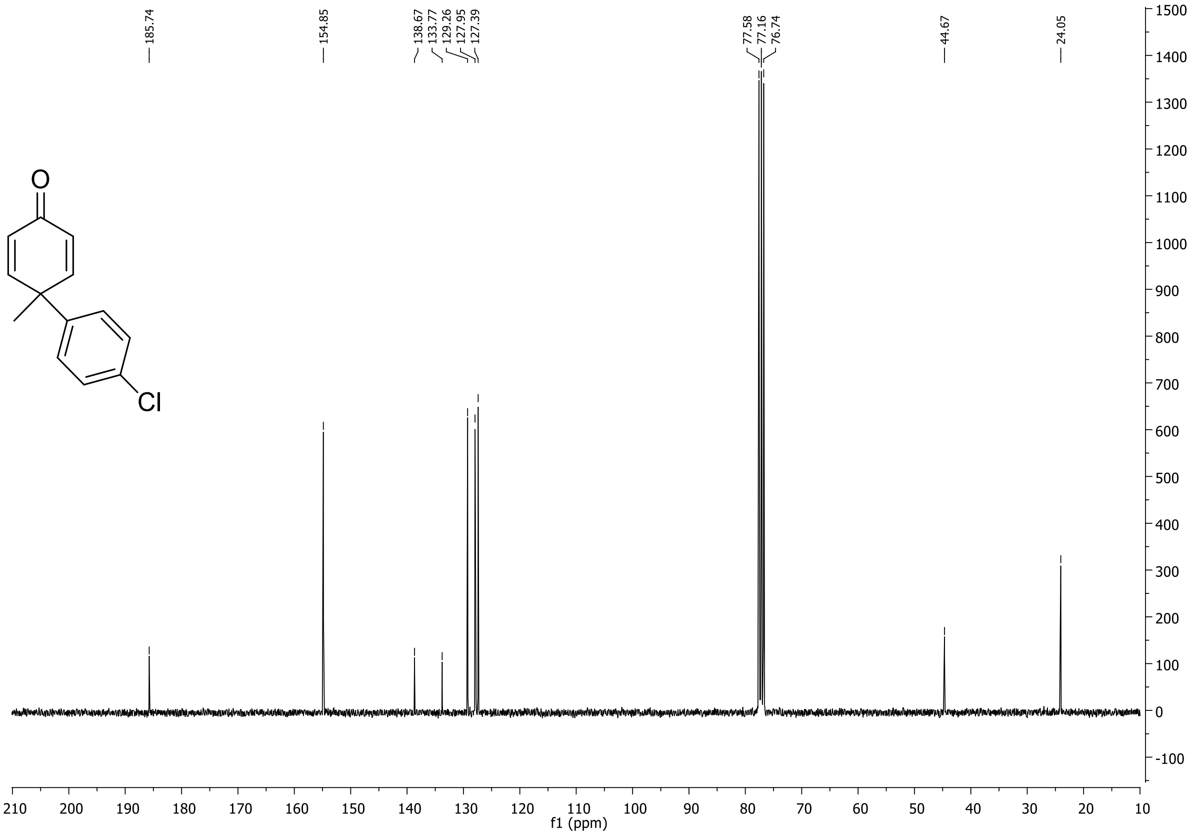

Figure S17. ^{13}C-NMR (75.53 MHz, $CDCl_3$) – **4’-Chloro-1-methyl-[1,1’-biphenyl]-4(1*H*)-one (1c)**.

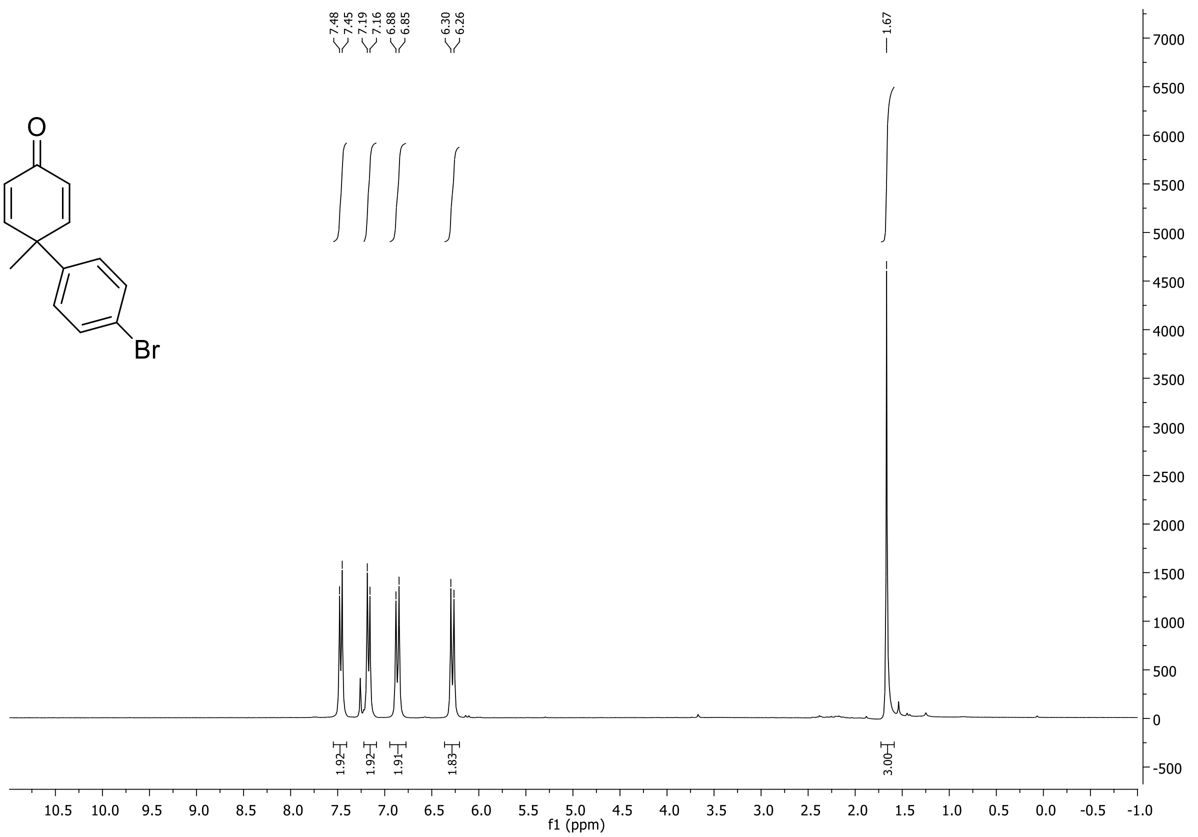

Figure S18. ^{1}H-NMR (300.36 MHz, $CDCl_3$) – **4'-Bromo-1-methyl-[1,1'-biphenyl]-4(1*H*)-one (1d)**.

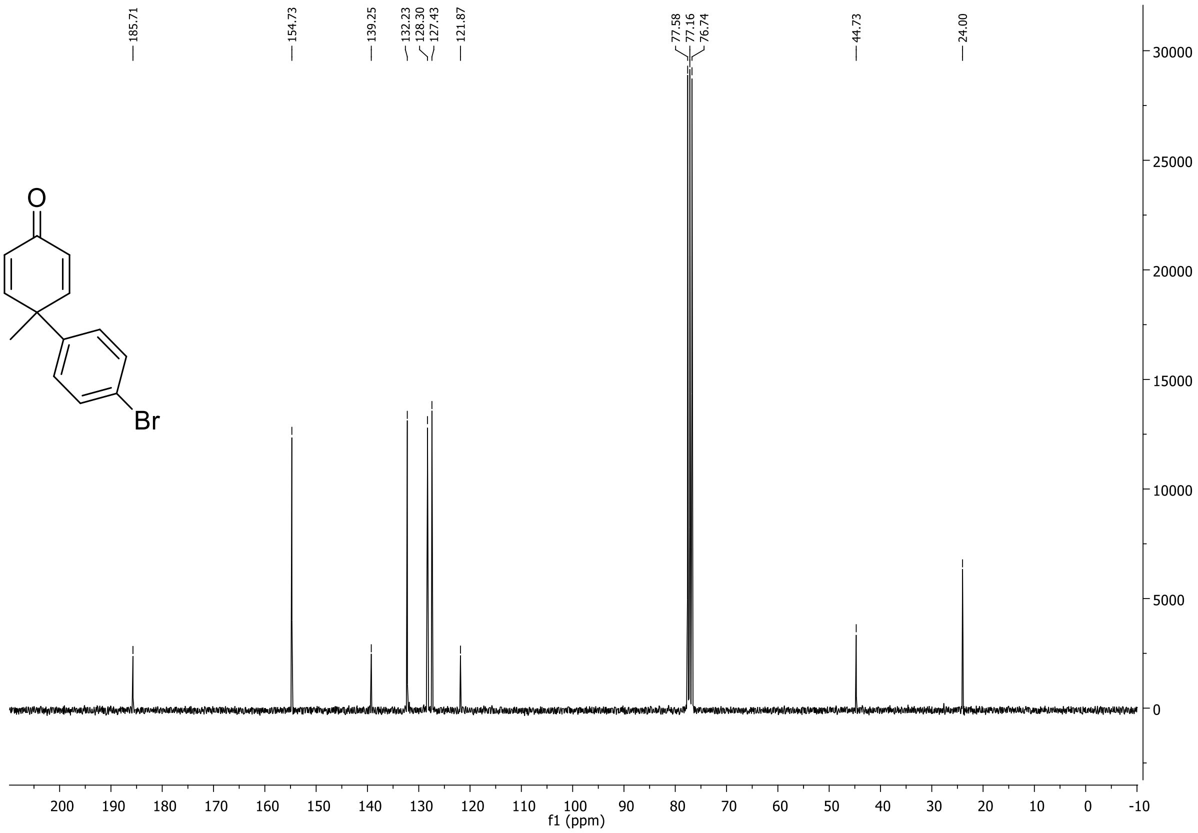

Figure S19. ^{13}C-NMR (75.53 MHz, $CDCl_3$) – **4'-Bromo-1-methyl-[1,1'-biphenyl]-4(1*H*)-one (1d)**.

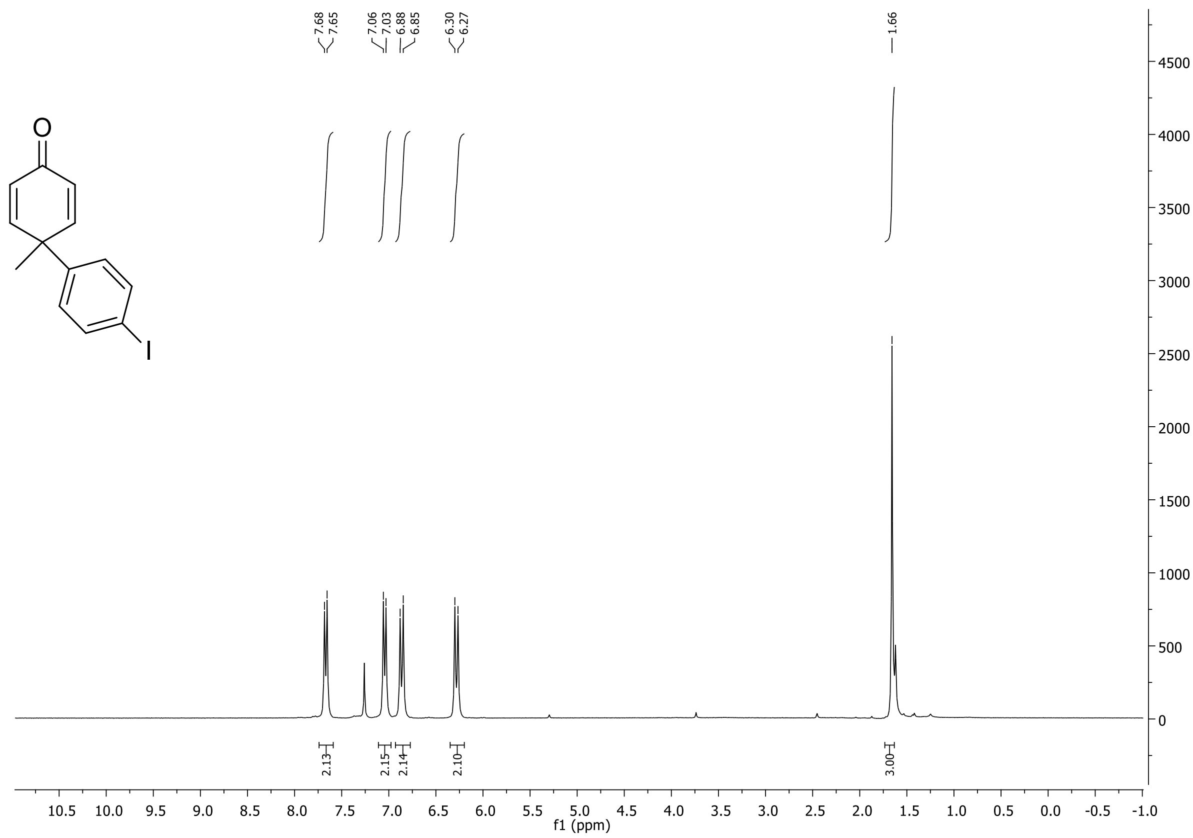

Figure S20. ^{1}H-NMR (300.36 MHz, $CDCl_3$) – **4’-Iodo-1-methyl-[1,1’-biphenyl]-4(1*H*)-one (1e)**.

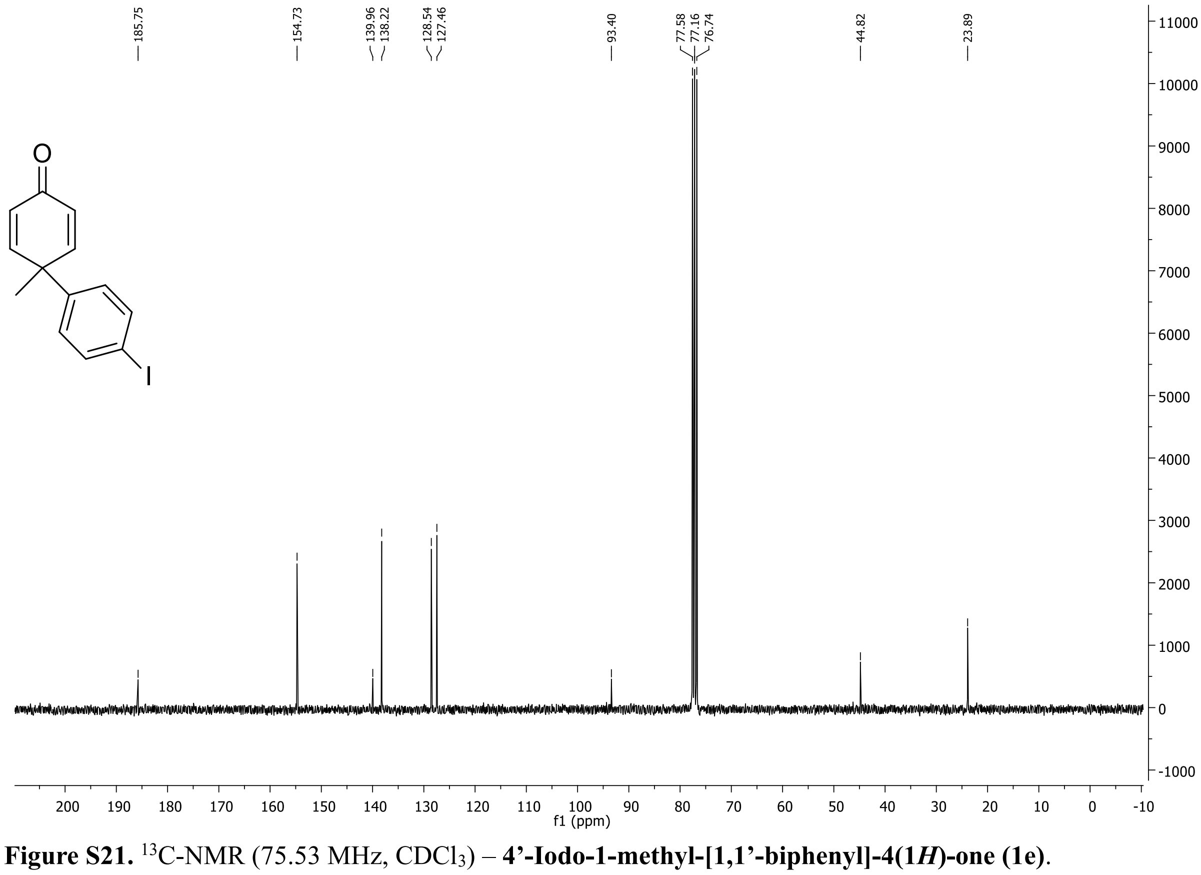

Figure S21. ^{13}C-NMR (75.53 MHz, $CDCl_3$) – **4’-Iodo-1-methyl-[1,1’-biphenyl]-4(1*H*)-one (1e)**.

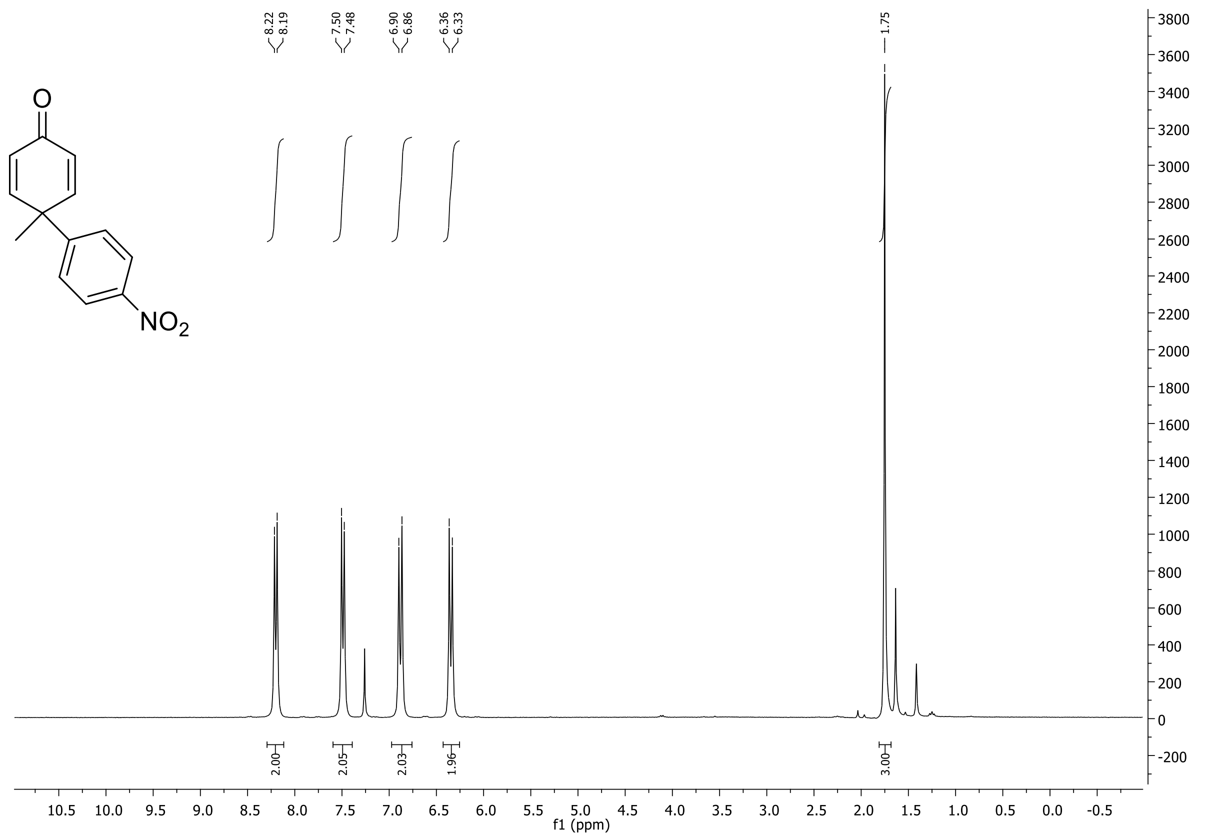

Figure S22. ^{1}H-NMR (300.36 MHz, $CDCl_3$) – **1-Methyl-4’-nitro-[1,1’-biphenyl]-4(1*H*)-one (1f)**.

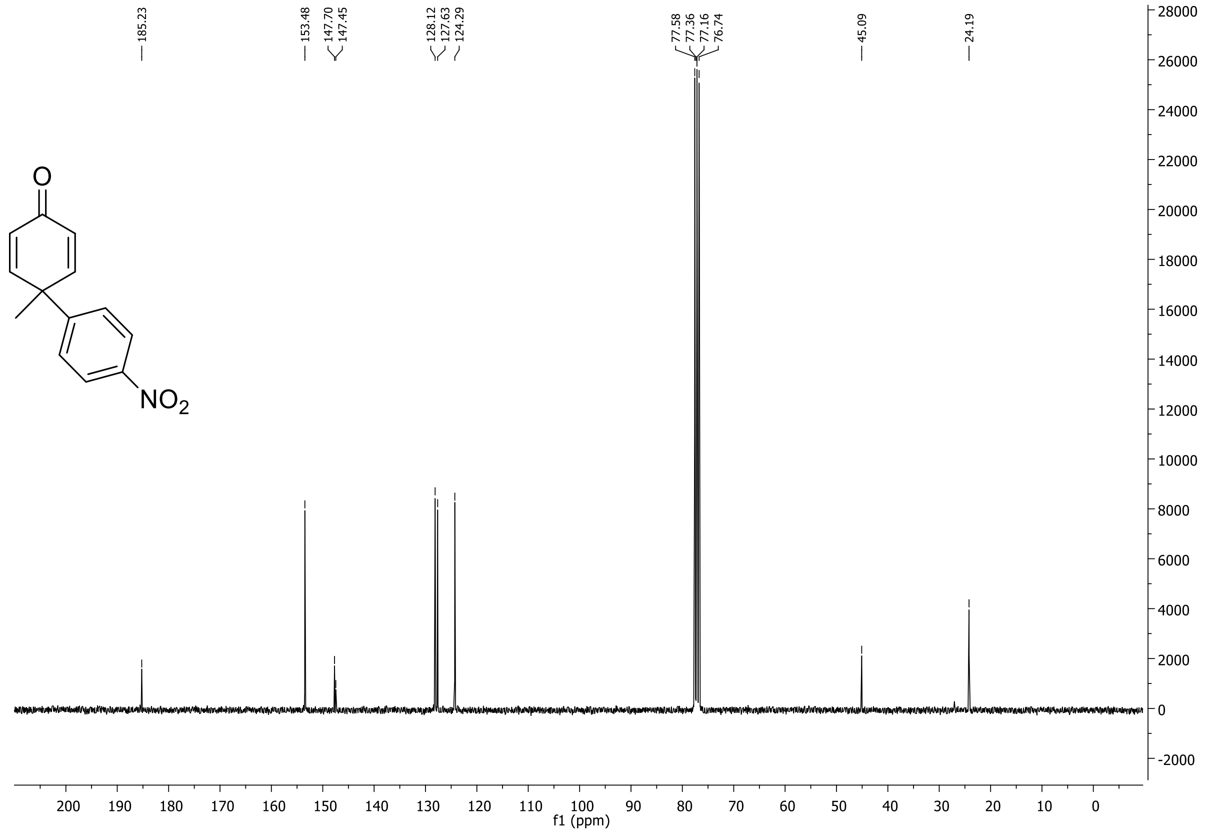

Figure S23. ^{13}C-NMR (75.53 MHz, $CDCl_3$) – **1-Methyl-4’-nitro-[1,1’-biphenyl]-4(1*H*)-one (1f)**.

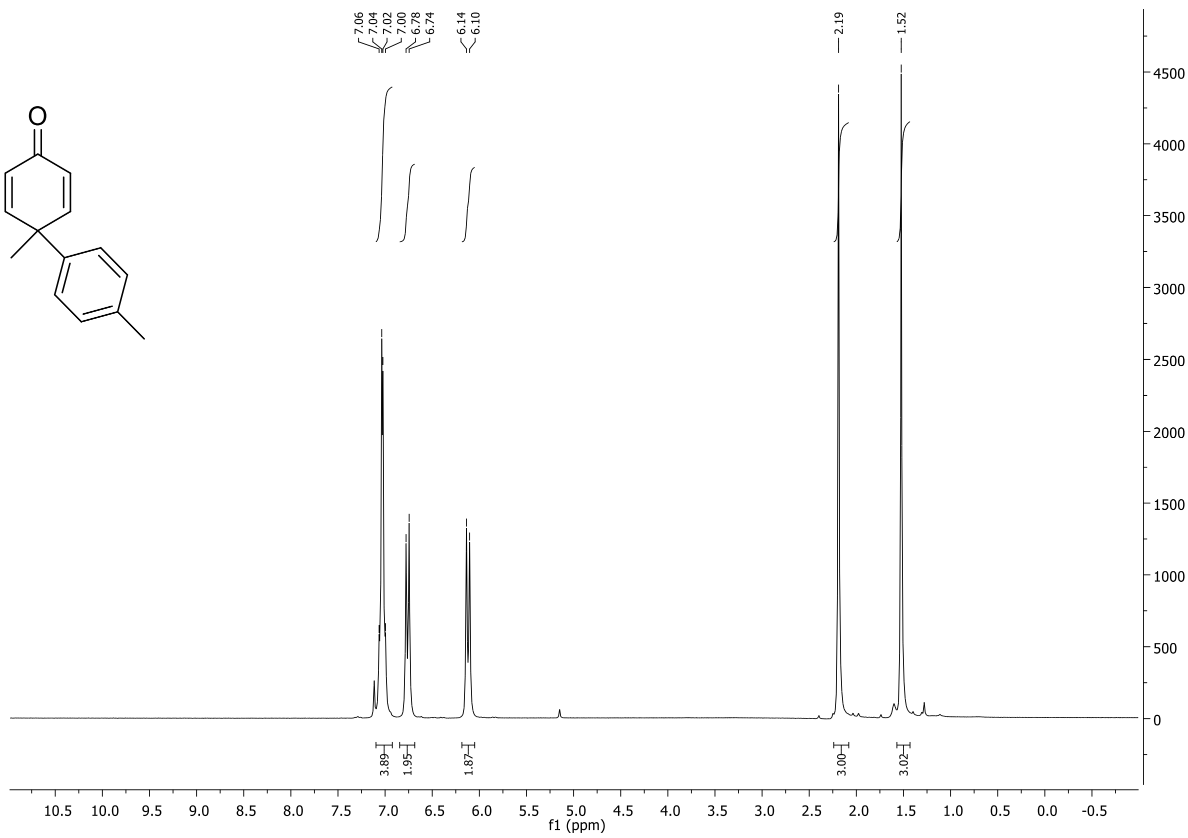

Figure S24. ^{1}H-NMR (300.36 MHz, $CDCl_3$) – **1,4'-Dimethyl-[1,1'-biphenyl]-4(1*H*)-one (1g)**.

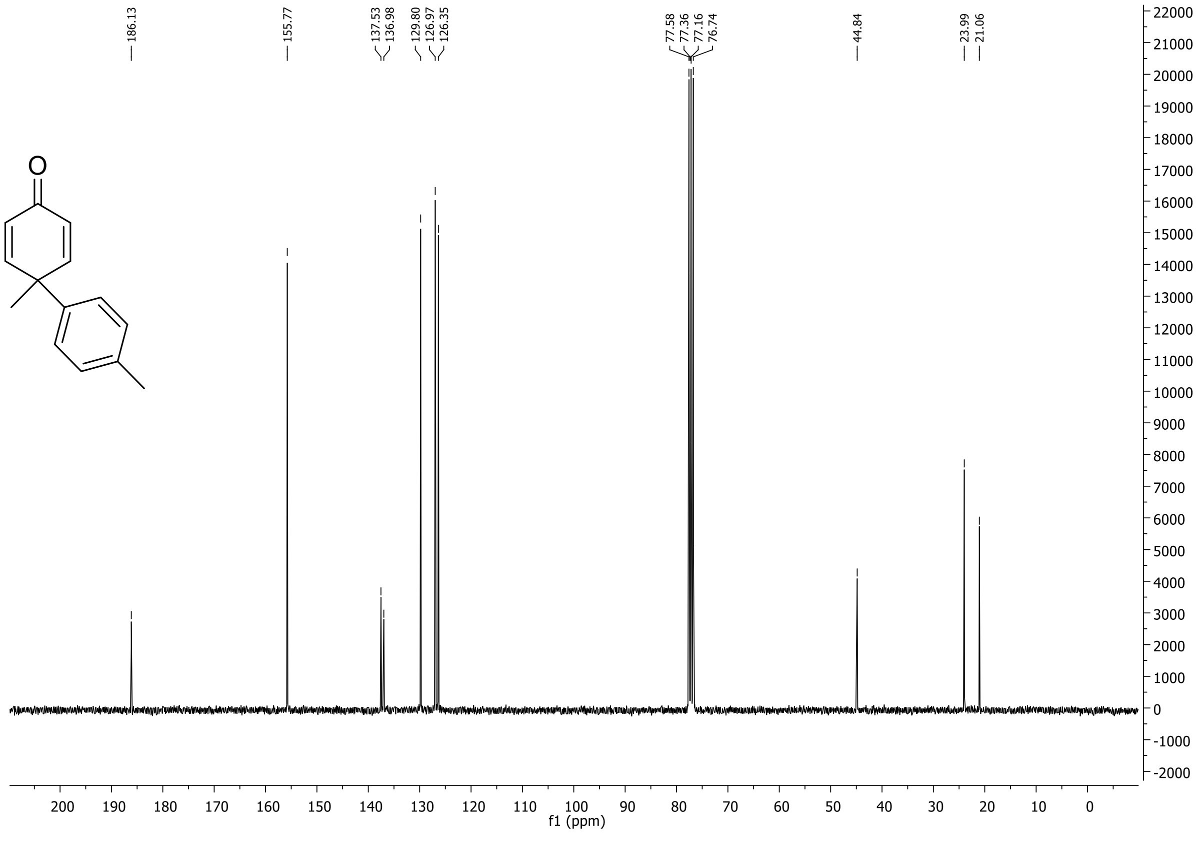

Figure S25. ^{13}C-NMR (75.53 MHz, $CDCl_3$) – **1,4'-Dimethyl-[1,1'-biphenyl]-4(1*H*)-one (1g)**.

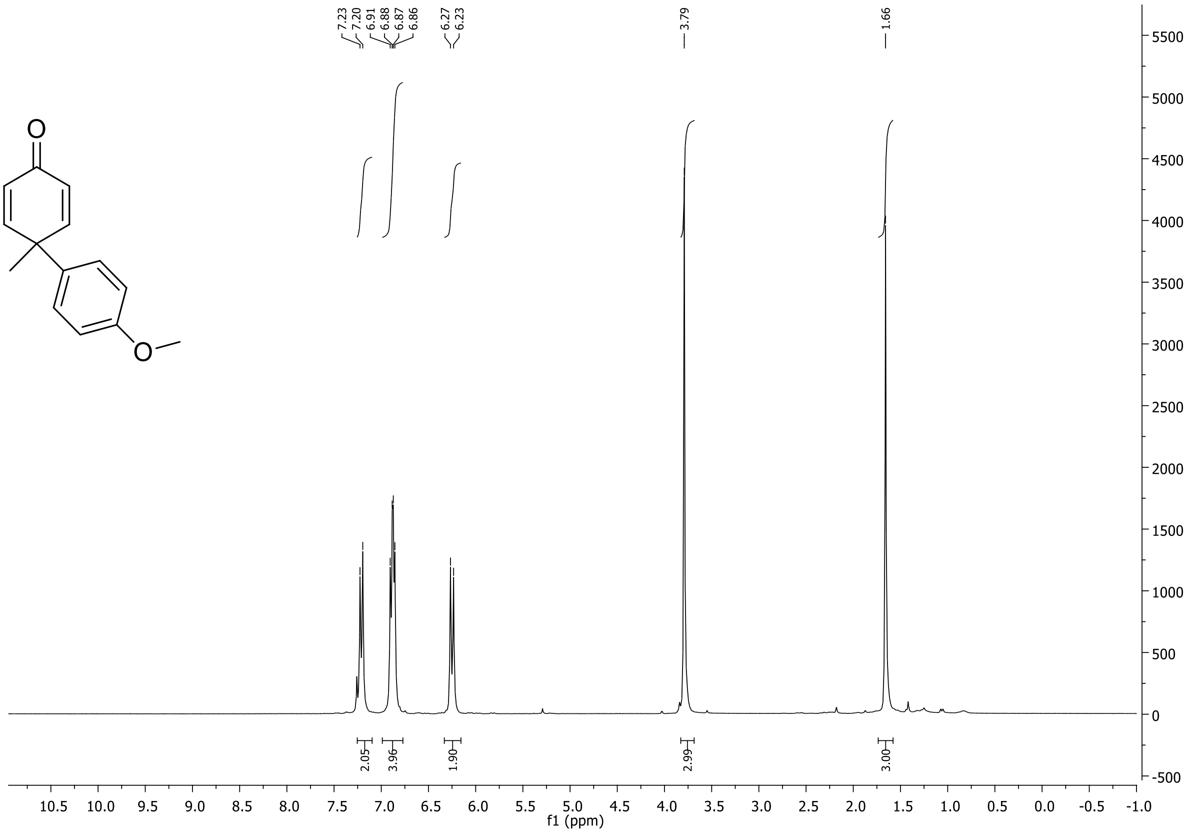

Figure S26. ^{1}H-NMR (300.36 MHz, $CDCl_3$) – **4'-Methoxy-1-methyl-[1,1'-biphenyl]-4(1*H*)-one (1h)**.

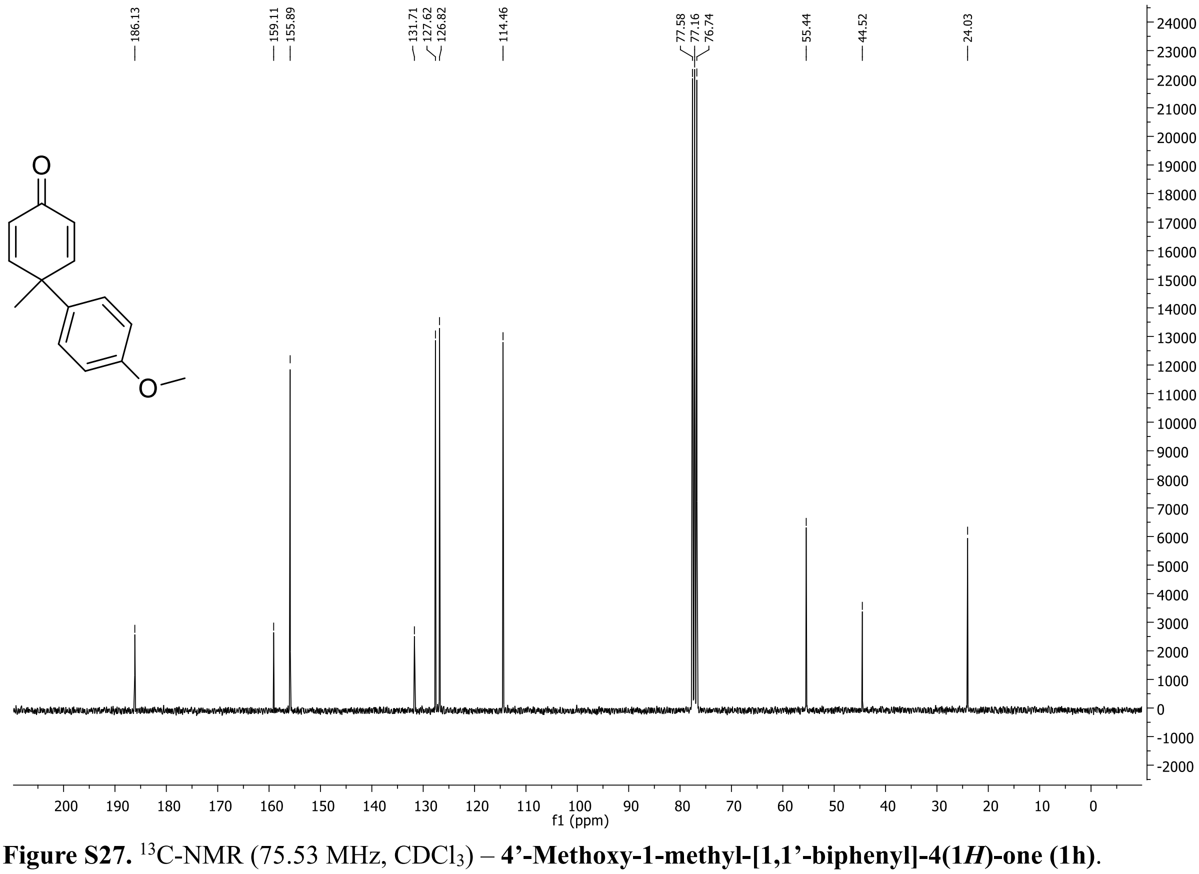

Figure S27. ^{13}C-NMR (75.53 MHz, $CDCl_3$) – **4'-Methoxy-1-methyl-[1,1'-biphenyl]-4(1*H*)-one (1h)**.

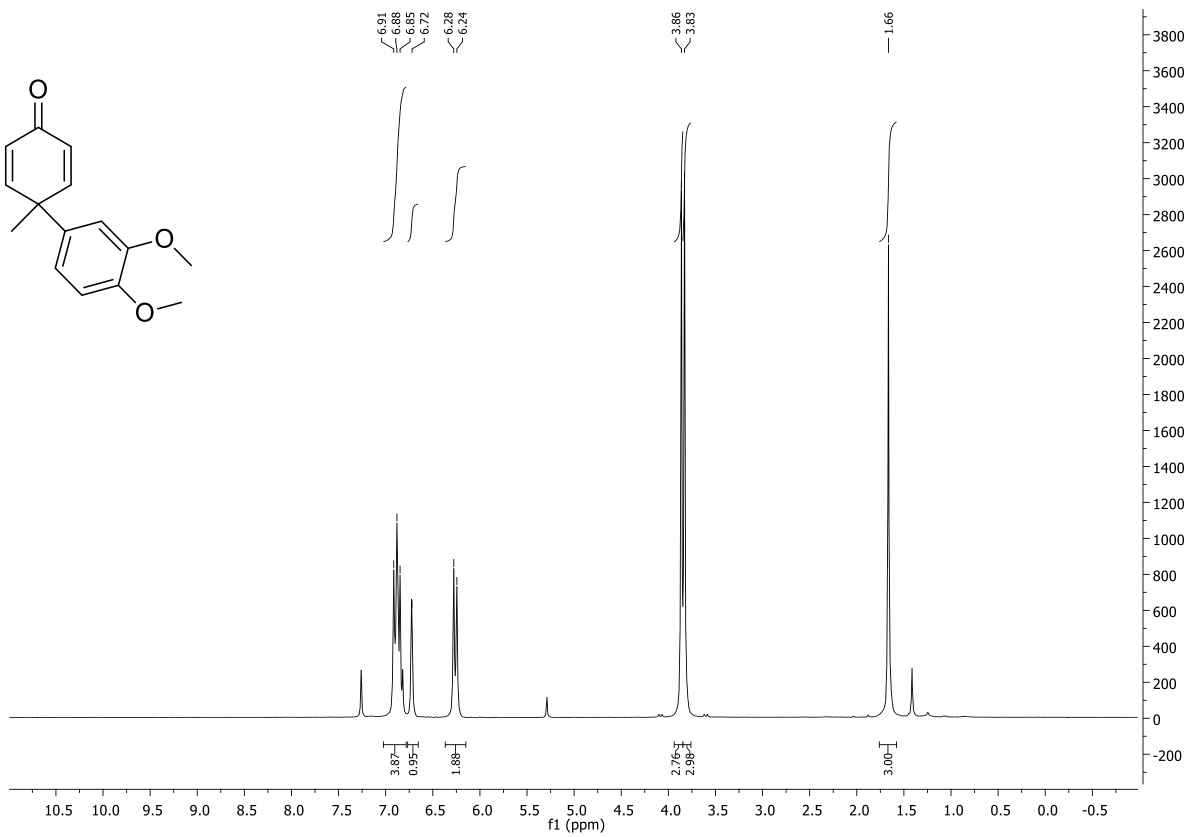

Figure S28. ^{1}H-NMR (300.36 MHz, $CDCl_3$) – **3',4'-Dimethoxy-1-methyl-[1,1'-biphenyl]-4(1*H*)-one (1i)**.

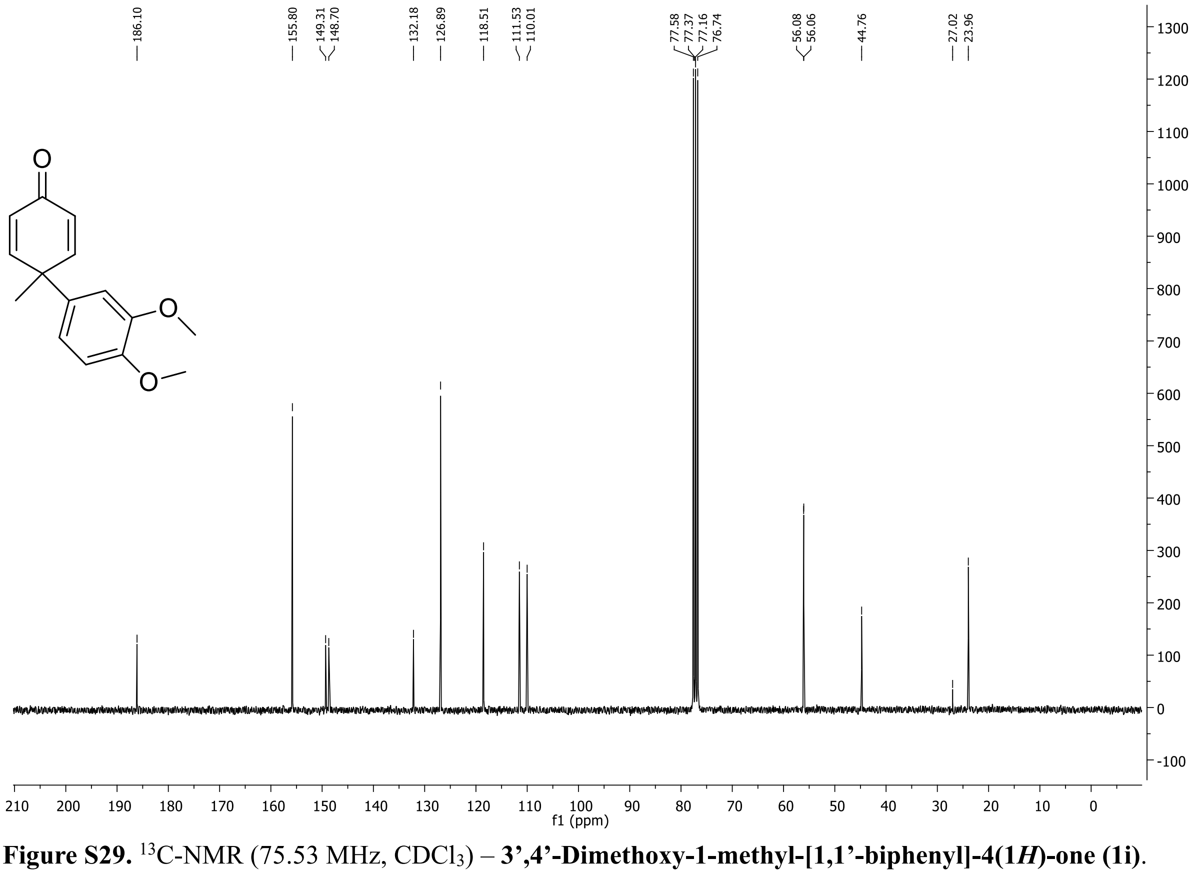

Figure S29. ^{13}C-NMR (75.53 MHz, $CDCl_3$) – **3',4'-Dimethoxy-1-methyl-[1,1'-biphenyl]-4(1*H*)-one (1i)**.

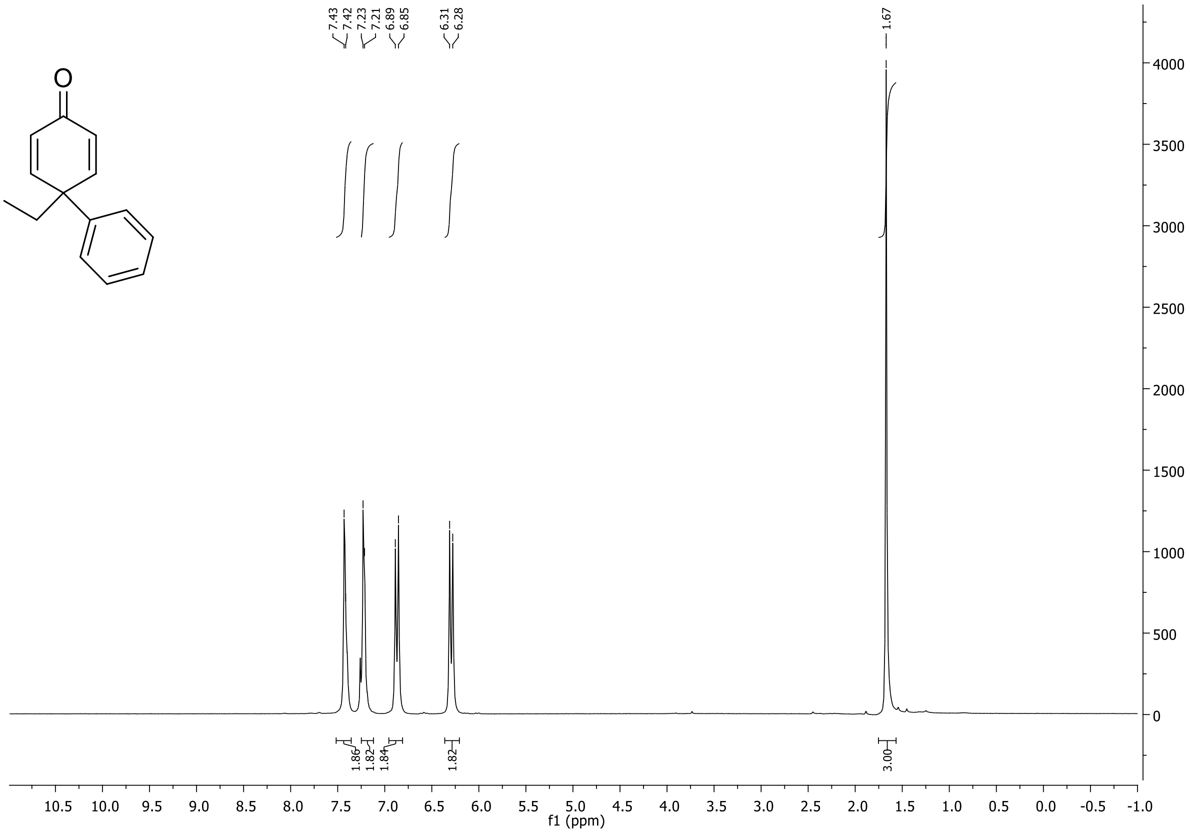

Figure S30. ^{1}H-NMR (300.36 MHz, $CDCl_3$) – **1-Ethyl-[1,1'-biphenyl]-4(1*H*)-one (1j)**.

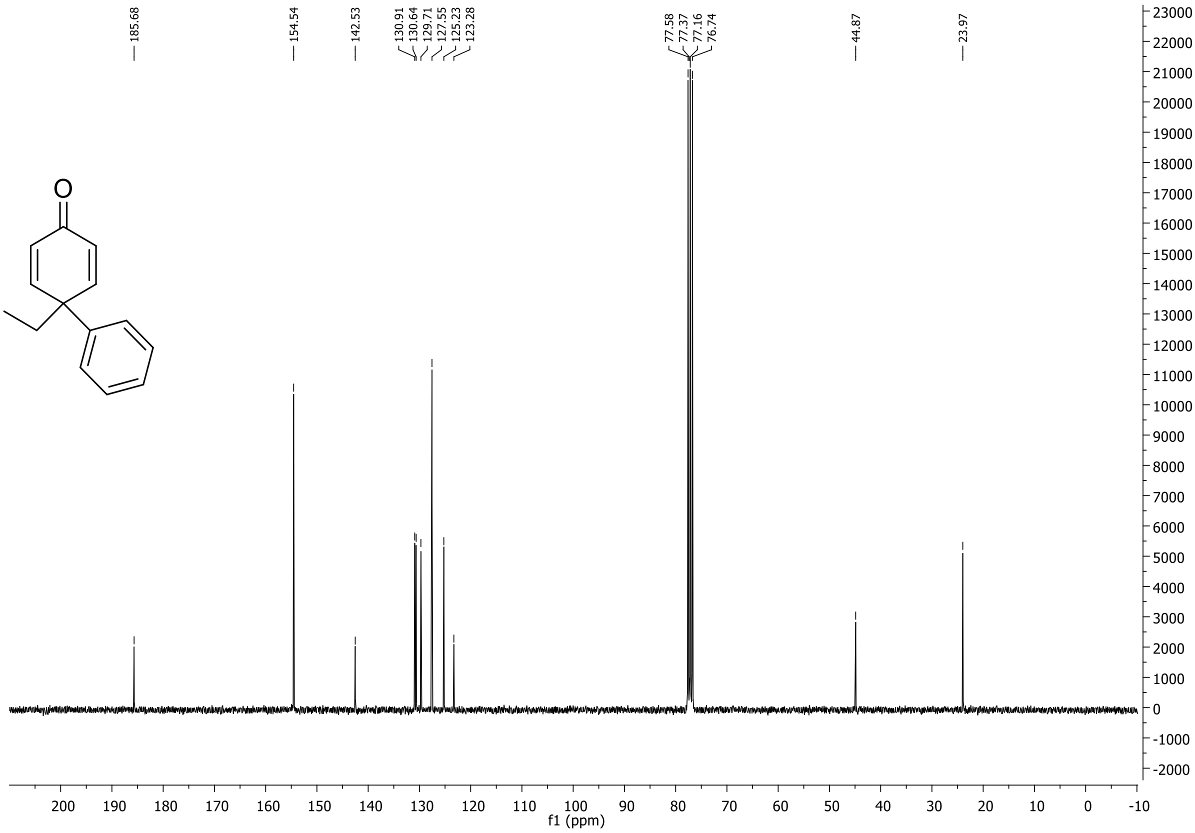

Figure S31. ^{13}C-NMR (75.53 MHz, $CDCl_3$) – **1-Ethyl-[1,1'-biphenyl]-4(1*H*)-one (1j)**.

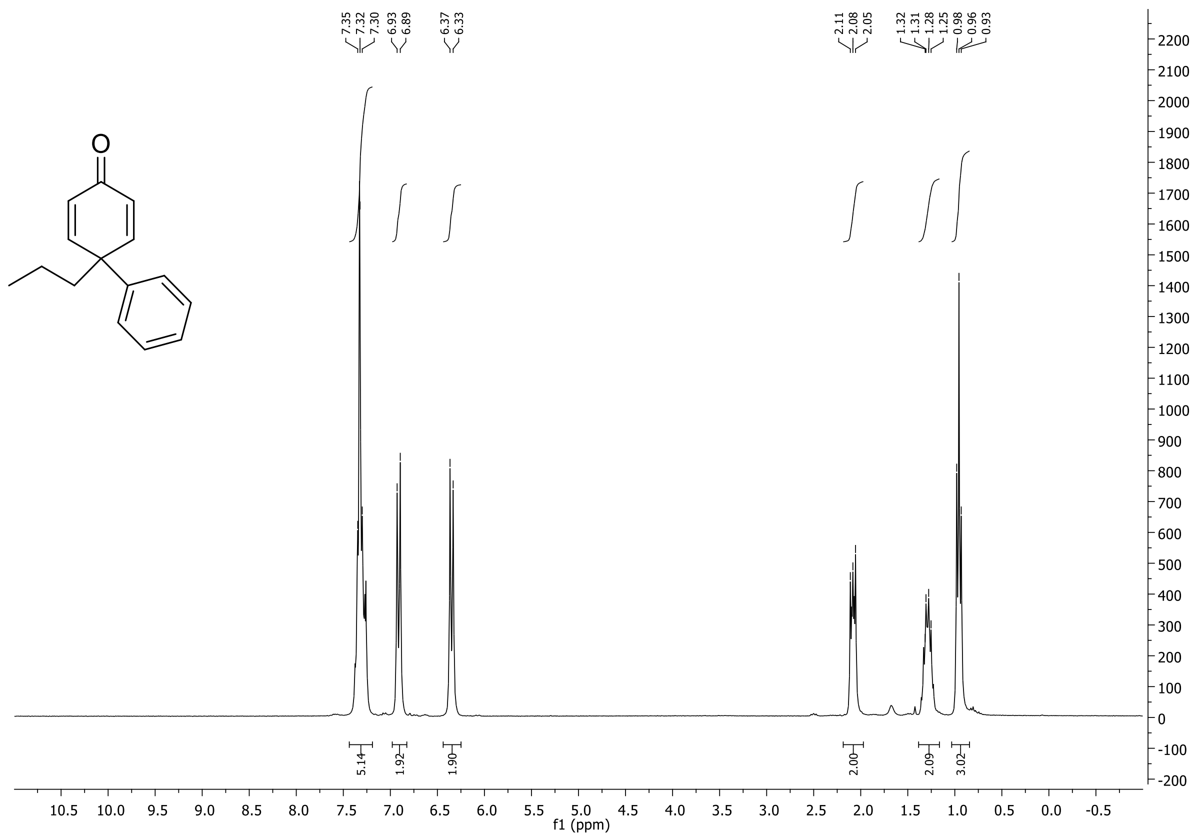

Figure S32. ^{1}H-NMR (300.36 MHz, $CDCl_3$) – **1-Propyl-[1,1'-biphenyl]-4(1*H*)-one (1k)**.

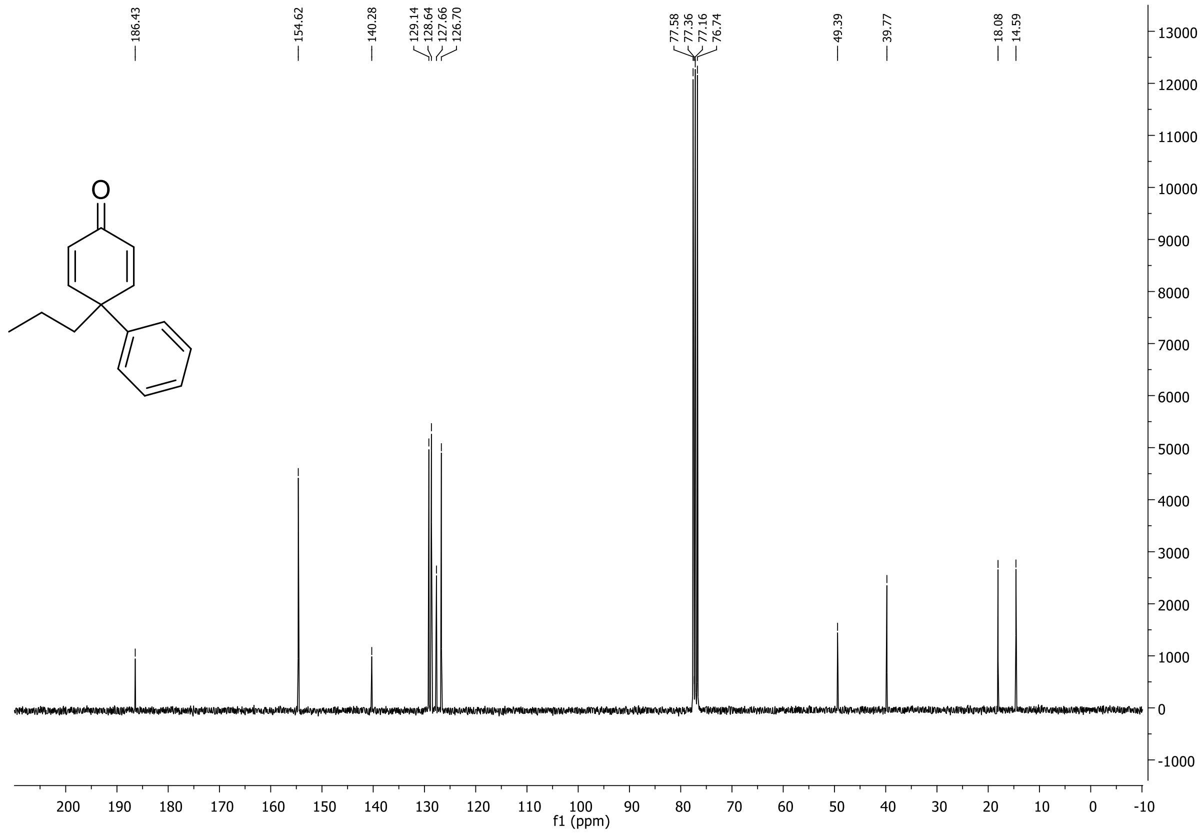

Figure S33. ^{13}C-NMR (75.53 MHz, $CDCl_3$) – **1-Propyl-[1,1'-biphenyl]-4(1*H*)-one (1k)**.

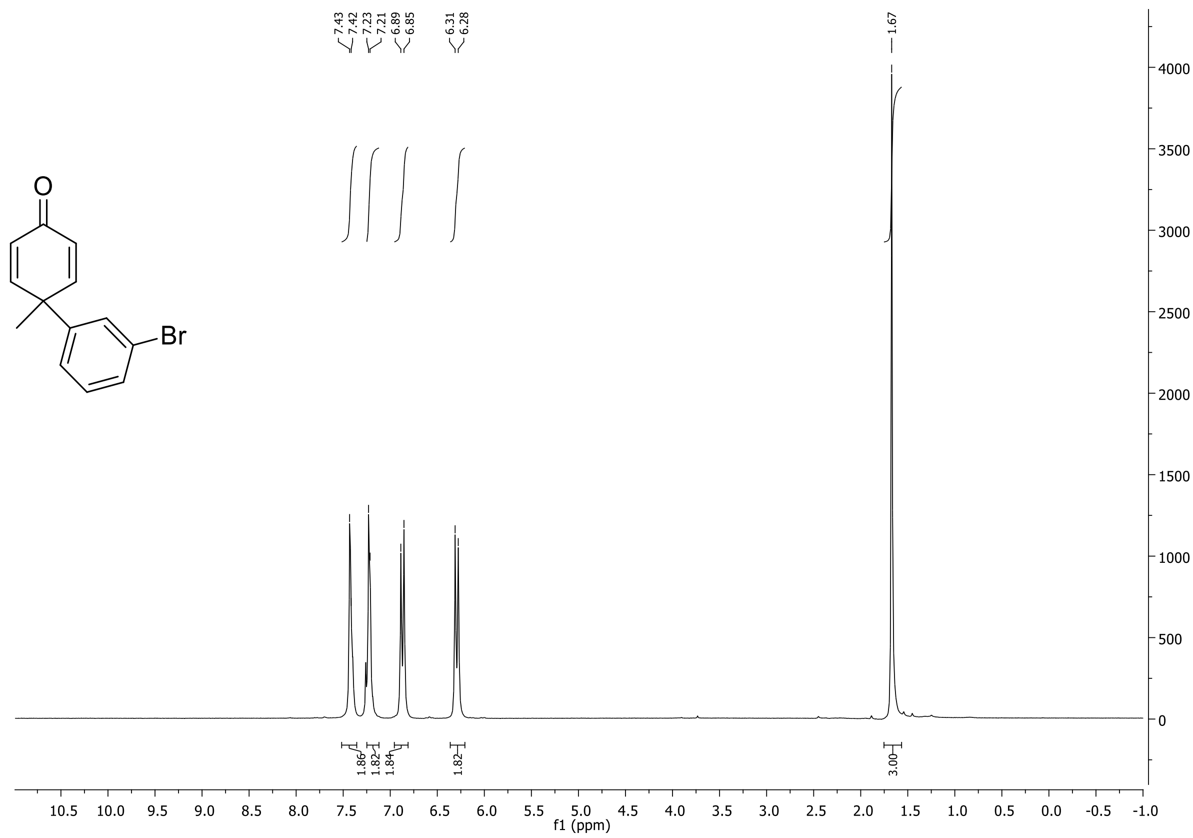

Figure S34. ^{1}H-NMR (300.36 MHz, $CDCl_3$) – **3’-Bromo-1-methyl-[1,1’-biphenyl]-4(1*H*)-one (1l)**.

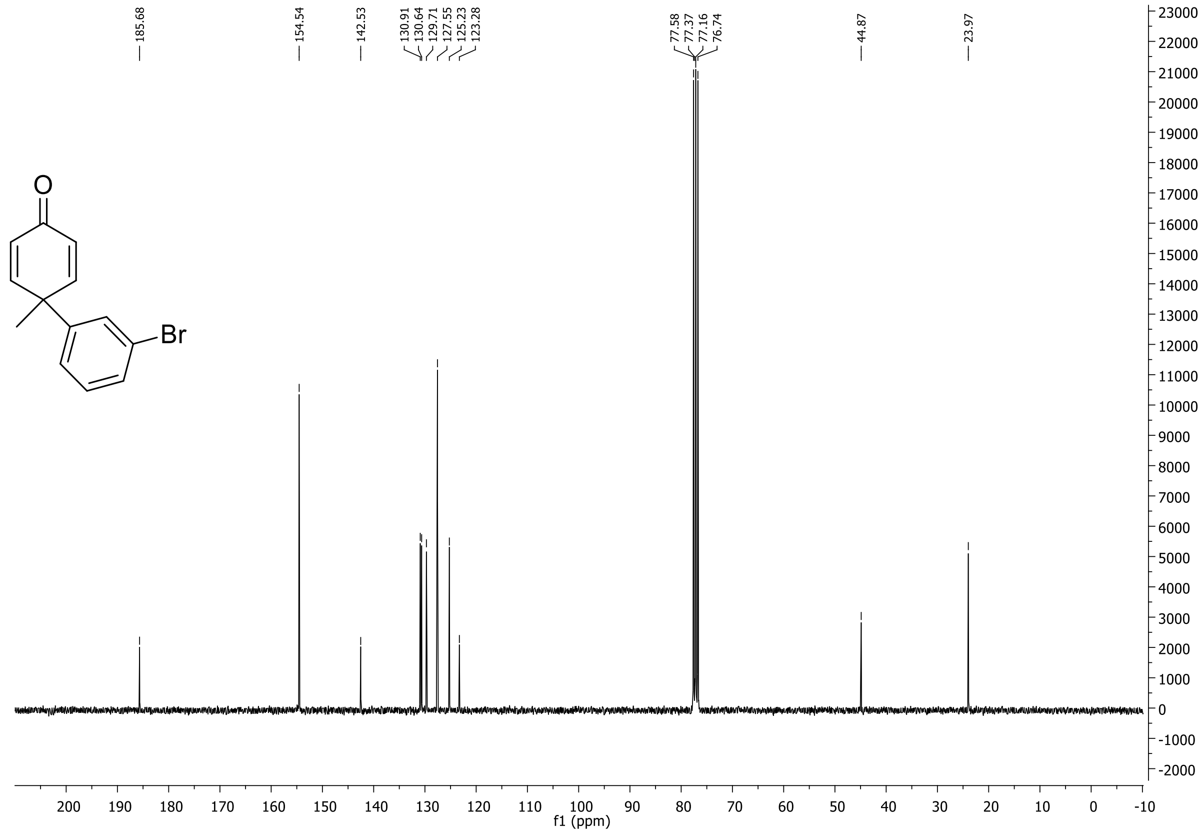

Figure S35. ^{13}C-NMR (75.53 MHz, $CDCl_3$) – **3’-Bromo-1-methyl-[1,1’-biphenyl]-4(1*H*)-one (1l)**.

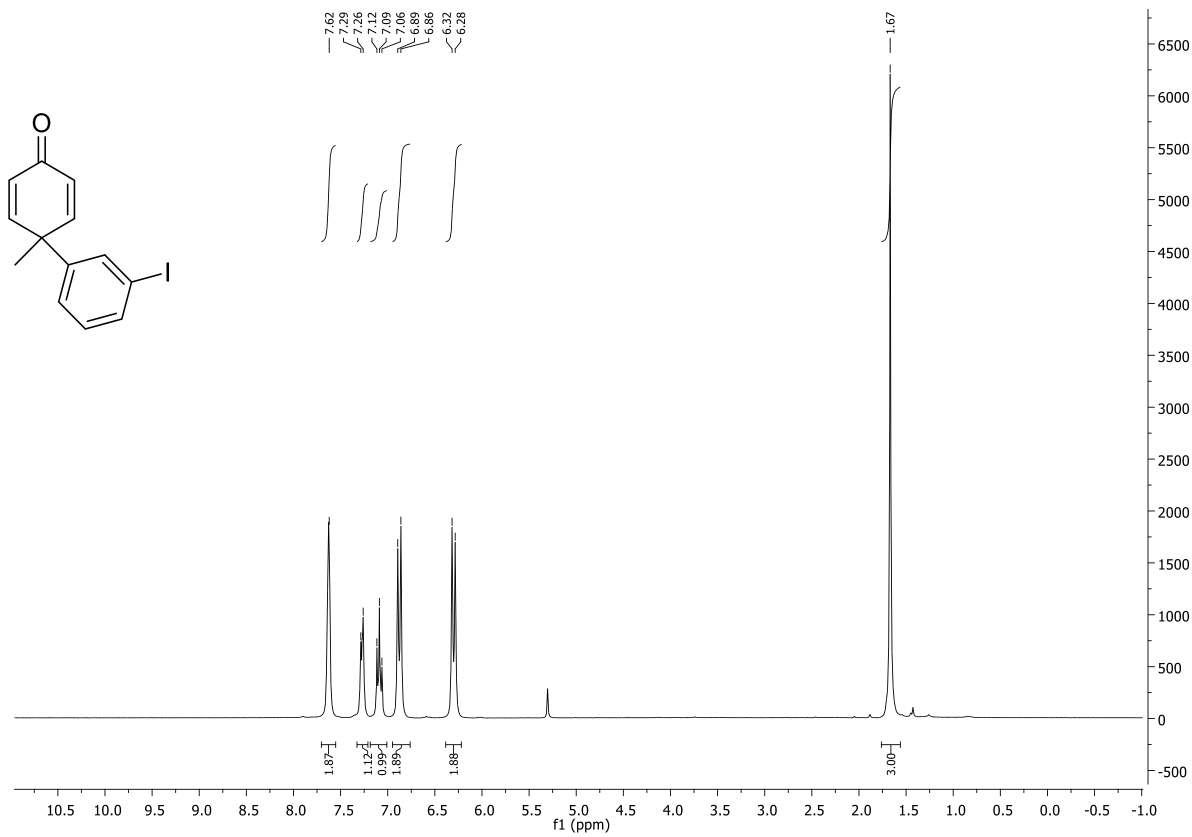

Figure S36. ^{1}H-NMR (300.36 MHz, $CDCl_3$) – **3'-Iodo-1-methyl-[1,1'-biphenyl]-4(1*H*)-one (1m)**.

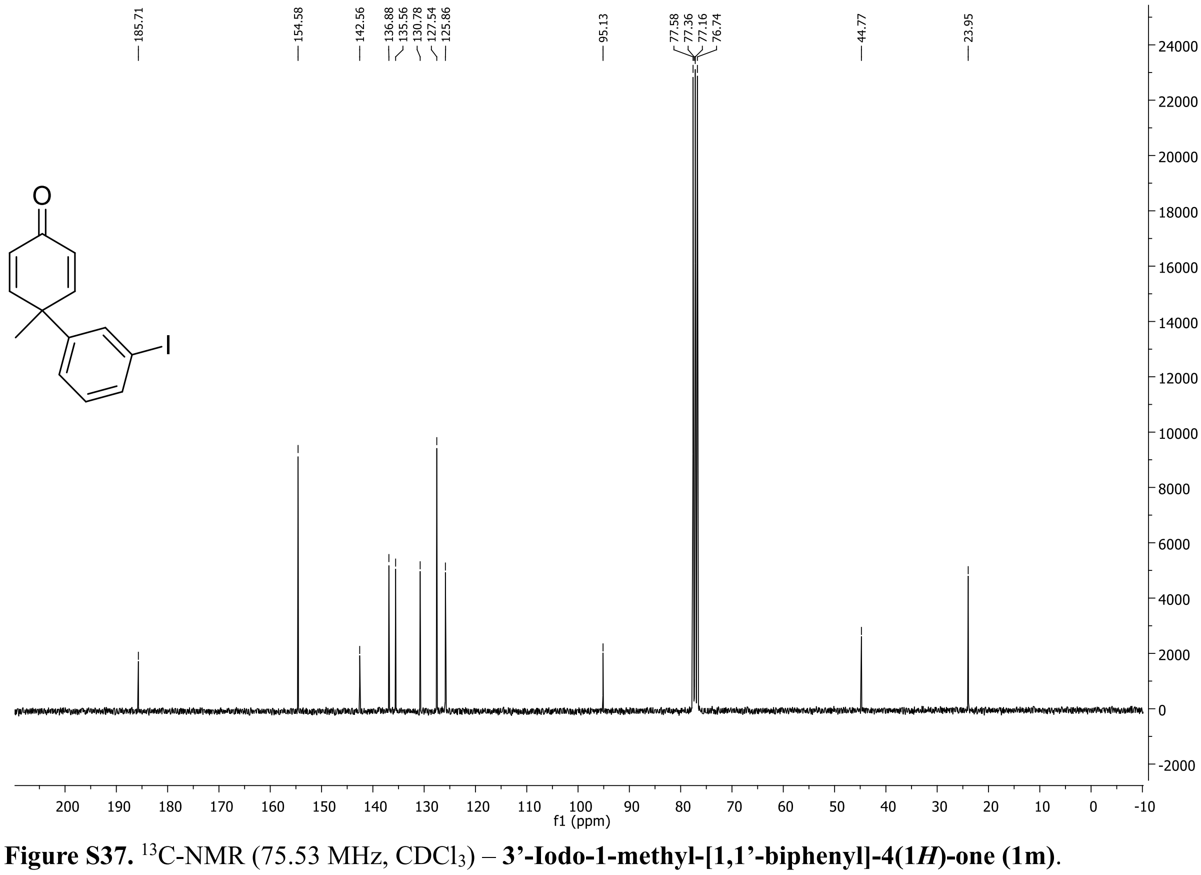

Figure S37. ^{13}C-NMR (75.53 MHz, $CDCl_3$) – **3'-Iodo-1-methyl-[1,1'-biphenyl]-4(1*H*)-one (1m)**.

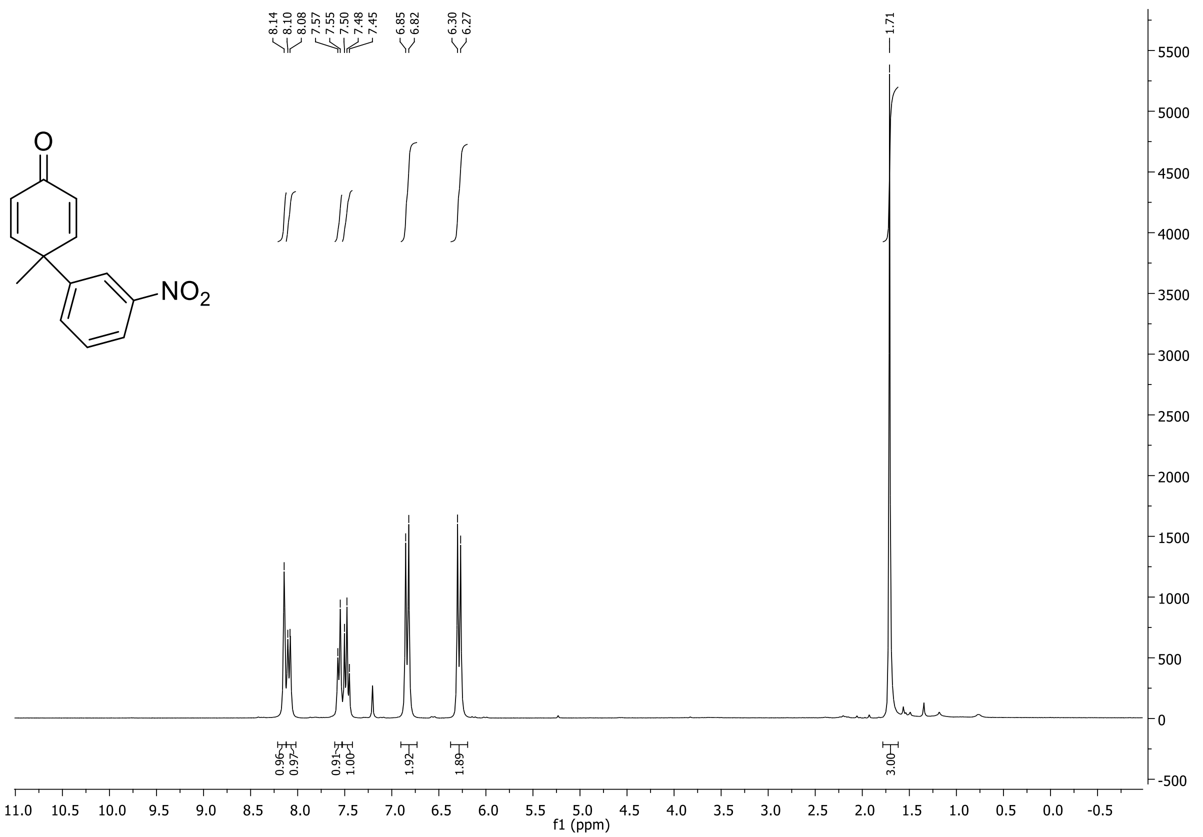

Figure S38. ^{1}H-NMR (300.36 MHz, $CDCl_3$) – **1-Methyl-3'-nitro-[1,1'-biphenyl]-4(1*H*)-one (1n)**.

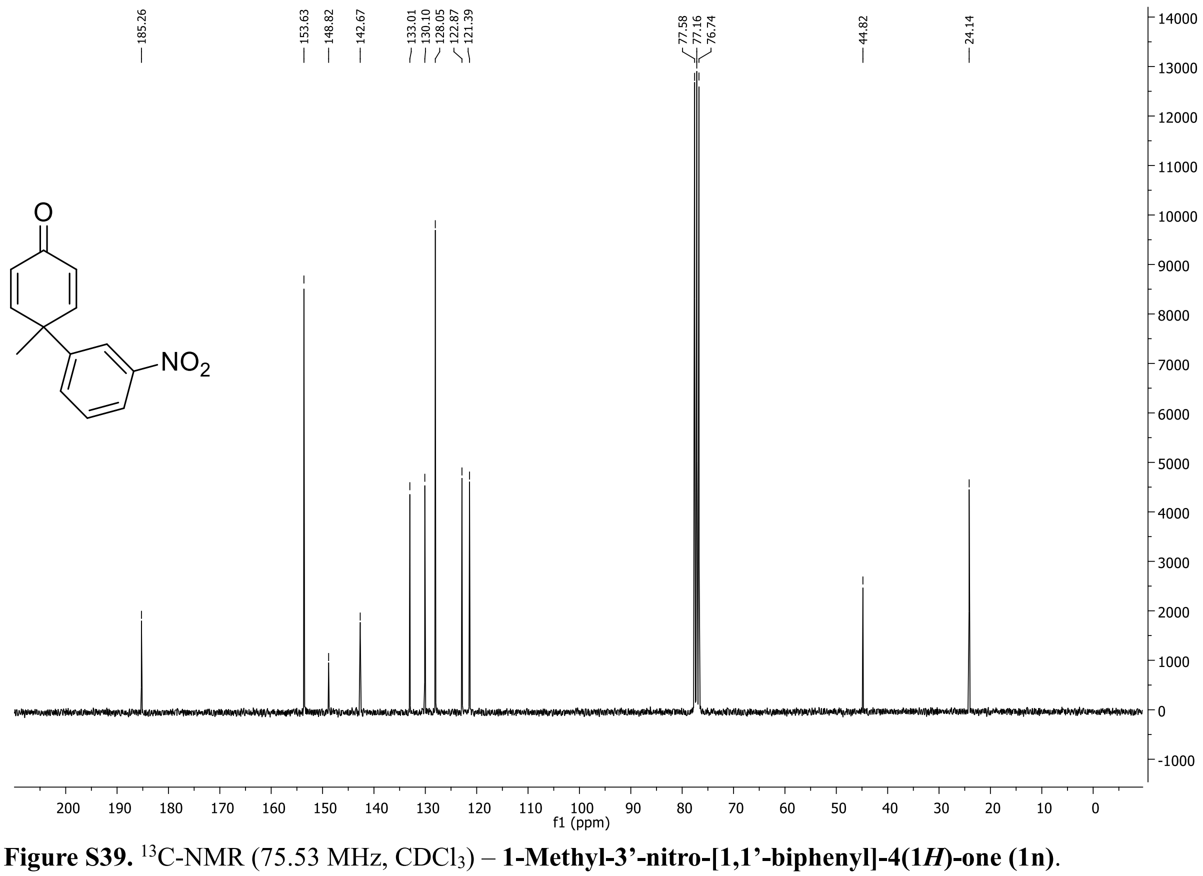

Figure S39. ^{13}C-NMR (75.53 MHz, $CDCl_3$) – **1-Methyl-3'-nitro-[1,1'-biphenyl]-4(1*H*)-one (1n)**.

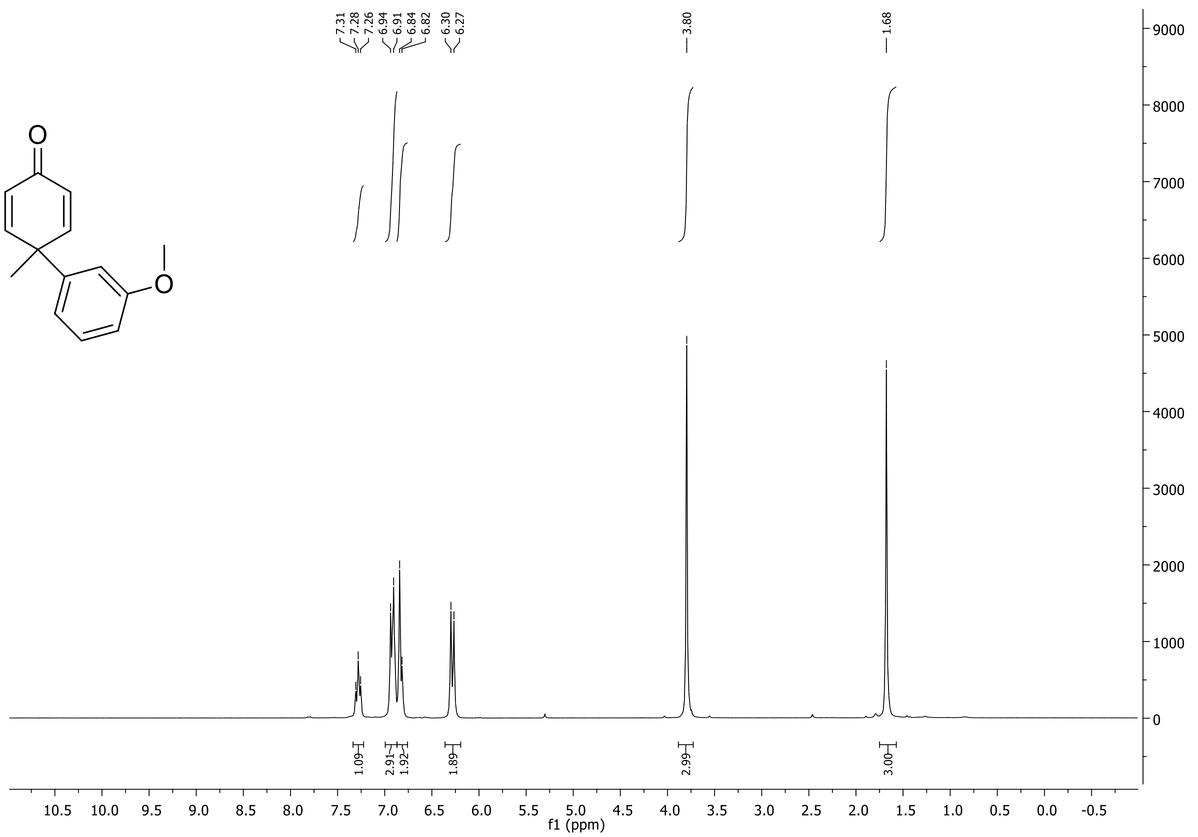

Figure S40. ^{1}H-NMR (300.36 MHz, $CDCl_3$) – **3'-Methoxy-1-methyl-[1,1'-biphenyl]-4(1*H*)-one (1o)**.

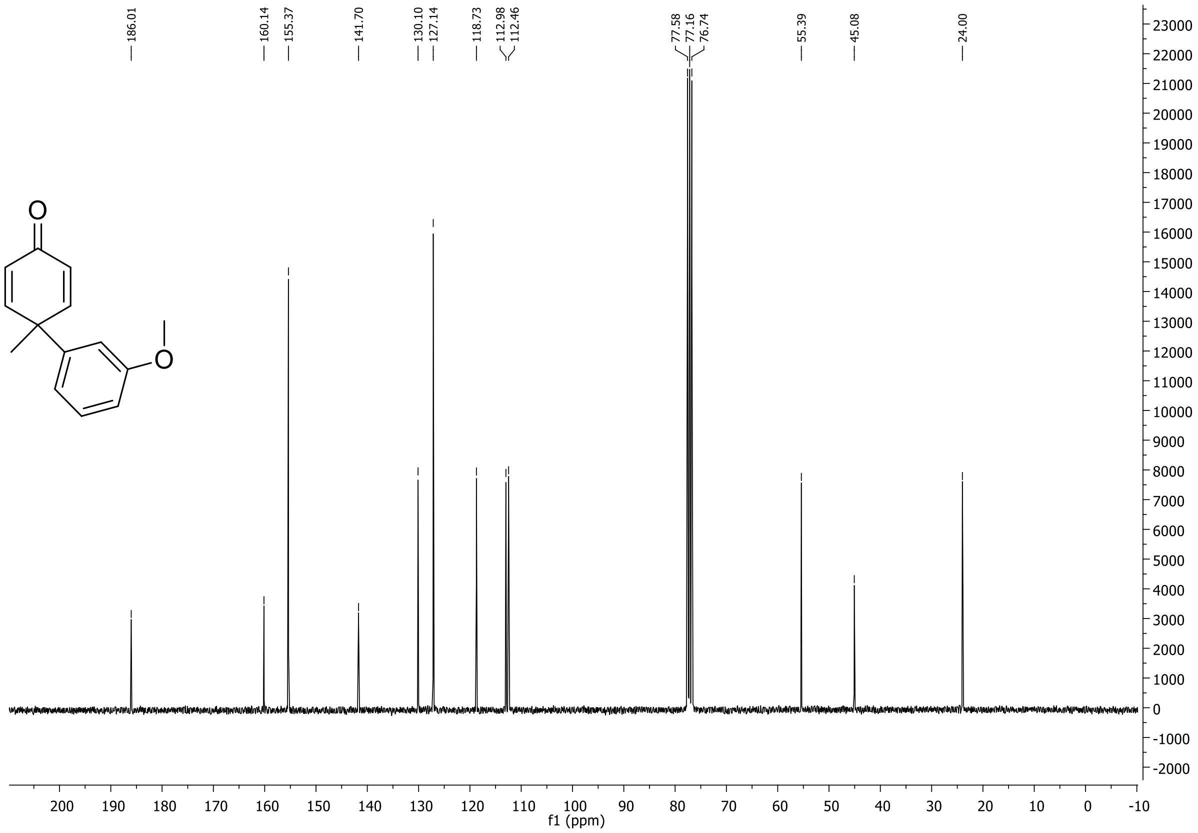

Figure S41. ^{13}C-NMR (75.53 MHz, $CDCl_3$) – **3'-Methoxy-1-methyl-[1,1'-biphenyl]-4(1*H*)-one (1o)**.

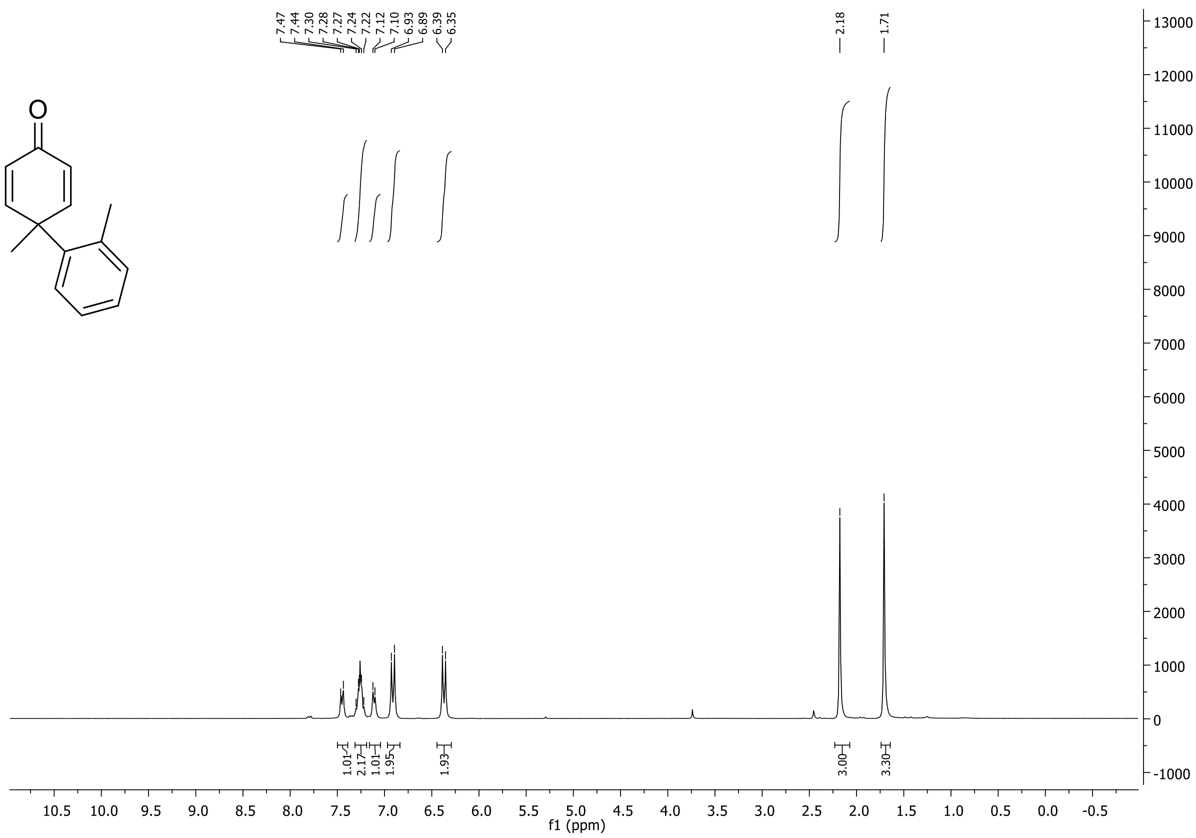

Figure S42. ^{1}H-NMR (300.36 MHz, $CDCl_3$) – **1,2'-Dimethyl-[1,1'-biphenyl]-4(1*H*)-one (1p)**.

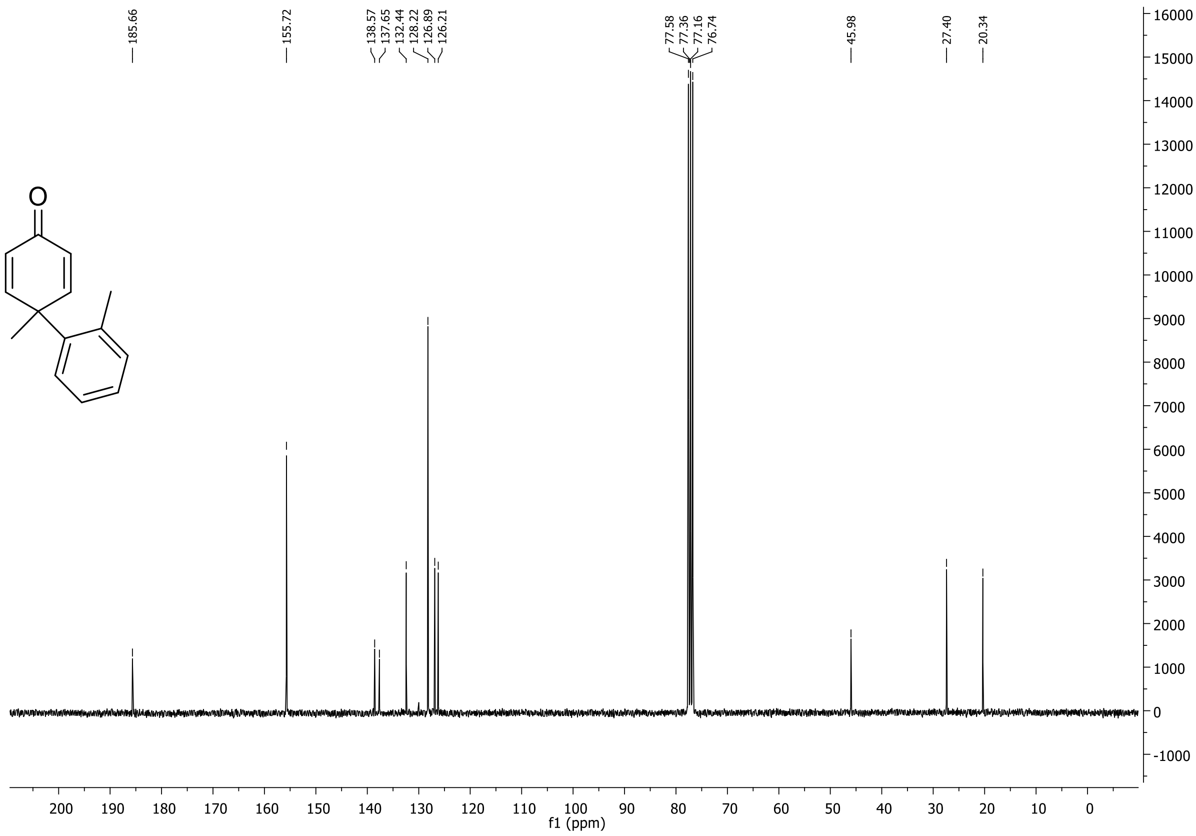

Figure S43. ^{13}C-NMR (75.53 MHz, $CDCl_3$) – **1,2'-Dimethyl-[1,1'-biphenyl]-4(1*H*)-one (1p)**.

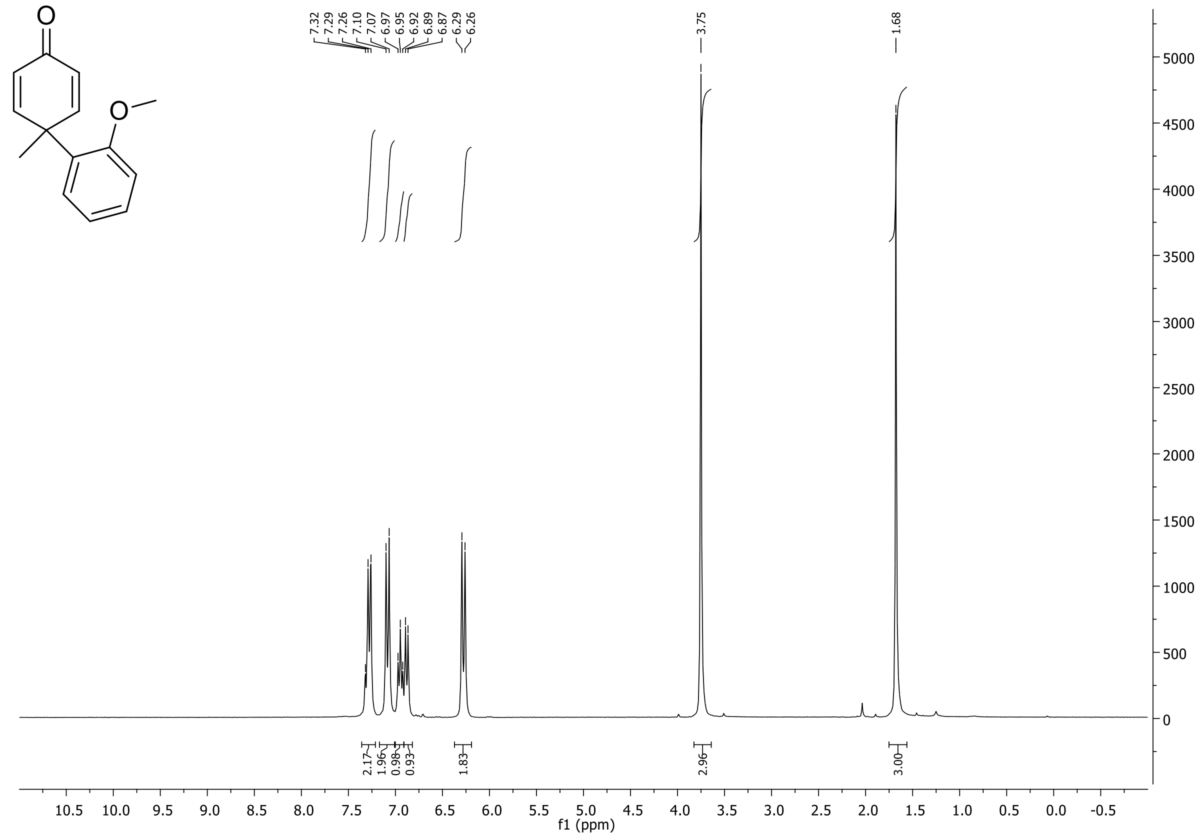

Figure S44. ^{1}H-NMR (300.36 MHz, $CDCl_3$) – **2'-Methoxy-1-methyl-1[1,1'-biphenyl]-4(1*H*)-one (1q)**.

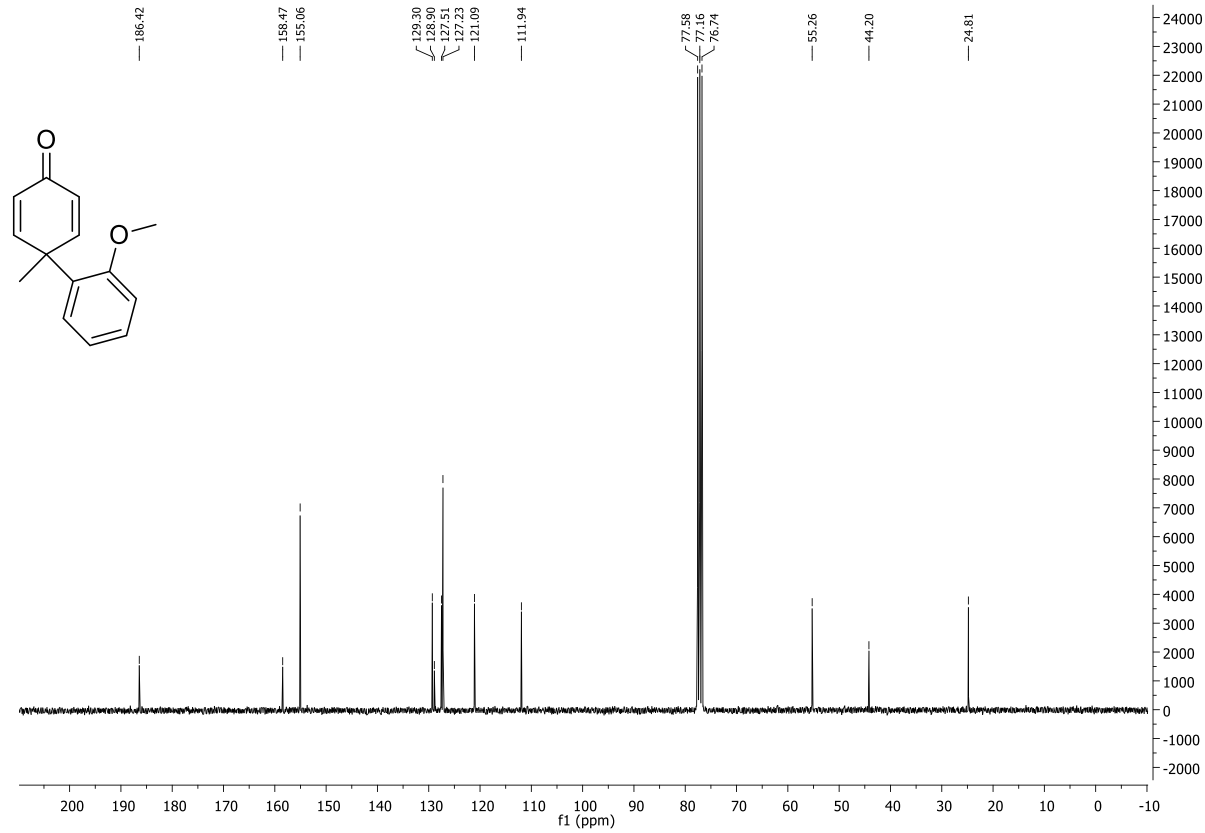

Figure S45. ^{13}C-NMR (75.53 MHz, $CDCl_3$) – **2'-Methoxy-1-methyl-1[1,1'-biphenyl]-4(1*H*)-one (1q)**.

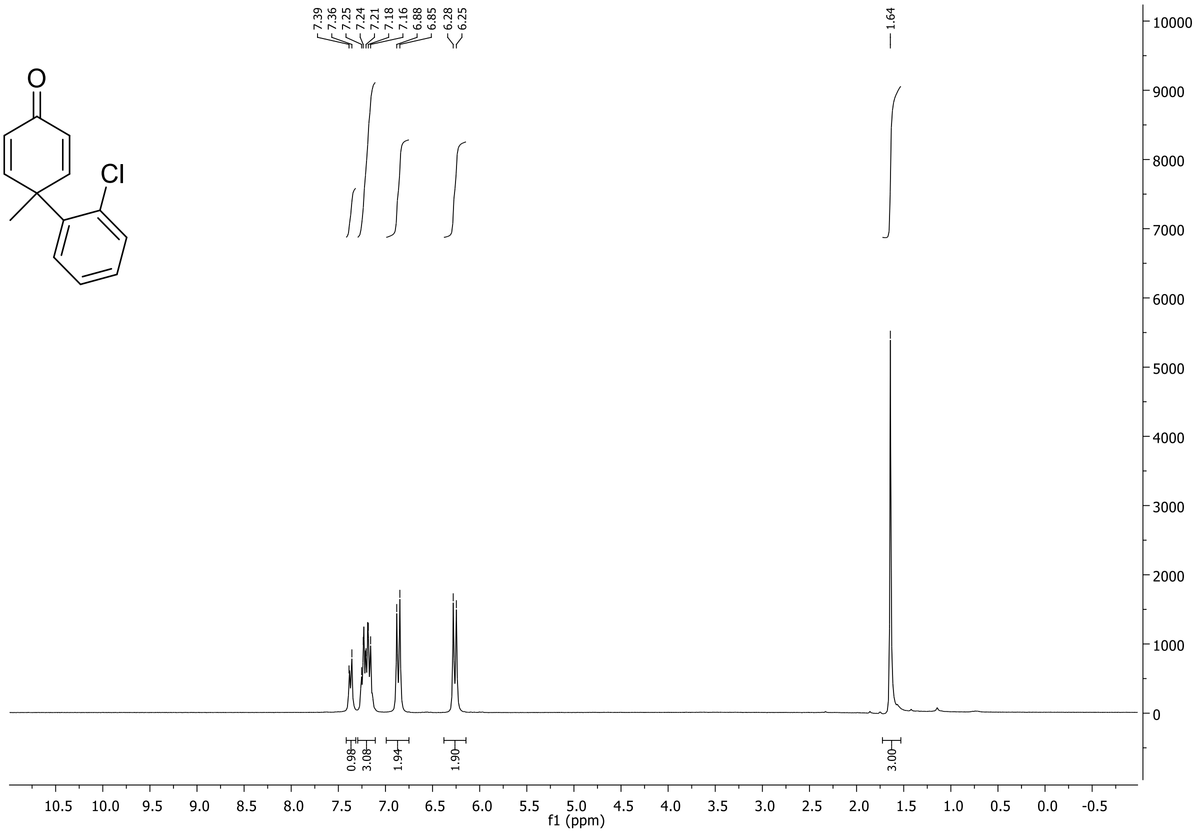

Figure S46. ^{1}H-NMR (300.36 MHz, $CDCl_3$) – **2’-Chloro-1-methyl-[1,1’-biphenyl]-4(1*H*)-one (1r)**.

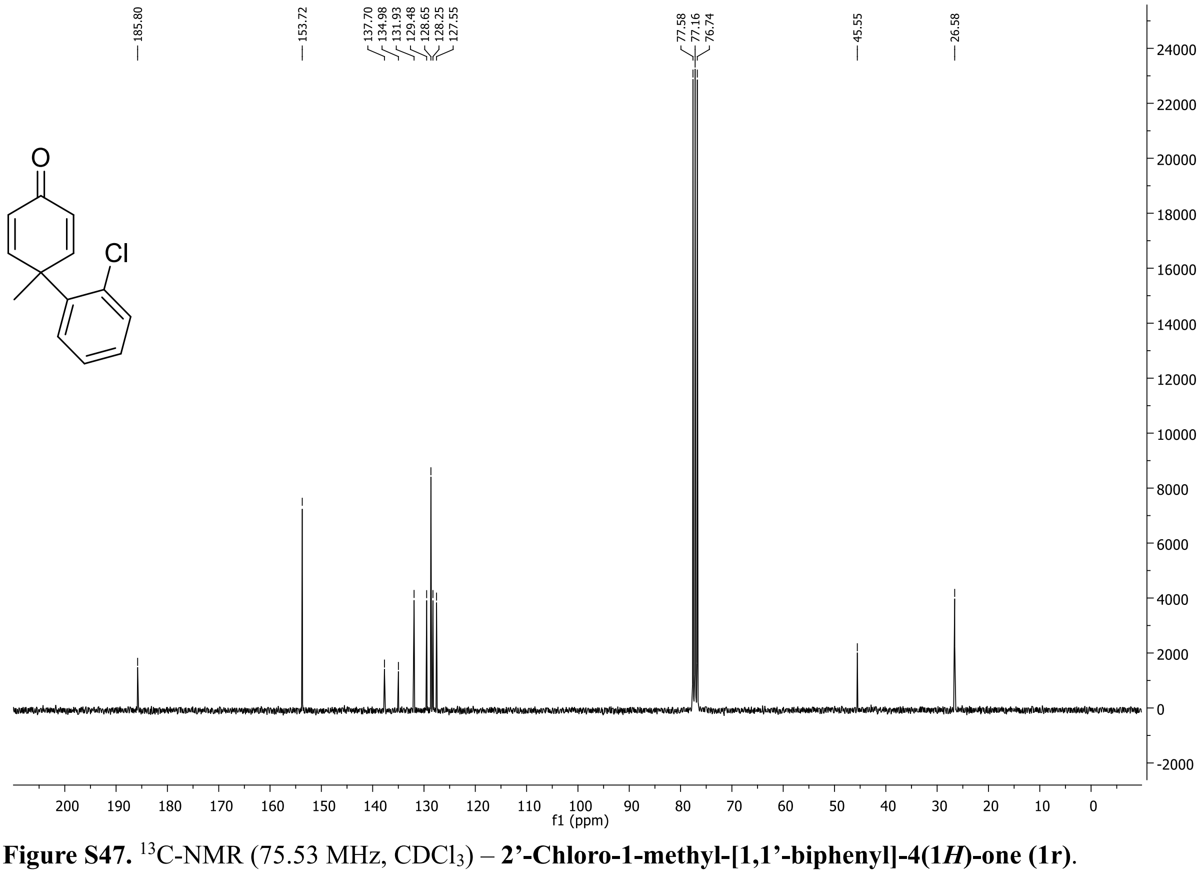

Figure S47. ^{13}C-NMR (75.53 MHz, $CDCl_3$) – **2’-Chloro-1-methyl-[1,1’-biphenyl]-4(1*H*)-one (1r)**.

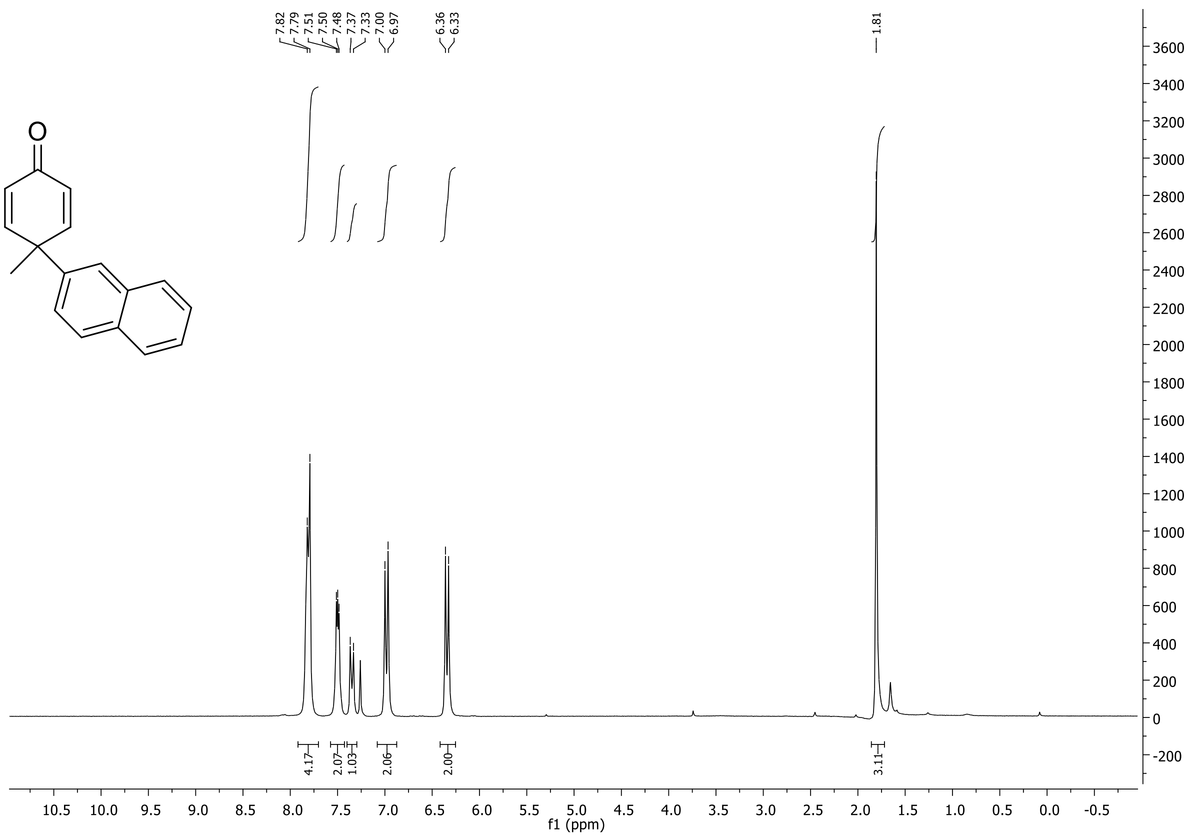

Figure S48. ^{1}H-NMR (300.36 MHz, $CDCl_3$) – **4-Methyl-4-(naphthalen-2-yl)cyclohexa-2,5-dienone (1s)**.

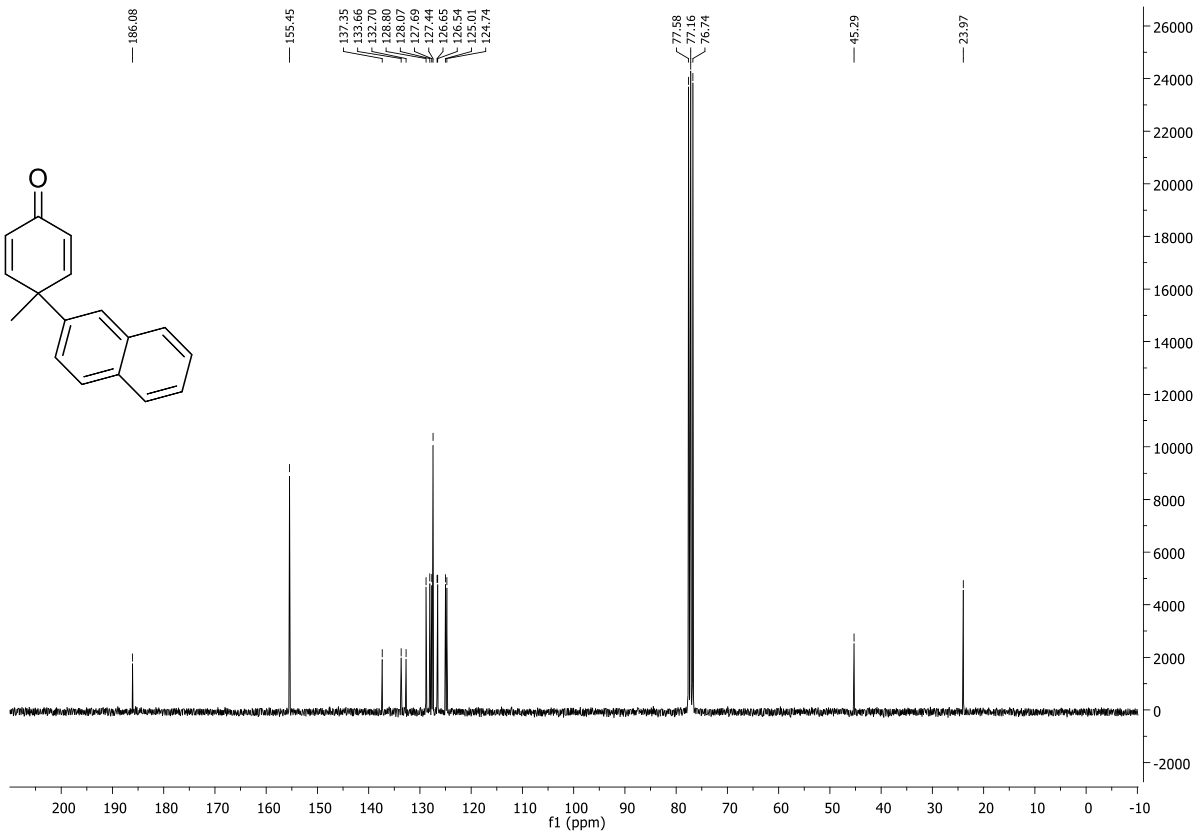

Figure S49. ^{13}C-NMR (75.53 MHz, $CDCl_3$) – **4-Methyl-4-(naphthalen-2-yl)cyclohexa-2,5-dienone (1s)**.

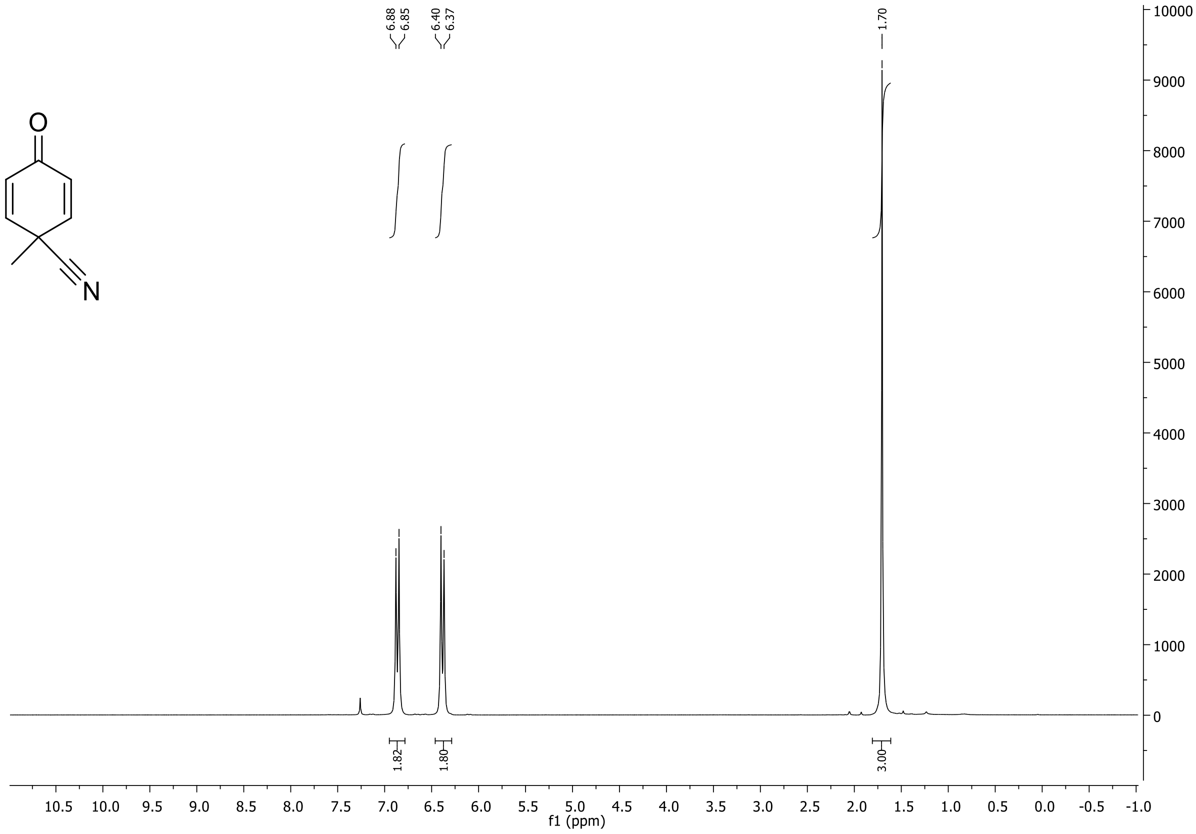

Figure S50. ^{1}H-NMR (300.36 MHz, $CDCl_3$) – **1-Methyl-4-oxocyclohexa-2,5-dienecarbonitrile (1t)**.

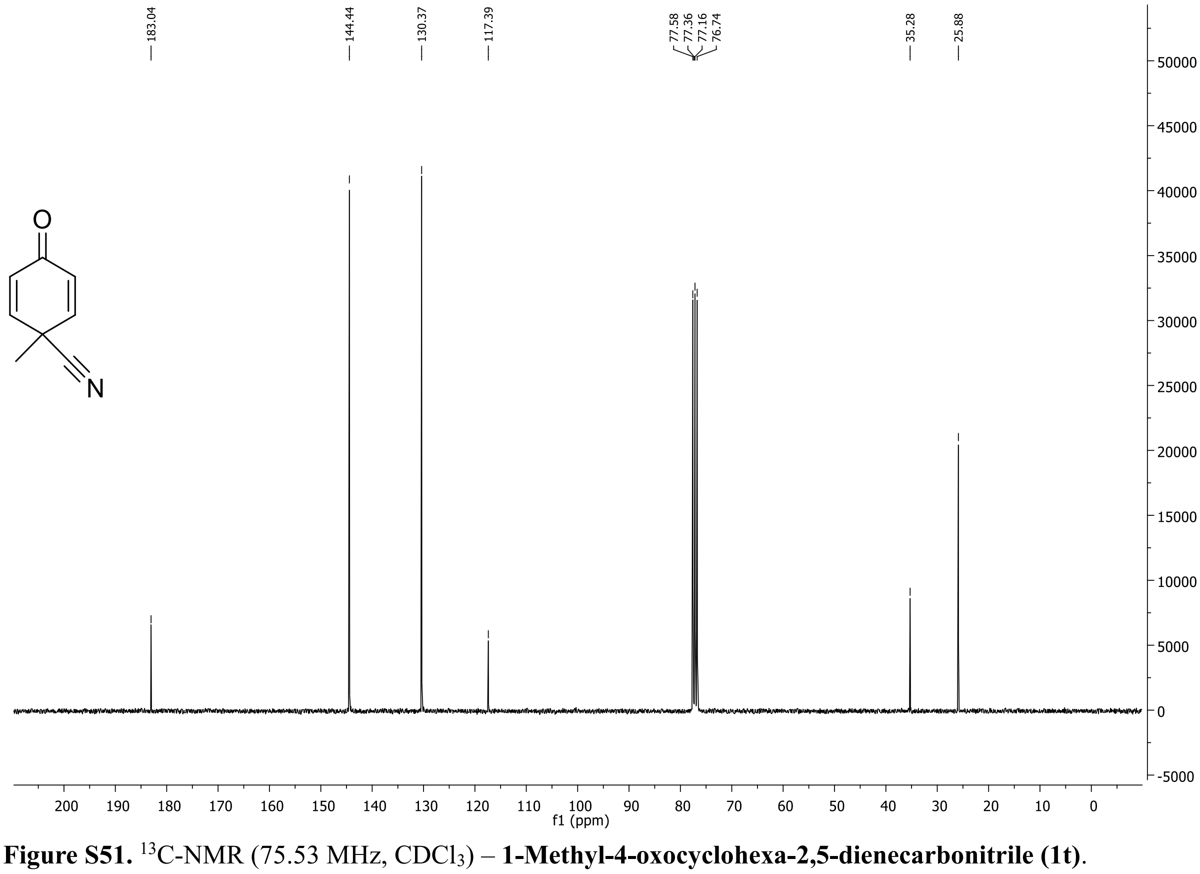

Figure S51. ^{13}C-NMR (75.53 MHz, $CDCl_3$) – **1-Methyl-4-oxocyclohexa-2,5-dienecarbonitrile (1t)**.

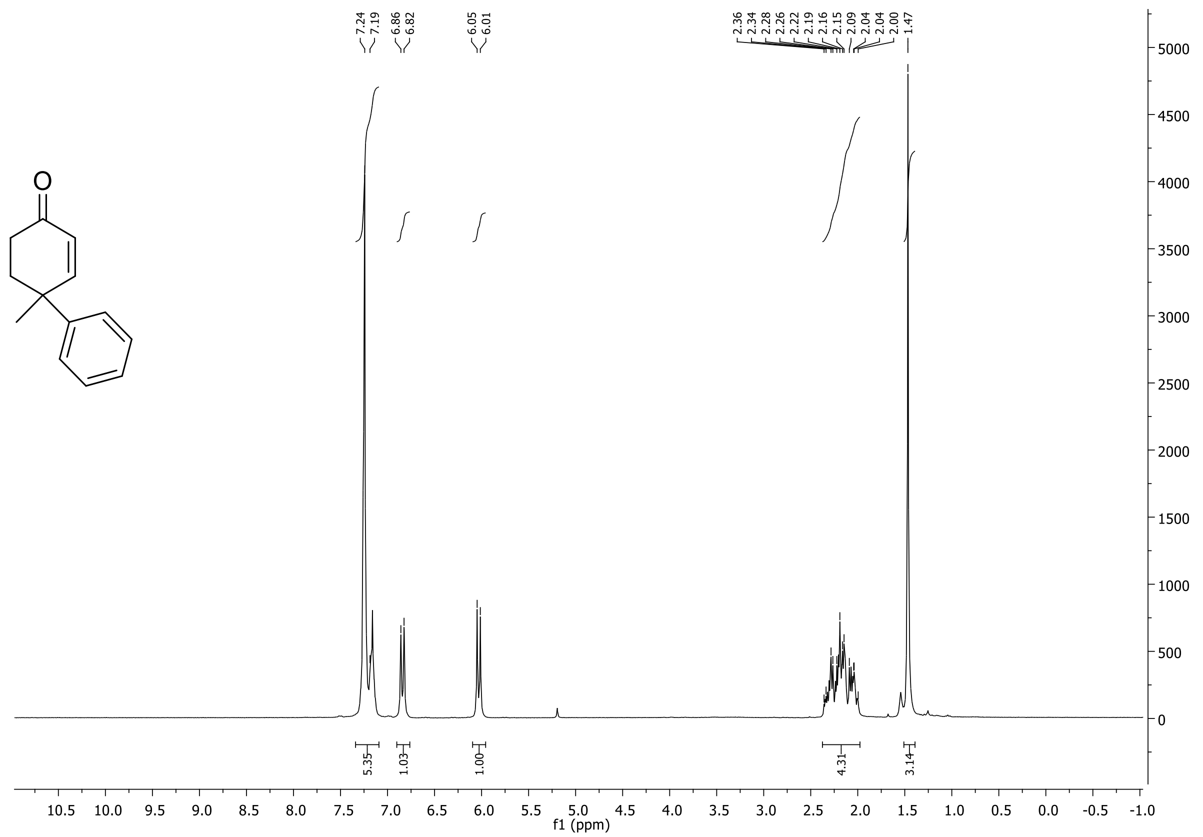

Figure S52. ^{1}H-NMR (300.36 MHz, $CDCl_3$) – **1-Methyl-2,3-dihydro-[1,1'-biphenyl]-4(1*H*)-one (rac-2a)**.

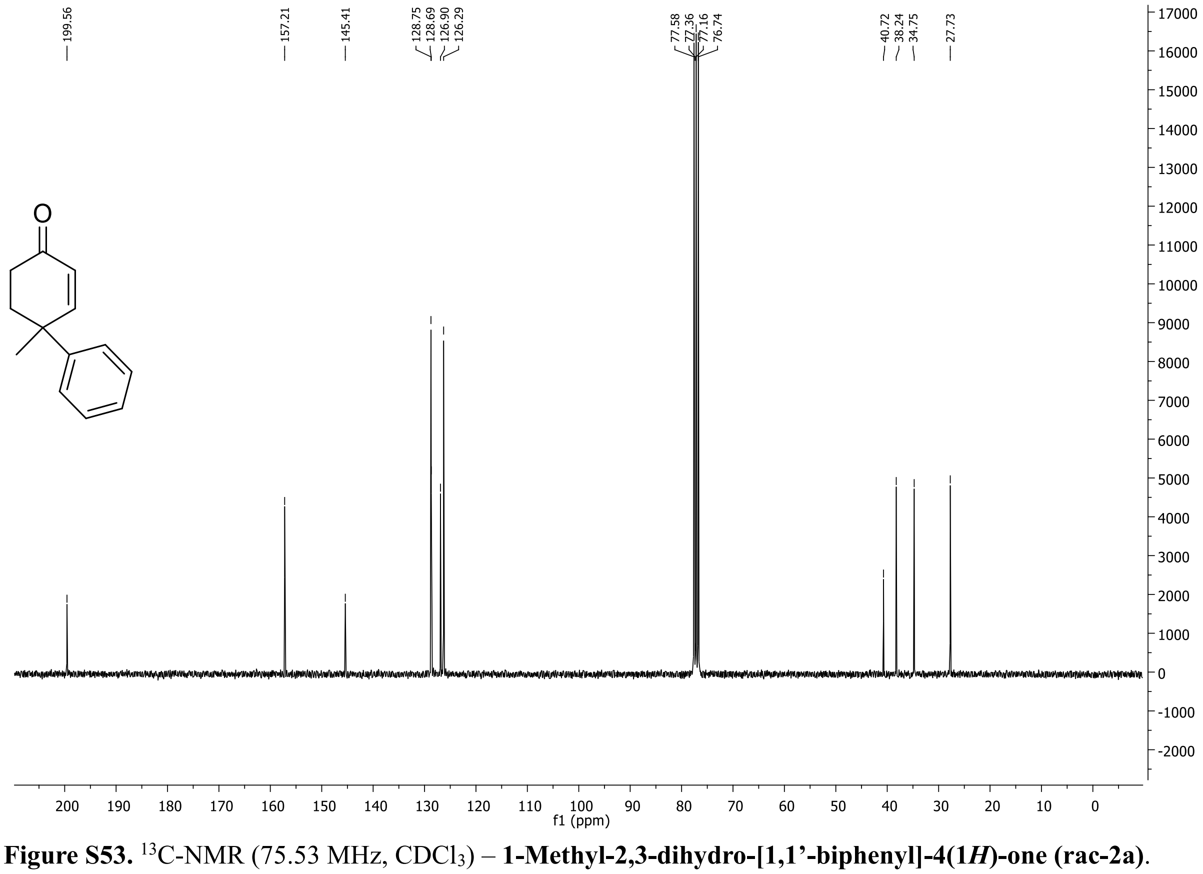

Figure S53. ^{13}C-NMR (75.53 MHz, $CDCl_3$) – **1-Methyl-2,3-dihydro-[1,1'-biphenyl]-4(1*H*)-one (rac-2a)**.

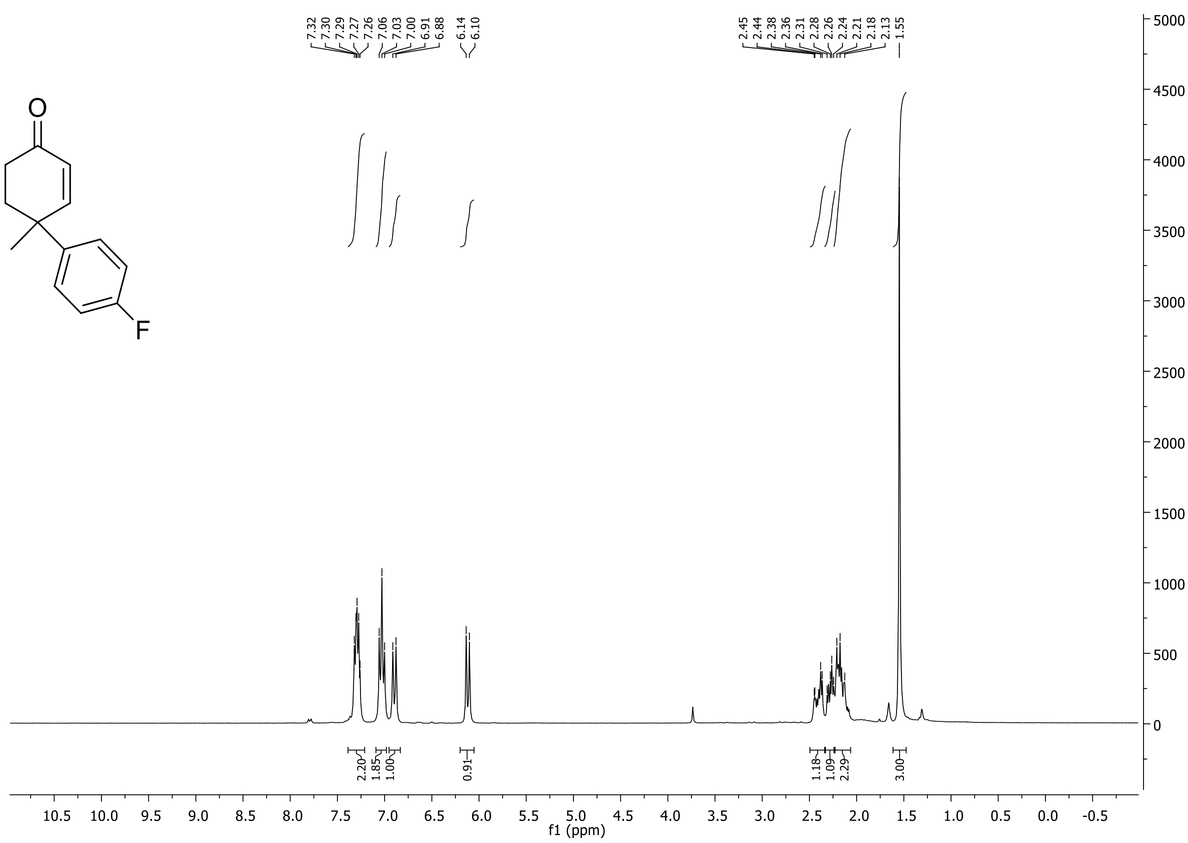

Figure S54. ^{1}H-NMR (300.36 MHz, $CDCl_3$) – **4’-Fluoro-1-methyl-2,3-dihydro-[1,1’-biphenyl]-4(1*H*)-one (rac-2b)**.

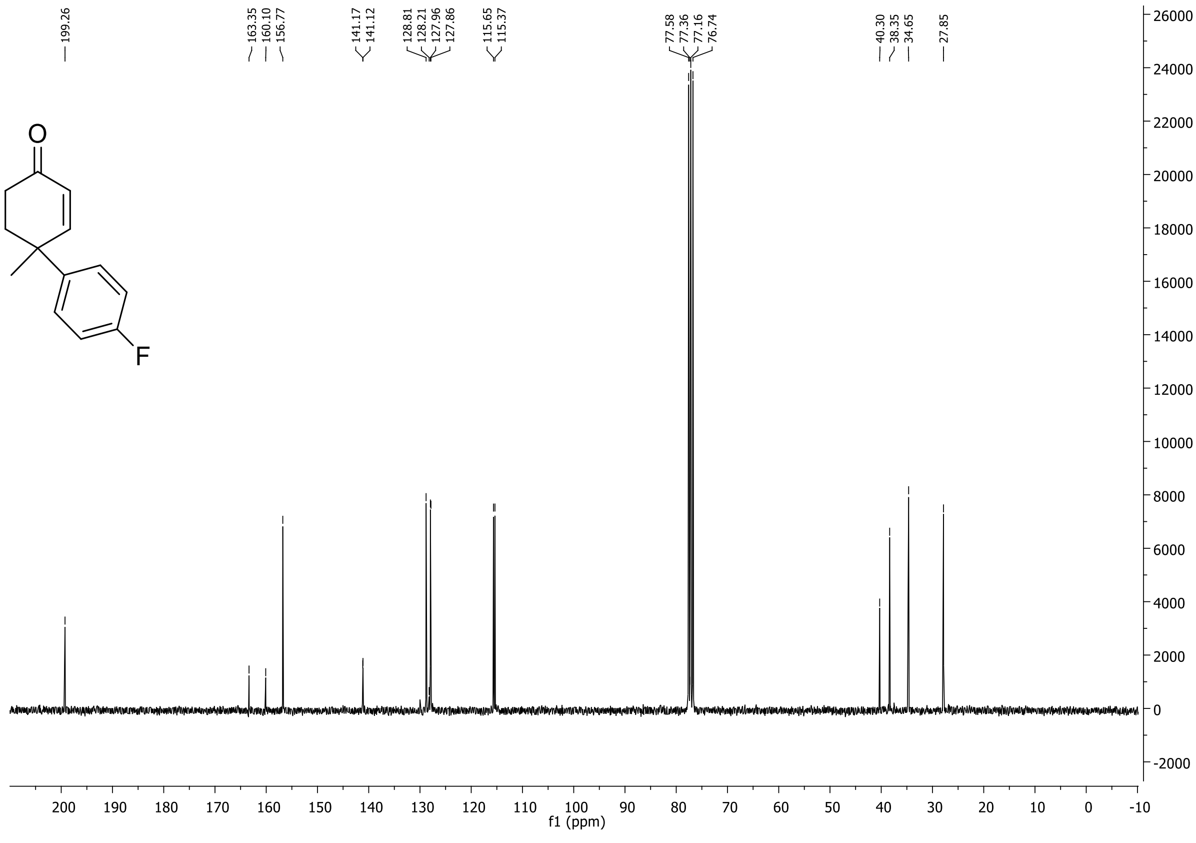

Figure S55. ^{13}C-NMR (75.53 MHz, $CDCl_3$) – **4’-Fluoro-1-methyl-2,3-dihydro-[1,1’-biphenyl]-4(1*H*)-one (rac-2b)**.

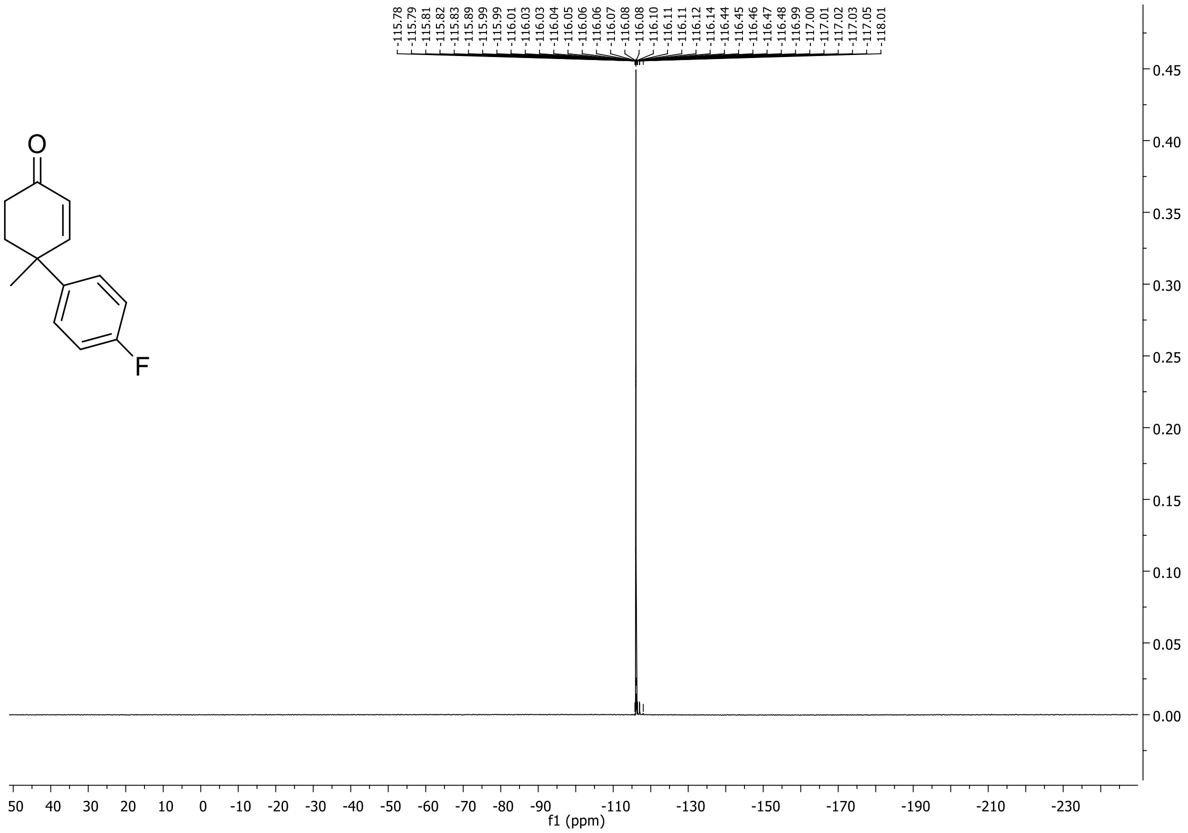

Figure S56. ^{19}F-NMR (376.17 MHz, $CDCl_3$) – **4'-Fluoro-1-methyl-2,3-dihydro-[1,1'-biphenyl]-4(1*H*)-one (rac-2b)**.

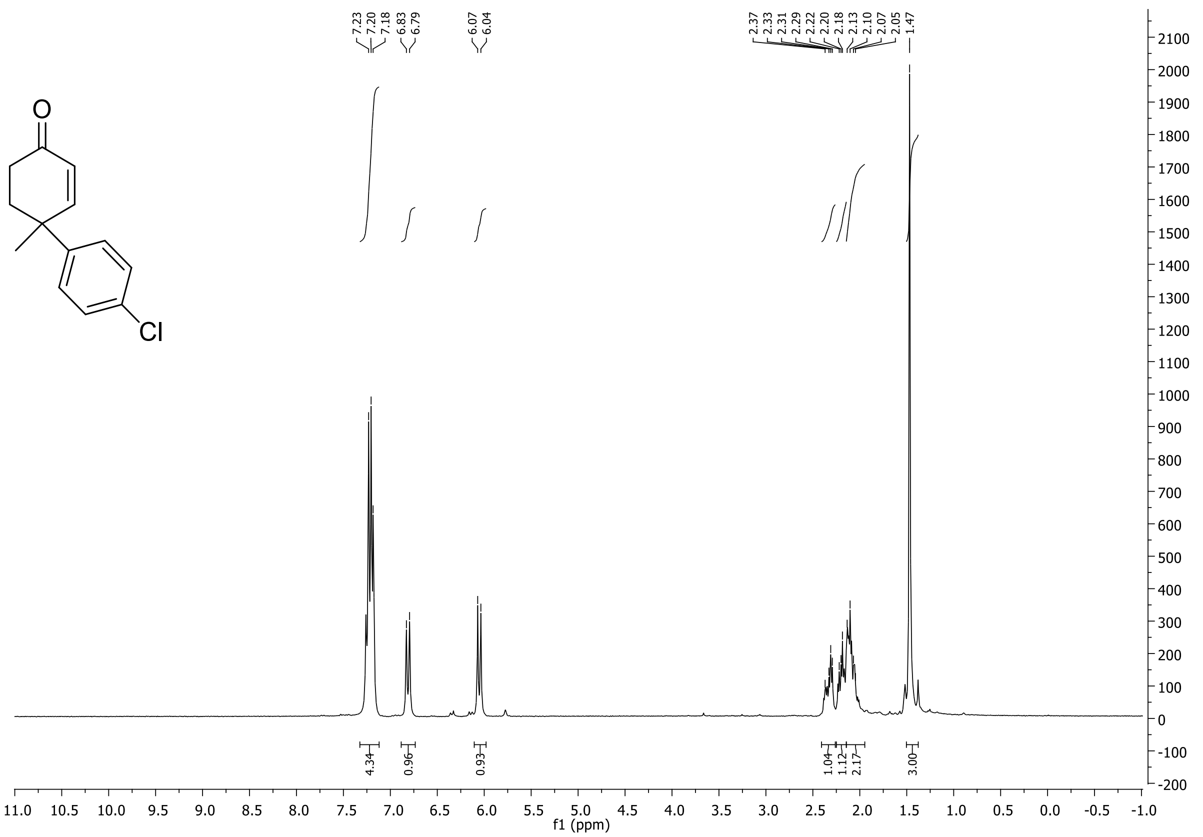

Figure S57. ^{1}H-NMR (300.36 MHz, $CDCl_3$) – **4’-Chloro-1-methyl-2,3-dihydro-[1,1’-biphenyl]-4(1*H*)-one (rac-2c)**.

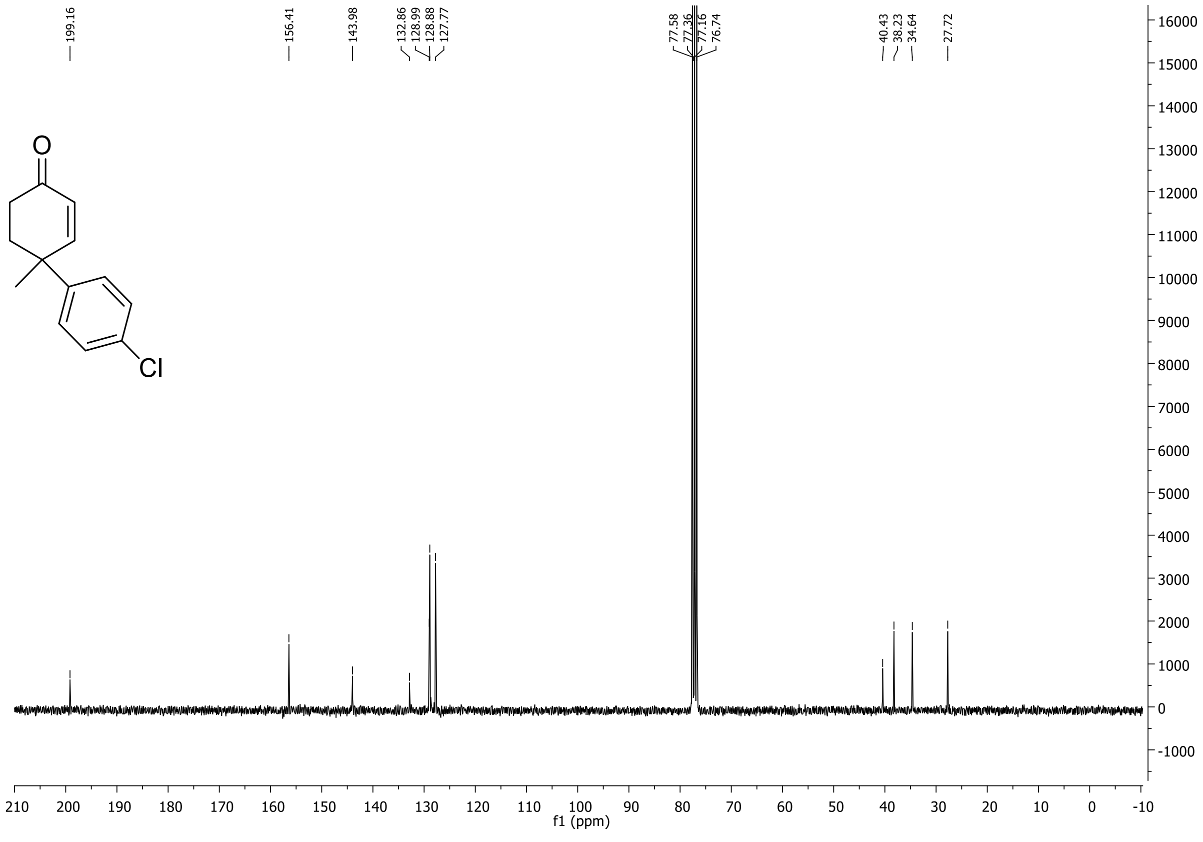

Figure S58. ^{13}C-NMR (75.53 MHz, $CDCl_3$) – **4’-Chloro-1-methyl-2,3-dihydro-[1,1’-biphenyl]-4(1*H*)-one (rac-2c)**.

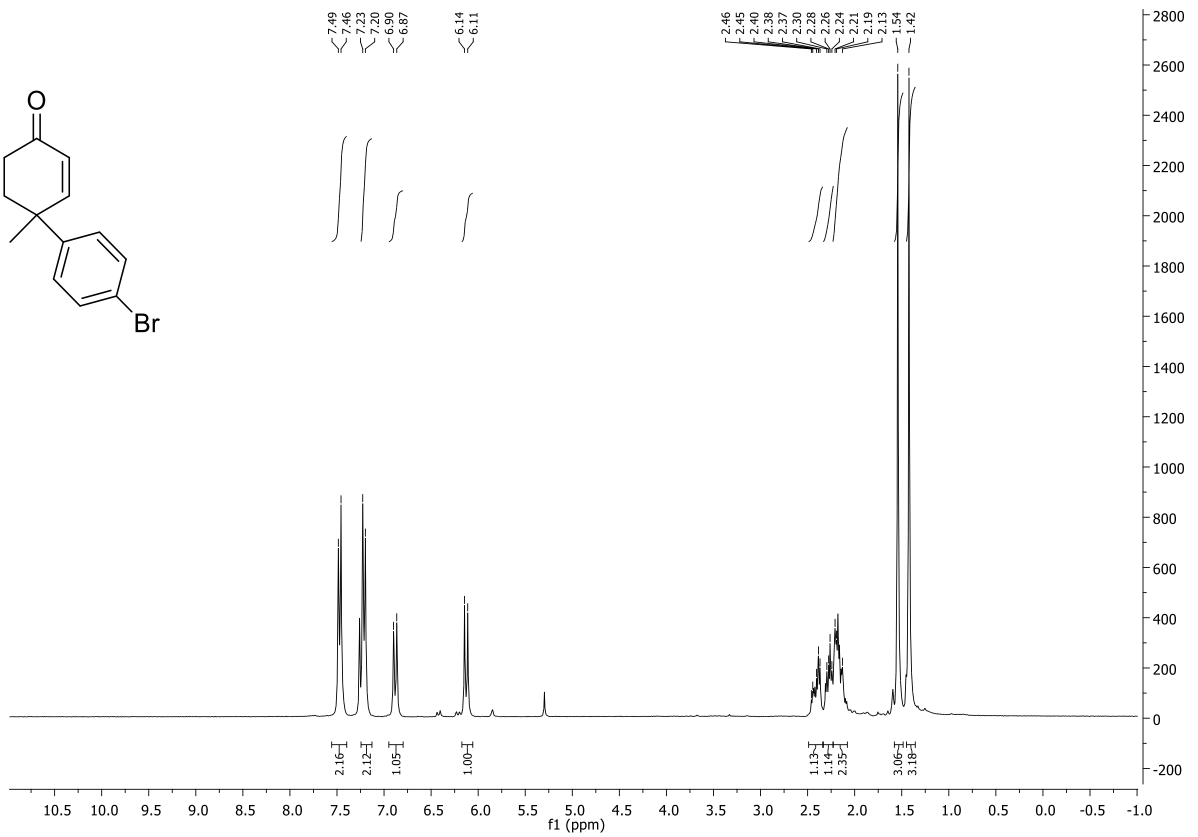

Figure S59. ^{1}H-NMR (300.36 MHz, $CDCl_3$) – **4'-Bromo-1-methyl-1,3-dihydro-[1,1'-biphenyl]-4(1*H*)-one (rac-2d)**.

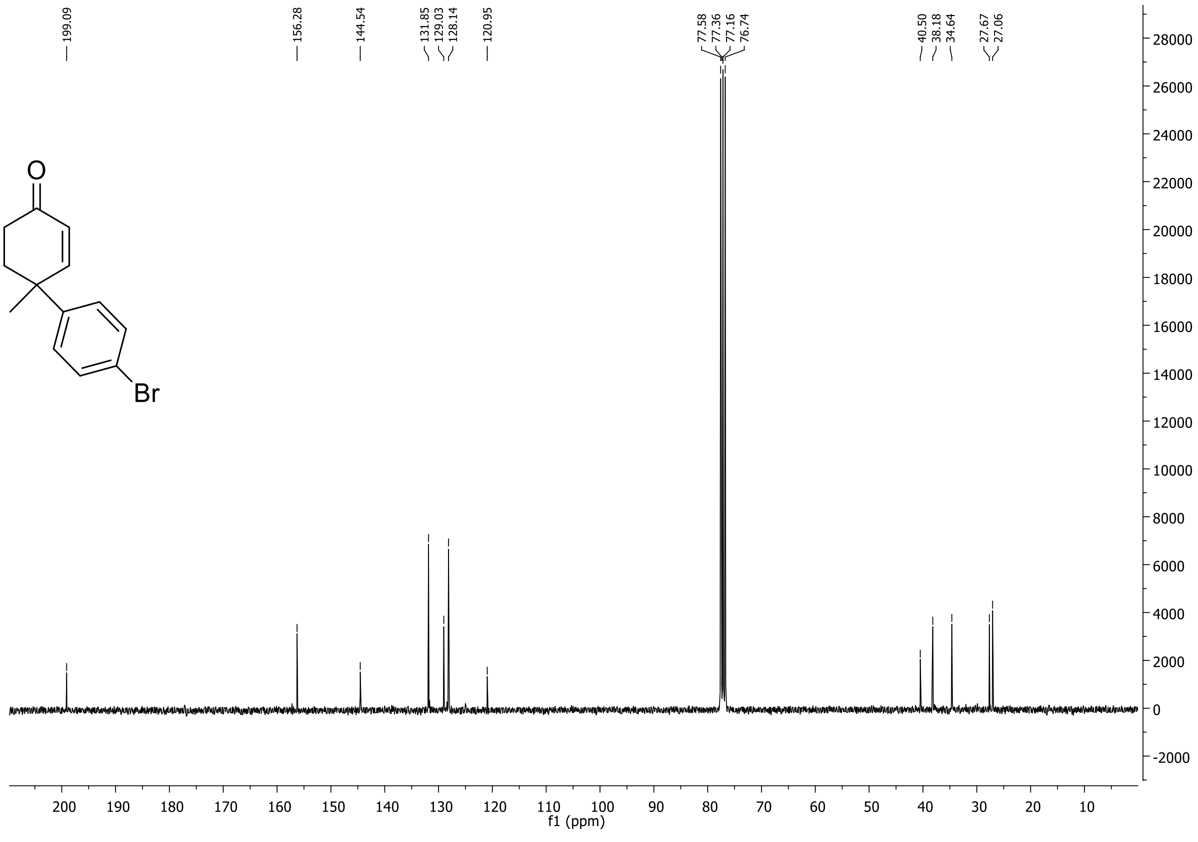

Figure S60. ^{13}C-NMR (75.53 MHz, $CDCl_3$) – **4'-Bromo-1-methyl-1,3-dihydro-[1,1'-biphenyl]-4(1*H*)-one (rac-2d)**

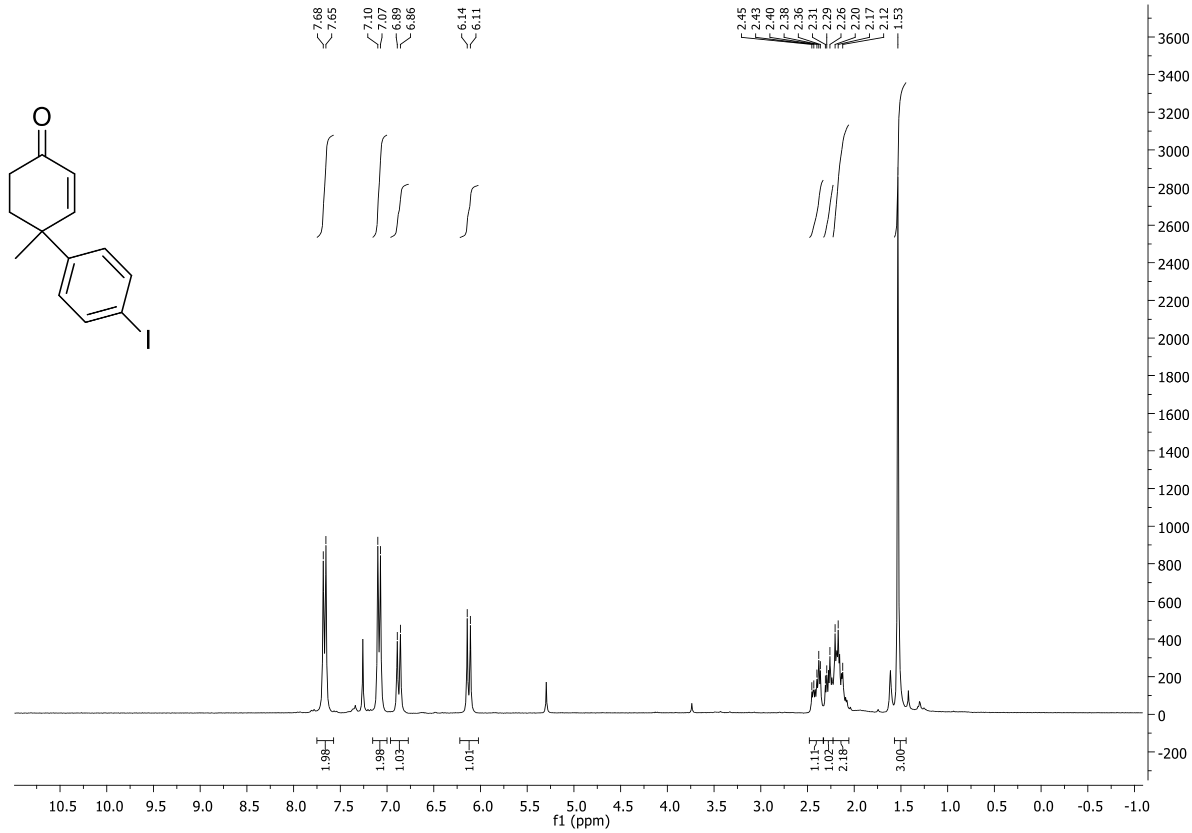

Figure S61. ^{1}H-NMR (300.36 MHz, $CDCl_3$) – **4'-Iodo-1-methyl-2,3-dihydro-[1,1'-biphenyl]-4(1*H*)-one (rac-2e)**.

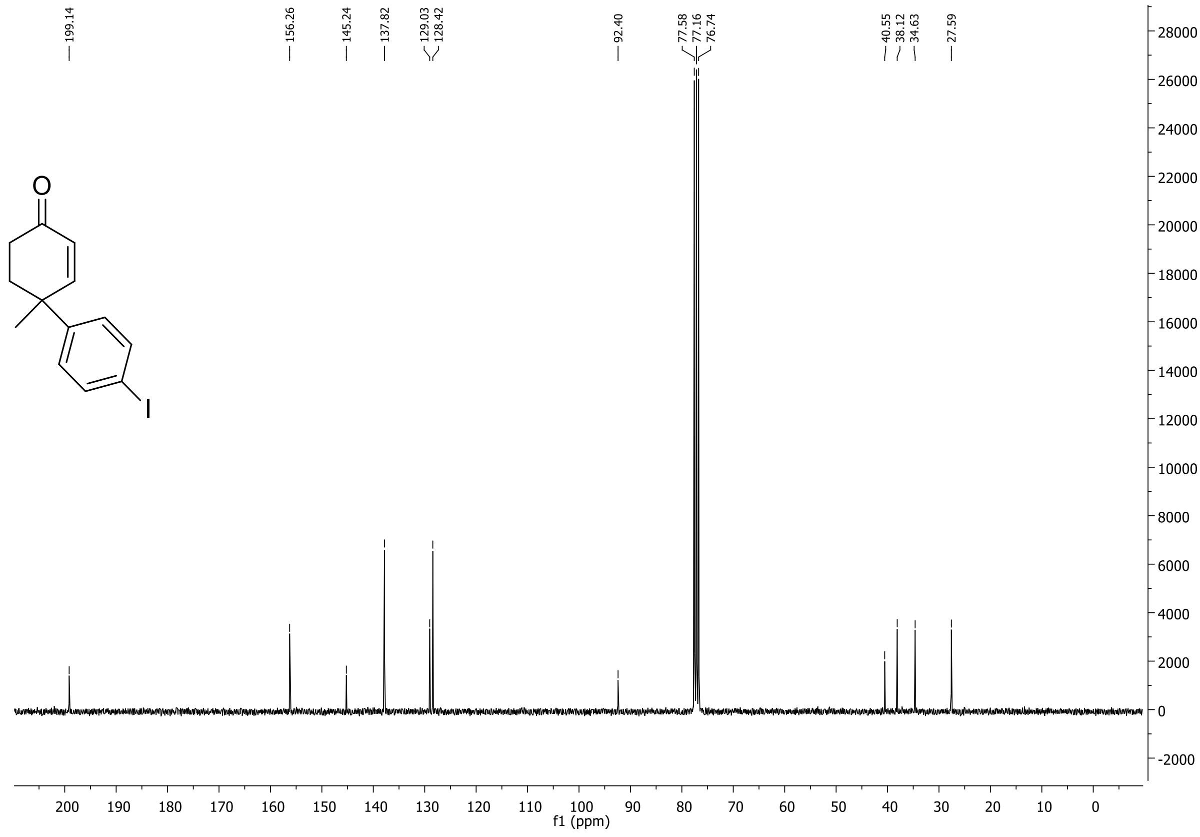

Figure S62. ^{13}C-NMR (75.53 MHz, $CDCl_3$) – **4'-Iodo-1-methyl-2,3-dihydro-[1,1'-biphenyl]-4(1*H*)-one (rac-2e)**.

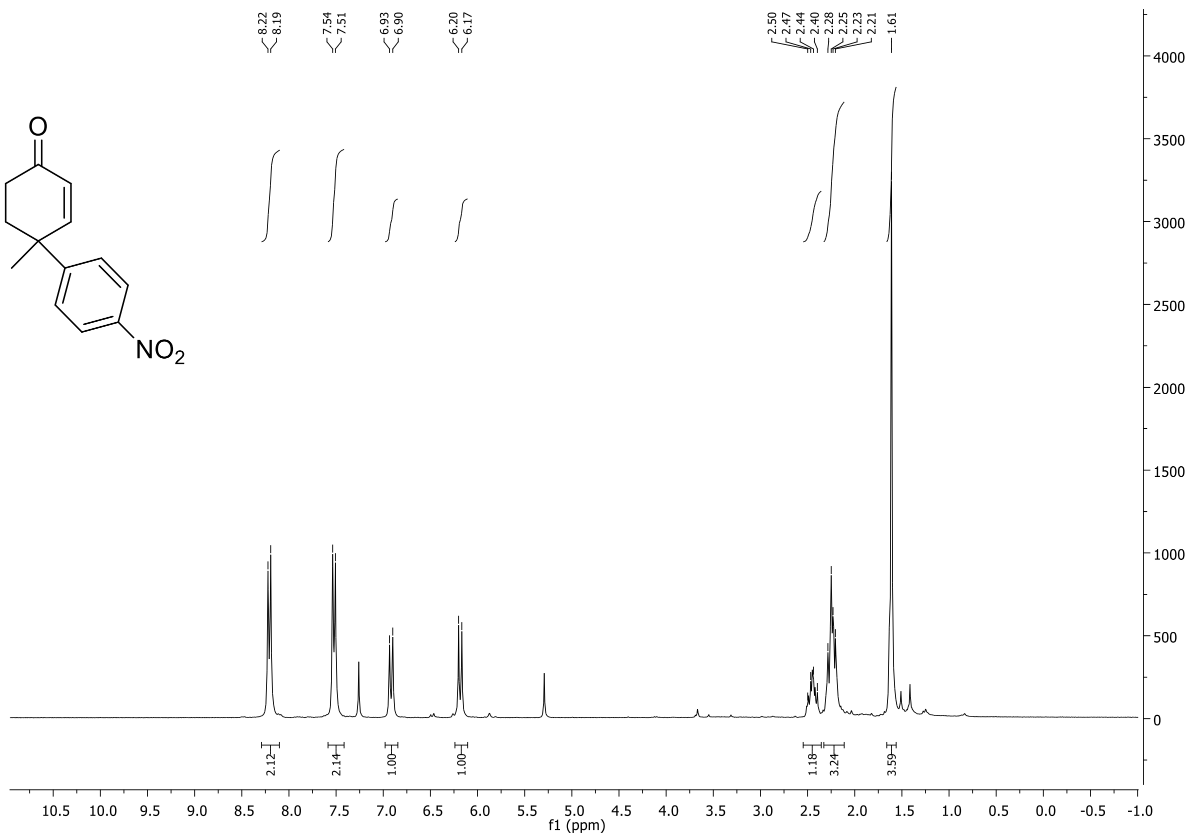

Figure S63. ^{1}H-NMR (300.36 MHz, $CDCl_3$) – **1-Methyl-4'-nitro-2,3-dihydro-[1,1'-biphenyl]-4(1*H*)-one (rac-2f)**.

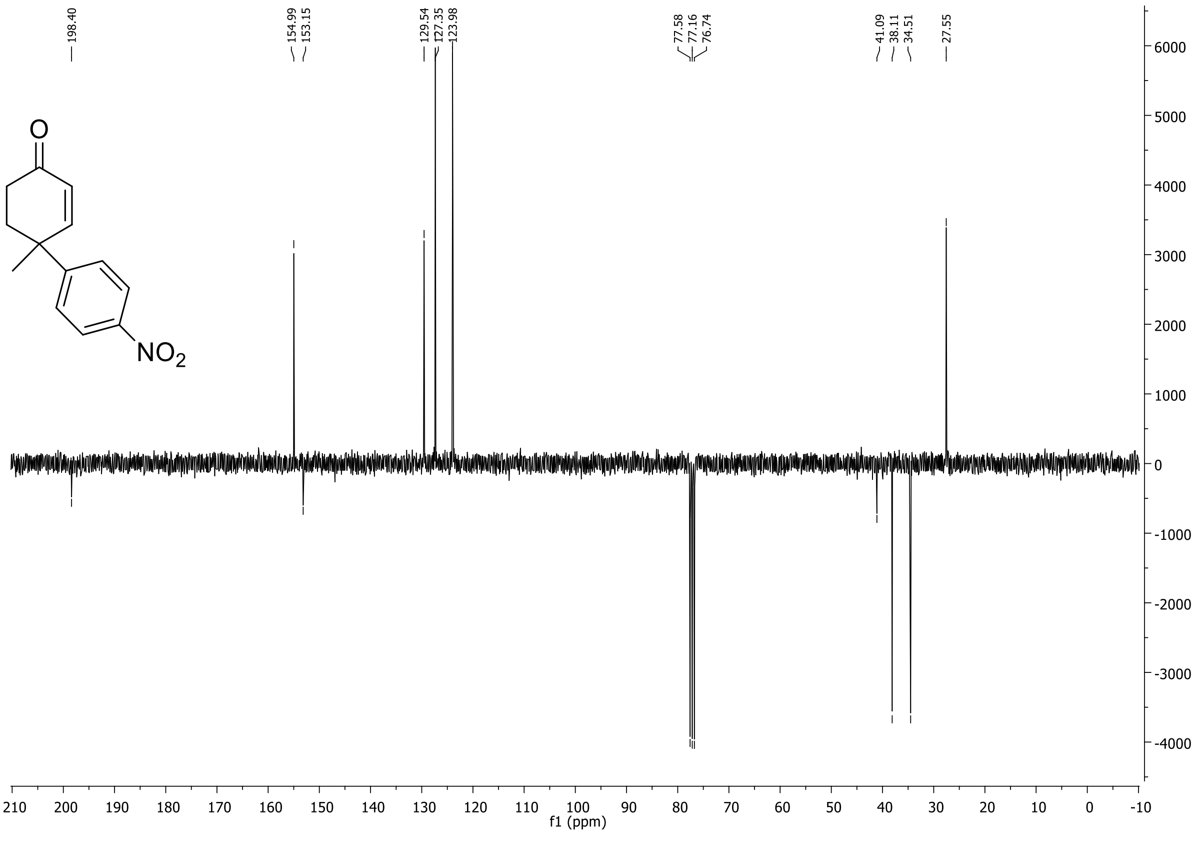

Figure S64. ^{13}C-NMR (75.53 MHz, $CDCl_3$) – **1-Methyl-4'-nitro-2,3-dihydro-[1,1'-biphenyl]-4(1*H*)-one (rac-2f)**.

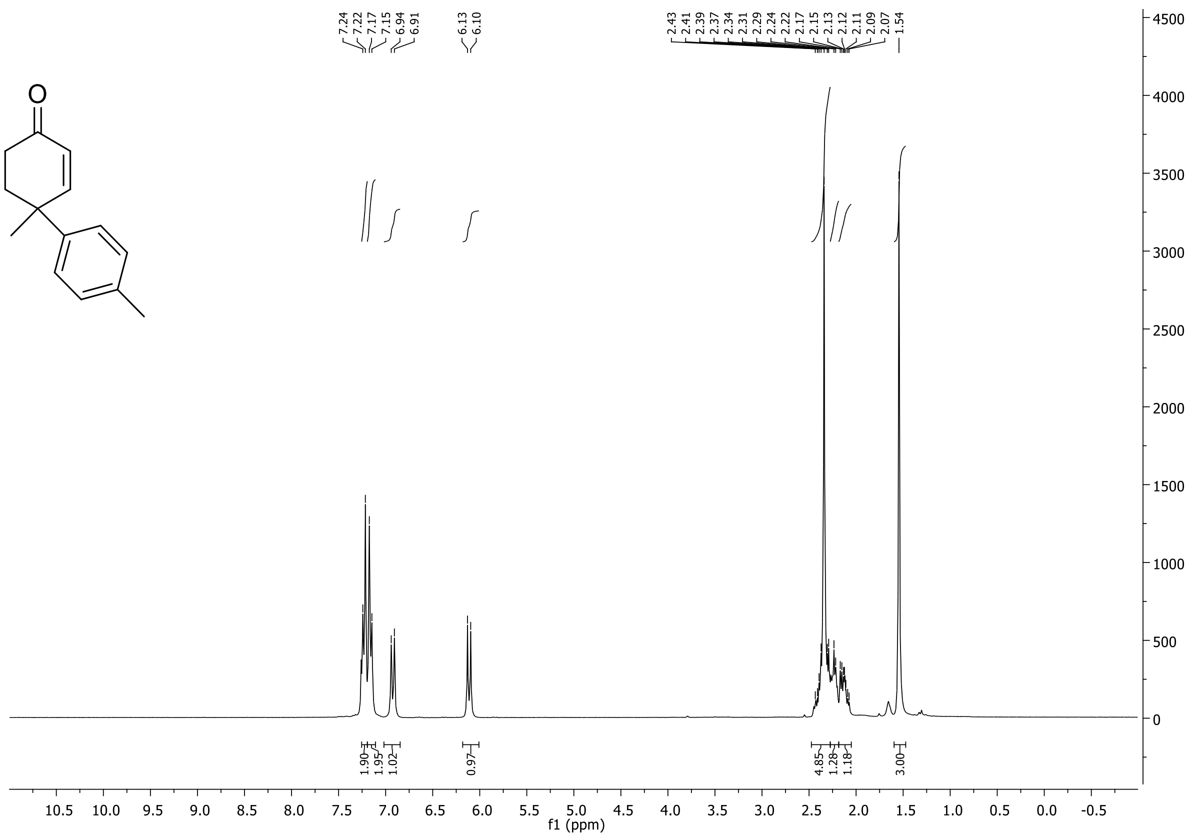

Figure S65. ^{1}H-NMR (300.36 MHz, $CDCl_3$) – **1,4'-Dimethyl-2,3-dihydro-[1,1'-biphenyl]-4(1*H*)-one (rac-2g)**.

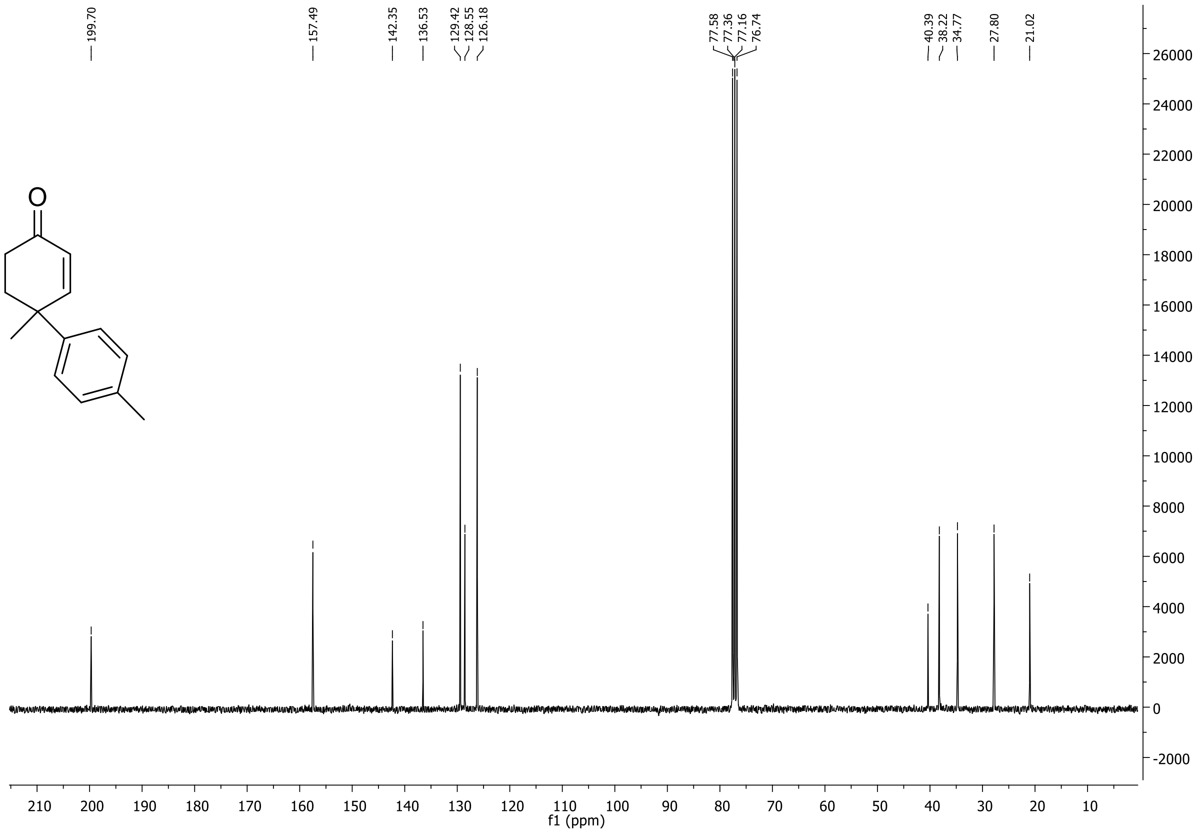

Figure S66. ^{13}C-NMR (75.53 MHz, $CDCl_3$) – **1,4'-Dimethyl-2,3-dihydro-[1,1'-biphenyl]-4(1*H*)-one (rac-2g)**.

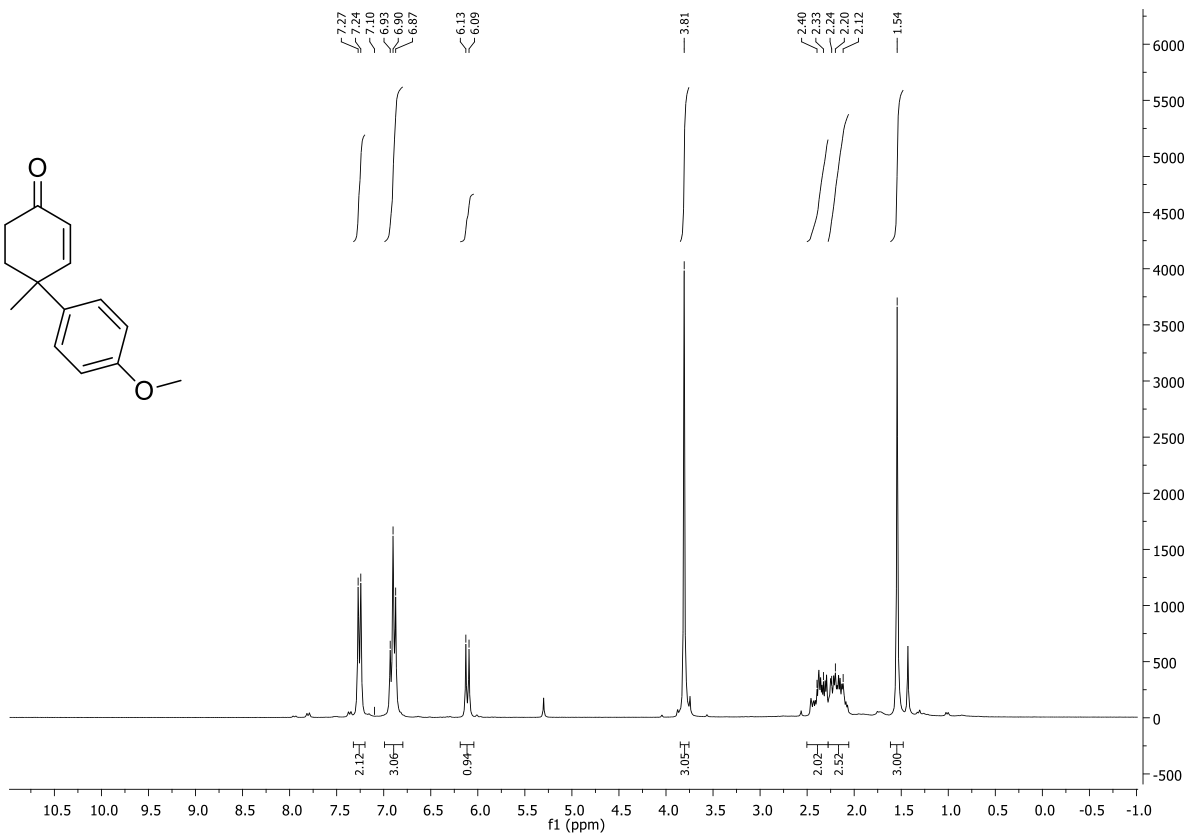

Figure S67. ^{1}H-NMR (300.36 MHz, $CDCl_3$) – **4'-Methoxy-1-methyl-2,3-dihydro-[1,1'-biphenyl]-4(1*H*)-one (rac-2h)**.

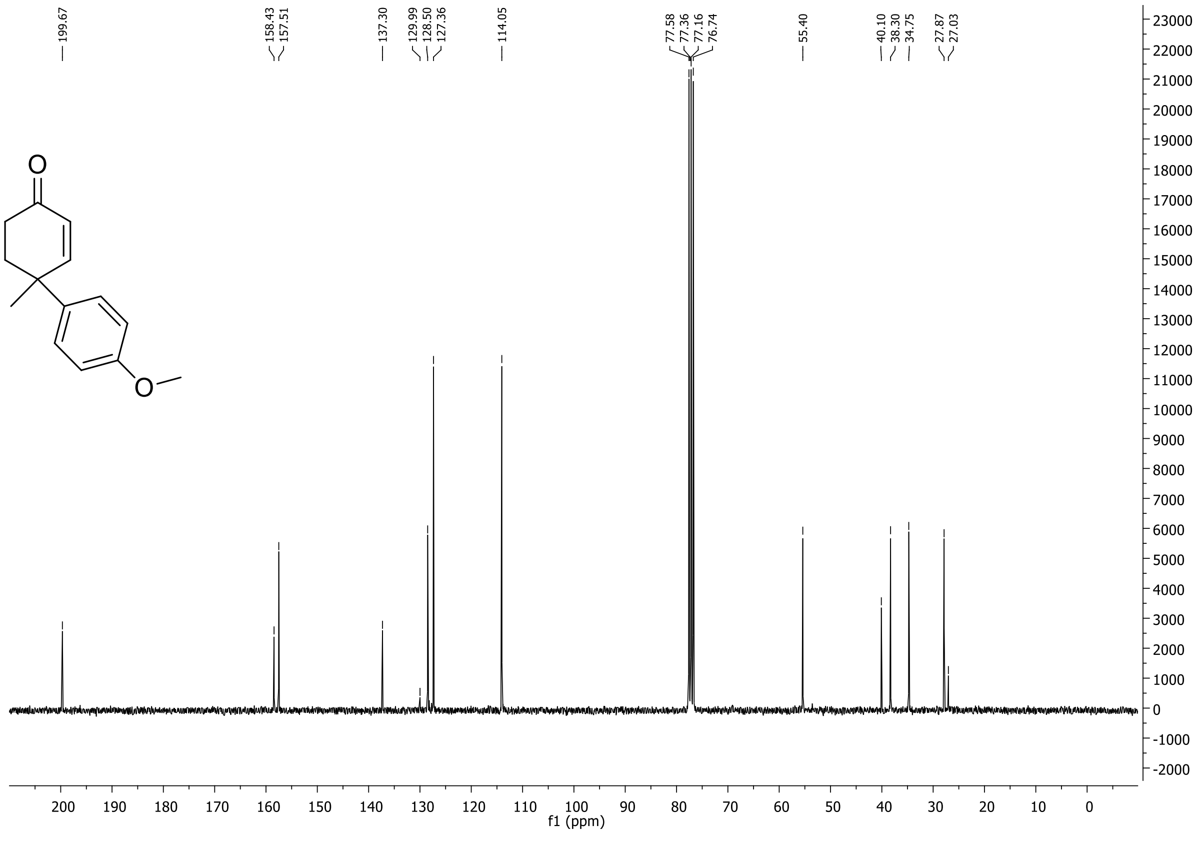

Figure S68. ^{13}C-NMR (75.53 MHz, $CDCl_3$) – **4'-Methoxy-1-methyl-2,3-dihydro-[1,1'-biphenyl]-4(1*H*)-one (rac-2h)**.

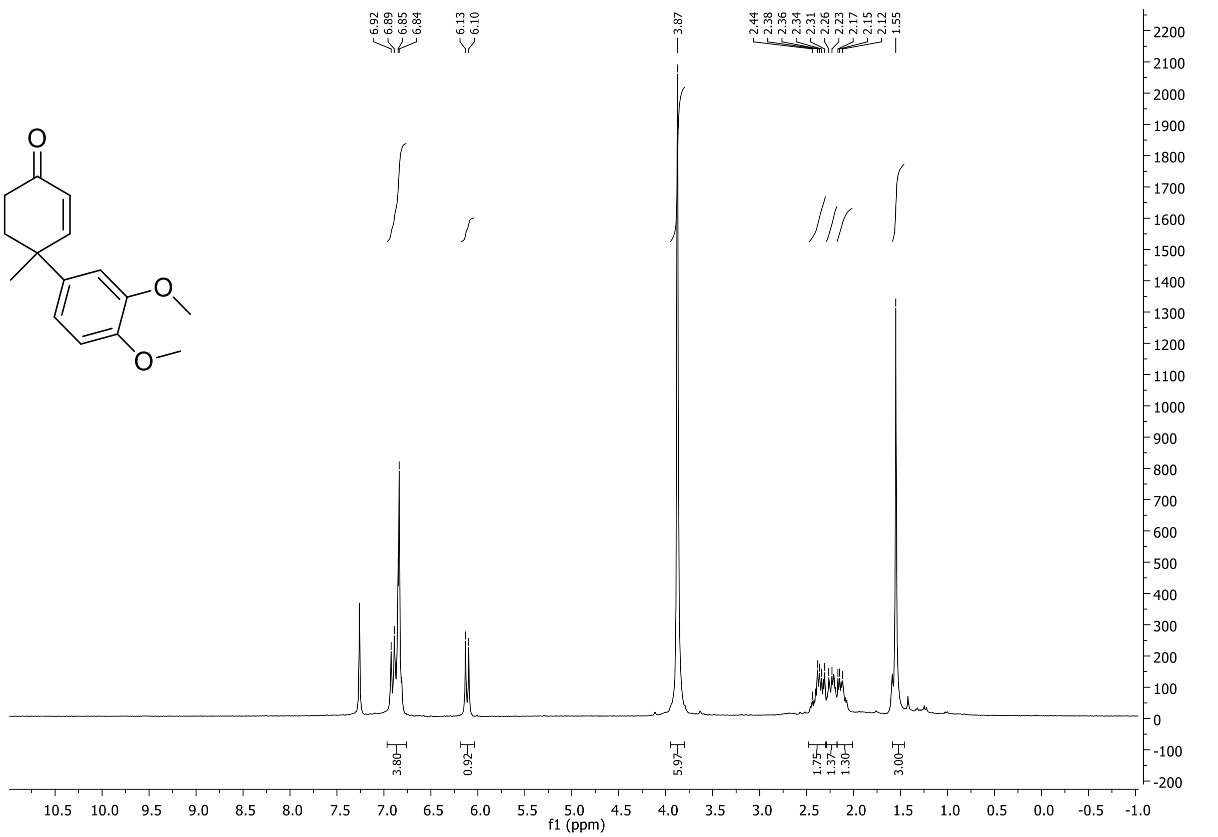

Figure S69. ^{1}H-NMR (300.36 MHz, $CDCl_3$) – **(3',4'-Dimethoxy-1-methyl-2,3-dihydro-[1,1'-biphenyl]-4(1*H*)-one (rac-2i)**.

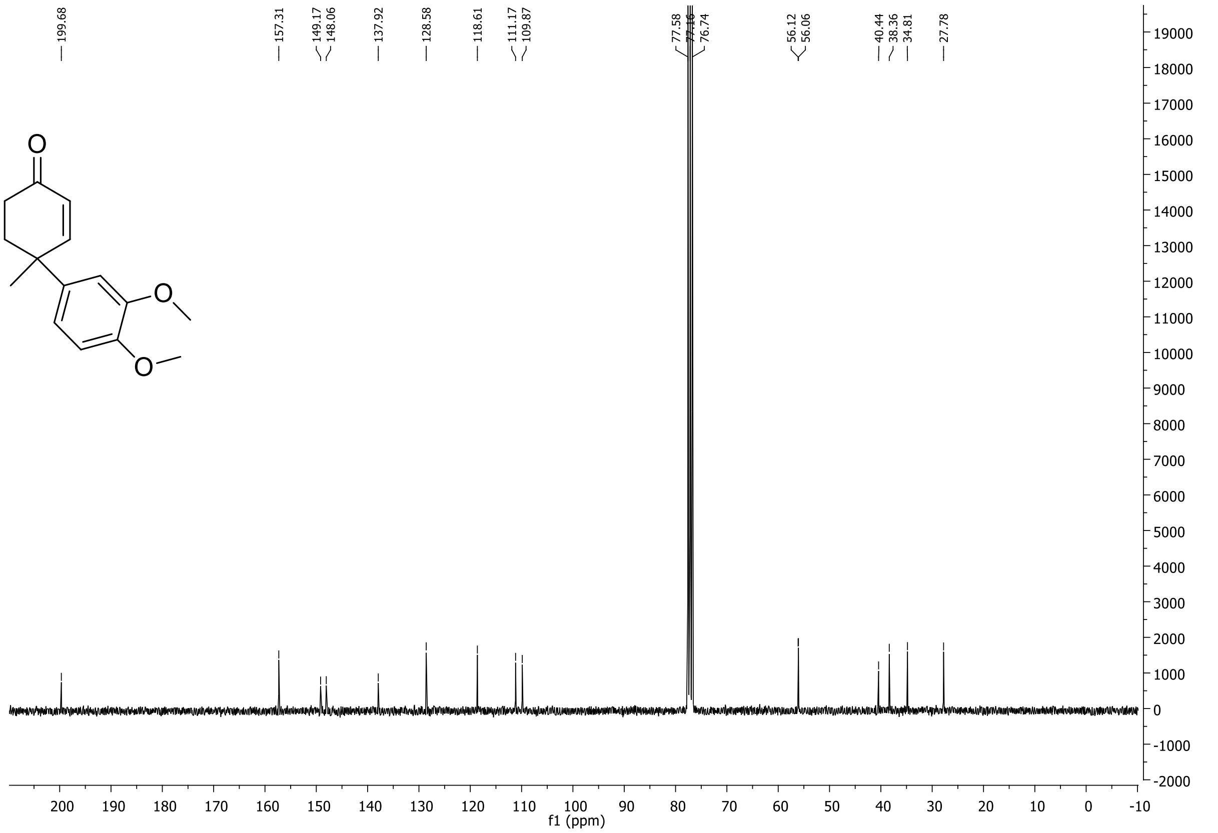

Figure S70. ^{13}C-NMR (75.53 MHz, $CDCl_3$) – **(3',4'-Dimethoxy-1-methyl-2,3-dihydro-[1,1'-biphenyl]-4(1*H*)-one (rac-2i)**.

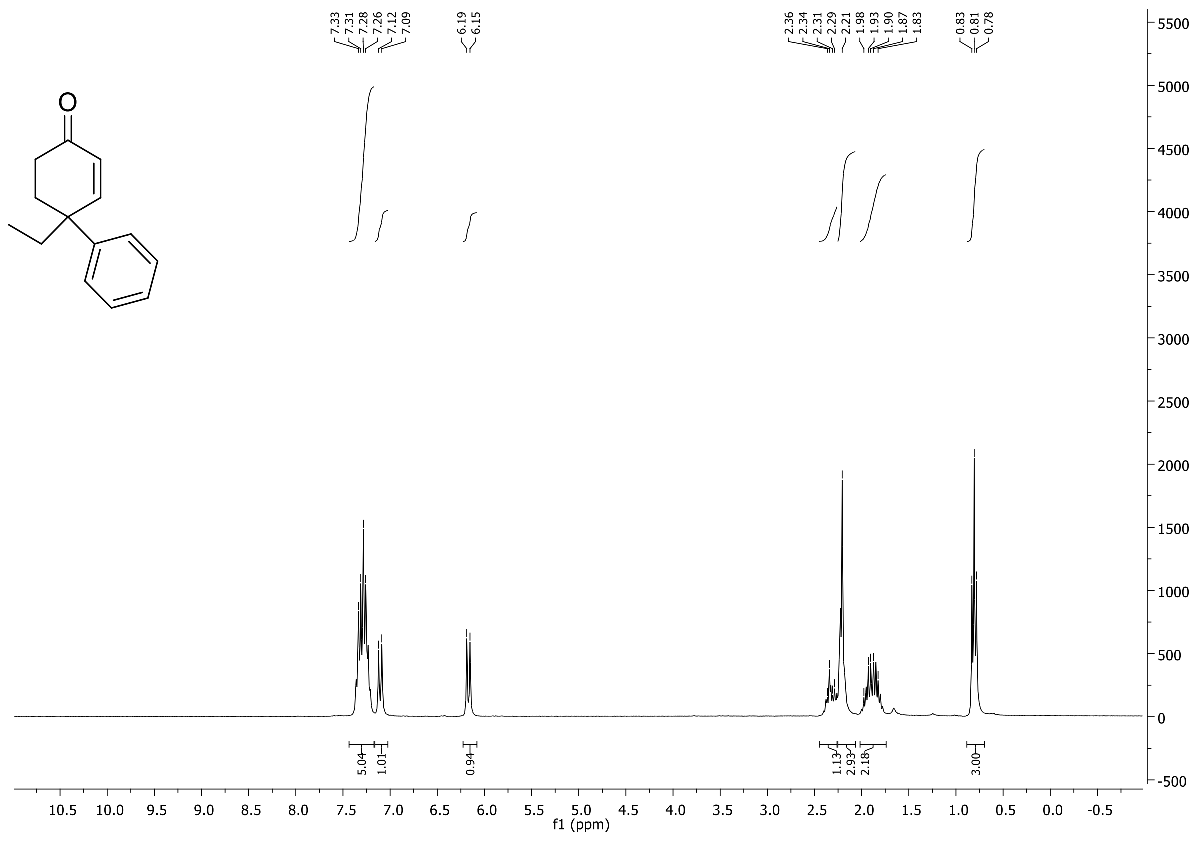

Figure S71. ^{1}H-NMR (300.36 MHz, $CDCl_3$) – **1-Ethyl-2,3-dihydro-[1,1’-biphenyl]-4(1*H*)-one (rac-2j)**.

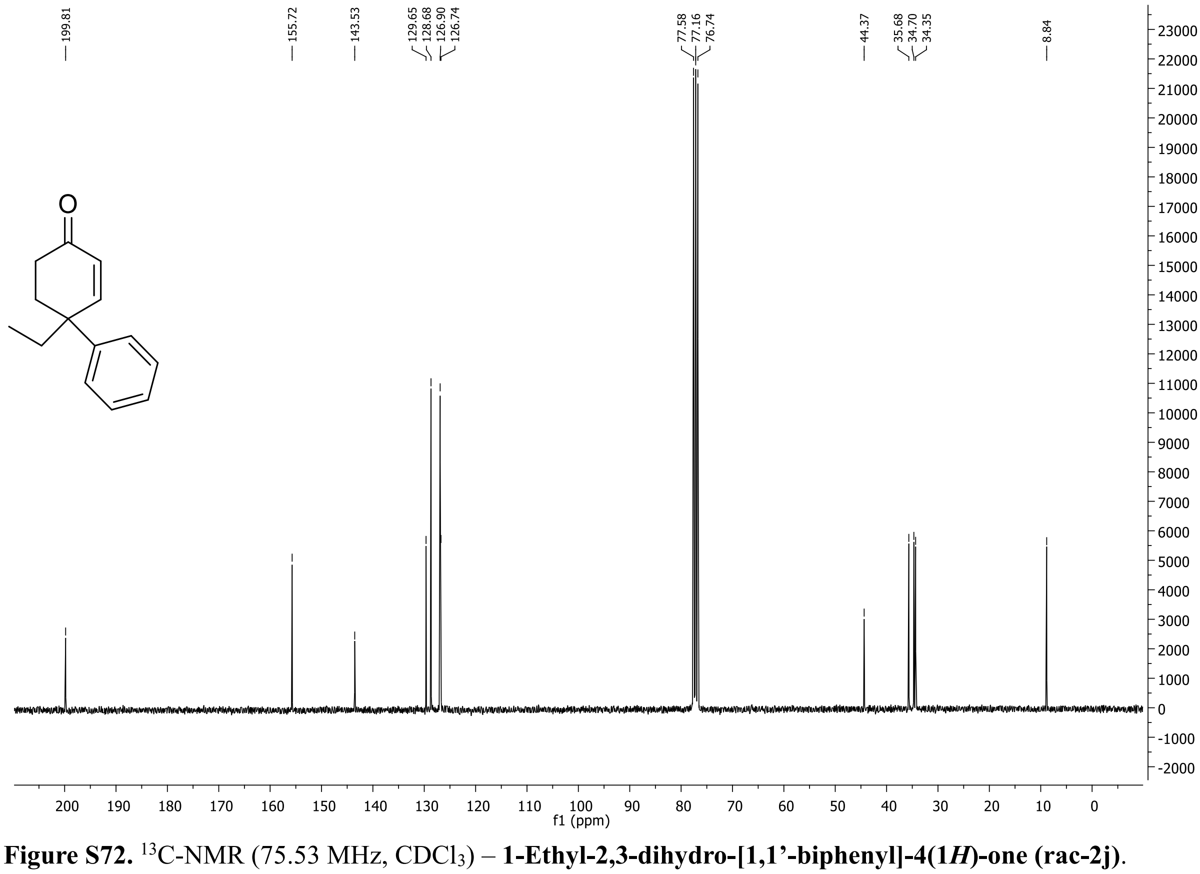

Figure S72. ^{13}C-NMR (75.53 MHz, $CDCl_3$) – **1-Ethyl-2,3-dihydro-[1,1’-biphenyl]-4(1*H*)-one (rac-2j)**.

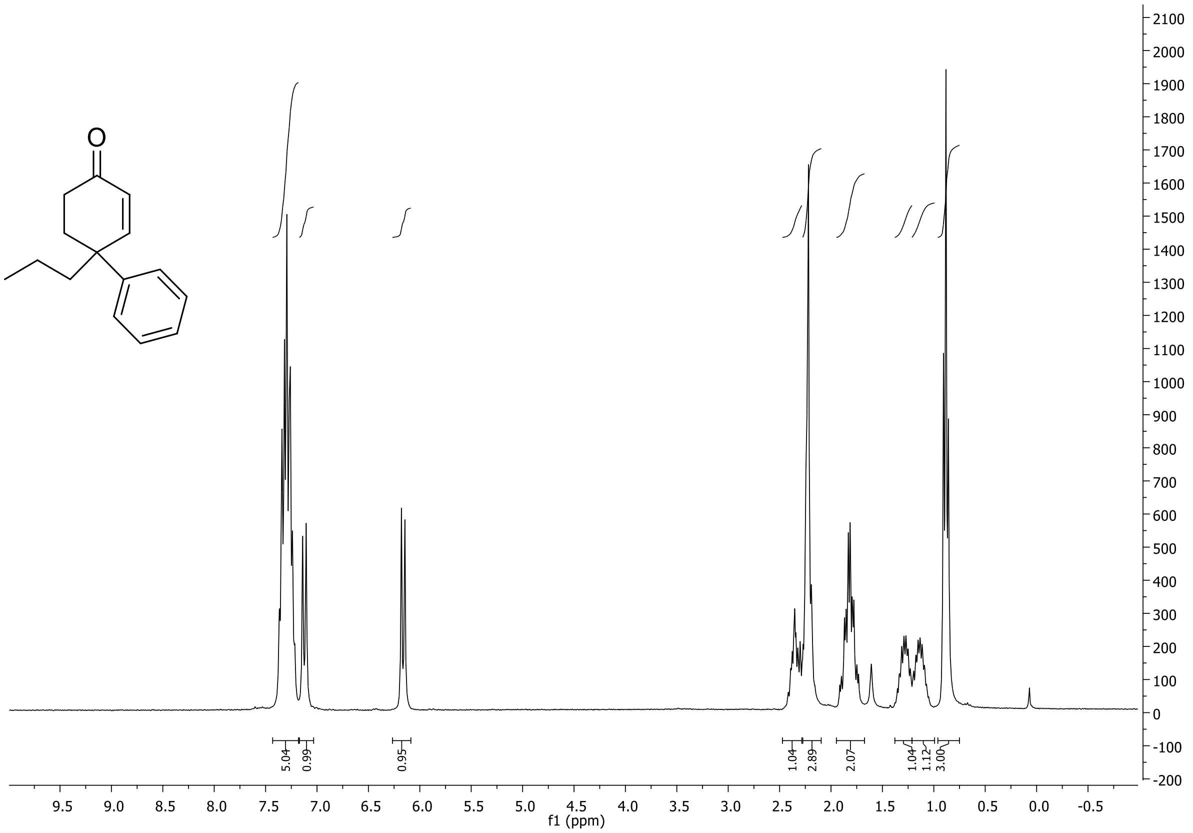

Figure S73. ^{1}H-NMR (300.36 MHz, $CDCl_3$) – **1-Propyl-2,3-dihydro-[1,1'-biphenyl]-4(1*H*)-one (rac-2k)**.

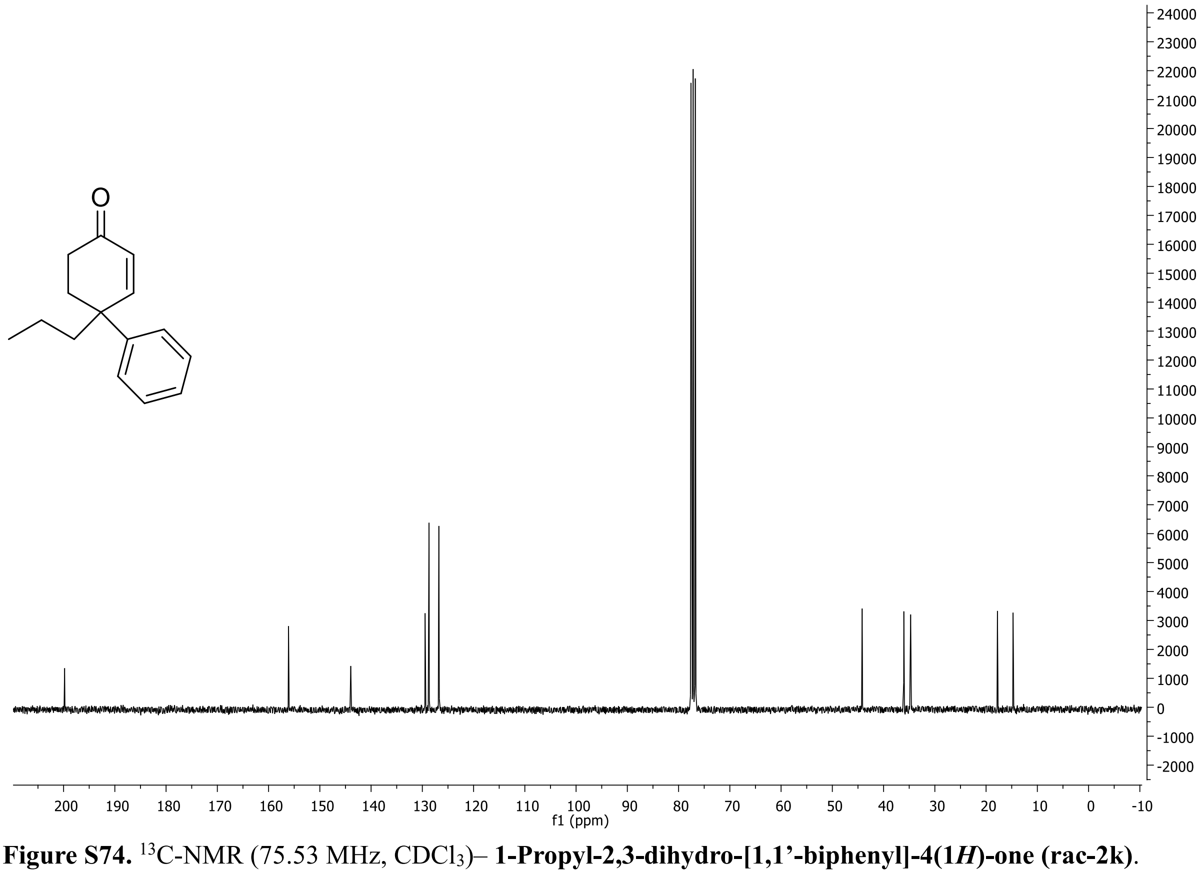

Figure S74. ^{13}C-NMR (75.53 MHz, $CDCl_3$)– **1-Propyl-2,3-dihydro-[1,1'-biphenyl]-4(1*H*)-one (rac-2k)**.

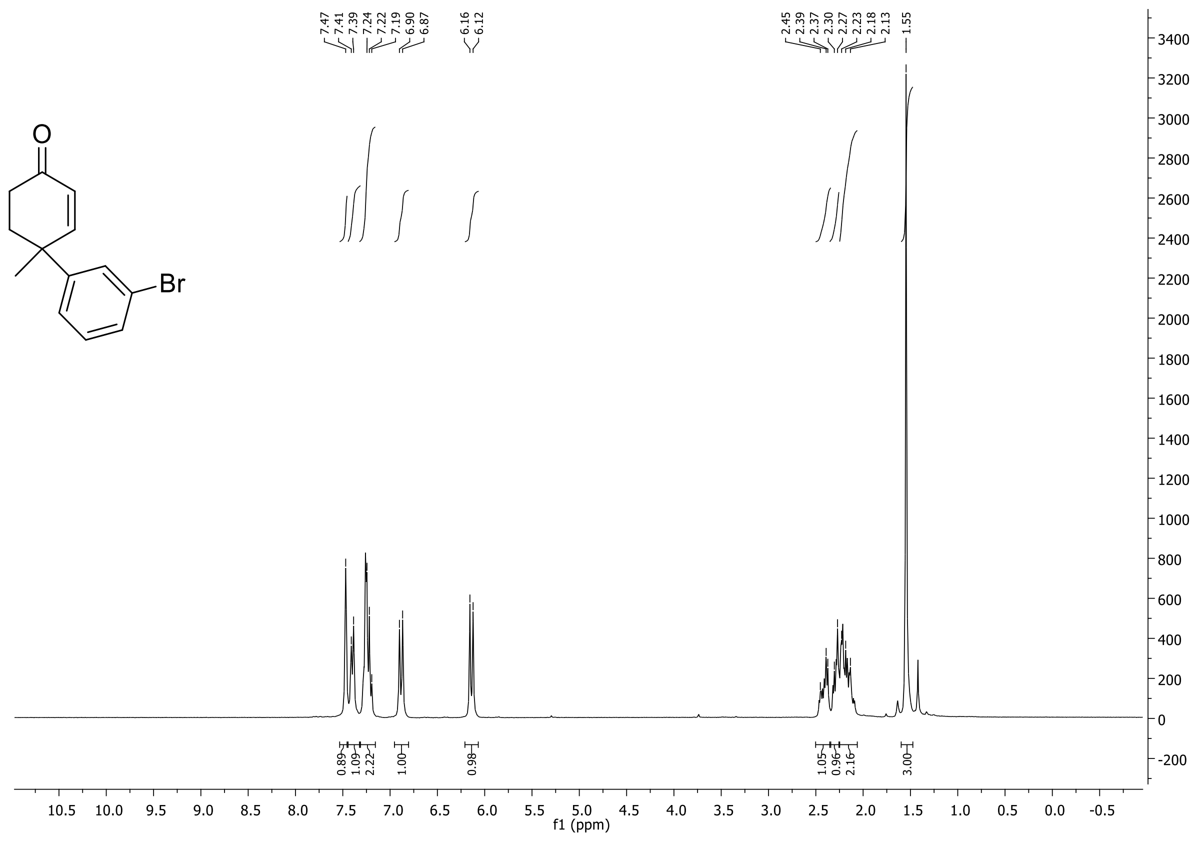

Figure S75. ^{1}H-NMR (300.36 MHz, $CDCl_3$) – **3'-Bromo-1-methyl-2,3-dihydro-[1,1'-biphenyl]-4(1*H*)-one (rac-2l)**.

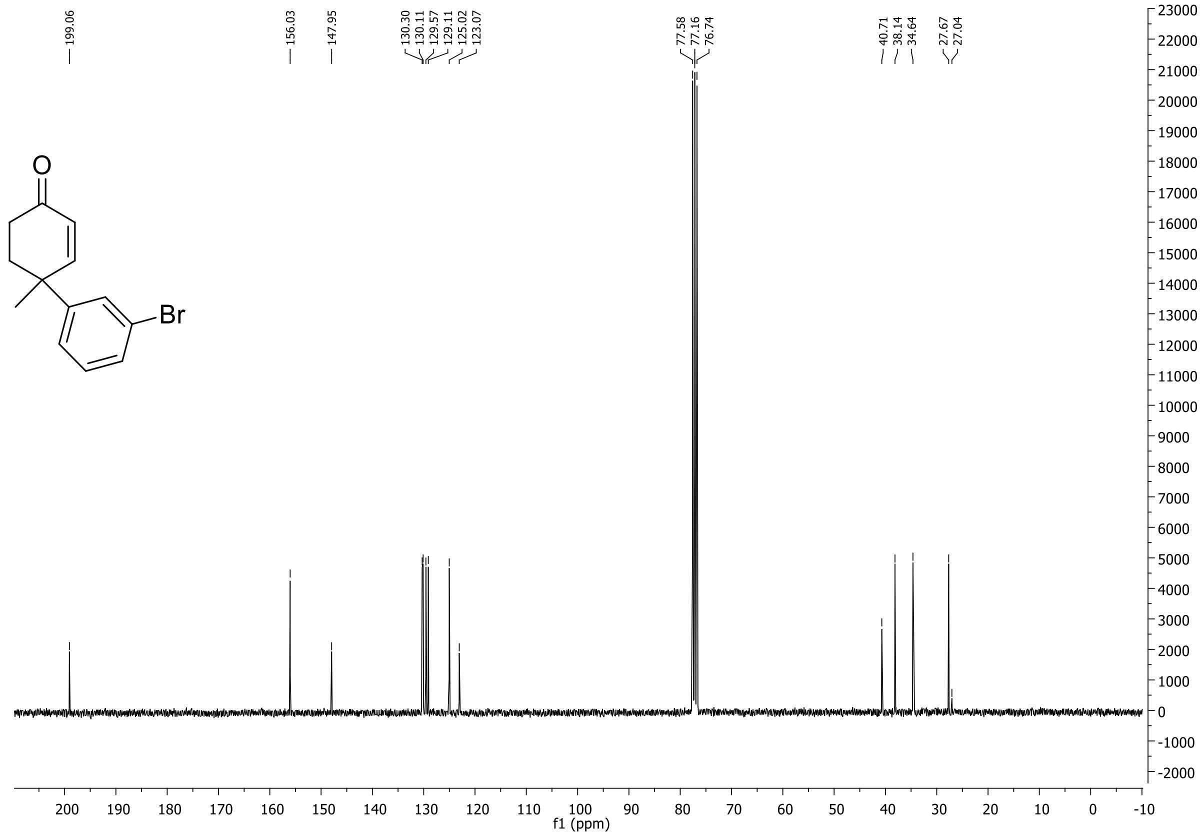

Figure S76. ^{13}C-NMR (75.53 MHz, $CDCl_3$) – **3'-Bromo-1-methyl-2,3-dihydro-[1,1'-biphenyl]-4(1*H*)-one (rac-2l)**.

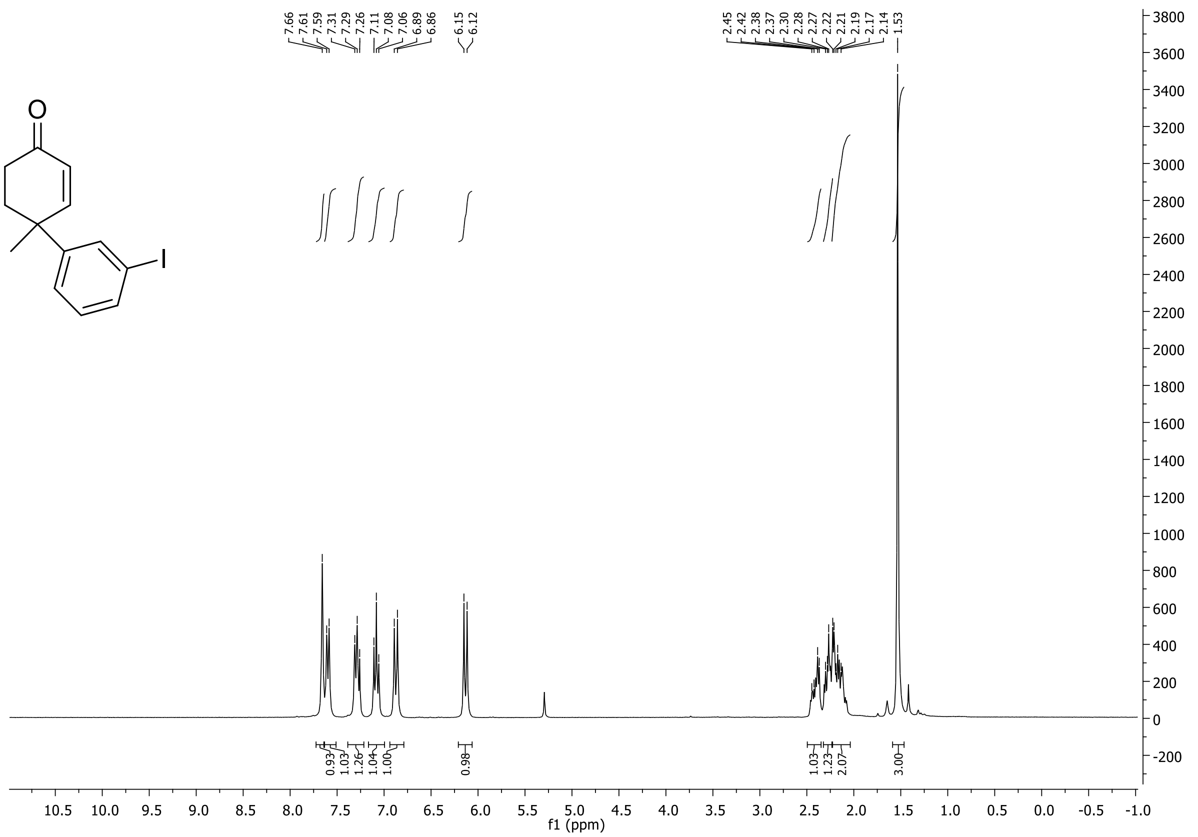

Figure S77. ^{1}H-NMR (300.36 MHz, $CDCl_3$) – **3'-Iodo-1-methyl-2,3-dihydro-[1,1'-biphenyl]-4(1*H*)-one (rac-2m)**.

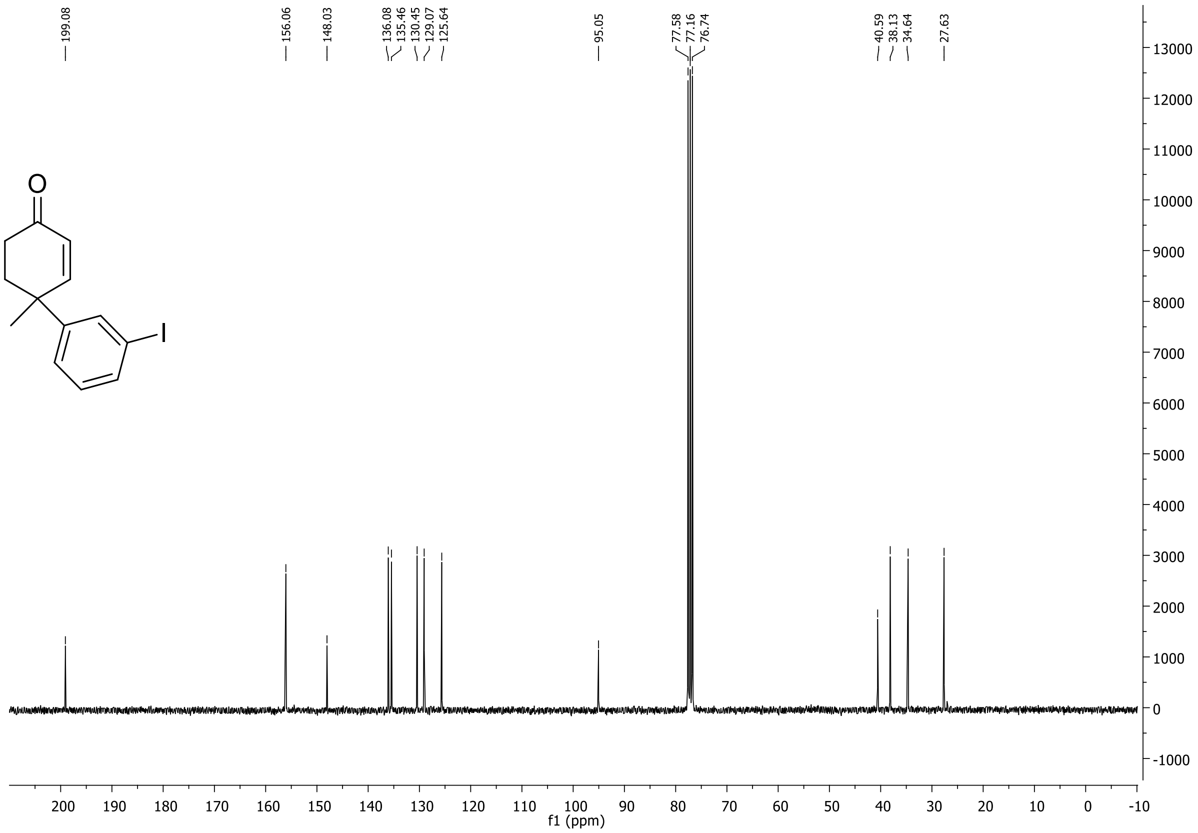

Figure S78. ^{13}C-NMR (75.53 MHz, $CDCl_3$) – **3'-Iodo-1-methyl-2,3-dihydro-[1,1'-biphenyl]-4(1*H*)-one (rac-2m)**.

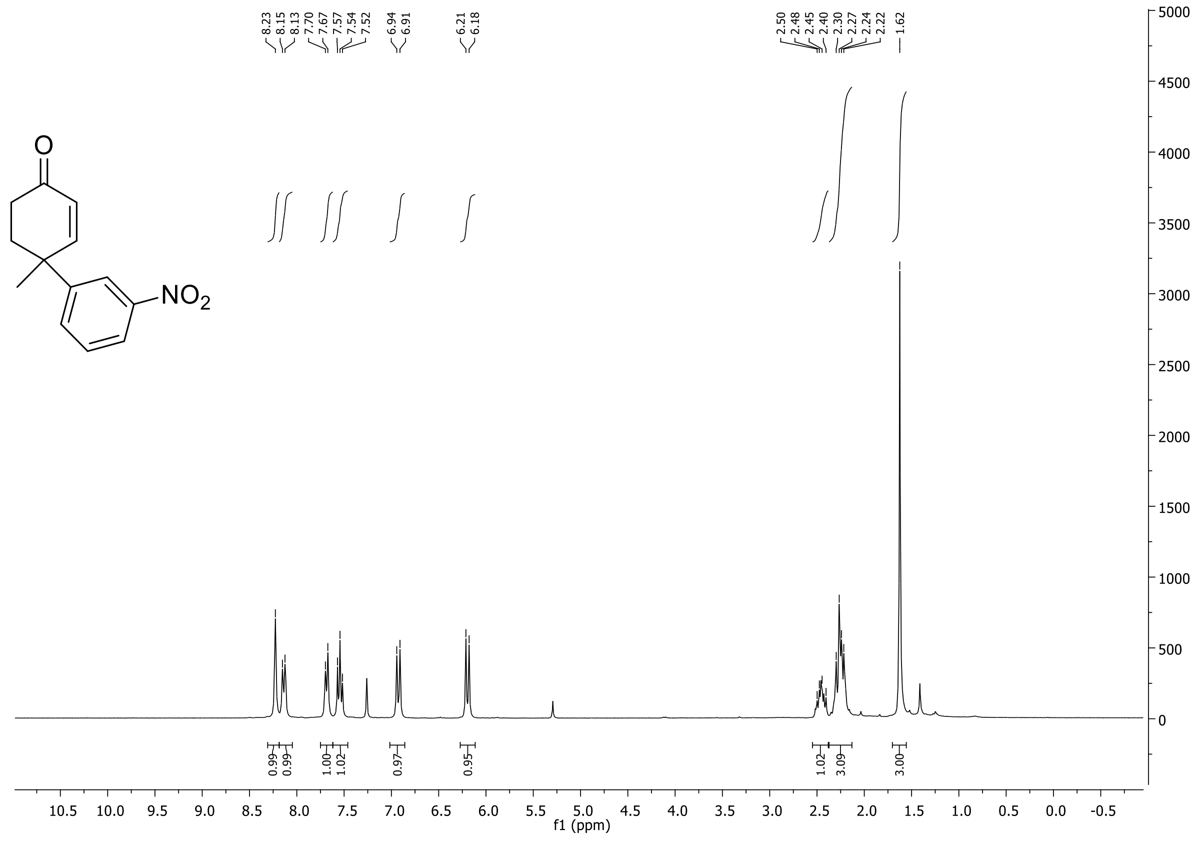

Figure S79. ^{1}H-NMR (300.36 MHz, $CDCl_3$) – **1-Methyl-3'-nitro-2,3-dihydro-[1,1'-biphenyl]-4(1*H*)-one (rac-2n)**.

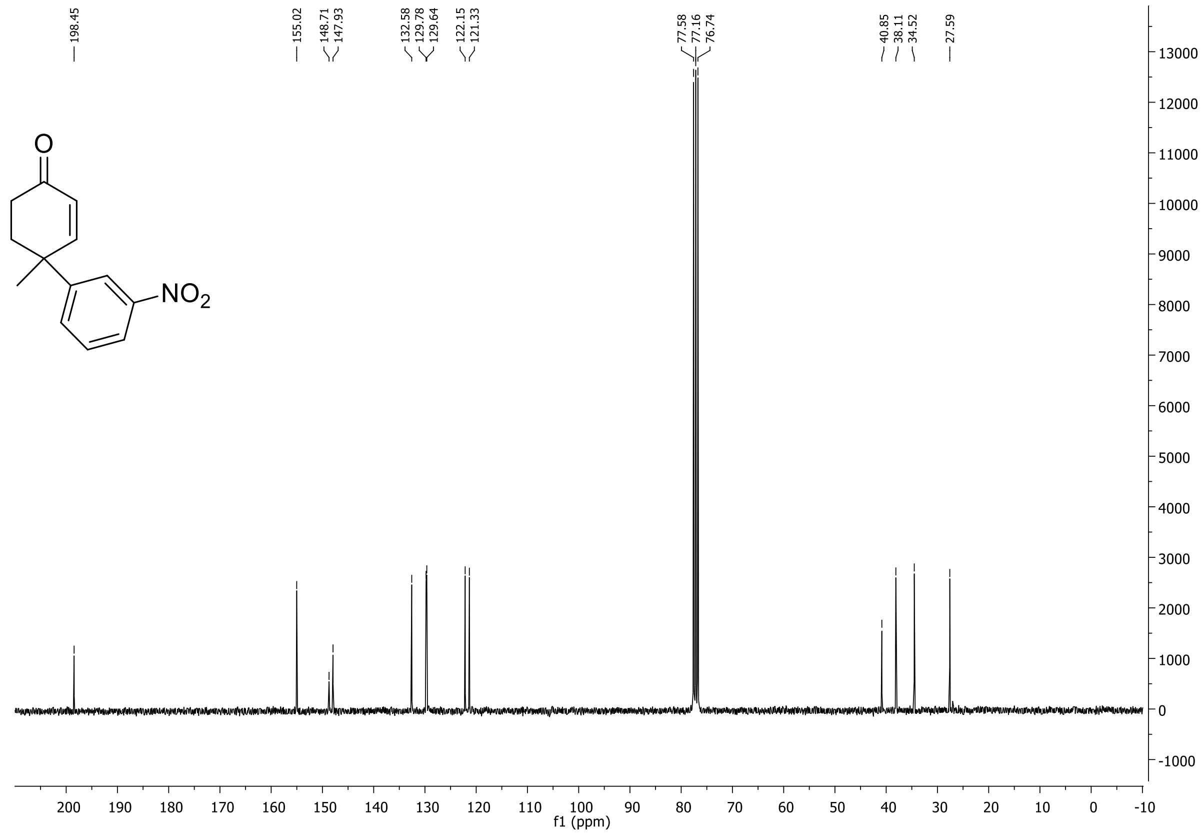

Figure S80. ^{13}C-NMR (75.53 MHz, $CDCl_3$) – **1-Methyl-3'-nitro-2,3-dihydro-[1,1'-biphenyl]-4(1*H*)-one (rac-2n)**.

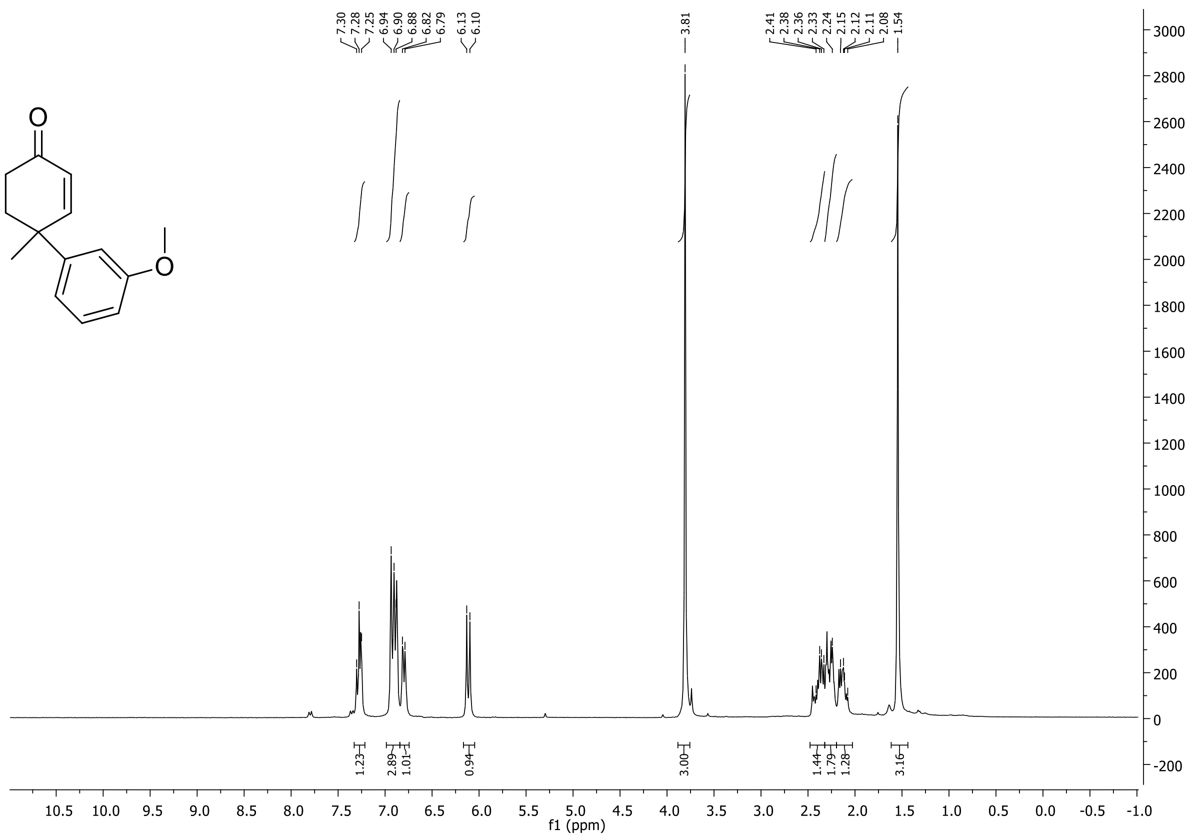

Figure S81. ^{1}H-NMR (300.36 MHz, $CDCl_3$) – **3’-Methoxy-1-methyl-2,3-dihydro-[1,1’-biphenyl]-4(1*H*)-one (rac-2o)**.

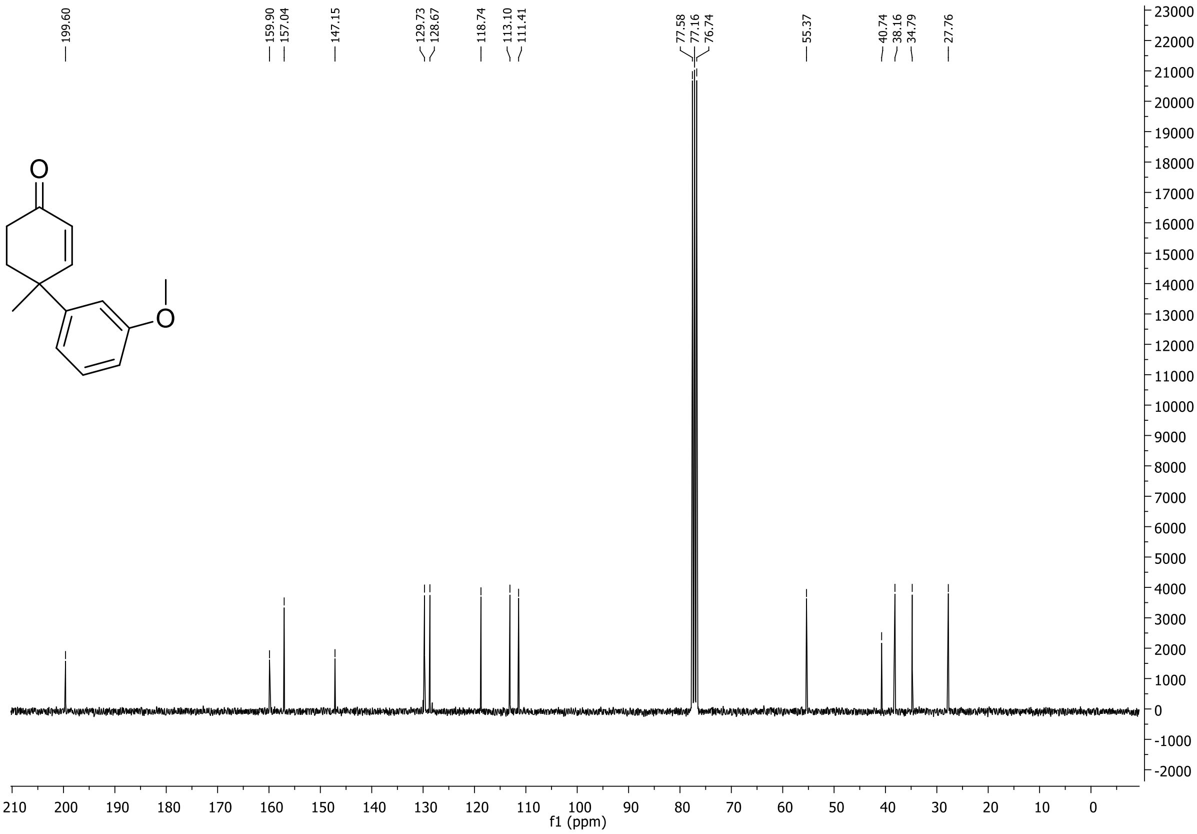

Figure S82. ^{13}C-NMR (75.53 MHz, $CDCl_3$) – **3’-Methoxy-1-methyl-2,3-dihydro-[1,1’-biphenyl]-4(1*H*)-one (rac-2o)**.

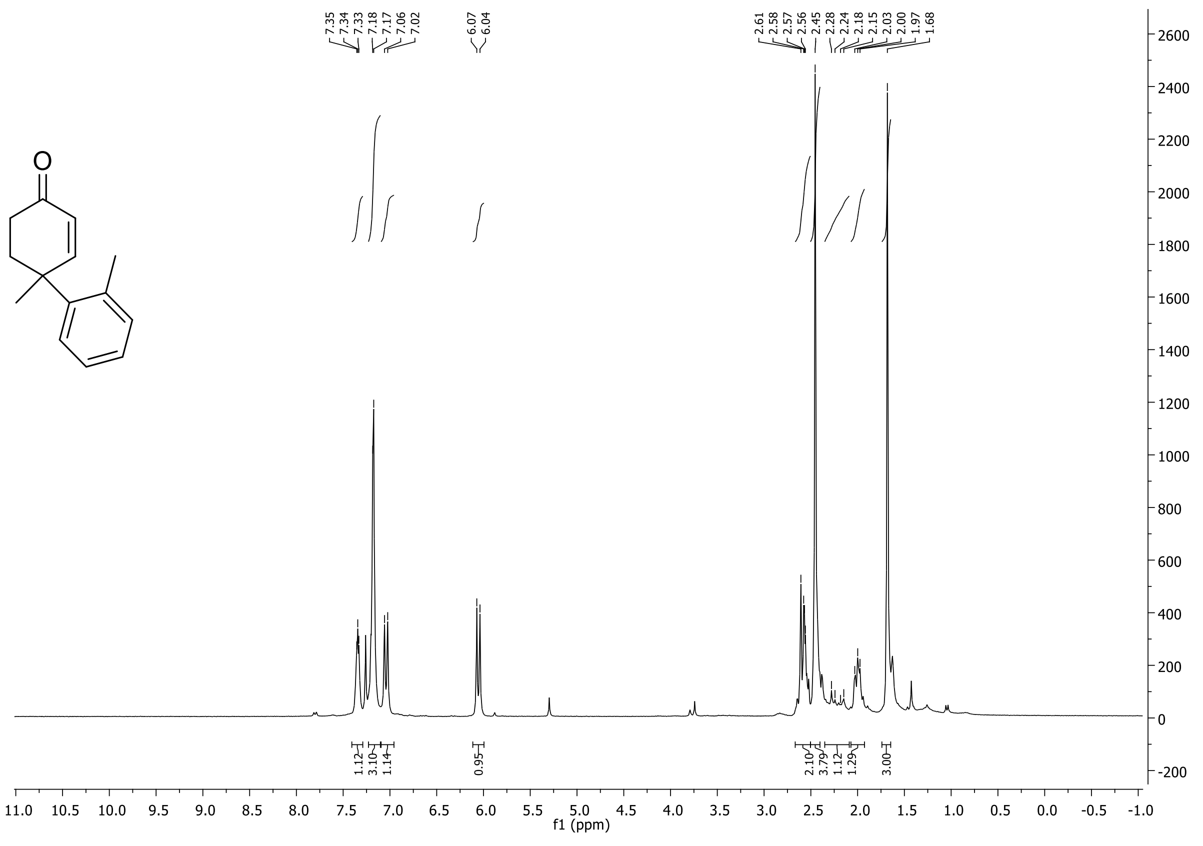

Figure S83. ^{1}H-NMR (300.36 MHz, $CDCl_3$) – **1,2'-Dimethyl-2,3-dihydro-[1,1'-biphenyl]-4(1*H*)-one (rac-2p)**.

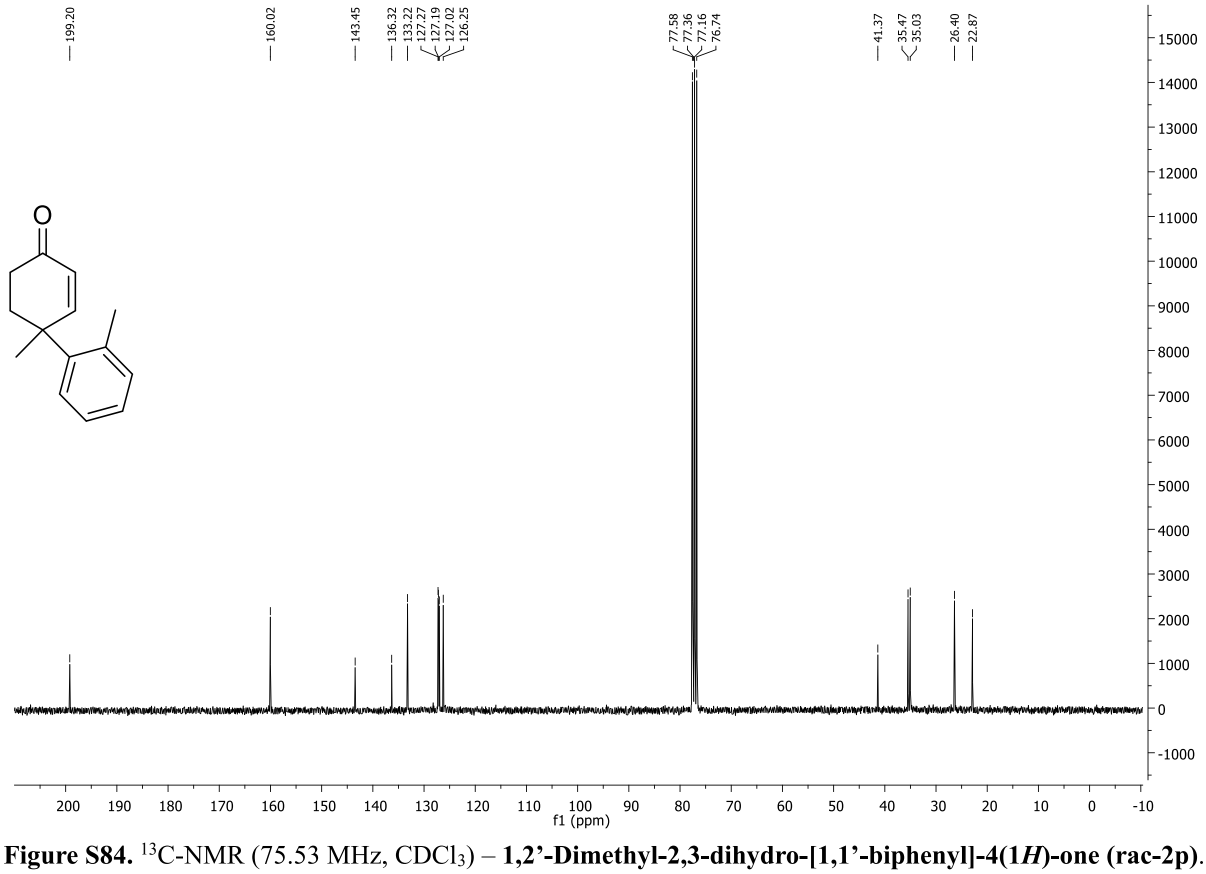

Figure S84. ^{13}C-NMR (75.53 MHz, $CDCl_3$) – **1,2'-Dimethyl-2,3-dihydro-[1,1'-biphenyl]-4(1*H*)-one (rac-2p)**.

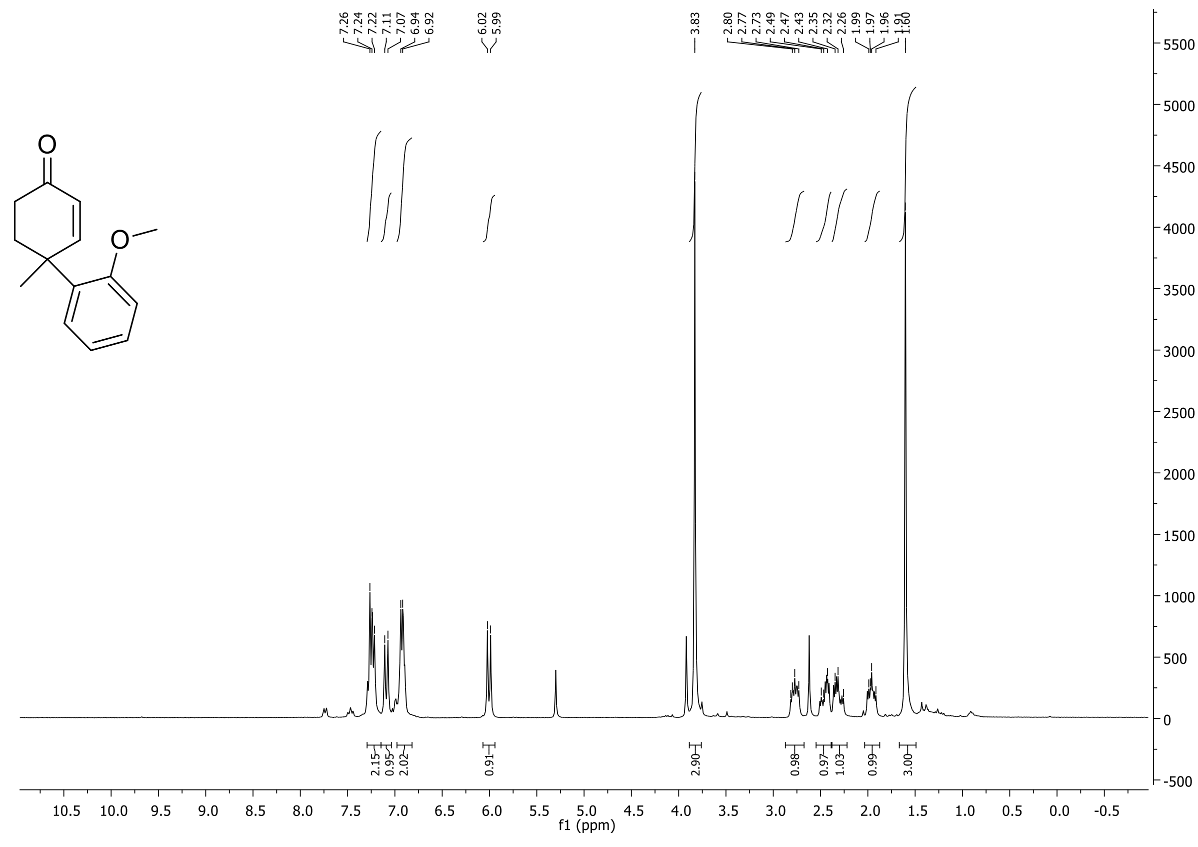

Figure S85. ^{1}H-NMR (300.36 MHz, $CDCl_3$) – **2'-Methoxy-1-methyl-2,3-dihydro-[1,1'-biphenyl]-4(1*H*)-one (rac-2q)**.

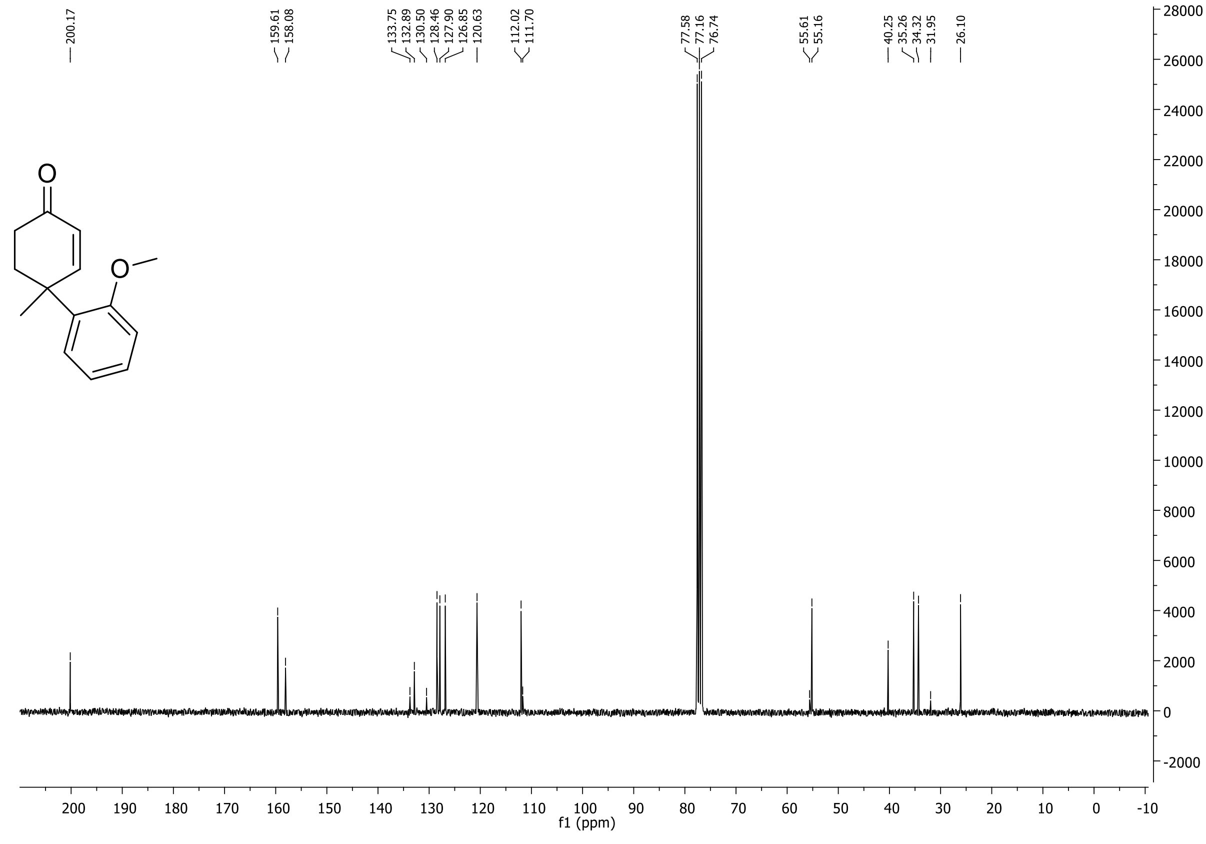

Figure S86. ^{13}C-NMR (75.53 MHz, $CDCl_3$) – **2'-Methoxy-1-methyl-2,3-dihydro-[1,1'-biphenyl]-4(1*H*)-one (rac-2q)**.

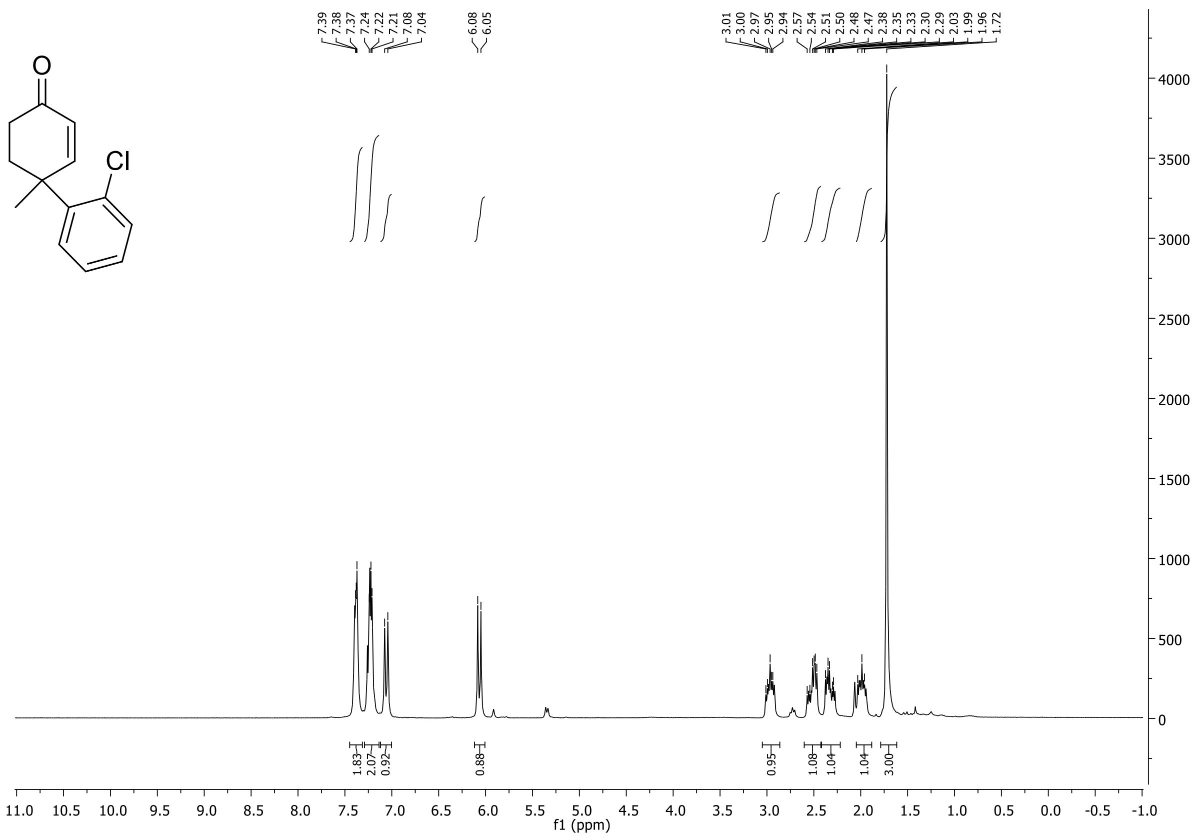

Figure S87. ^{1}H-NMR (300.36 MHz, $CDCl_3$) – **2’-Chloro-1-methyl-2,3-dihydro-[1,1’-biphenyl]-4-(1*H*)-one (rac-2r)**.

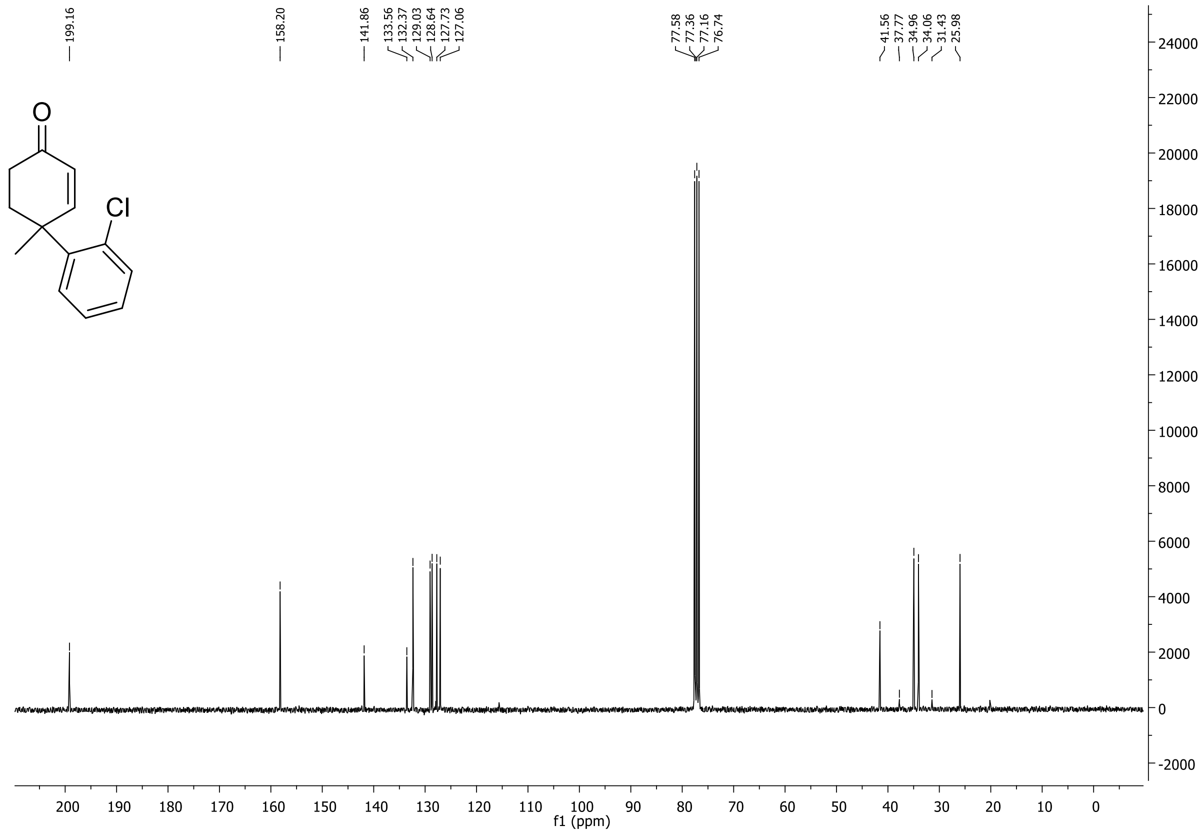

Figure S88. ^{13}C-NMR (75.53 MHz, $CDCl_3$) – **2’-Chloro-1-methyl-2,3-dihydro-[1,1’-biphenyl]-4-(1*H*)-one (rac-2r)**.

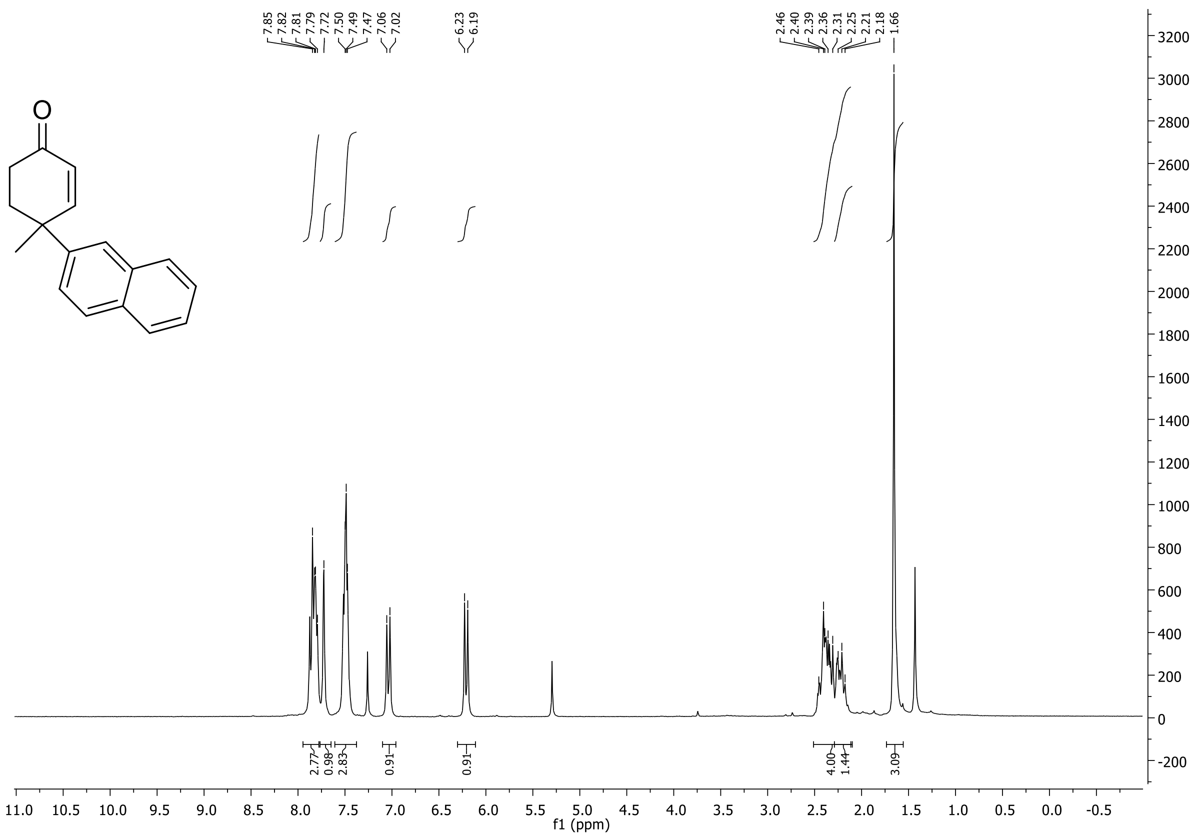

Figure S89. ^{1}H-NMR (300.36 MHz, $CDCl_3$) – **4-Methyl-4-(naphthalen-2-yl)cyclohex-2-enone (rac-2s)**.

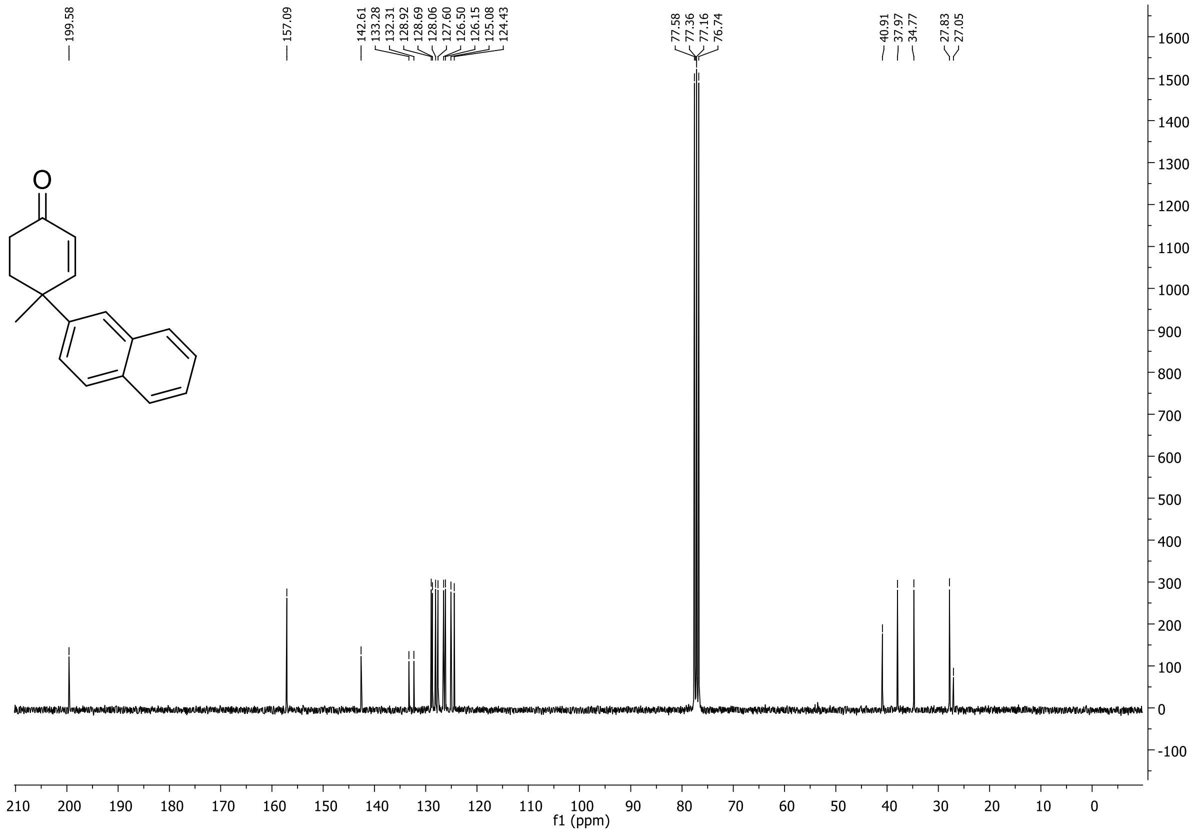

Figure S90. ^{13}C-NMR (75.53 MHz, $CDCl_3$) – **4-Methyl-4-(naphthalen-2-yl)cyclohex-2-enone (rac-2s)**.

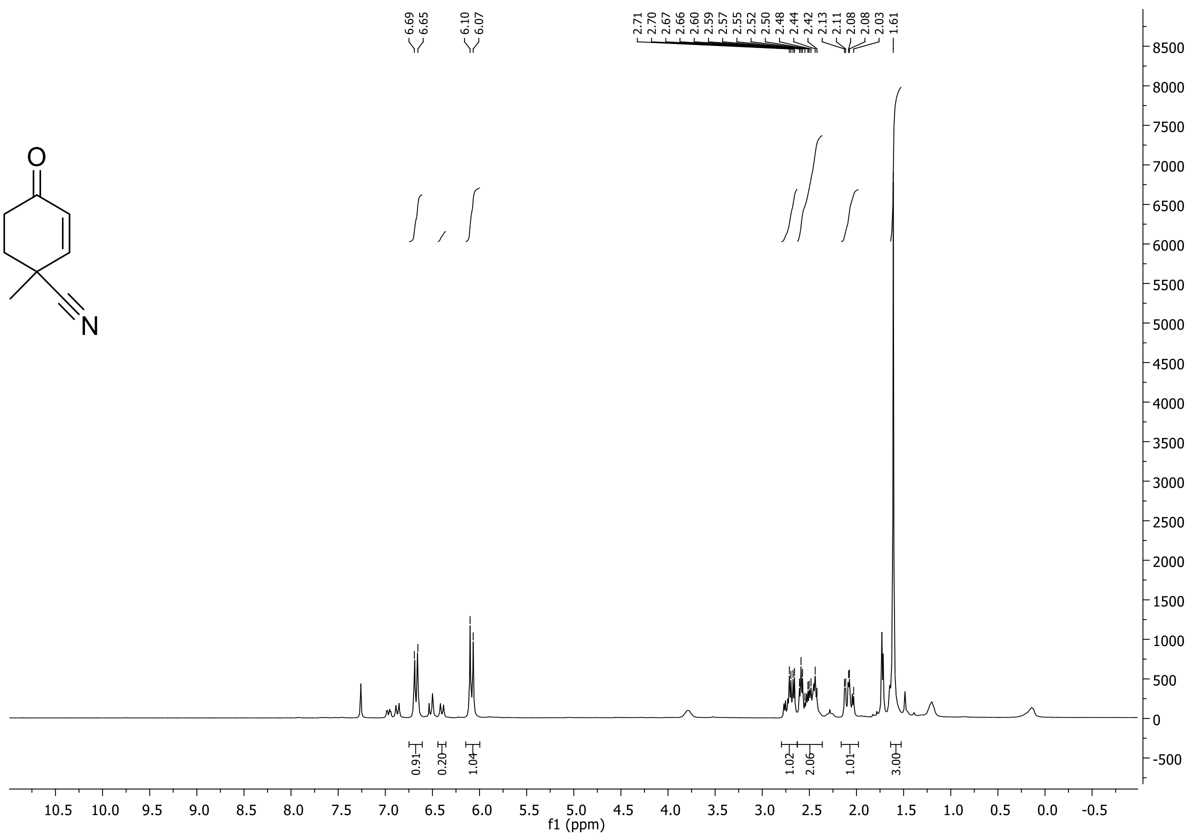

Figure S91. ^{1}H-NMR (300.36 MHz, $CDCl_3$) – **1-Methyl-4-oxocyclohex-2-enecarbonitrile (rac-2t)**.

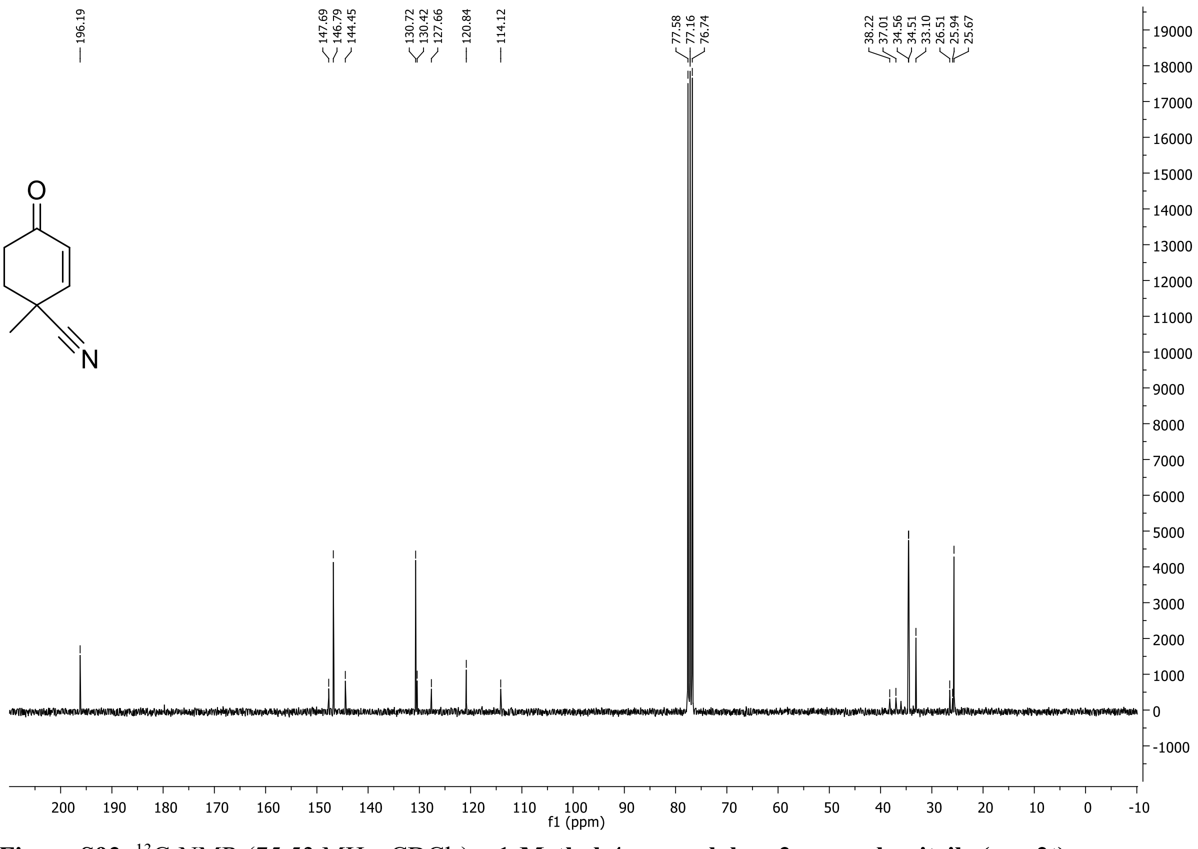

Figure S92. ^{13}C-NMR (75.53 MHz, $CDCl_3$) – **1-Methyl-4-oxocyclohex-2-enecarbonitrile (rac-2t)**.

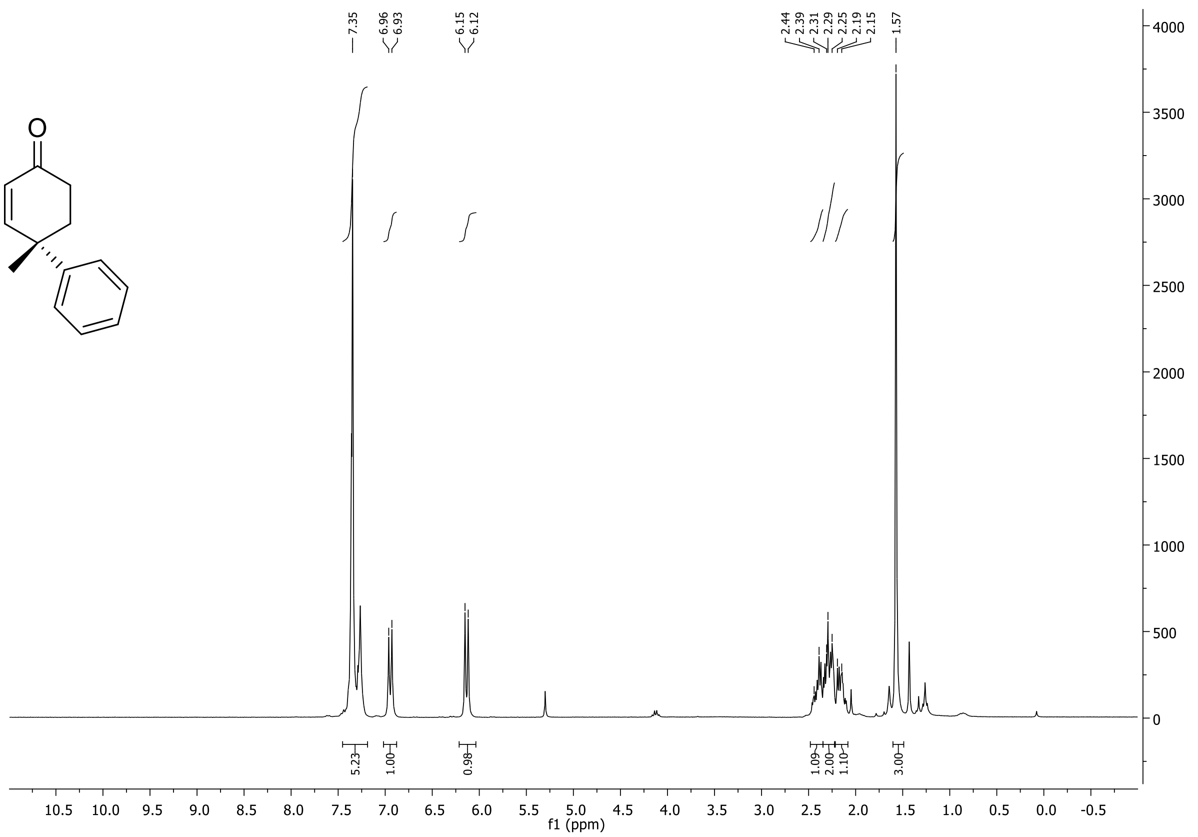

Figure S93. ^{1}H-NMR (300.36 MHz, $CDCl_3$) – **(*S*)-1-Methyl-2,3-dihydro-[1,1'-biphenyl]-4(1*H*)-one (2a)**.

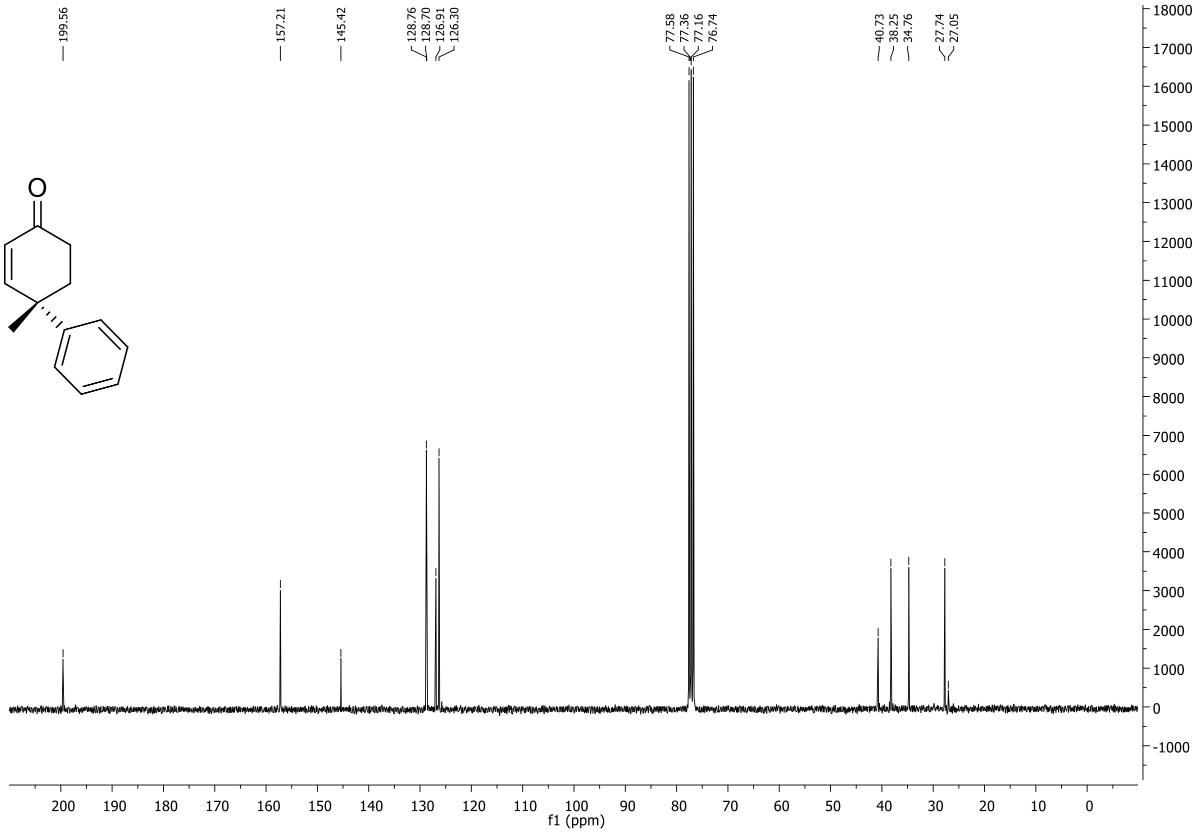

Figure S94. ^{13}C-NMR (75.53 MHz, $CDCl_3$) – **(*S*)-1-Methyl-2,3-dihydro-[1,1'-biphenyl]-4(1*H*)-one (2a)**.

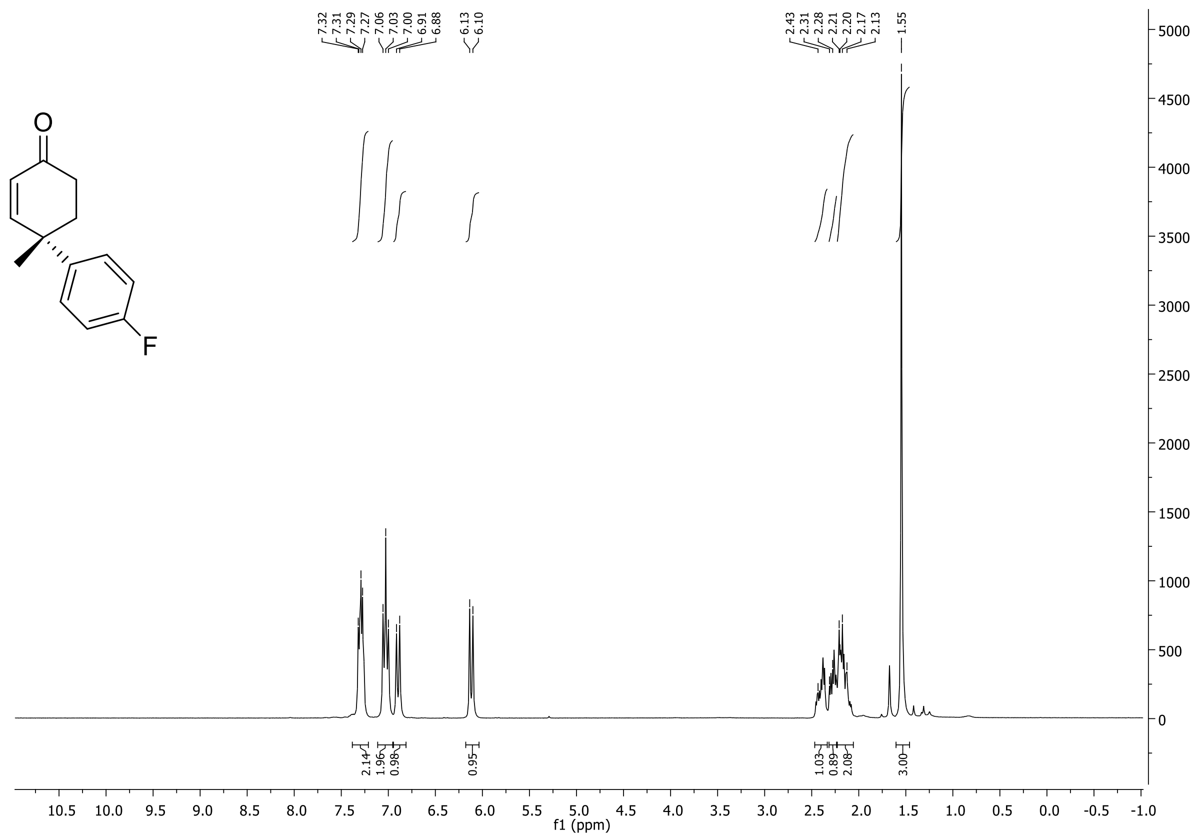

Figure S95. ^{1}H-NMR (300.36 MHz, $CDCl_3$) – **(*S*)-4’-Fluoro-1-methyl-2,3-dihydro-[1,1’-biphenyl]-4(1*H*)-one (2b)**.

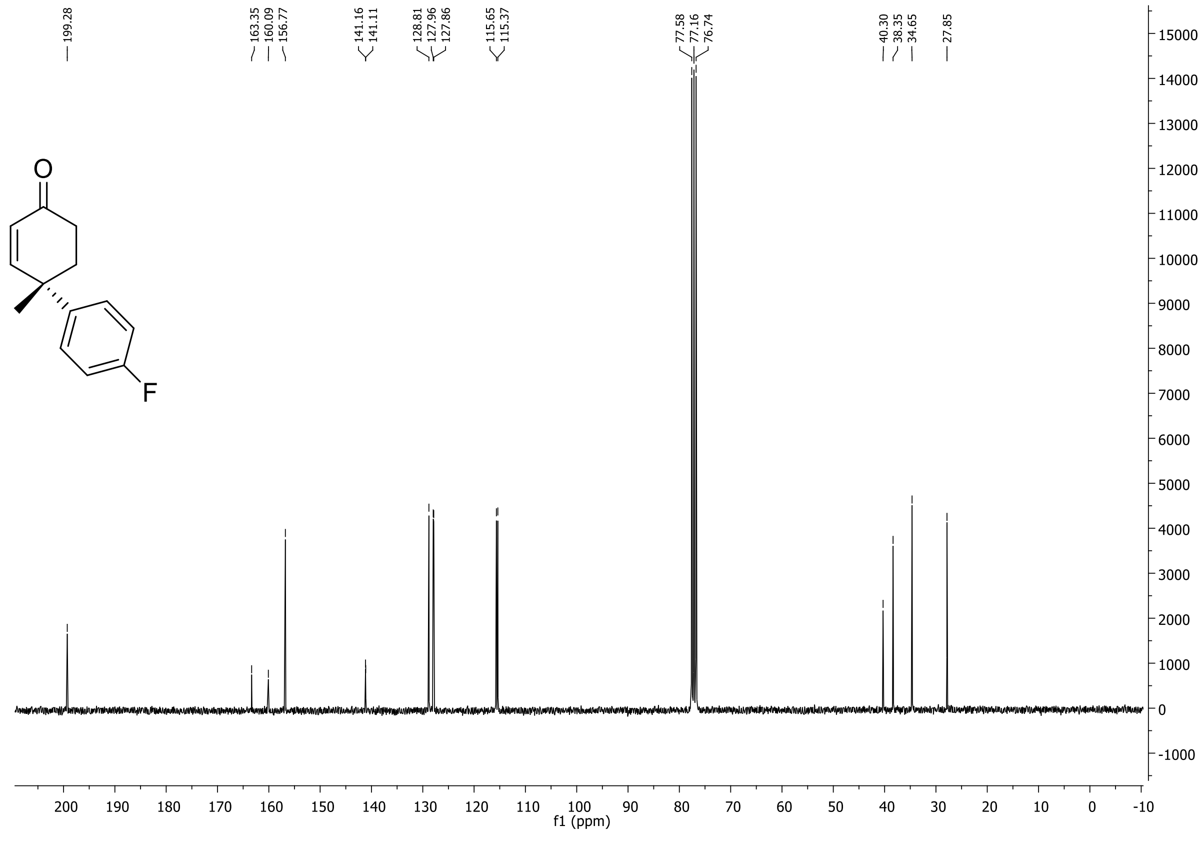

Figure S96. ^{13}C-NMR (75.53 MHz, $CDCl_3$) – **(*S*)-4’-Fluoro-1-methyl-2,3-dihydro-[1,1’-biphenyl]-4(1*H*)-one (2b)**.

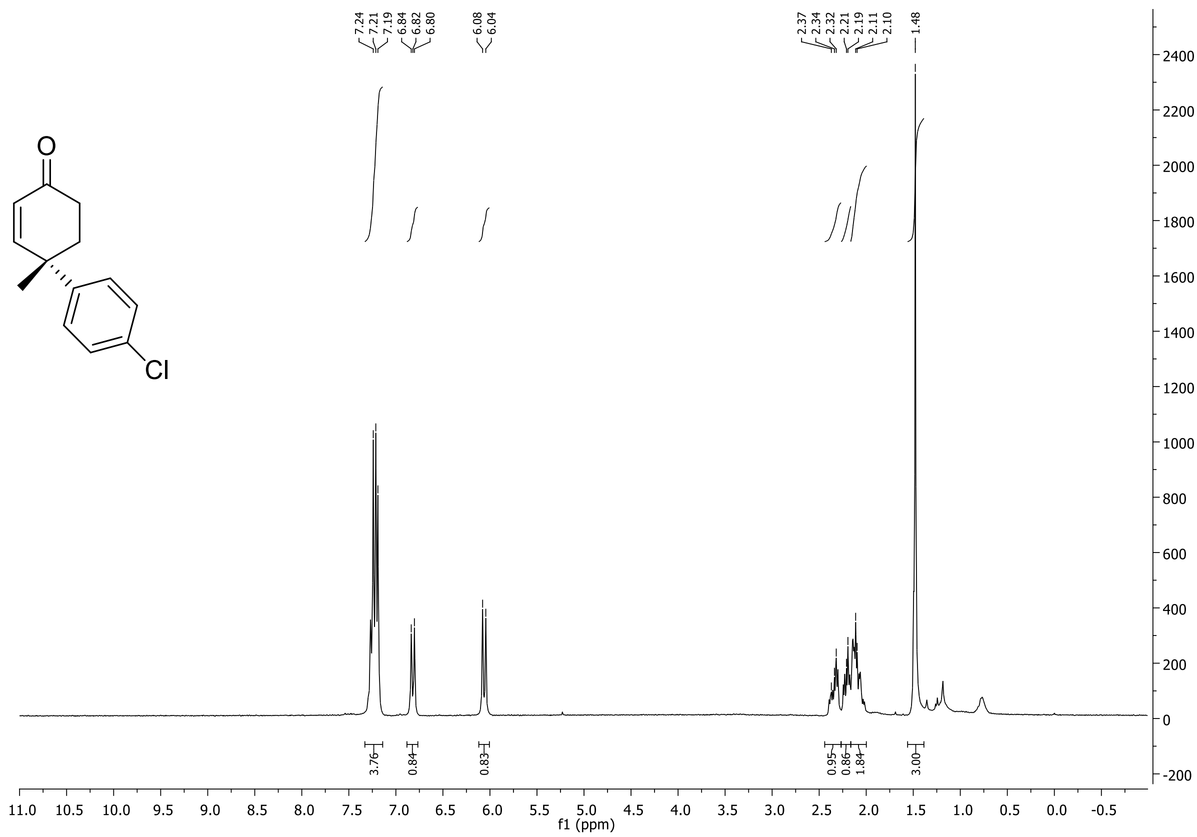

Figure S97. ^{1}H-NMR (300.36 MHz, $CDCl_3$) – **(*S*)-4'-Chloro-1-methyl-2,3-dihydro-[1,1'-biphenyl]-4(1*H*)-one (2c)**.

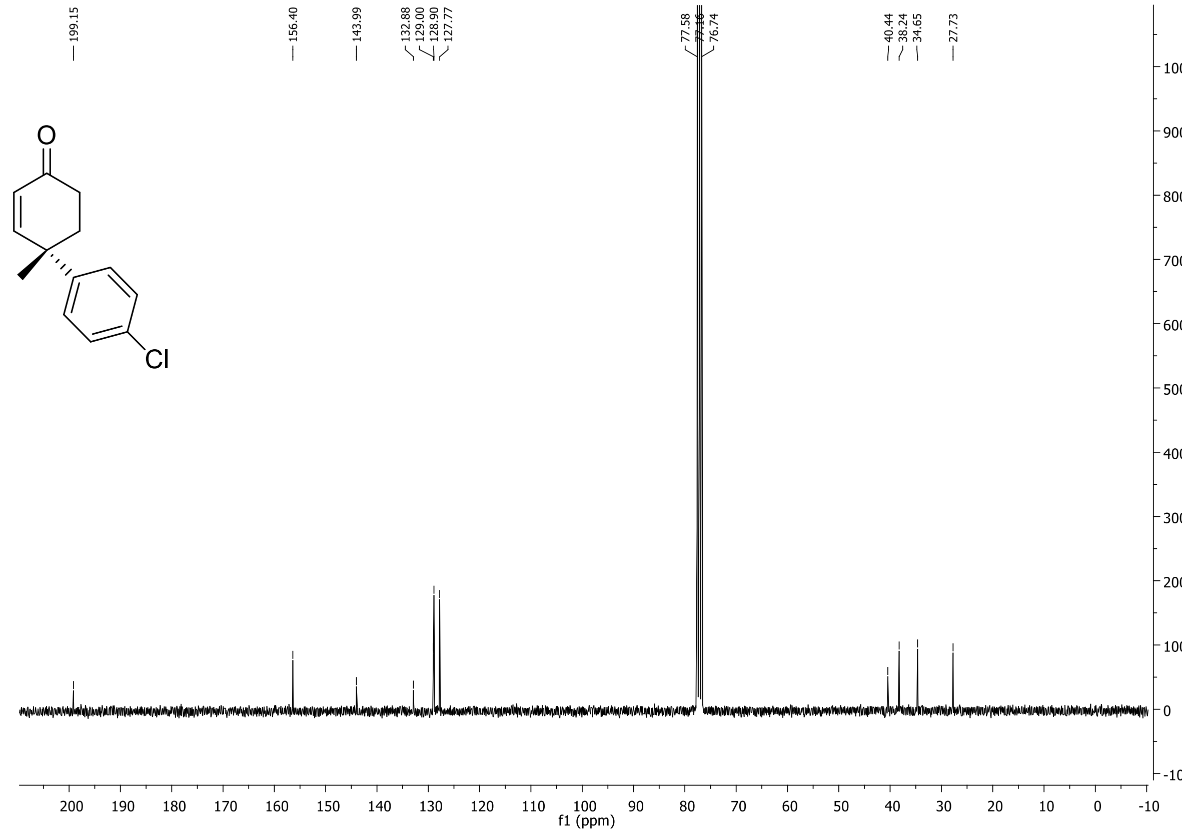

Figure S98. ^{13}C-NMR (75.53 MHz, $CDCl_3$) – **(*S*)-4'-Chloro-1-methyl-2,3-dihydro-[1,1'-biphenyl]-4(1*H*)-one (2c)**.

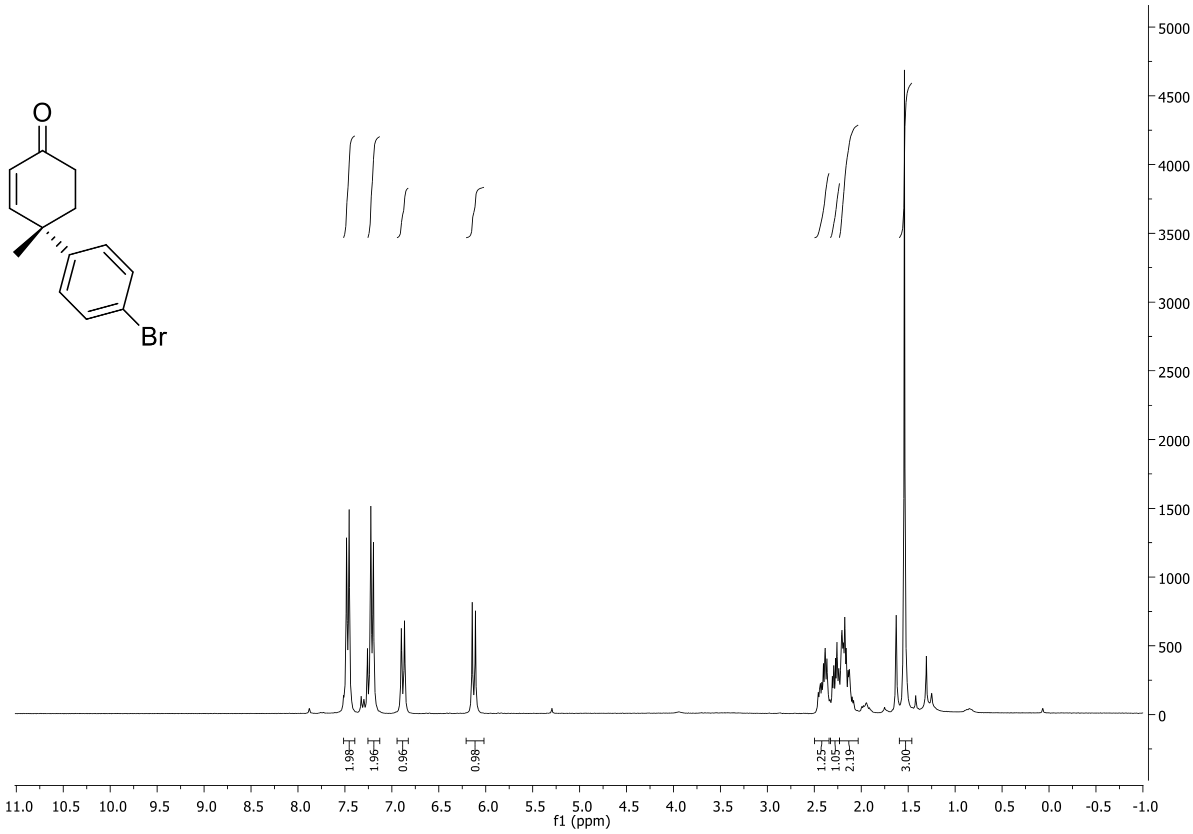

Figure S99. ^{1}H-NMR (300.36 MHz, $CDCl_3$) – **(*S*)-4'-Bromo-1-methyl-2,3-dihydro-[1,1'-biphenyl]-4(1*H*)-one (2d)**.

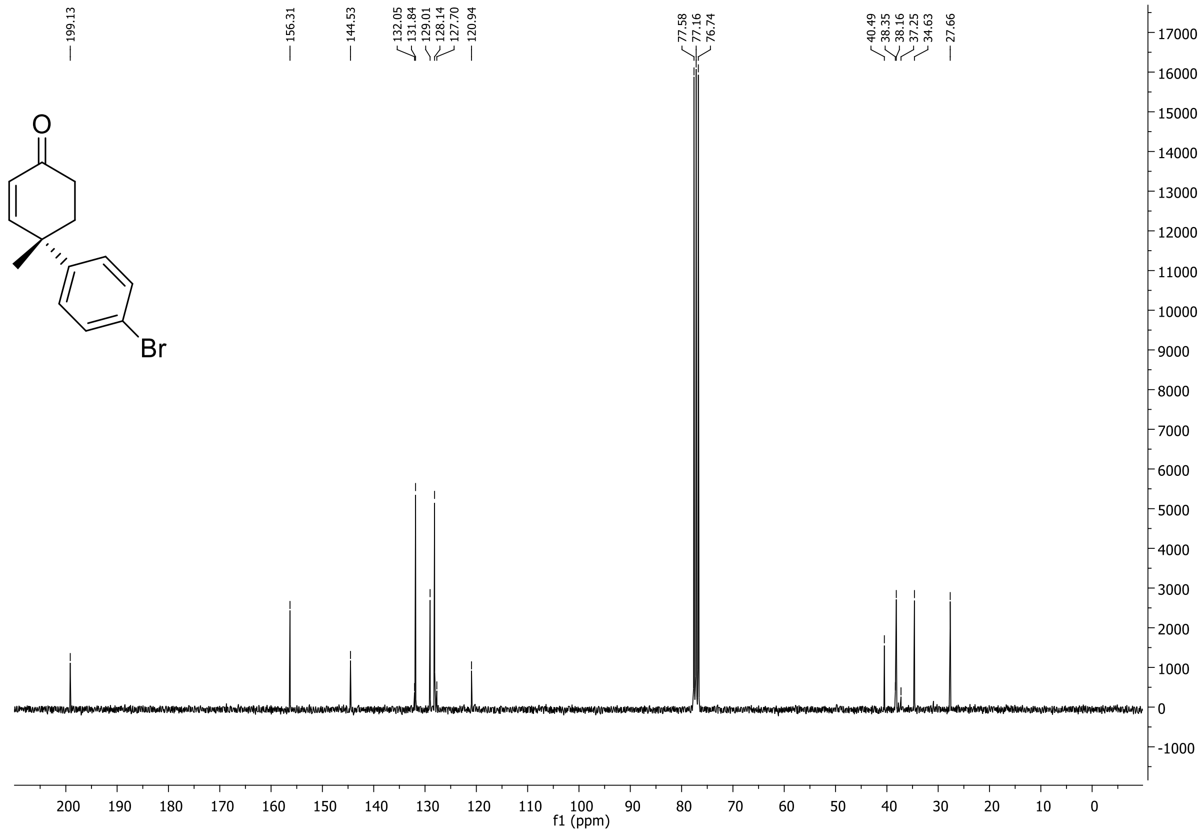

Figure S100. ^{13}C-NMR (75.53 MHz, $CDCl_3$) – **(*S*)-4'-Bromo-1-methyl-2,3-dihydro-[1,1'-biphenyl]-4(1*H*)-one (2d)**.

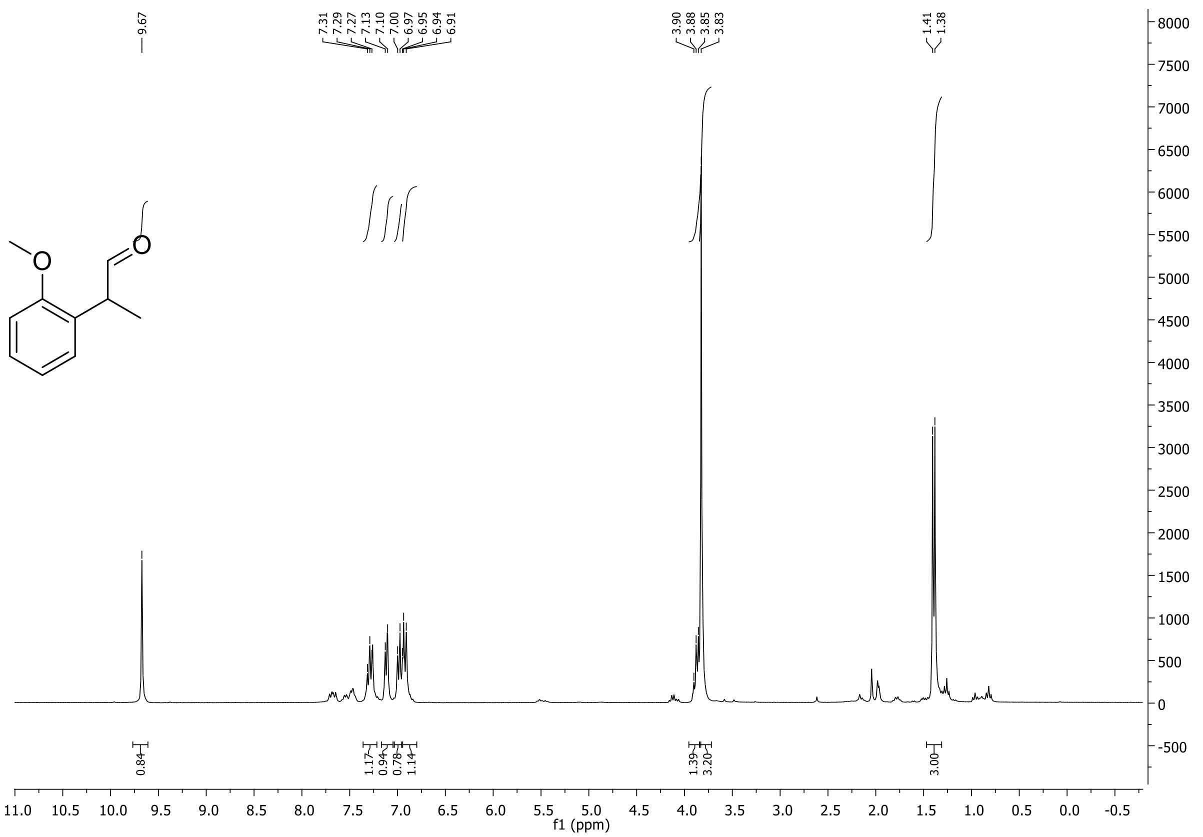

Figure S101. ^{1}H-NMR (300.36 MHz, $CDCl_3$) – **2-(2-Methoxyphenyl)propanal (3q)**.

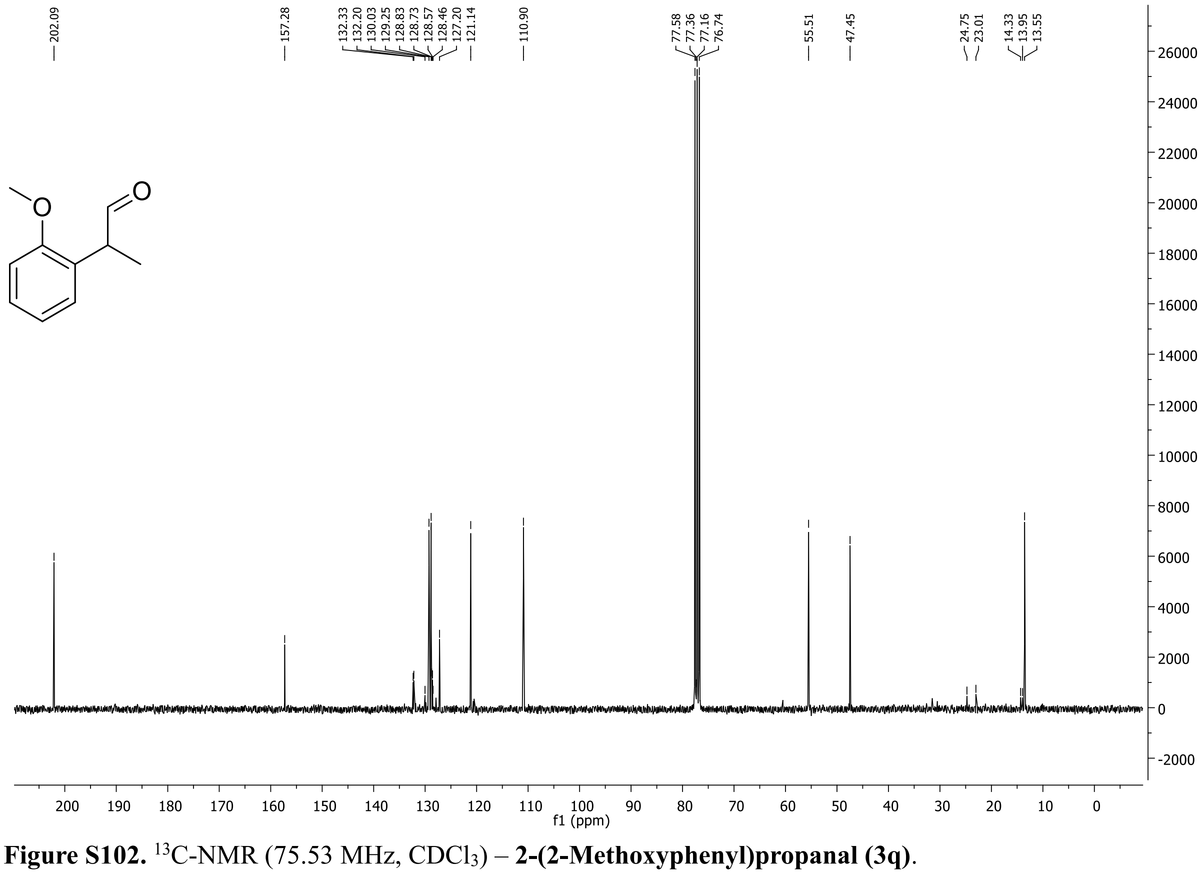

Figure S102. ^{13}C-NMR (75.53 MHz, $CDCl_3$) – **2-(2-Methoxyphenyl)propanal (3q)**.

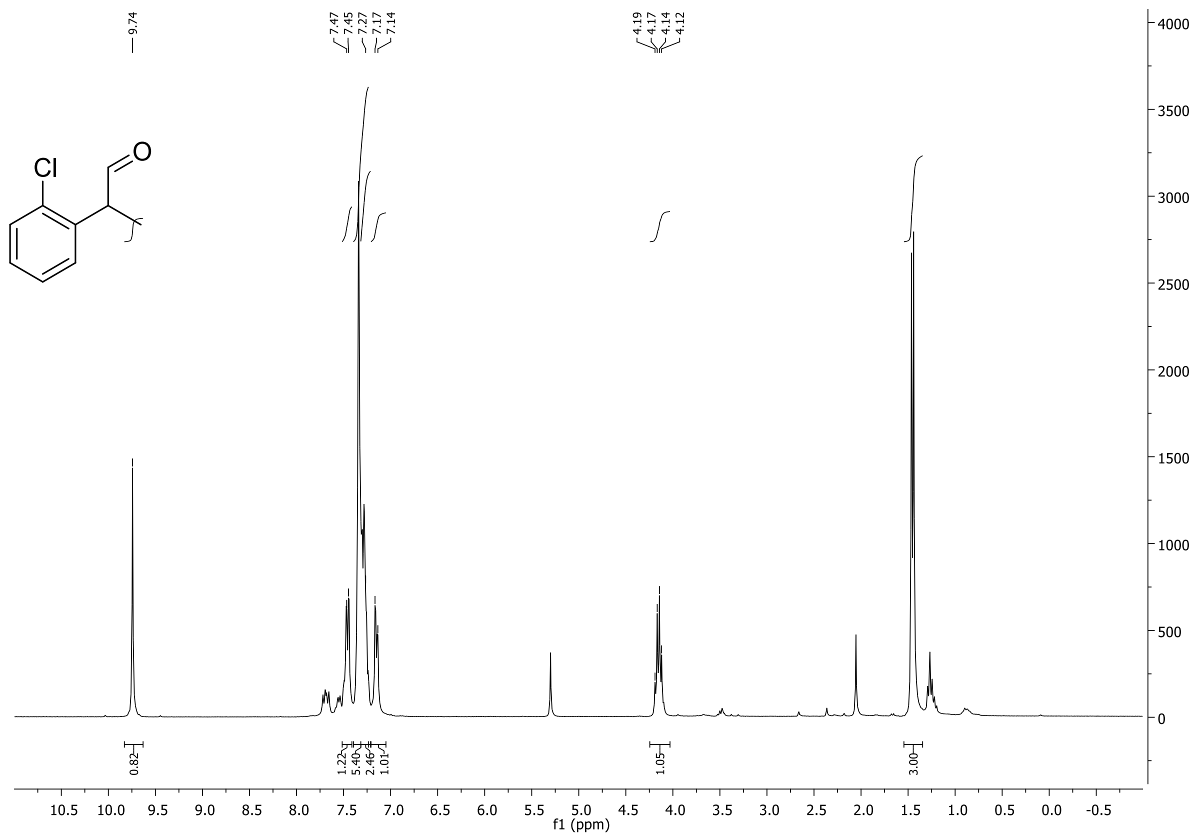

Figure S103. ^{1}H-NMR (300.36 MHz, $CDCl_3$) – **(2-Chlorophenyl)propanal (3r)**.

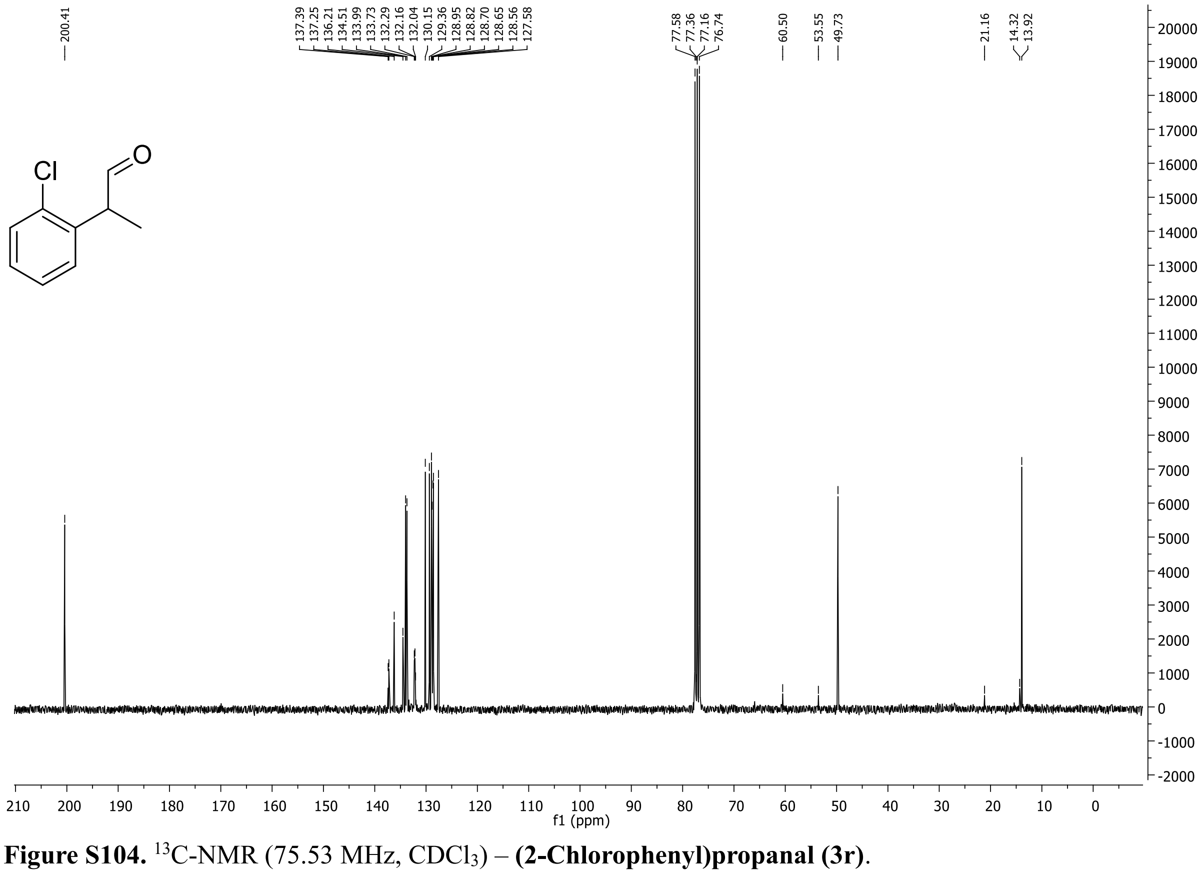

Figure S104. ^{13}C-NMR (75.53 MHz, $CDCl_3$) – **(2-Chlorophenyl)propanal (3r)**.

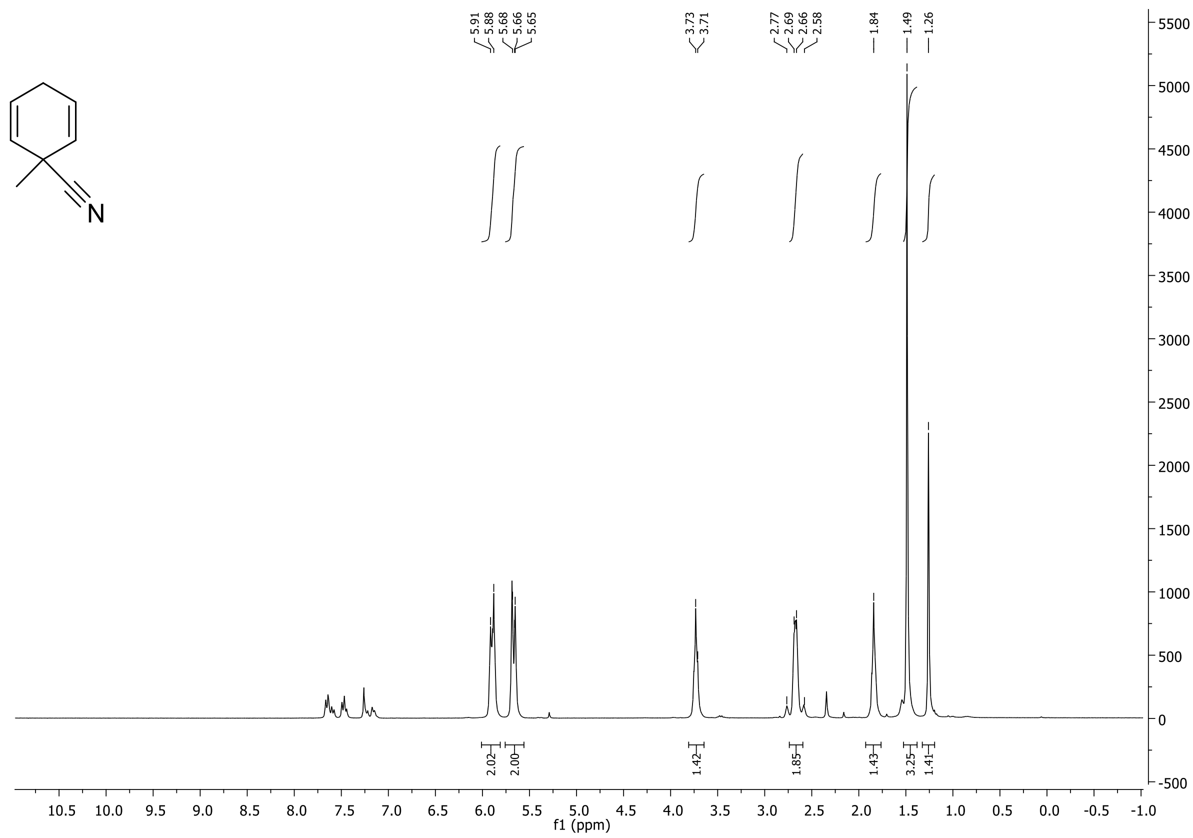

Figure S105. ^{1}H-NMR (300.36 MHz, $CDCl_3$) – **1-Methylcyclohexa-2,5-diene-1-carbonitrile (3t)**.

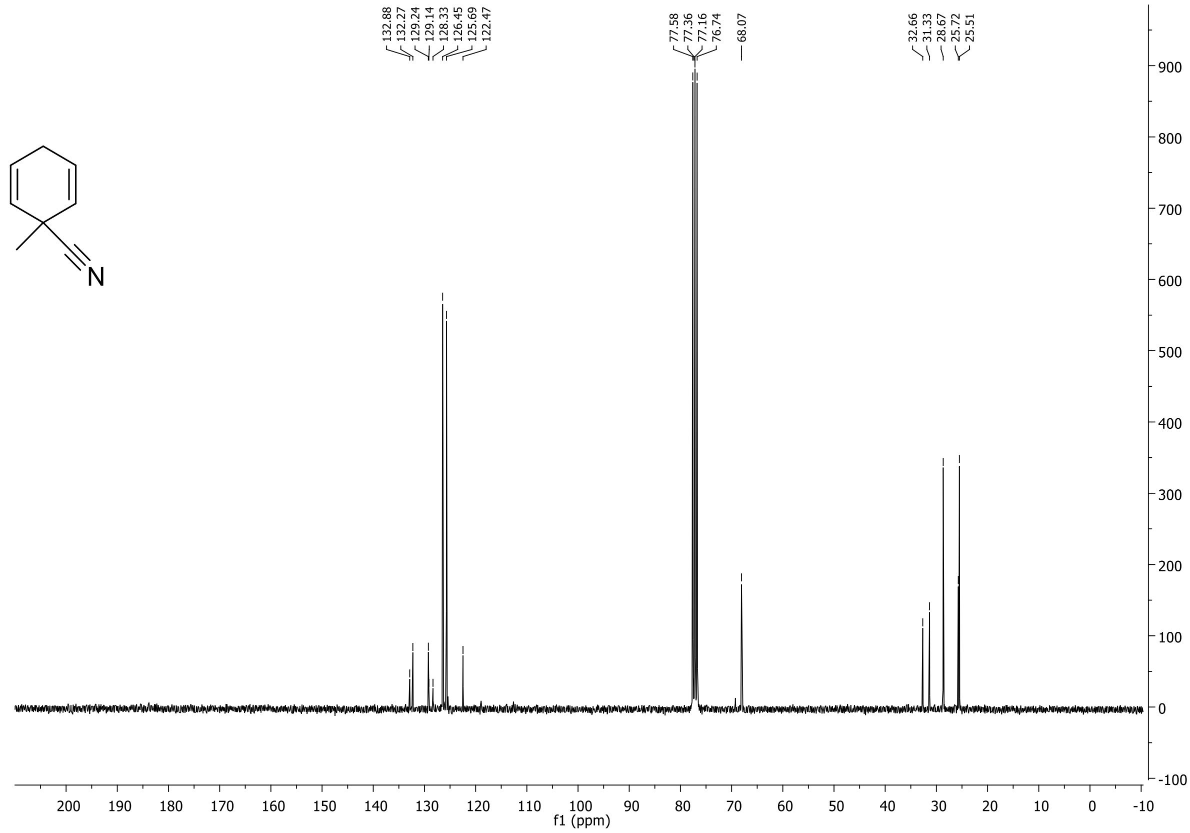

Figure S106. ^{13}C-NMR (75.53 MHz, $CDCl_3$) – **1-Methylcyclohexa-2,5-diene-1-carbonitrile (3t)**.

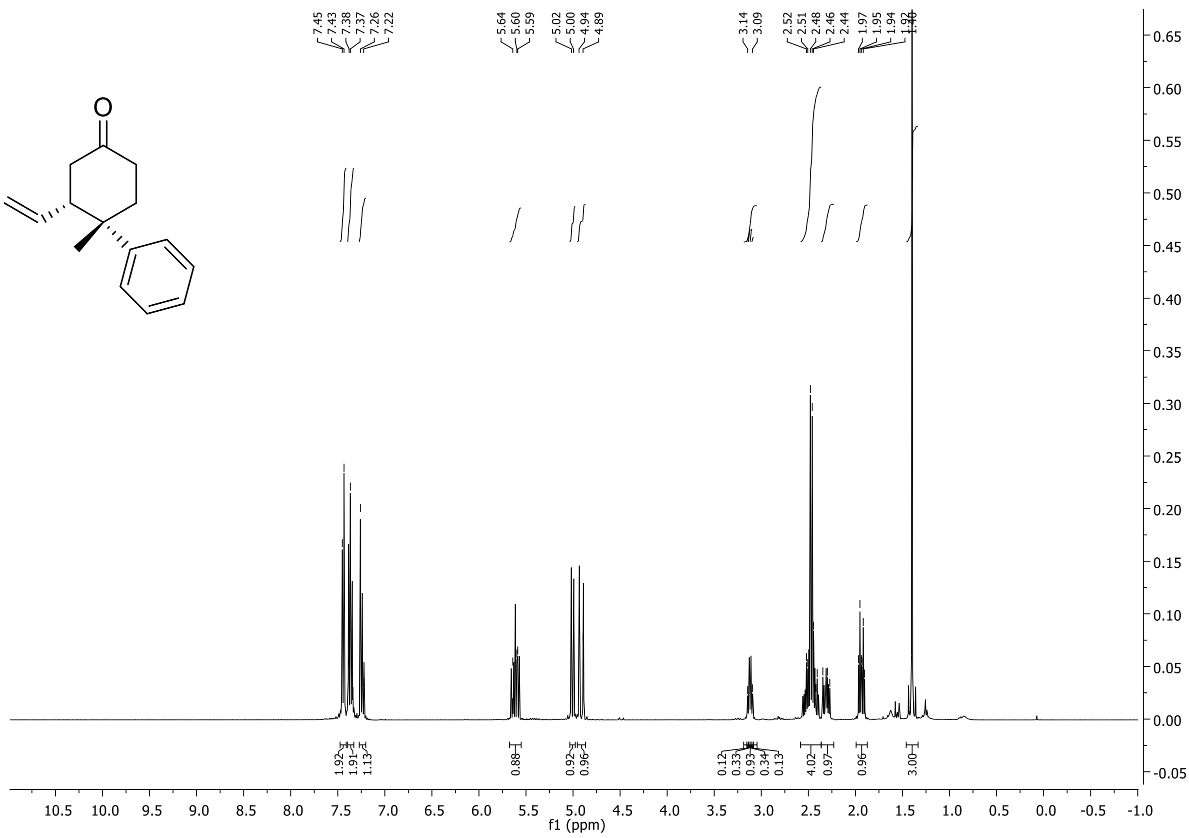

Figure S107. ^{1}H-NMR (399.78 MHz, $CDCl_3$) – **(3*S*,4*S*)-4-Methyl-4-phenyl-3-vinylcyclohexanone (4)**.

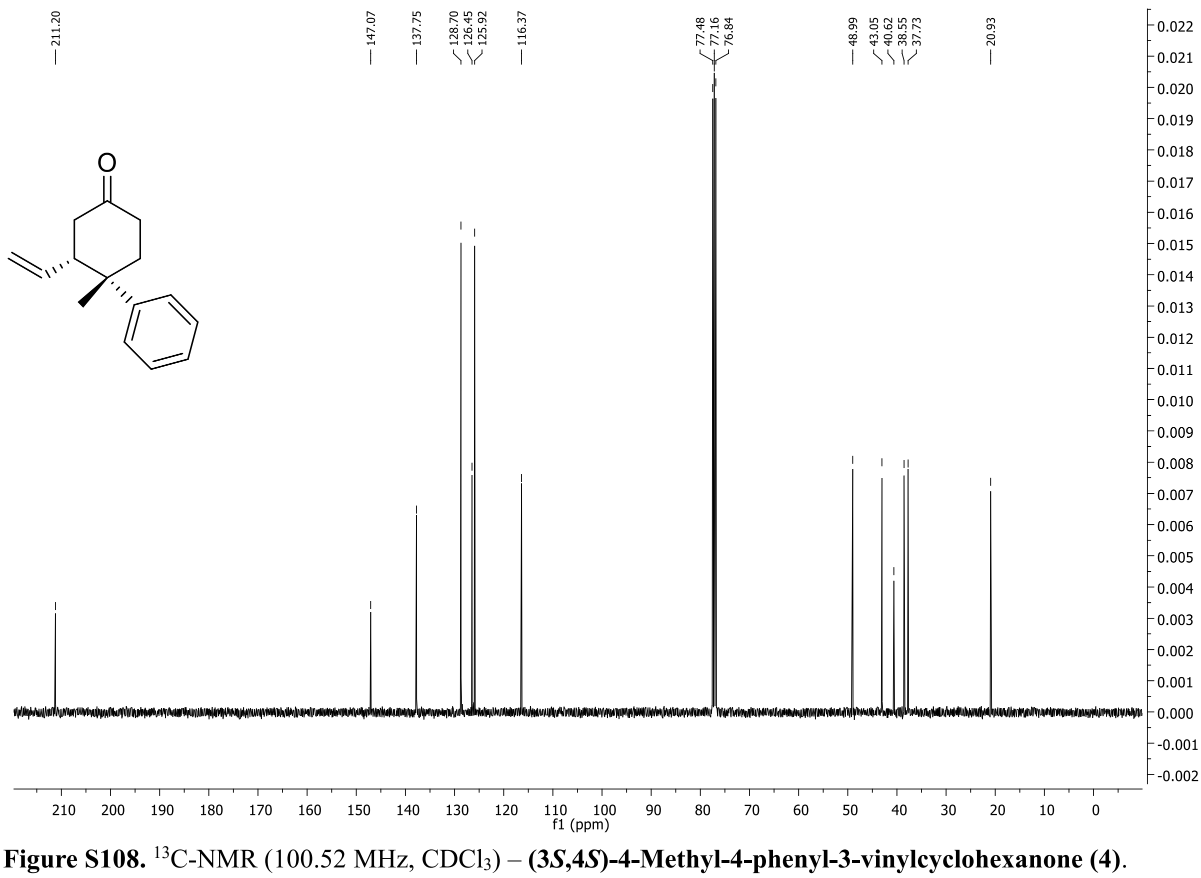

Figure S108. ^{13}C-NMR (100.52 MHz, $CDCl_3$) – **(3*S*,4*S*)-4-Methyl-4-phenyl-3-vinylcyclohexanone (4)**.

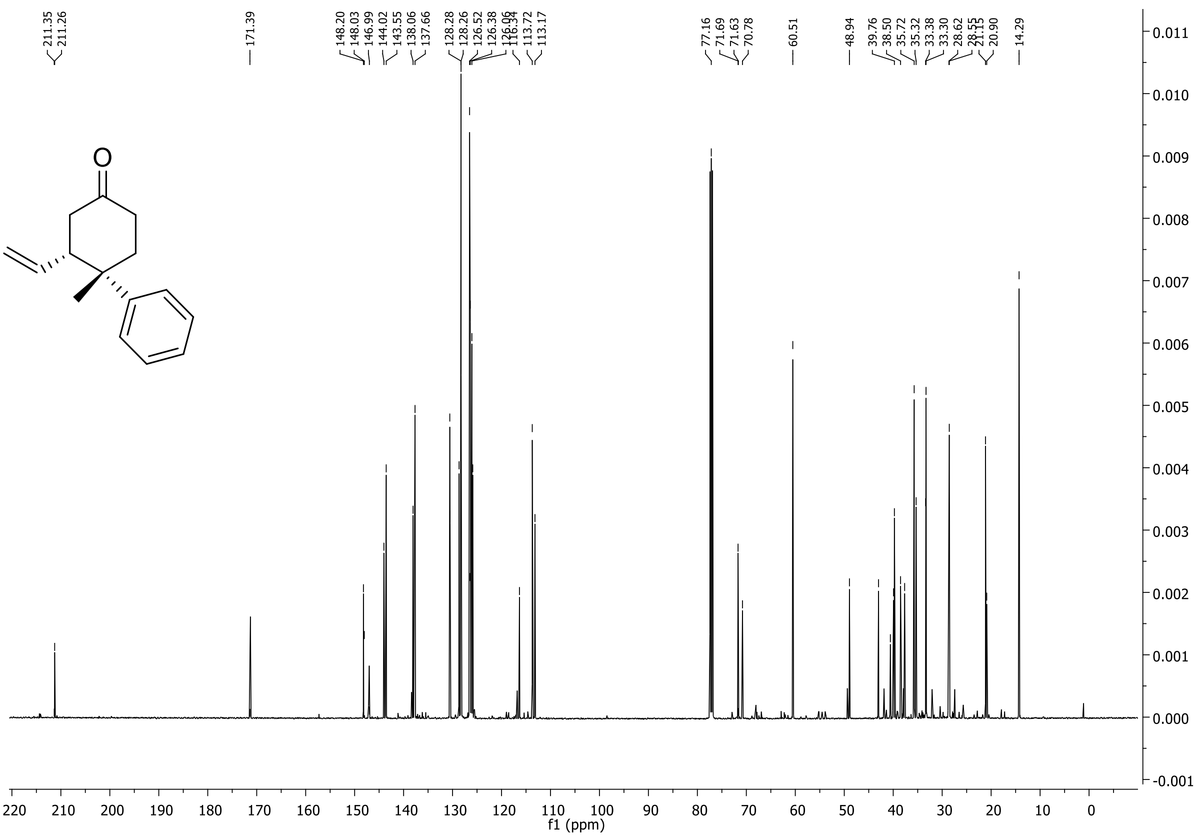

Figure S109. Crude ^{13}C-NMR (125.65 MHz, $CDCl_3$) – **(3*S*,4*S*)-4-Methyl-4-phenyl-3-vinylcyclohexanone (4)**.

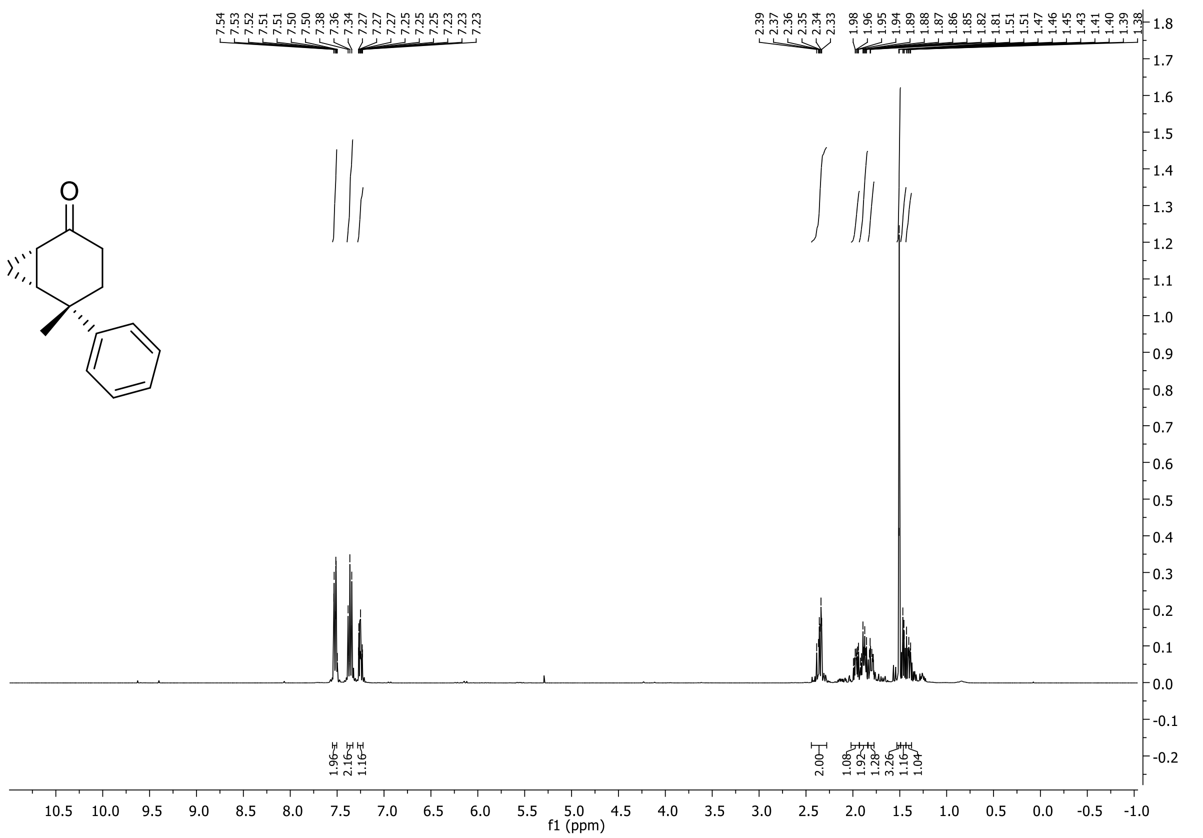

Figure S110. ^{1}H-NMR (399.78 MHz, $CDCl_3$) – **(1*S*,5*S*,6*R*)-5-Methyl-5-phenylbicyclo[4.1.0]heptan-2-one (5)**.

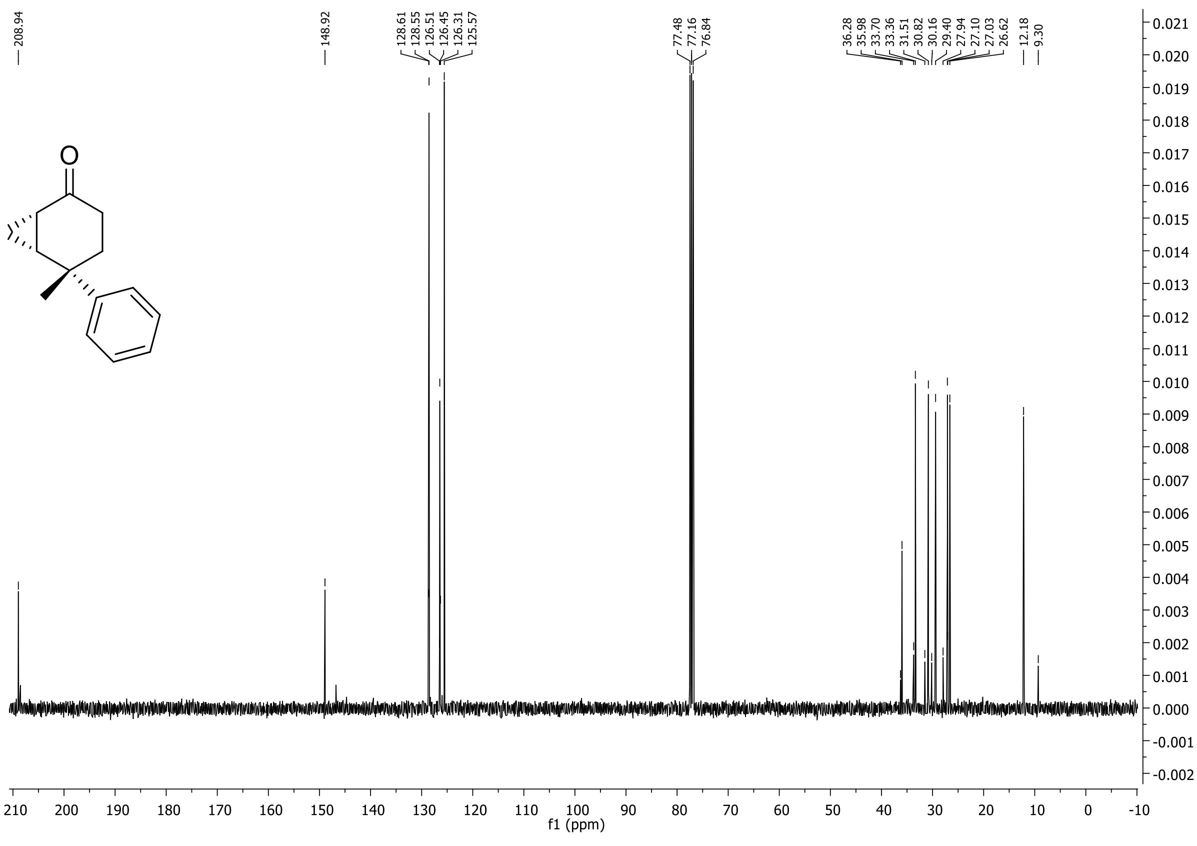

Figure S111. ^{13}C-NMR (100.52 MHz, $CDCl_3$) – **(1*S*,5*S*,6*R*)-5-Methyl-5-phenylbicyclo[4.1.0]heptan-2-one (5)**.

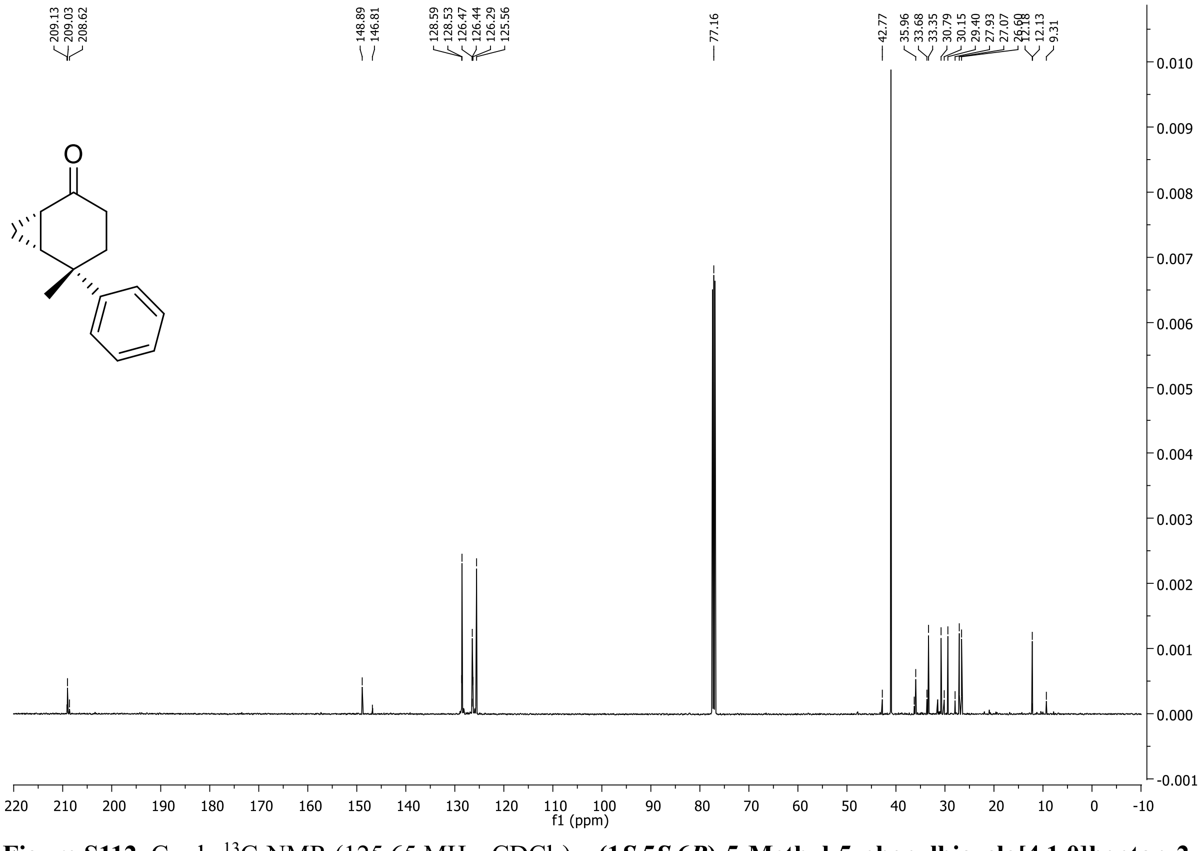

Figure S112. Crude ^{13}C-NMR (125.65 MHz, $CDCl_3$) – **(1*S*,5*S*,6*R*)-5-Methyl-5-phenylbicyclo[4.1.0]heptan-2-one (5)**.

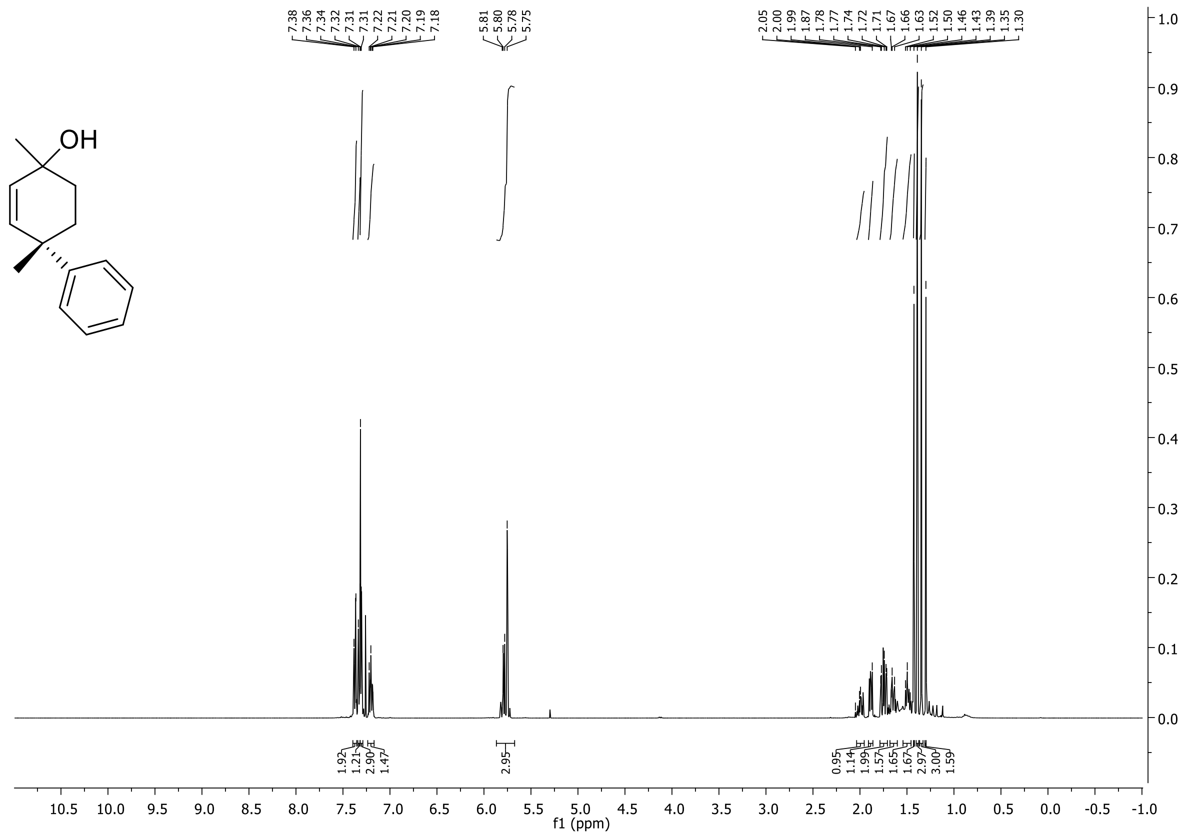

Figure S113. ^{1}H-NMR (399.78 MHz, $CDCl_3$) – **(1*S*)-1,4-Dimethyl-1,2,3,4-tetrahydro-[1,1'-biphenyl]-4-ol (6)**.

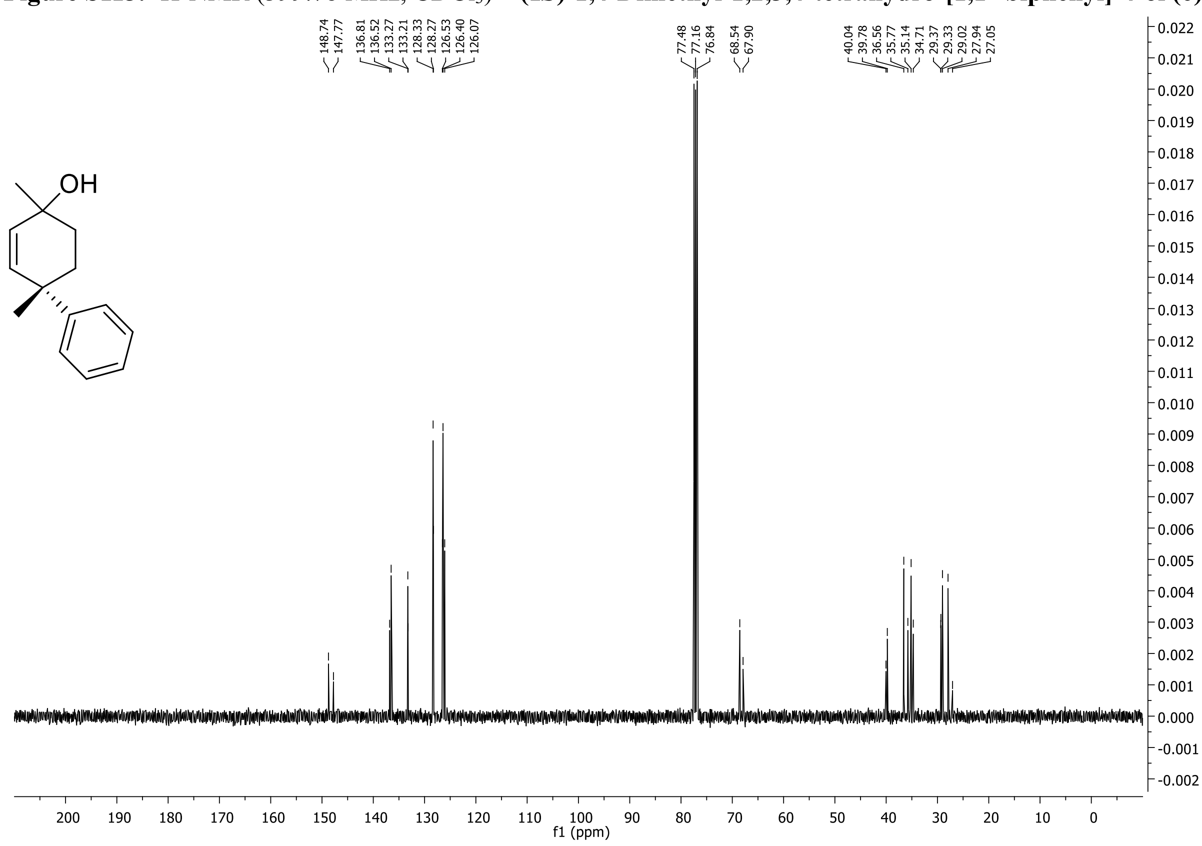

Figure S114. ^{13}C-NMR (100.52 MHz, $CDCl_3$) – **(1*S*)-1,4-Dimethyl-1,2,3,4-tetrahydro-[1,1'-biphenyl]-4-ol (6)**.

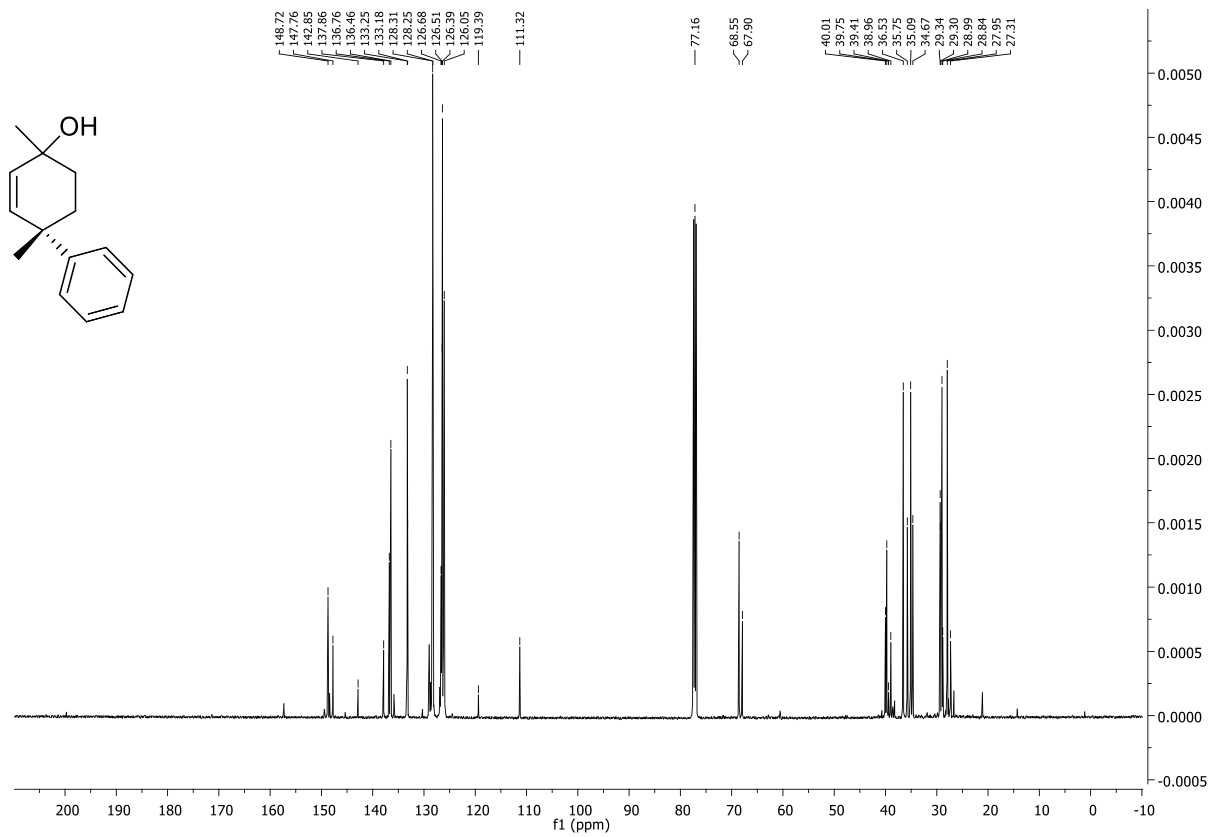

Figure S115. Crude ^{13}C-NMR (125.65 MHz, $CDCl_3$) – **(1*S*)-1,4-Dimethyl-1,2,3,4-tetrahydro-[1,1'-biphenyl]-4-ol (6)**.

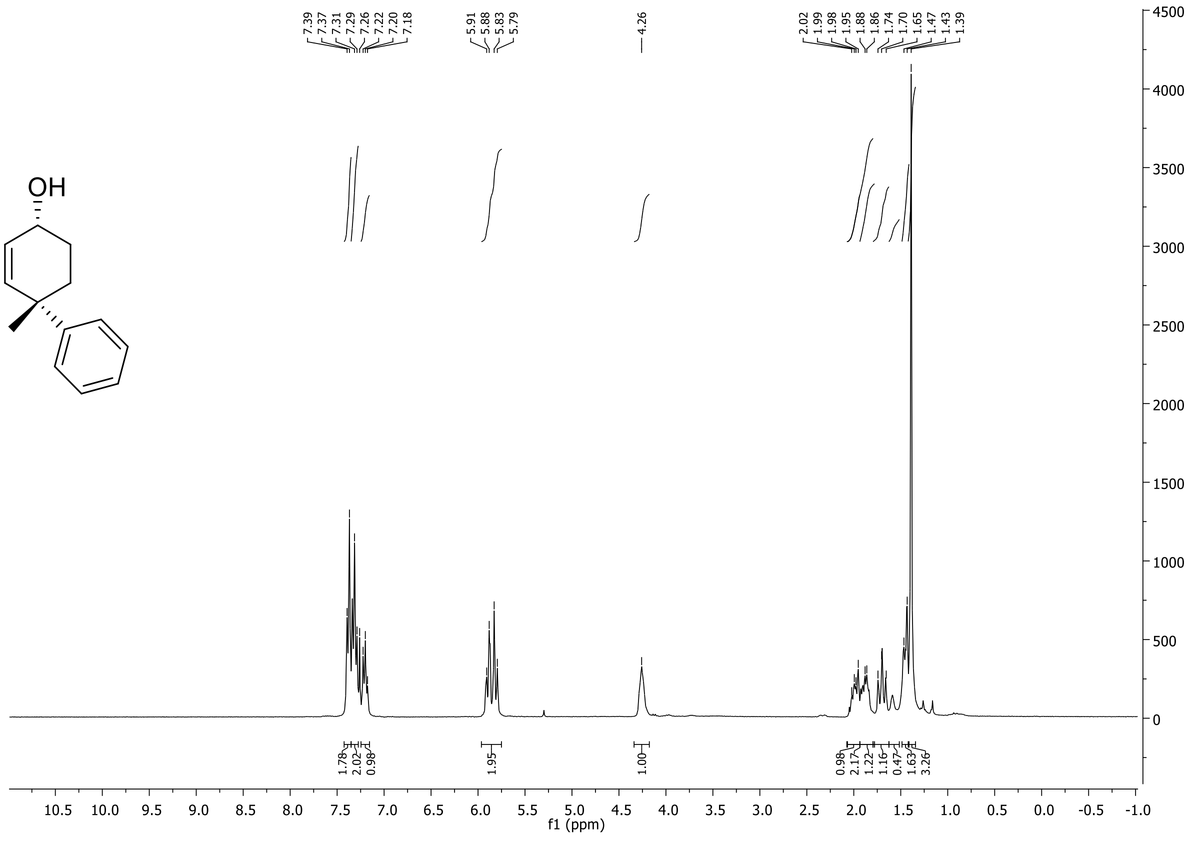

Figure S116. ^{1}H-NMR (300.36 MHz, $CDCl_3$) – **(1*S*,4*R*)-1-Methyl-1,2,3,4-tetrahydro-[1,1'-biphenyl]-4-ol (7)**.

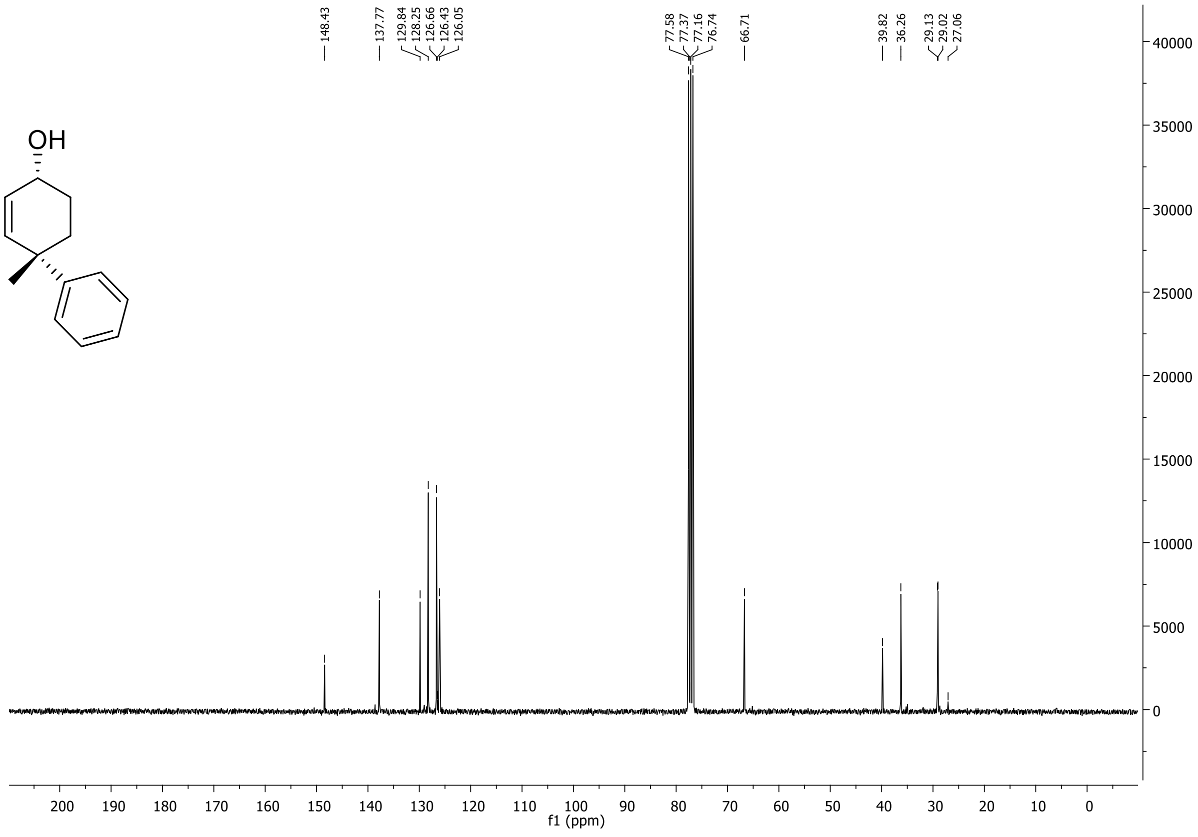

Figure S117. ^{13}C-NMR (75.53 MHz, $CDCl_3$) – **(1*S*,4*R*)-1-Methyl-1,2,3,4-tetrahydro-[1,1'-biphenyl]-4-ol (7)**.

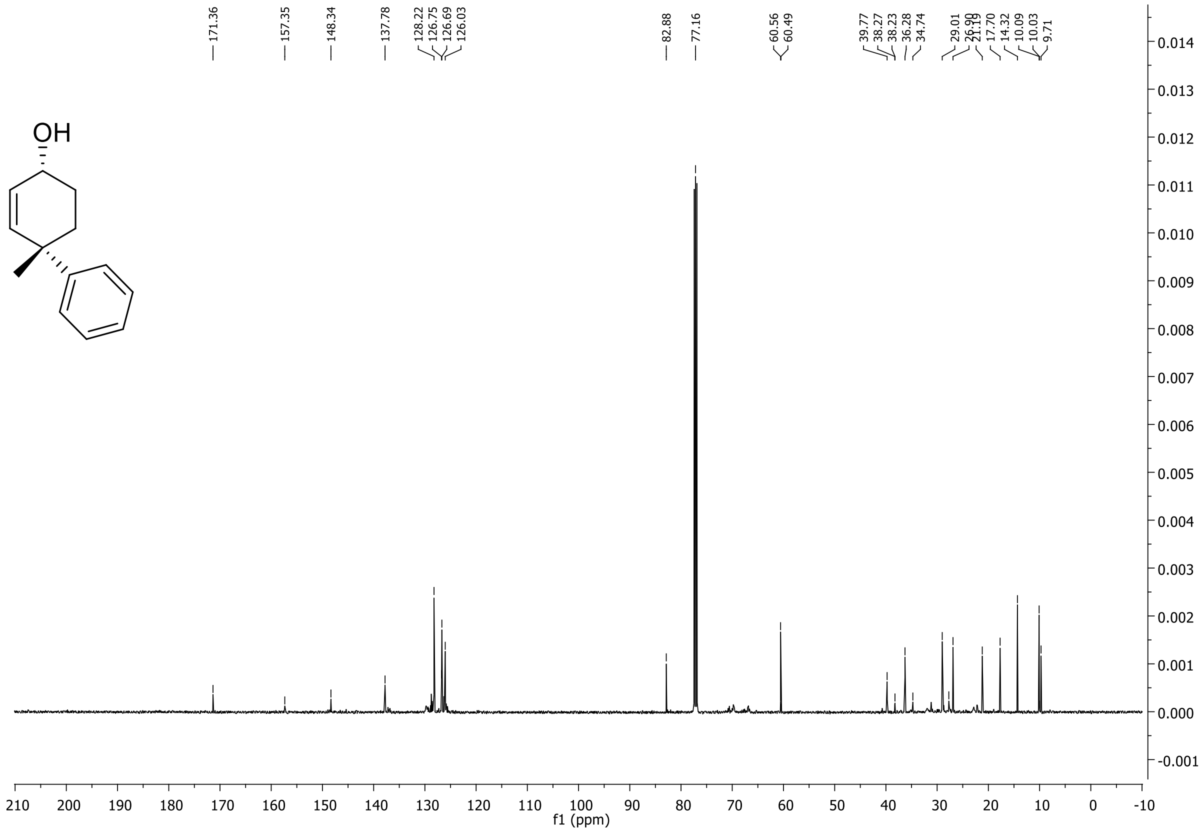

Figure S118. Crude ^{13}C-NMR (125.65 MHz, $CDCl_3$) – **(1*S*,4*R*)-1-Methyl-1,2,3,4-tetrahydro-[1,1'-biphenyl]-4-ol (7)**.
